# Catalytic three-component C–C bond forming dearomatization of bromoarenes with malonates and diazo compounds†

**DOI:** 10.1039/d0sc02881a

**Published:** 2020-07-29

**Authors:** Hiroki Kato, Itsuki Musha, Masaaki Komatsuda, Kei Muto, Junichiro Yamaguchi

**Affiliations:** Department of Applied Chemistry, Waseda University 3-4-1, Ohkubo, Shinjuku Tokyo 169-8555 Japan junyamaguchi@waseda.jp; Waseda Institute for Advanced Study, Waseda University Shinjuku Tokyo 169-8050 Japan keimuto@aoni.waseda.jp

## Abstract

A Pd-catalyzed dearomative three-component C–C bond formation of bromoarenes with diazo compounds and malonates was developed. Various bromoarenes ranging from benzenoids to azines and heteroles were transformed to the corresponding substituted alicyclic molecules. The key to this reaction is the generation of a benzyl–palladium intermediate, which reacts with malonates to form a Pd–*O*-enolate species. Strikingly, the present method enabled rapid access to multi-substituted alicycles through subsequent elaboration of dearomatized products.

## Abstract 2

A catalytic three-component C–C bond forming dearomatization of bromoarenes was developed, enabling rapid access to multi-substituted alicycles.

## Graphical abstract

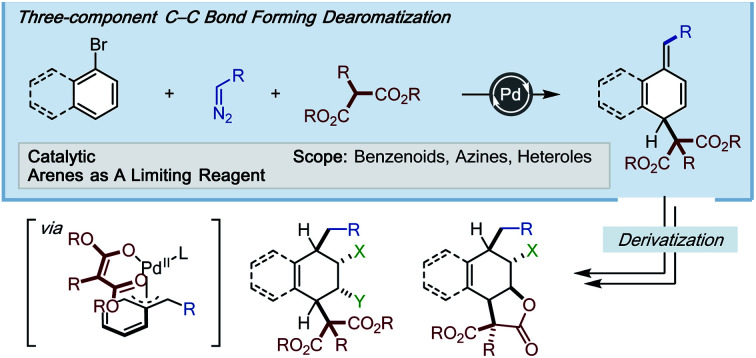

## Introduction

Dearomatization is a powerful method to generate molecular complexity, because this transformation can convert two-dimensional chemical feedstock arenes to highly valuable three-dimensional (alicyclic) architectures. Because of its potential, the development of dearomative methods is a topic of intense study in organic synthesis.^1^ Birch reduction^2^ and metal-catalyzed hydrogenations^3^ are the most established transformations, as well as oxidative dearomatizations. However, as a drawback, the vast majority of dearomative functionalizations rely on the electronic nature of the parent arenes.^4^ The dearomative reactions of non-activated arenes such as benzenes and naphthalenes have often seen a lack of reaction efficiency, requiring stoichiometric amounts of metal reagents and excess substrates.^5^ Although a few dearomative reactions of inactive arenes as a limiting agent (including catalytic fashion) have recently emerged, the development of dearomative methods of a new class of arenes is still highly valuable.^6,7^

**Fig. 1:**
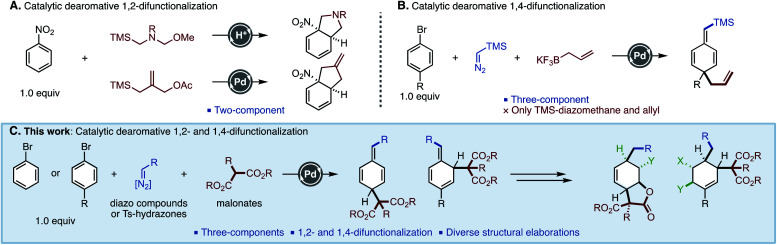
Catalytic dearomative difunctionalization of benzenoids. (A) 1,2-Difunctionalization of nitroarenes. (B) Three-component 1,4-difunctionalization of bromoarenes. (C) Three-component 1,2- and 1,4-difunctionalizations of bromoarenes.

Dearomative difunctionalization of benzenoids is a useful and step-economical methodology to build three-dimensional carbon frameworks. As representative examples using benzenoids as a limiting reagent, photo-induced intramolecular cycloadditions,^8^ reactions of metal–arene complexes,^9^ and nucleophilic dearomatizations^7,10^ have been developed. Despite these advances, they still require the tedious preparation of metal–arene complexes and the presence of specific substituents such as oxazolines and iodanes. In this context, catalytic dearomative two-component C–C bond formations of nitroarenes with ylides were reported by Piettre and Trost, independently (Fig. 1A).^11*a*,*b*^ These reactions enabled the dearomatization of one equivalent of nitroarenes. 1,2-Difunctionalized products were commonly obtained, and only one example of 1,4-difunctionalization was shown. Alternatively, we recently developed a Pd-catalyzed three-component dearomatization of bromoarenes by using TMS–diazomethane and allyl–BF_3_K, realizing two simultaneous C–C bond formations at 1,4-positions on the benzenoid (Fig. 1B).^11*c*^ The key of this reaction was the generation of a Pd–benzyl complex intermediate through a Pd–carbene migratory insertion.^12^ However, applicable carbon units were limited to TMS–diazomethane and an allyl nucleophile, which hampered further synthetic applications of the method. In order to perform a dearomative synthesis of multi-functionalized carbocyclic molecules, the development of new methods to introduce other functional groups is needed. To this end, we envisaged that an ester functionality in the form of a malonate would be a versatile handle for further derivatization. However, it is known that malonates predominantly react with Pd–benzyl complexes to give benzyl substitution products.^13,14^ Although one report by the group of Bao showed a dearomative alkylation of benzyl chlorides with malonates, only naphthylmethyl chlorides were suitable substrates.^15^ Herein, we report the development of a Pd-catalyzed three-component C–C bond forming dearomatization of haloarenes using malonate nucleophiles, giving 1,4- and 1,2-difunctionalized alicyclic molecules. This method can use aryl *N*-tosylhydrazones^16^ as an alternative to TMS–diazomethane. Furthermore, it was found that the present methodology was amenable to arenes with various electronic nature, including azines and heteroles.

## Results and discussion

First, we explored optimized conditions for the dearomative alkylation using 1-bromonaphthalene (**1A**), TMS–diazomethane (**2**), and diethylmalonate **3a** as model substrates (Table 1). Through extensive investigations, we identified the best conditions with Pd(OAc)_2_ (5.0 mol%) and **L1** (20 mol%) as the catalyst, along with NaH, and 3 Å molecular sieves (MS) in toluene at 60 °C, obtaining desired dearomatized product **4Aa** in 88% yield (entry 1, see the ESI† for details). Of all the triarylphosphines tested in this screening, decreasing the electron-donating ability of the phosphine led to a lower yield of **4Aa** (entries 2–4). Regarding to diphosphines, DPEphos had a comparable effect to **L1**,producing **4Aa** in a high yield (entry 5). The reaction in the absence of ligand generated no product (entry 6). The choice of base was critical: when less basic LiO^*t*^Bu and Cs_2_CO_3_ were used instead of NaH, the yield of **4Aa** significantly dropped (entries 7 and 8). The reaction in the absence of 3 Å MS decreased the yield of **4Aa** (entry 9). It was found that preformed sodium enolate **3a′** was also an applicable alkylating agent under the reaction conditions without NaH (entry 10). Additionally, this reaction favored non-polar solvents, as cyclohexane also afforded **4Aa** in 54% yield (entry 11). Through these studies, no benzylic substitution was observed.

**Table 1:** Conditions optimization^a^

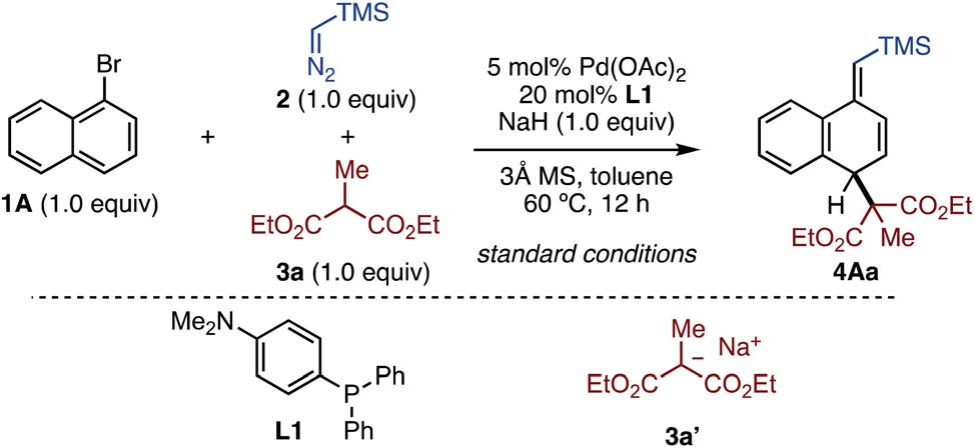		
Entry	Variation from standard conditions	**4Aa** b/%
1	None	88
2	P(*p*-anisyl)_3_ instead of **L1**	81
3	PPh_3_ instead of **L1**	58
4	P(C_6_F_5_)_3_ instead of **L1**	0
5	DPEphos (10 mol%) instead of **L1**	92
6	Without **L1**	0
7	LiO^*t*^Bu instead of NaH	2
8	Cs_2_CO_3_ instead of NaH	0
9	Without 3 Å MS	71
10	**3a′** was used instead of **3a**^c^	62
11	Cyclohexane instead of toluene	54

a Conditions: **1A** (0.20 mmol), **2** (0.20 mmol), **3a** (0.20 mmol), Pd(OAc)_2_ (5.0 mol%), **L1** (20 mol%), NaH (1.0 equiv.), 3 Å MS (50 mg), toluene (1.0 mL), 60 °C, 12 h.

b NMR yield.

c Without NaH and 3 Å MS at 70 °C.

**Scheme 1:**
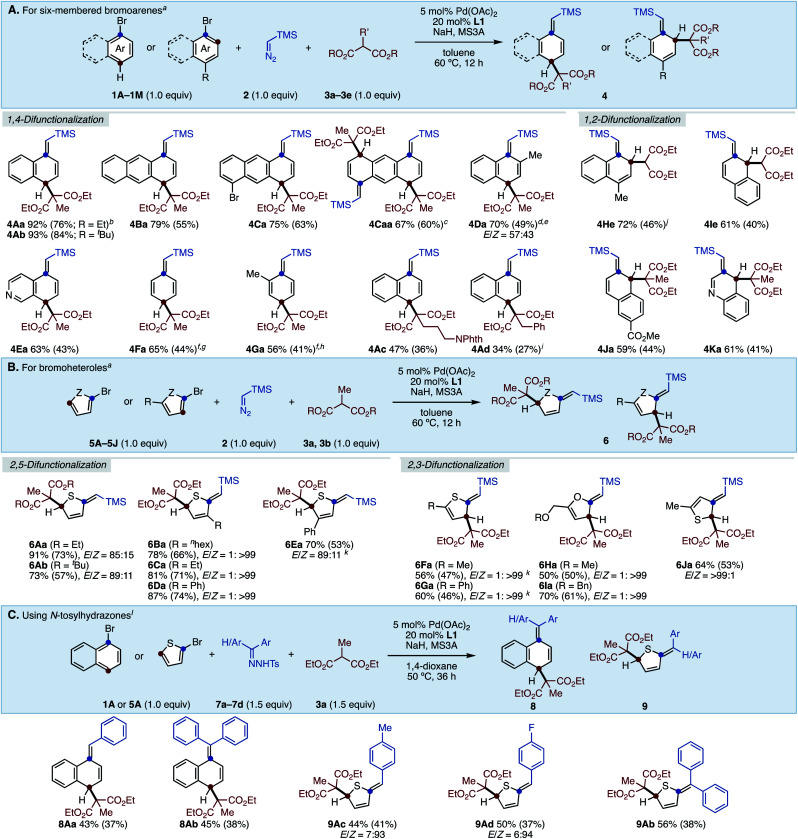
Substrate scope. (A) Using six-membered bromoarenes. (B) Using bromoheteroles. (C) Using *N*-tosylhydrazones. ^*a*^Conditions. **1** or **5** (0.20 mmol), **2** (0.20 mmol), **3** (0.20 mmol), Pd(OAc)_2_ (5.0 mol%), **L1** (20 mol%), NaH (1.0 equiv.), 3 Å MS (50 mg), toluene (1.0 mL), 60 °C, 12 h. NMR yields were shown and numbers in parenthesis are isolated yield. ^*b*^DPEphos was used instead of **L1**. ^*c*^2.0 equiv. of **2**, **3**, and NaH. Cyclohexane as solvent. ^*d*^1.5 equiv. of **3** and NaH. ^*e*^90 °C. ^*f*^Pd(cod)Cl_2_ (5.0 mol%), DPEphos (10 mol%) as catalyst and KBr (2.0 equiv.) were used. ^*g*^40 °C. ^*h*^50 °C. ^*i*^70 °C. ^*j*^DPEphos at 80 °C. ^*k*^Cyclohexane as solvent. ^*l*^Conditions. **1A** or **5A** (0.20 mmol), **7** (0.30 mmol), **3a** (0.30 mmol), Pd(OAc)_2_ (5.0 mol%), **L1** (20 mol%), NaH (3.0 equiv.), 3 Å MS (50 mg), 1,4-dioxane (1.0 mL), 50 °C, 36 h.

With the optimized conditions in hand, we next investigated the substrate generality of this reaction (Scheme 1A). As π-extended aromatics, anthracene also showed good reactivity to deliver dihydroanthracene **4Ba** in high yield. The reaction of dibromoanthracene **1C** smoothly proceeded to give dearomatized product **4Ca** in good yield by using one equivalent of malonate **3a**. For the same substrate **1C**, increasing the amounts of **2**, **3a**, and NaH to two equivalents each furnished tetrahydroanthracene **4Caa** in 67% NMR yield. Bromoarene with steric hindrance around the C–Br bond was also applicable to this reaction, generating C2-methyl-substituted product **4Da** in good yield albeit as a diastereoisomeric mixture (57 : 43). 5-Bromoisoquinoline (**1E**) was successfully dearomatized under the present conditions, giving **4Ea** in moderate yield. It is noteworthy that less reactive and simple phenyl rings were successfully converted to the corresponding cyclohexadiene skeletons **4Fa** and **4Ga** (see the ESI† for details). Although rearomatization occurred during the reactions probably due to the low stability of the products, **4Fa** and **4Ga** can be isolated in 44% and 41% yields, respectively. Regarding the scope of malonates, it was found that the nature of the ester groups did not affect the reaction efficiency, yielding **4Ab** in almost the same yield as **4Aa**. Moreover, we subjected diethylmalonates bearing phthalimidylalkyl (**3c**) as well as benzyl (**3d**) to this protocol, generating the corresponding products **4Ac** and **4Ad** in moderate yields. Probably due to the steric hinderance around the active methine moiety, higher temperatures were required for the reaction of **3d**. Under the present conditions, only malonates can be used and other active methylene compounds were ineffective (see the ESI† for details). On the other hand, in the case of “*para*-substituted” bromoarenes, the reaction occurred in a 1,2-difunctionalization fashion. 4-Methylbromonaphthalene (**1H**) as well as 2-bromonaphthalenes (**1I** and **1J**) gave 1,2-difunctionalized compounds. Pleasingly, the dearomative three-component C–C bond formation of 4-bromoquinoline (**1K**) was successful, furnishing a 3,4-dihydroquinoline core in a good yield.

Bromoheteroles were also reactive under the present reaction system (Scheme 1B). 2-Bromothiophene (**5A**) dearomatively assembled with **2** and malonates **3** to afford the corresponding dihydrothiophenes **6Aa** and **6Ab** in good yields with moderate *E*-selectivity. C3-Substituted 2-bromothiophenes also smoothly underwent the dearomative reaction, giving 2,5-difunctionalized heterocycles (**6Ba–6Da**) with remarkable *Z*-selectivity due to the steric repulsion. 4-Phenyl-2-bromothiophene (**5E**) was converted to **6Ea** in good yield. In contrast, bromoheteroles bearing substituents at the C5-position led to 2,3-difunctionalization, yielding the corresponding dearomatized products **6Fa–6Ia** in moderate yield with exclusive *Z*-selectivity. 3-Bromothiophene was dearomatized in a 2,3-difunctionalization manner (**6Ja**).

Furthermore, although diazo esters were not applicable under these conditions (see the ESI† for details), we found that aryl *N*-tosylhydrazones^16^ can be used in this reaction instead of TMS–diazomethane (Scheme 1C). For instance, aryl *N*-tosylhydrazones successfully reacted with 1-bromonaphthalene (**1A**) as well as 2-bromothiophene (**5A**) to give the corresponding products in moderate yields. Dearomatized products with tetra-substituted olefins were also synthesized by using diphenyl *N-*tosylhydrazone **7b** in acceptable yields.

**Scheme 2:**
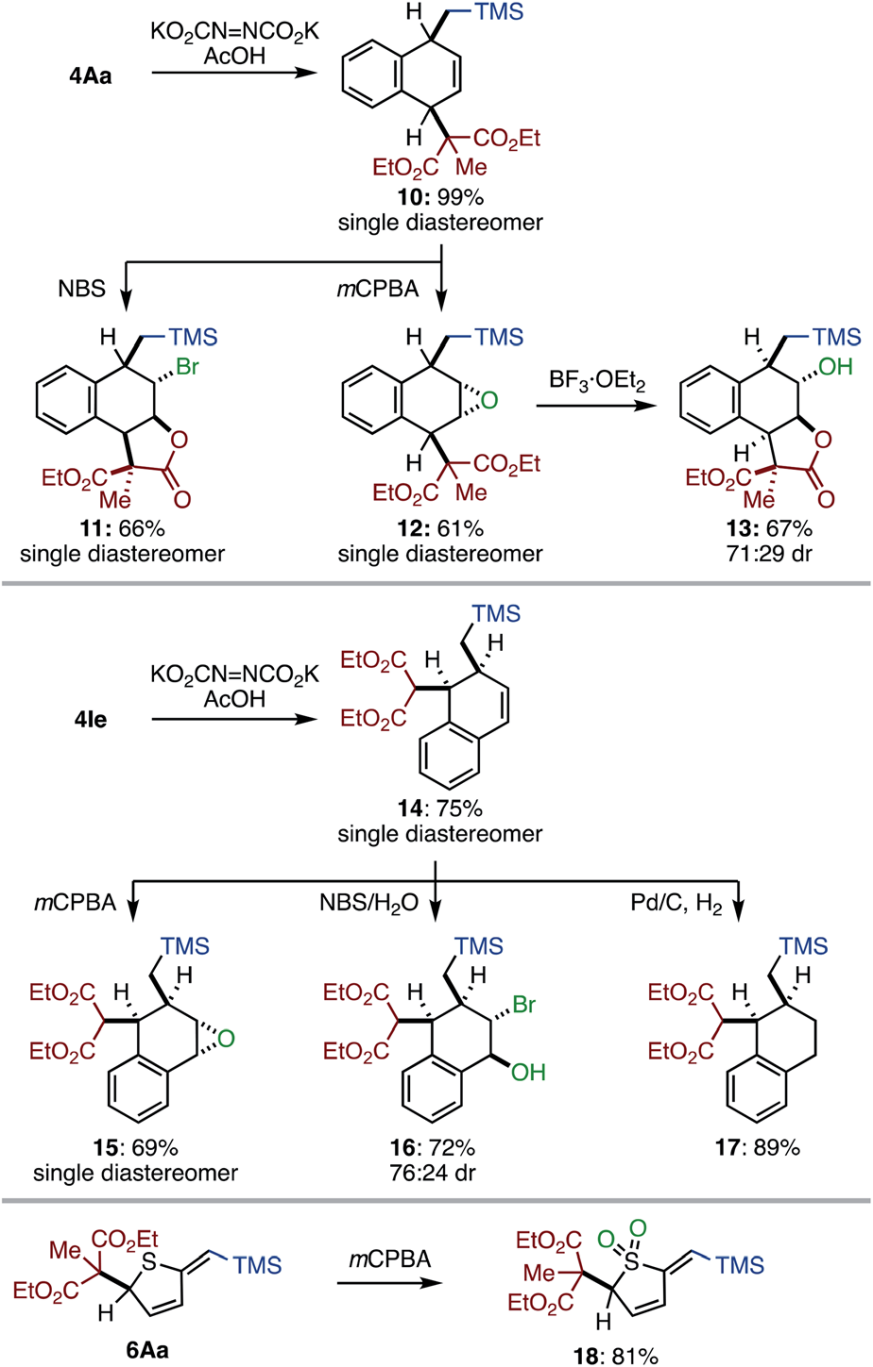
Derivatization of the products.

The most significant value of the method is that the dearomatized product can be elaborated to various multi-substituted alicyclic systems (Scheme 2). Partial reduction of **4Aa** under diimide conditions produced dihydronaphthalene **10** in high yield with remarkable diastereoselectivity. The remaining olefin of **10** can be further functionalized. For instance, the treatment of **10** with NBS furnished bromolactone **11** in good yield with high diastereoselectivity. *m*CPBA oxidation of **10** produced epoxide **12** as a single diastereoisomer; epoxide **12** was further converted by using BF_3_·OEt_2_ to construct *trans* C–O bonds on the cyclic skeleton (**13**). A similar structural diversification was operative for 1,2-difunctionalized product **4Ie**. Diastereo- and site-selective reduction of **4Ie**, followed by epoxidation, bromohydrin formation, or hydrogenation furnished the corresponding multiply-functionalized alicyclic systems (**15–17**). Heterocycle **6Aa** was successfully converted to sulfone **18** in good yield. These results strongly support that the present dearomative method can lead to a diverse range of highly functionalized alicyclic systems.

**Scheme 3:**
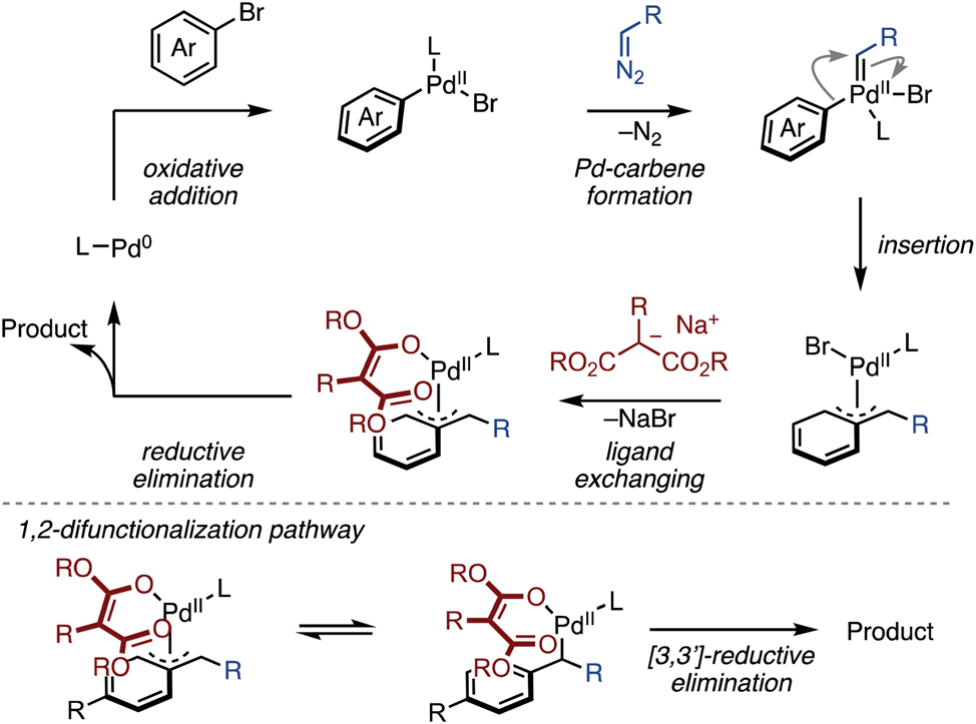
Proposed mechanism.

Our proposed mechanism is outlined in Scheme 3. The first oxidative addition of a bromoarene, followed by a Pd–carbene formation with a diazo compound generates an Ar–Pd–carbene complex. The Pd–carbene species leads to migratory insertion^12^ of the aryl moiety, giving a Pd–benzyl complex. Subsequently, the Pd–benzyl complex reacts with a sodium malonate to produce a benzyl–Pd–*O*-enolate species. The final C–C bond formation from the Pd–*O*-enolate species releases a dearomatized product. In this reaction mechanism, the key for the final C–C bond formation would be the generation and reactivity of the benzyl–Pd–*O*-enolate intermediate.^17^ The highly coordinating ability of malonates probably favors formation of the benzyl–Pd–*O*-enolates species, which then allows the inner-sphere C–C bond formation to give 1,4-difunctionalized products. Although a detailed mechanism and the site-selectivity^17,18^ is unclear at this stage, in the case of C4-substituted arenes, the C–C bond formation at the C2 position takes place from a σ-benzyl–Pd–*O*-enolate. This likely occurs a 3,3′-sigmatropic reductive elimination mechanism, probably due to steric repulsion between the malonate moiety and the C4-substituent.^19^

## Conclusions

In summary, we have developed the dearomative three-component C–C bond formation of bromoarenes with diazo compounds and malonates by a palladium catalyst. The reaction was applicable to a variety of aryl bromides including azines and heteroles, which suggests that this reaction system is not restricted to the electronic nature of the arenes. The structural elaboration of the dearomatized products showcase the synthetic utility of the present method. Further studies to develop asymmetric dearomatizations using a chiral catalyst^20^ and to elucidate the reaction mechanism are underway in our laboratory.^21^

## Conflicts of interest

There are no conflicts to declare.

## Supplementary Material

SC-011-D0SC02881A-s001
