# Supplementary material for: Catalytic three-component C–C bond forming dearomatization of bromoarenes with malonates and diazo compounds
Source: Chem Sci. 2020 Jul 29;11(33):8779–84. doi: 10.1039/d0sc02881a (PMC8163412; doi:10.1039/d0sc02881a)

Electronic Supplementary Material (ESI) for Chemical Science.
This journal is © The Royal Society of Chemistry 2020

Supplementary Information

Catalytic Three-component C–C Bond Forming Dearomatization of Bromoarenes with Malonates and Diazo Compounds

Hiroki Kato,[a] Itsuki Musha,[a] Masaaki Komatsuda,[a] Kei Muto,*[b] Junichiro Yamaguchi*[a]

[a] *Department of Applied Chemistry, Waseda University, 3-4-1 Ohkubo, Shinjuku, Tokyo 169-8555, Japan*

[b] *Waseda Institute for Advanced Study, Waseda University, Shinjuku, Tokyo 169-8050, Japan.*

E-mail: keimuto@aoni.waseda.jp (KM), junyamaguchi@waseda.jp (JY)

Table of Contents

1. General S2–S3
2. Synthesis of diethyl 2-(3-(1,3-dioxoisoindolin-2-yl)propyl)malonate (**3c**) S4
3. Pd-Catalyzed three-component C–C bond forming dearomatization of **1** S5–S12
4. Pd-Catalyzed three-component C–C bond forming dearomatization of **5** S12–S17
5. Pd-Catalyzed three-component C–C bond forming dearomatization using *N*-tosylhydrazones S17–S20
6. Derivatization of products S21–S26
7. Attempts toward asymmetric dearomatization S27–S28
8. Effect of parameters S29–S35
9. References S36
10. ^{1}H and ^{13}C NMR Spectra S37–S119
11. Crude ^{1}H NMR spectra of **4**, **6**, **8** and **9** S120–S150

1. General

Unless otherwise noted, all reactants or reagents including dry solvents were obtained from commercial suppliers and used as received. $Pd(OAc)_2$ and bromobenzene (**1F**) were obtained from FUJIFILM Wako Pure Chemical Corporation. (Trimethylsilyl)diazomethane solution 2.0 M in diethyl ether, 4-(dimethylamino)phenyldiphenylphosphine (**L1**), molecular sieves, 3 Å (3Å MS) and 2-bromonaphthalene (**1I**) were obtained from Sigma-Aldrich. 1-Bromonaphthalene (**1A**), 1-bromoanthracene (**1B**), 1,5-dibromoanthracene (**1C**), 1-bromo-2-methylnaphthalene (**1D**), 5-bromoisoquinoline (**1E**), 2-bromotoluene (**1G**), methyl 6-bromo-2-naphthoate (**1J**), 3-bromoquinoline (**1K**), diethyl methylmalonate (**3a**), diethyl benzylmalonate (**3d**), diethyl malonate (**3e**), 2-bromo-3-hexylthiophene (**5B**), 2-bromo-5-phenylthiophene (**5G**) and 4-bromo-2-methylthiophene (**5J**) were obtained from Tokyo Chemical Industry (TCI). 2-Bromothiophene (**5A**) was obtained from Acros Organics. 1-Bromo-4-methylnaphthalene (**1H**),[1] 2-bromo-3-ethylthiophene (**5C**),[2] 2-bromo-3-phenylthiophene (**5D**),[3] 2-bromo-4-phenylthiophene (**5E**),[4,5] 2-bromo-5-methylthiophene (**5F**),[6] 2-bromo-5-(methoxymethyl)furan (**5H**),[7] 2-((benzyloxy)methyl)-5-bromofuran (**5I**),[7] di-*tert*-butyl 2-methylmalonate (**3b**),[8] diethyl 2-(3-(1,3-dioxoisoindolin-2-yl)propyl)malonate (**3c**),[9] *N*'-benzylidene-4-methylbenzenesulfonohydrazide (**7a**),[10] *N*'-(diphenyl methylene)-4-methylbenzenesulfonohydrazide (**7b**),[11] 4-methyl-*N*'-(4-methylbenzylidene) benzenesulfonohydrazide (**7c**),[10] and *N*'-(4-fluorobenzylidene)-4-methylbenzenesulfonohydrazide (**7d**)[10] were synthesized according to procedures and the spectra matched with those of compounds reported in the literature. Unless otherwise noted, all reactions were performed with dry solvents under an atmosphere of N_2 in dried glassware using standard vacuum-line techniques. All three-component C–C bond forming dearomatizations were performed in an 8-mL glass vessel tube equipped with a screw cap and heated (IKA Plate RCT digital) in a 16-well aluminum reaction block (IKA DB4.3 Block) unless otherwise noted. All work-up and purification procedures were carried out with reagent-grade solvents in air.

Analytical thin-layer chromatography (TLC) was performed using Silica-gel 70 TLC Plate-Wako (0.25 mm). The developed chromatogram was analyzed by UV lamp (254 nm). Flash column chromatography was performed with Biotage Isolera® equipped with Biotage SNAP Cartridge KP-Sil columns and hexane/EtOAc as an eluent unless otherwise noted. Preparative thin-layer chromatography (PTLC) was performed using Wakogel B5-F silica coated plates (0.75 mm) prepared in our laboratory. Basic alumina chromatography was performed using basic alumina, activated (pH = 9.0–11.0) from Wako Pure Chemical Corporation. Preparative recycling gel permeation chromatography (GPC) was performed with a JAI LaboACE LC-5060 instrument equipped with JAIGEL-2HR columns using $CHCl_3$ as an eluent. High-resolution mass spectra were conducted on Thermo Fisher Scientific ExactivePlus (ESI). Chiral high performance liquid chromatography (HPLC)

was performed using SHIMADZU Prominence-i LC-2030C Plus® equipped with DAICEL Chiralcel®. Details of chromatographic conditions on the separation of the products are described with compound data in Section 7. Nuclear magnetic resonance (NMR) spectra were recorded on a JEOL JNM-ECS-400 (^{1}H 400 MHz, ^{13}C 101 MHz) spectrometer. Chemical shifts for ^{1}H NMR are expressed in parts per million (ppm) relative to tetramethylsilane (δ 0.00 ppm). Chemical shifts for ^{13}C NMR are expressed in ppm relative to $CDCl_3$ (δ 77.0 ppm). Data are reported as follows: chemical shift, multiplicity (s = singlet, d = doublet, dd = doublet of doublets, ddd = doublet of doublets of doublets, t = triplet, dt = doublet of triplets, td = triplet of doublets, q = quartet, qd = quartet of doublets, m = multiplet), coupling constant (Hz), and integration.

2. Synthesis of diethyl 2-(3-(1,3-dioxoisoindolin-2-yl)propyl)malonate (3c)[9]

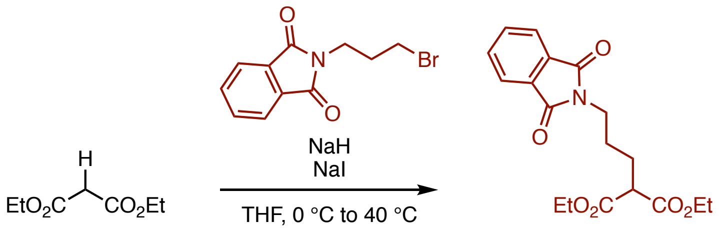

3e **3c**

To a solution of sodium hydride (60%, dispersion in paraffin liquid: 143.8 mg, 3.6 mmol, 1.2 equiv) in THF (7.8 mL) was added diethyl malonate (**3e**: 460 μL, 3.0 mmol, 1.0 equiv) at 0 °C. After stirring at room temperature for 20 min, to the mixture were added the solution of *N*-(3-bromopropyl)phthalimide (816.5 mg, 3.0 mmol, 1.0 equiv) in THF (5.2 mL) slowly and sodium iodide (45.0 mg, 300 μmol, 0.10 equiv). After stirring the mixture at room temperature for 10 h with monitoring the reaction progress by TLC, the mixture was stirred at 40 °C overnight. The mixture was diluted with Et_2O and quenched with 1M HCl aq. and brine. The solvent was extracted three times with Et_2O. The combined organic layer was dried over $MgSO_4$, filtrated, and concentrated *in vacuo*. Purification by Isolera® (hexane:EtOAc = 99:1 to 3:1) afforded diethyl 2-(3-(1,3-dioxoisoindolin-2-yl)propyl)malonate (**3c**: 543 mg, 1.56 mmol, 52% yield) as a yellow oil. The spectra are in accordance with those of the compounds reported in the literature.

3. Pd-Catalyzed three-component C–C bond forming dearomatization of 1

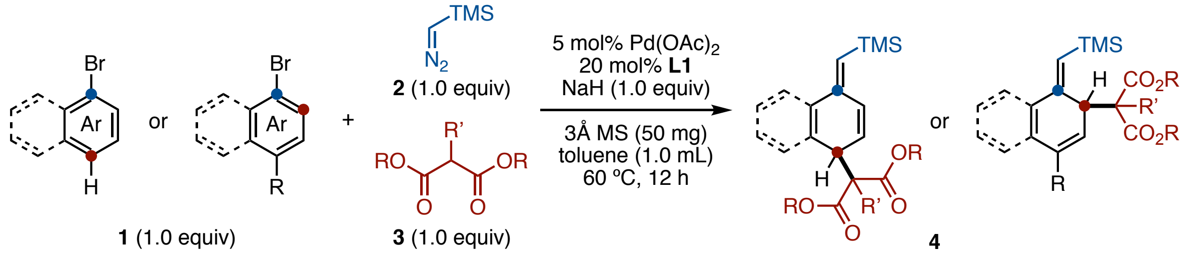

General Procedure A

To an 8-mL glass vessel equipped with a screw cap containing a magnetic stirring bar and 3Å MS (50.0 mg) was dried with a heat-gun *in vacuo* and filled with N_2 after cooling to room temperature. To this vessel were added bromoarene **1** (0.20 mmol, 1.0 equiv), $Pd(OAc)_2$ (2.3 mg, 0.010 mmol, 5.0 mol%), 4-(dimethylamino)phenyldiphenylphosphine (**L1**: 12.2 mg, 0.040 mmol, 20 mol%), sodium hydride (60%, dispersion in paraffin liquid: 8.0 mg, 0.20 mmol, 1.0 equiv) and malonate **3** (0.20 mmol, 1.0 equiv). The vessel was placed under vacuum and refilled N_2 gas three times, and then added a solution of (trimethylsilyl)diazomethane (**2**, 2.0 M in Et_2O: 100 μL, 0.20 mmol, 1.0 equiv) and toluene (1.0 mL). The vessel was sealed with a screw cap and then heated at 60 °C for 12 h with stirring. After cooling the reaction mixture to room temperature, the mixture was passed through a short alumina pad with hexane/EtOAc (1:1) as an eluent. The filtrate was concentrated *in vacuo*. The yield of **4** was determined by ^1H NMR analysis using CH_2Br_2 as an internal standard. The residue was purified by Isolera® with a basic alumina column cartridge (hexane) to afford the corresponding dearomatized product **4**.

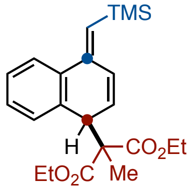

Diethyl (*E*)-2-methyl-2-(4-((trimethylsilyl)methylene)-1,4-dihydronaphthalen-1-yl)malonate (4Aa)

Following the General Procedure A, the reaction was conducted by using DPEphos (10.7 mg, 0.020 mmol, 10 mol%) as the ligand. ^1H NMR yield of **4Aa** was 92% (^1H NMR peak at 4.59 ppm (d, J = 5.6 Hz, 1H) was used). Purification by Isolera® with a basic alumina column cartridge (hexane/EtOAc = 99:1 to 9:1) afforded **4Aa** (58.5 mg, 152 μmol, 76% yield) as a colorless oil. A part of product decomposed during purification (rearomatization). ^1H NMR (400 MHz, $CDCl_3$) δ 7.72 (d, J = 8.0 Hz, 1H), 7.32–7.25 (m, 1H), 7.20–7.13 (m, 2H), 6.83 (d, J = 10.0 Hz, 1H), 6.30 (s, 1H), 6.27 (dd, J = 10.0, 5.6 Hz, 1H), 4.59 (d, J = 5.6 Hz, 1H), 4.29–4.10 (m, 4H), 1.30–1.21 (m, 6H), 1.05 (s, 3H), 0.23 (s, 9H); ^{13}C NMR (101 MHz, $CDCl_3$) δ 170.8, 145.4, 136.9, 133.6, 132.0, 128.4, 128.3, 127.00, 126.96,

125.1, 123.8, 61.4, 44.9, 14.5, 14.0, 13.9, 0.2 (three peaks are missing due to overlapping); HRMS (ESI) *m/z* calcd for $C_{22}H_{31}O_4Si$ $[M + H]^+$: 387.1986 found 387.1980.

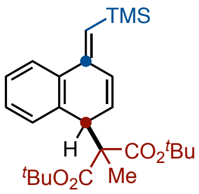

Di-*tert*-butyl (*E*)-2-methyl-2-(4-((trimethylsilyl)methylene)-1,4-dihydronaphthalen-1-yl)malonate (4Ab)

^{1}H NMR yield of **4Ab** was 93% (^{1}H NMR peak at 4.56 ppm (d, J = 5.2 Hz, 1H) was used). Purification by Isolera® with a basic alumina column cartridge (hexane/EtOAc = 99:1 to 9:1) afforded the mixture of **4Ab** and **L1** (80.2 mg, **4Ab**:**L1** = 90:10) and the yield of **4Ab** was determined as 84%. Further purification by Isolera® with a basic alumina column cartridge (hexane/EtOAc = 99:1 to 9:1, two times) was performed to give partially pure **4Ab** as a yellow oil for the characterization. A part of product decomposed during purification (rearomatization). ^{1}H NMR (400 MHz, $CDCl_3$) δ 7.71 (d, J = 8.0 Hz, 1H), 7.37 (d, J = 8.0 Hz, 1H), 7.27–7.23 (m, 1H), 7.18–7.14 (m, 1H), 6.81 (d, J = 10.8 Hz, 1H), 6.29–6.25 (m, 2H), 4.56 (d, J = 5.2 Hz, 1H), 1.50 (s, 9H), 1.46 (s, 9H), 0.94 (s, 3H), 0.23 (s, 9H); ^{13}C NMR (101 MHz, $CDCl_3$) δ 170.0, 169.9, 145.5, 136.7, 134.1, 131.5, 129.1, 129.0, 126.8, 124.5, 123.7, 81.7, 81.4, 62.7, 44.4, 27.9, 27.8, 14.7, 0.2 (one peak is missing due to overlapping); HRMS (ESI) *m/z* calcd for $C_{26}H_{38}O_4NaSi$ $[M + Na]^+$: 465.2432 found 465.2427.

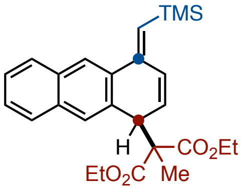

Diethyl (*E*)-2-methyl-2-(4-((trimethylsilyl)methylene)-1,4-dihydroanthracen-1-yl)malonate (4Ba)

^{1}H NMR yield of **4Ba** was 79% (^{1}H NMR peak at 4.77 ppm (d, J = 5.6 Hz, 1H) was used). Purification by Isolera® with a basic alumina column cartridge (hexane/EtOAc = 99:1 to 9:1) afforded **4Ba** (47.6 mg, 109 μmol, 55% yield) as a colorless oil. A part of product decomposed during purification (rearomatization). ^{1}H NMR (400 MHz, $CDCl_3$) δ 8.17 (s, 1H), 7.86–7.82 (m, 1H), 7.71–7.68 (m, 1H), 7.66 (s, 1H), 7.45–7.38 (m, 2H), 6.90 (d, J = 10.0 Hz, 1H), 6.50 (s, 1H), 6.33 (ddd, J = 10.0, 5.6, 1.6 Hz, 1H), 4.77 (d, J = 5.6 Hz, 1H), 4.31–4.14 (m, 4H), 1.29 (t, J = 7.2 Hz, 3H), 1.18 (t, J = 7.2 Hz, 3H), 1.09 (s, 3H), 0.27 (s, 9H); ^{13}C NMR (101 MHz, $CDCl_3$) δ 170.8, 170.7, 145.8, 135.1, 132.3, 132.2, 131.9, 128.3, 128.0, 127.3, 127.2, 126.0, 125.9, 125.4, 122.8, 62.0, 61.5, 44.8, 14.7, 14.0, 13.9, 0.2 (two peaks are missing due to overlapping); HRMS (ESI) *m/z* calcd for $C_{26}H_{33}O_4Si$ $[M + H]^+$: 437.2143 found 437.2139.

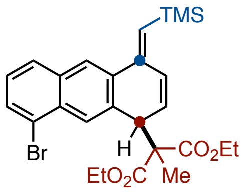

Diethyl (*E*)-2-(8-bromo-4-((trimethylsilyl)methylene)-1,4-dihydroanthracen-1-yl)-2-methylmalonate (4Ca)

^{1}H NMR yield of **4Ca** was 75% (^{1}H NMR peak at 4.83 ppm (d, J = 5.6 Hz, 1H) was used). Purification by Isolera® with a basic alumina column cartridge (hexane/EtOAc = 99:1 to 9:1) afforded **4Ca** (65.0 mg, 126 µmol, 63% yield) as a colorless oil. A part of product decomposed during purification (rearomatization). ^{1}H NMR (400 MHz, $CDCl_3$) δ 8.15 (s, 1H), 8.01 (s, 1H), 7.80 (d, J = 8.0 Hz, 1H), 7.70 (d, J = 8.0 Hz, 1H), 7.27 (t, J = 8.0 Hz, 1H), 6.90 (d, J = 10.0 Hz, 1H), 6.52 (s, 1H), 6.39 (dd, J = 10.0, 5.6 Hz, 1H), 4.83 (d, J = 5.6 Hz, 1H), 4.37–4.18 (m, 4H), 1.30 (t, J = 7.2 Hz, 3H), 1.24 (t, J = 7.2 Hz, 3H), 1.09 (s, 3H), 0.27 (s, 9H); ^{13}C NMR (101 MHz, $CDCl_3$) δ 170.8, 170.4, 145.2, 136.0, 133.6, 133.5, 132.0, 130.7, 129.9, 128.5, 128.1, 126.5, 126.4, 126.2, 123.3, 122.2, 62.0, 61.9, 61.5, 44.9, 14.6, 14.0, 13.9, 0.2; HRMS (ESI) *m/z* calcd for $C_{26}H_{32}BrO_4Si$ [M + H]$^+$: 515.1248 found 515.1245.

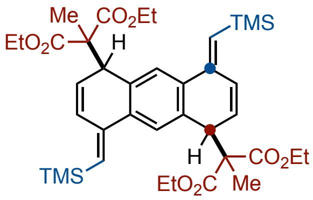

Tetraethyl 2,2'-((4*E*,8*E*)-4,8-bis((trimethylsilyl)methylene)-1,4,5,8-tetrahydroanthracene-1,5-diyl)bis(2-methylmalonate) (4Caa)

Following the general procedure, the reaction was conducted by using **2** (2.0 equiv), **3a** (2.0 equiv), and NaH (2.0 equiv) in cyclohexane. ^{1}H NMR yield of **4Caa** was 67% (^{1}H NMR peak at 4.63 ppm (d, J = 4.8 Hz, 2H) was used). Purification by Isolera® with a basic alumina column cartridge (hexane/EtOAc = 99:1 to 9:1) afforded the mixture of **4Caa** and **4Ca** (102.1 mg, **4Caa**:**4Ca** = 77:23) and the yield of **4Caa** was determined as 60%. Further purification by Isolera® with a basic alumina column cartridge (hexane/EtOAc = 99:1 to 9:1) was performed to give partially pure **4Caa** as a yellow oil for the characterization. A part of product decomposed during purification (rearomatization). ^{1}H NMR (400 MHz, $CDCl_3$) δ 7.54 (s, 2H), 6.79 (d, J = 10.0 Hz, 2H), 6.24–6.20 (m, 4H), 4.63 (d, J = 4.8 Hz, 2H), 4.31–4.13 (m, 8H), 1.29 (t, J = 7.2 Hz, 6H), 1.23 (t, J = 7.2 Hz, 6H), 1.04 (s, 6H), 0.21 (s, 18H); ^{13}C NMR (101 MHz, $CDCl_3$) δ 170.7, 145.1, 135.4, 132.4, 131.8, 128.4, 125.2, 124.0, 61.53, 61.47, 45.0, 14.3, 14.0, 13.9, 0.2 (two peaks are missing due to overlapping); HRMS (ESI) *m/z* calcd for $C_{38}H_{55}O_8Si_2$ [M + H]$^+$: 695.3430 found 695.3419.

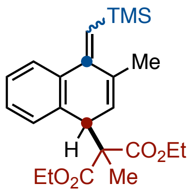

Diethyl (*E*)-2-methyl-2-(3-methyl-4-((trimethylsilyl)methylene)-1,4-dihydronaphthalen-1-yl) malonate (4Da)

Following the General Procedure A, the reaction was conducted by using **3a** (1.5 equiv) and NaH (1.5 equiv) at 90 °C. ^{1}H NMR yield of **4Da** was 70%, *E*/*Z* = 57:43 (^{1}H NMR peak at 6.13 ppm (s, 0.57H), 5.74 ppm (s, 0.43H) was used). Purification by Isolera® with a basic alumina column cartridge (hexane/EtOAc = 99:1 to 9:1) afforded **4Da** as an inseparable diastereoisomeric mixture (39.0 mg, 97.4 µmol, 49% yield, *E*/*Z* = 57:43) as a yellow oil. A part of product decomposed during purification (rearomatization). ^{1}H NMR (400 MHz, $CDCl_3$) δ 7.57 (d, *J* = 7.6 Hz, 0.57H), 7.51–7.49 (m, 0.43H), 7.25–7.19 (m, 2H), 7.13–7.12 (m, 1H), 6.17–6.15 (m, 0.57H), 6.13 (s, 0.57H), 5.97–5.96 (m, 0.43H), 5.74 (s, 0.43H), 4.48–4.45 (m, 1H), 4.29–4.08 (m, 4H), 2.12 (s, 1.71H), 2.03 (s, 1.29H), 1.30–1.19 (m, 6H), 1.07 (s, 1.71H), 1.04 (s, 1.29H), 0.24 (s, 5.13H), 0.14 (s, 3.87H); ^{13}C NMR (101 MHz, $CDCl_3$) δ 171.1, 170.90, 170.87, 170.8, 151.3, 150.9, 141.7, 140.9, 139.3, 138.4, 135.5, 133.7, 128.3, 127.8, 127.6, 127.3, 127.0, 126.8, 126.2, 126.1, 125.9, 124.2, 123.9, 123.1, 62.1, 62.0, 61.4, 61.3, 53.4, 45.3, 45.1, 23.2, 19.7, 14.8, 14.7, 14.03, 14.01, 13.9, 1.0, 0.6 (two peaks are missing due to overlapping); HRMS (ESI) *m/z* calcd for $C_{23}H_{33}O_4Si$ $[M + H]^+$: 401.2143 found 401.2139.

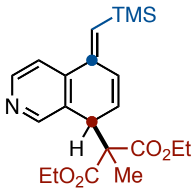

Diethyl (*E*)-2-methyl-2-(5-((trimethylsilyl)methylene)-5,8-dihydroisoquinolin-8-yl)malonate (4Ea)

^{1}H NMR yield of **4Ea** was 63% (^{1}H NMR peak at 6.47 ppm (s, 1H) was used). Purification by Isolera® (hexane/EtOAc = 19:1 to 4:1) afforded **4Ea** (33.5 mg, 86.4 µmol, 43% yield) as a colorless oil. A part of product decomposed during purification (rearomatization). ^{1}H NMR (400 MHz, $CDCl_3$) δ 8.48 (d, *J* = 5.2 Hz, 1H), 8.42 (s, 1H), 7.53 (d, *J* = 5.2 Hz, 1H), 6.82 (d, *J* = 10.4 Hz, 1H), 6.47 (s, 1H), 6.30–6.26 (m, 1H), 4.63 (d, *J* = 5.2 Hz, 1H), 4.28–4.13 (m, 4H), 1.28 (t, *J* = 7.2 Hz, 3H), 1.23 (t, *J* = 7.2 Hz, 3H), 1.07 (s, 3H), 0.25 (s, 9H); ^{13}C NMR (101 MHz, $CDCl_3$) δ 170.5, 170.4, 150.1, 148.1, 143.8, 143.1, 131.2, 129.6, 128.7, 128.4, 117.3, 61.8, 61.7, 61.1, 42.3, 14.9, 14.0, 13.9, 0.0; HRMS (ESI) *m/z* calcd for $C_{21}H_{30}NO_4Si$ $[M + H]^+$: 388.1939 found 388.1935.

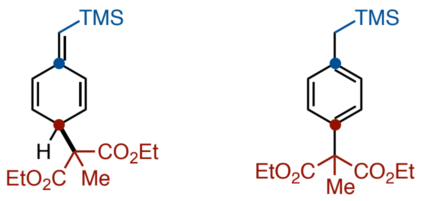

4Fa **4Fa'**

Diethyl 2-methyl-2-(4-((trimethylsilyl)methylene)cyclohexa-2,5-dien-1-yl)malonate (4Fa)

Following the General Procedure A, the reaction was conducted by using Pd(cod)Cl_2 (2.9 mg, 0.010 mmol, 5.0 mol%) and DPEphos (10.7 mg, 0.020 mmol, 10 mol%) as the catalyst and KBr (2.0 equiv) as an additive at 40 °C. ^{1}H NMR yield of **4Fa** and **4Fa'** were 65% and 34%, respectively (^{1}H NMR peaks at 5.37 ppm (s, 1H) for **4Fa** and 2.06 ppm (s, 2H, CH_2) for **4Fa'** were used). Purification by Isolera® with a basic alumina column cartridge (hexane/EtOAc = 99:1 to 9:1) afforded a mixture of **4Fa** and rearomatized compound **4Fa'** (50.2 mg, 149 µmol, 75% yield, **4Fa**:**4Fa'** = 59:41) and the yield of **4Fa** was determined as 44%. Further purification by PTLC (hexane/EtOAc = 9:1) was performed to give **4Fa** as a colorless oil for the characterization. A part of product decomposed during purification (rearomatization). ^{1}H NMR (400 MHz, $CDCl_3$) δ 6.57 (d, J = 10.0 Hz, 1H), 6.28 (d, J = 10.0 Hz, 1H), 5.82–5.79 (m, 1H), 5.73–5.70 (m, 1H), 5.37 (s, 1H), 4.25–4.17 (m, 4H), 3.96–3.95 (m, 1H), 1.28–1.24 (m, 9H), 0.15 (s, 9H); ^{13}C NMR (101 MHz, $CDCl_3$) δ 170.7, 143.7, 134.4, 129.7, 129.5, 128.7, 127.2, 61.5, 57.5, 42.3, 16.1, 14.0, 0.2 (three peaks are missing due to overlapping); HRMS (ESI) m/z calcd for $C_{18}H_{29}O_4Si$ [M + H]$^+$: 337.1830 found 337.1824.

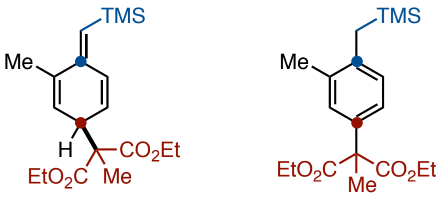

4Ga **4Ga'**

Diethyl (*E*)-2-methyl-2-(3-methyl-4-((trimethylsilyl)methylene)cyclohexa-2,5-dien-1-yl)malonate (4Ga)

Following the General Procedure A, the reaction was conducted by using Pd(cod)Cl_2 (2.9 mg, 0.010 mmol, 5.0 mol%) and DPEphos (10.7 mg, 0.020 mmol, 10 mol%) as the catalyst and KBr (2.0 equiv) as an additive at 50 °C. ^{1}H NMR yield of **4Ga** and **4Ga'** were 56% and 34%, respectively (^{1}H NMR peaks at 5.55 ppm (s, 1H) for **4Ga** and 2.08 ppm (s, 2H, CH_2) for **4Ga'** were used). Purification by PTLC (hexane/EtOAc = 19:1) afforded **4Ga** (29.0 mg, 82.7 µmol, 41% yield) as a colorless oil. A part of product decomposed during purification (rearomatization). ^{1}H NMR (400 MHz, $CDCl_3$) δ 6.60 (dd, J = 10.0, 1.6 Hz, 1H), 5.82–5.78 (m, 1H), 5.67–5.66 (m, 1H), 5.55 (s, 1H), 4.26–4.17 (m, 4H), 3.91–3.90 (m, 1H), 1.87 (s, 3H), 1.28–1.24 (m, 9H), 0.16 (s, 9H); ^{13}C NMR (101 MHz, $CDCl_3$) δ 170.9, 145.2, 136.2, 130.6, 127.9, 125.7, 124.8, 61.4, 57.6, 42.8, 20.0, 16.0 14.0, 0.3 (three peaks are missing due to overlapping); HRMS (ESI) m/z calcd for $C_{19}H_{31}O_4Si$ [M + H]$^+$: 351.1986 found 351.1983.

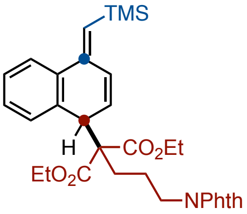

Diethyl (*E*)-2-(3-(1,3-dioxoisoindolin-2-yl)propyl)-2-(4-((trimethylsilyl)methylene)-1,4-dihydronaphthalen-1-yl)malonate (4Ac)

^{1}H NMR yield of **4Ac** was 47% (^{1}H NMR peak at 4.41 ppm (d, *J* = 5.2 Hz, 1H) was used). Purification by Isolera® with a basic alumina column cartridge (hexane/EtOAc = 99:1 to 9:1) afforded **4Ac** (40.1 mg, 71.6 μmol, 36% yield) as a colorless oil. A part of product decomposed during purification (rearomatization). ^{1}H NMR (400 MHz, $CDCl_3$) δ 7.84–7.80 (m, 2H), 7.73–7.69 (m, 2H), 7.67 (d, *J* = 8.0 Hz, 1H), 7.28–7.22 (m, 2H), 7.16 (t, *J* = 8.0 Hz, 1H), 6.79 (d, *J* = 10.0 Hz, 1H), 6.28 (s, 1H), 6.25–6.21 (m, 1H), 4.41 (d, *J* = 5.2 Hz, 1H), 4.18–3.91 (m, 4H), 3.57 (t, *J* = 7.2 Hz, 2H), 1.88–1.77 (m, 2H), 1.66–1.58 (m, 2H), 1.18 (t, *J* = 7.2 Hz, 3H), 1.09 (t, *J* = 7.2 Hz, 3H), 0.22 (s, 9H); ^{13}C NMR (101 MHz, $CDCl_3$) δ 169.9, 169.7, 168.1, 145.0, 136.8, 133.8, 133.7, 132.1, 131.7, 129.1, 127.8, 126.9, 126.8, 124.9, 123.7, 123.1, 64.4, 61.4, 61.2, 45.1, 38.2, 29.5, 24.5, 13.9, 13.7, 0.2; HRMS (ESI) *m/z* calcd for $C_{32}H_{38}NO_6Si$ $[M + H]^+$: 560.2463 found 560.2450.

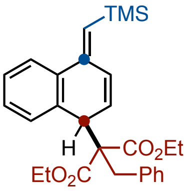

Diethyl (*E*)-2-benzyl-2-(4-((trimethylsilyl)methylene)-1,4-dihydronaphthalen-1-yl)malonate (4Ad)

Following the General Procedure A, the reaction was conducted at 70 °C. ^{1}H NMR yield of **4Ad** was 34% (^{1}H NMR peak at 4.54 ppm (d, *J* = 5.6 Hz, 1H) was used). Purification by Isolera® with a basic alumina column cartridge (hexane/EtOAc = 99:1 to 9:1) afforded **4Ad** (25.0 mg, 54.0 μmol, 27% yield) as a yellow oil. A part of product decomposed during purification (rearomatization). ^{1}H NMR (400 MHz, $CDCl_3$) δ 7.69 (d, *J* = 7.6 Hz, 1H), 7.36 (d, *J* = 7.6 Hz, 1H), 7.30–7.25 (m, 1H), 7.22–7.14 (m, 6H), 6.88 (d, *J* = 10.0 Hz, 1H), 6.46 (ddd, *J* = 10.0, 5.6, 1.2 Hz, 1H), 6.28 (s, 1H), 4.53 (d, *J* = 5.6 Hz, 1H), 4.03–3.63 (m, 4H), 3.33 (d, *J* = 14.0 Hz, 1H), 3.13 (d, *J* = 14.0 Hz, 1H), 0.95 (t, *J* = 7.6 Hz, 3H), 0.88 (t, *J* = 7.6 Hz, 3H), 0.24 (s, 9H); ^{13}C NMR (101 MHz, $CDCl_3$) δ 169.7, 169.4, 145.1, 137.3, 137.0, 134.1, 131.9, 130.3, 129.2, 127.9, 127.8, 126.9, 126.5, 124.9, 123.6, 67.1, 61.1, 60.9, 46.6, 39.0, 13.5, 13.4, 0.2 (one peak is missing due to overlapping); HRMS (ESI) *m/z* calcd for $C_{28}H_{35}O_4Si$ $[M + H]^+$: 463.2299 found 463.2296.

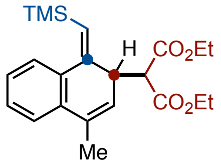

Diethyl (*Z*)-2-(4-methyl-1-((trimethylsilyl)methylene)-1,2-dihydronaphthalen-2-yl)malonate (4He)

Following the General Procedure A, the reaction was conducted by using DPEphos (21.5 mg, 0.040 mmol, 20 mol%) as the ligand. ^{1}H NMR yield of **4He** was 72% (^{1}H NMR peak at 5.88 ppm (s, 1H) was used). Purification by Isolera® with a basic alumina column cartridge (hexane/EtOAc = 99:1 to 9:1) afforded **4He** (35.3 mg, 91.3 μmol, 46% yield) as a yellow oil. A part of product decomposed during purification (rearomatization). ^{1}H NMR (400 MHz, $CDCl_3$) δ 7.44 (dd, J = 7.2, 1.6 Hz, 1H), 7.31–7.16 (m, 3H), 6.09 (d, J = 6.0 Hz, 1H), 5.88 (s, 1H), 4.21–3.90 (m, 5H), 3.28 (d, J = 9.6 Hz, 1H), 2.05 (s, 3H), 1.23 (t, J = 6.8 Hz, 3H), 1.13 (t, J = 6.8 Hz, 3H), 0.21 (s, 9H); ^{13}C NMR (101 MHz, $CDCl_3$) δ 168.1, 167.9, 151.9, 136.7, 133.7, 133.4, 132.6, 128.2, 127.8, 126.0, 125.3, 123.0, 61.3, 61.1, 57.1, 40.3, 19.5, 14.2, 14.0, 0.5; HRMS (ESI) m/z calcd for $C_{22}H_{31}O_4Si$ $[M + H]^+$: 387.1986 found 387.1983.

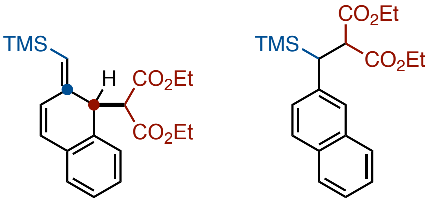

4Ie **4Ie’**

Diethyl (*E*)-2-(2-((trimethylsilyl)methylene)-1,2-dihydronaphthalen-1-yl)malonate (4Ie)

^{1}H NMR yield of **4Ie** was 61% (^{1}H NMR peak at 5.78 ppm (s, 1H) was used). Purification by Isolera® with a basic alumina column cartridge (hexane/EtOAc = 99:1 to 9:1) afforded the mixture of **4Ie** and benzyl substituted compound **4Ie’** (38.6 mg, 104 μmol, 52% yield, **4Ie**:**4Ie’** = 78:22) and the yield of **4Ie** was determined as 40%. A part of product decomposed during purification (rearomatization). **4Ie** was used to derivatization (diimide reduction to give **14**, see the section 6) without further purification. We determined the structure of **4Ie** by the structural analysis of **14**.

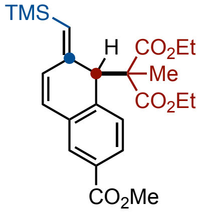

Diethyl (*E*)-2-(6-(methoxycarbonyl)-2-((trimethylsilyl)methylene)-1,2-dihydronaphthalen-1-yl) -2-methylmalonate (4Ja)

^{1}H NMR yield of **4Ja** was 59% (^{1}H NMR peak at 4.66 ppm (s, 1H) was used). Purification by Isolera® with a basic alumina column cartridge (hexane/EtOAc = 99:1 to 3:1) afforded **4Ja** (39.5 mg, 88.8 μmol, 44% yield) as a colorless oil. A part of product decomposed during purification (rearomatization). ^{1}H NMR (400 MHz, $CDCl_3$) δ 7.79–7.74 (m, 2H), 7.50 (d, *J* = 7.6 Hz, 1H), 6.54 (d, *J* = 10.0 Hz, 1H), 6.46 (d, *J* = 10.0 Hz, 1H), 5.79 (s, 1H), 4.66 (s, 1H), 4.22–4.03 (m, 4H), 3.90 (s, 3H), 1.27 (t, *J* = 7.2 Hz, 3H), 1.21 (s, 3H), 1.15 (t, *J* = 7.2 Hz, 3H), 0.16 (s, 9H); ^{13}C NMR (101 MHz, $CDCl_3$) δ 170.6, 169.8, 166.9, 148.6, 140.7, 138.5, 134.6, 129.5, 129.4, 129.1, 128.1, 127.9, 127.2, 61.9, 61.4, 61.2, 52.1, 51.8, 16.8, 14.0, 13.8, 0.1; HRMS (ESI) *m/z* calcd for $C_{24}H_{33}O_6Si$ $[M + H]^+$: 445.2041 found 445.2037.

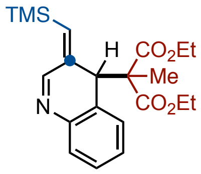

Diethyl (*Z*)-2-methyl-2-(3-((trimethylsilyl)methylene)-3,4-dihydroquinolin-4-yl)malonate (4Ka)

^{1}H NMR yield of **4Ka** was 61% (^{1}H NMR peak at 4.53 ppm (s, 1H) was used). Purification by Isolera® with a basic alumina column cartridge (hexane/EtOAc = 19:1 to 4:1) afforded **4Ka** (31.5 mg, 81.2 μmol, 41% yield) as a colorless oil. A part of product decomposed during purification (rearomatization). ^{1}H NMR (400 MHz, $CDCl_3$) δ 8.21 (s, 1H), 7.40 (dd, *J* = 7.6, 1.2 Hz, 1H), 7.36 (dd, *J* = 7.6, 1.2 Hz, 1H), 7.29 (td, *J* = 7.6, 1.2 Hz, 1H), 7.15 (td, *J* = 7.6, 1.2 Hz, 1H), 6.33 (s, 1H), 4.53 (s, 1H), 4.23–4.05 (m, 4H), 1.28 (t, *J* = 7.2 Hz, 3H), 1.20–1.16 (m, 6H), 0.22 (s, 9H); ^{13}C NMR (101 MHz, $CDCl_3$) δ 170.2, 169.5, 158.8, 147.7, 144.8, 144.0, 129.3, 128.3, 127.9, 127.7, 127.2, 61.7, 61.5, 61.3, 48.2, 15.8, 14.0, 13.8, 0.3; HRMS (ESI) *m/z* calcd for $C_{21}H_{30}NO_4Si$ $[M + H]^+$: 388.1939 found 388.1932.

4. Pd-Catalyzed three-component C–C bond forming dearomatization of 5

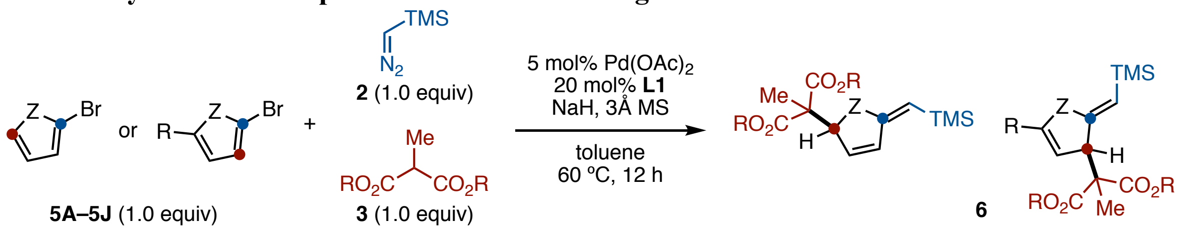

General Procedure B

Following the General Procedure A, bromoheterol **5** was subjected to the catalytic dearomative reaction at 60 °C for 12 h. After cooling the reaction mixture to room temperature, the mixture was passed through a short alumina pad with hexane/EtOAc (1:1) as an eluent. The filtrate was concentrated *in vacuo*. The yield of **6** was determined by ^{1}H NMR analysis using CH_2Br_2 as an internal standard.

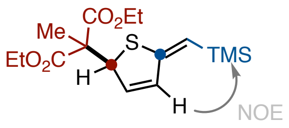

Diethyl (*E*)-2-methyl-2-(5-((trimethylsilyl)methylene)-2,5-dihydrothiophen-2-yl)malonate (6Aa)

^{1}H NMR yield of **6Aa** was 91%, *E*/*Z* = 85:15 (^{1}H NMR peaks at 5.58 ppm (s, 0.85H) and 5.46 ppm (s, 0.15H) were used). Purification by Isolera® with a basic alumina column cartridge (hexane/EtOAc = 99:1 to 9:1) afforded **6Aa** (49.7 mg, 145 μmol, 73% yield, *E*/*Z* = 84:16) as a colorless oil. A part of product decomposed during purification (rearomatization). ^{1}H NMR (400 MHz, $CDCl_3$) δ 6.53–6.51 (m, 0.84H), 6.29–6.26 (m, 0.16H), 6.19–6.16 (m, 0.84H), 6.01–5.99 (m, 0.16H), 5.58 (s, 0.84H), 5.46 (s, 0.16H), 5.24–5.23 (m, 0.16H), 5.17–5.15 (m, 0.84H), 4.28–4.14 (m, 4H), 1.41 (s, 2.52H), 1.40 (s, 0.48H), 1.29–1.24 (m, 6H), 0.15 (s, 1.44H), 0.13 (s, 7.56H); ^{13}C NMR (101 MHz, $CDCl_3$) δ 170.5, 170.33, 170.29, 158.5, 156.8, 138.1, 137.1, 133.6, 133.5, 116.6, 115.9, 61.8, 61.1, 59.0, 58.1, 57.9, 15.7, 15.3, 14.01, 13.99, 0.3, –1.2 (several peaks are missing due to overlapping); HRMS (ESI) *m/z* calcd for $C_{16}H_{27}O_4SSi$ $[M + H]^+$: 343.1394 found 343.1389.

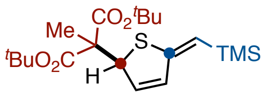

Di-*tert*-butyl (*E*)-2-methyl-2-(5-((trimethylsilyl)methylene)-2,5-dihydrothiophen-2-yl)malonate (6Ab)

^{1}H NMR yield of **6Ab** was 73%, *E*/*Z* = 89:11 (^{1}H NMR peaks at 5.56 ppm (s, 0.89H) and 5.43 ppm (s, 0.11H) were used). Purification by Isolera® with a basic alumina column cartridge (hexane/EtOAc = 99:1 to 9:1) afforded **6Ab** (45.6 mg, 114 μmol, 57% yield, *E*/*Z* = 89:11) as a colorless oil. A part of product decomposed during purification (rearomatization). ^{1}H NMR (400 MHz, $CDCl_3$) δ 6.50–6.48 (m, 0.89H), 6.26–6.24 (m, 0.11H), 6.20–6.18 (m, 0.89H), 6.02–6.00 (m, 0.11H), 5.56 (s, 0.89H), 5.43 (s, 0.11H), 5.13 (d, J = 1.2 Hz, 0.11H), 5.06 (d, J = 1.2 Hz, 0.89H), 1.46 (s, 18H), 1.33 (s, 0.33H), 1.32 (s, 2.67H), 0.15 (s, 0.99H), 0.12 (s, 8.01H); ^{13}C NMR (101 MHz, $CDCl_3$) δ 169.8, 169.5, 157.2, 137.7, 133.1, 116.0, 82.0, 59.3, 59.0, 27.8, 16.0, 0.4 (two peaks are missing due to overlapping); HRMS (ESI) *m/z* calcd for $C_{20}H_{34}O_4SSiNa$ $[M + Na]^+$: 421.1839 found 421.1831.

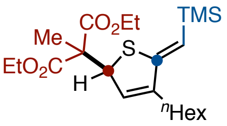

Diethyl (*Z*)-2-(4-hexyl-5-((trimethylsilyl)methylene)-2,5-dihydrothiophen-2-yl)-2-methylmalonate (6Ba)

^{1}H NMR yield of **6Ba** was 78% (^{1}H NMR peak at 5.38 ppm (s, 1H) was used). Purification by Isolera® with a basic alumina column cartridge (hexane/EtOAc = 99:1 to 9:1) afforded **6Ba** (55.9 mg, 131 µmol, 66% yield) as a colorless oil. A part of product decomposed during purification (rearomatization). ^{1}H NMR (400 MHz, $CDCl_3$) δ 5.80 (d, *J* = 1.2 Hz, 1H), 5.38 (s, 1H), 5.00 (d, *J* = 1.2 Hz, 1H), 4.29–4.15 (m, 4H), 2.20 (t, *J* = 7.6 Hz, 2H), 1.54–1.47 (m, 2H), 1.36 (s, 3H), 1.33–1.24 (m, 12H), 0.89 (t, *J* = 7.2 Hz, 3H), 0.17 (s, 9H); ^{13}C NMR (101 MHz, $CDCl_3$) δ 170.7, 170.5, 158.6, 147.3, 129.8, 111.0, 61.7, 58.2, 56.6, 31.6, 29.3, 28.2, 27.8, 22.6, 15.1, 14.04, 14.02, 13.99, –1.1 (one peak is missing due to overlapping); HRMS (ESI) *m/z* calcd for $C_{22}H_{39}O_4SSi$ $[M + H]^+$: 427.2333 found 427.2329.

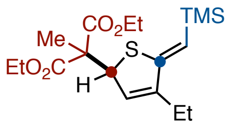

Diethyl (*Z*)-2-(4-ethyl-5-((trimethylsilyl)methylene)-2,5-dihydrothiophen-2-yl)-2-methylmalonate (6Ca)

^{1}H NMR yield of **6Ca** was 81% (^{1}H NMR peak at 5.38 ppm (s, 1H) was used). Purification by Isolera® with a basic alumina column cartridge (hexane/EtOAc = 99:1 to 9:1) afforded **6Ca** (52.9 mg, 143 µmol, 71% yield) as a colorless oil. A part of product decomposed during purification (rearomatization). ^{1}H NMR (400 MHz, $CDCl_3$) δ 5.82–5.81 (m, 1H), 5.38 (s, 1H), 5.00 (d, *J* = 1.2 Hz, 1H), 4.28–4.15 (m, 4H), 2.23 (qd, *J* = 7.2, 1.2 Hz, 2H), 1.36 (s, 3H), 1.28–1.24 (m, 6H), 1.14 (t, *J* = 7.2 Hz, 3H), 0.17 (s, 9H); ^{13}C NMR (101 MHz, $CDCl_3$) δ 170.7, 170.5, 158.5, 148.7, 129.3, 111.0, 61.7, 58.2, 56.6, 21.0, 15.1, 14.00, 13.97, 12.8, –1.1 (one peak is missing due to overlapping); HRMS (ESI) *m/z* calcd for $C_{18}H_{31}O_4SSi$ $[M + H]^+$: 371.1707 found 371.1703.

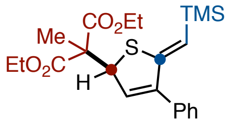

Diethyl (*Z*)-2-methyl-2-(4-phenyl-5-((trimethylsilyl)methylene)-2,5-dihydrothiophen-2-yl)malonate (6Da)

^{1}H NMR yield of **6Da** was 87% (^{1}H NMR peak at 5.40 ppm (s, 1H) was used). Purification by Isolera® with a basic alumina column cartridge (hexane/EtOAc = 99:1 to 9:1) afforded **6Da** (61.8 mg, 148 µmol, 74% yield) as a colorless oil. A part of product decomposed during purification (rearomatization). ^{1}H NMR (400 MHz, $CDCl_3$) δ 7.40–7.34 (m, 3H), 7.29–7.27 (m, 2H), 6.05 (d, *J* = 3.2 Hz, 1H), 5.40 (s, 1H), 5.16 (d, *J* = 3.2 Hz, 1H), 4.31–4.17 (m, 4H), 1.49 (s, 3H), 1.30–1.24 (m, 6H), 0.14 (s, 9H); ^{13}C NMR (101 MHz, $CDCl_3$) δ 170.6, 170.2, 157.4, 148.7, 134.9, 133.0, 128.9, 128.2, 128.0, 115.5, 61.8, 58.5, 56.9, 15.7, 14.03, 14.00, –1.1 (one peak is missing due to overlapping); HRMS (ESI) *m/z* calcd for $C_{22}H_{31}O_4SSi$ $[M + H]^+$: 419.1707 found 419.1701.

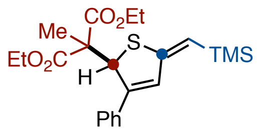

Diethyl (*Z*)-2-methyl-2-(3-phenyl-5-((trimethylsilyl)methylene)-2,5-dihydrothiophen-2-yl)malonate (6Ea)

Following the general procedure, the reaction was conducted by using cyclohexane as the solvent. ^{1}H NMR yield of **6Ea** was 70%, *E*/*Z* = 89:11 (^{1}H NMR peaks at 5.58 ppm (s, 0.85H) and 5.52 ppm (s, 0.15H) were used). Purification by Isolera® with a basic alumina column cartridge (hexane/EtOAc = 99:1 to 9:1) afforded **6Ea** (44.7 mg, 107 µmol, 53% yield, *E*/*Z* = 85:15) as a colorless oil. A part of product decomposed during purification (rearomatization). ^{1}H NMR (400 MHz, $CDCl_3$) δ 7.36–7.28 (m, 5H), 6.53 (s, 0.85H), 6.31 (s, 0.15H), 5.76 (s, 0.15H), 5.68 (s, 0.85H), 5.58 (s, 0.85H), 5.52 (s, 0.15H), 4.25–4.09 (m, 2H), 3.58–3.46 (m, 2H), 1.44 (s, 2.55H), 1.43 (s, 0.45H), 1.26–1.21 (m, 3H), 1.05–1.01 (m, 3H), 0.18 (s, 1.35H), 0.15 (s, 7.65H); ^{13}C NMR (101 MHz, $CDCl_3$) δ 170.9, 168.8, 155.6, 150.5, 135.6, 133.0, 128.5, 128.2, 127.4, 117.0, 61.9, 61.4, 59.3, 58.7, 13.9, 13.7, 13.5, 0.4; HRMS (ESI) *m/z* calcd for $C_{22}H_{31}O_4SSi$ $[M + H]^+$: 419.1707 found 419.1703.

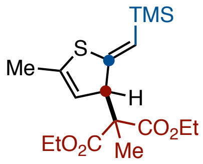

Diethyl (*Z*)-2-methyl-2-(5-methyl-2-((trimethylsilyl)methylene)-2,3-dihydrothiophen-3-yl)malonate (6Fa)

Following the general procedure, the reaction was conducted by using cyclohexane as the solvent. ^{1}H NMR yield of **6Fa** was 56% (^{1}H NMR peak at 5.45 ppm (d, *J* = 1.6 Hz, 1H) was used). Purification by Isolera® with a basic alumina column cartridge (hexane/EtOAc = 99:1 to 9:1) afforded **6Fa** (33.3 mg, 93.4 µmol, 47% yield) as a colorless oil. A part of product decomposed during purification (rearomatization). ^{1}H NMR (400 MHz, $CDCl_3$) δ 5.45 (d, *J* = 1.6 Hz, 1H), 5.32–5.31 (m, 1H), 4.49–4.47 (m, 1H), 4.25–4.11 (m, 4H), 1.99 (s, 3H), 1.40 (s, 3H), 1.29–1.24 (m, 6H), 0.12 (s, 9H); ^{13}C NMR (101 MHz, $CDCl_3$) δ 170.8, 170.0, 156.5, 139.0, 122.1, 118.5, 61.4, 61.3, 60.5, 59.9, 16.7, 14.1, 14.02, 14.00, –1.3; HRMS (ESI) *m/z* calcd for $C_{17}H_{29}O_4SSi$ $[M + H]^+$: 357.1550 found 357.1548.

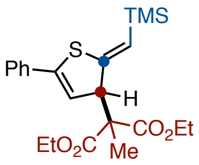

Diethyl (*Z*)-2-methyl-2-(5-phenyl-2-((trimethylsilyl)methylene)-2,3-dihydrothiophen-3-yl) malonate (6Ga)

Following the general procedure, the reaction was conducted by using cyclohexane as the solvent. ^{1}H NMR yield of **6Ga** was 60% (^{1}H NMR peak at 5.35 ppm (d, J = 1.6 Hz, 1H) was used). Purification by Isolera® with a basic alumina column cartridge (hexane/EtOAc = 99:1 to 9:1) afforded **6Ga** (38.3 mg, 91.5 µmol, 46% yield) as a colorless oil. A part of product decomposed during purification (rearomatization). ^{1}H NMR (400 MHz, $CDCl_3$) δ 7.46–7.43 (m, 2H), 7.37–7.30 (m, 3H), 6.06 (d, J = 3.2 Hz, 1H), 5.53 (d, J = 1.6 Hz, 1H), 4.68–4.67 (m, 1H), 4.28–4.16 (m, 4H), 1.47 (s, 3H), 1.30 (t, J = 7.2 Hz, 3H), 1.26 (t, J = 7.2 Hz, 3H), 0.17 (s, 9H); ^{13}C NMR (101 MHz, $CDCl_3$) δ 170.8, 169.8, 154.6, 142.6, 133.5, 128.6, 128.5, 126.4, 123.2, 118.0, 61.6, 61.34, 61.25, 60.2, 14.4, 14.03, 14.00, –1.2; HRMS (ESI) *m/z* calcd for $C_{22}H_{31}O_4SSi$ [M + H]$^+$: 419.1707 found 419.1700.

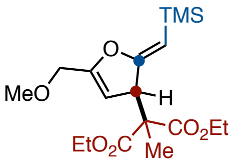

Diethyl (*Z*)-2-(5-(methoxymethyl)-2-((trimethylsilyl)methylene)-2,3-dihydrofuran-3-yl)-2-methylmalonate (6Ha)

^{1}H NMR yield of **6Ha** was 50% (^{1}H NMR peak at 5.09–5.08 ppm (m, 1H) was used). Purification by Isolera® with a basic alumina column cartridge (hexane/EtOAc = 99:1 to 9:1) afforded **6Ha** (36.9 mg, 100 µmol, 50% yield) as a colorless oil. ^{1}H NMR (400 MHz, $CDCl_3$) δ 5.09–5.08 (m, 1H), 4.36–4.34 (m, 2H), 4.29–4.12 (m, 4H), 4.02 (s, 2H), 3.38 (s, 3H), 1.31 (s, 3H), 1.30–1.23 (m, 6H), 0.09 (s, 9H); ^{13}C NMR (101 MHz, $CDCl_3$) δ 170.6, 170.0, 166.0, 154.9, 101.2, 98.1, 66.7, 61.5, 61.4, 58.5, 57.9, 50.9, 15.3, 14.0, –0.5 (one peak is missing due to overlapping); HRMS (ESI) *m/z* calcd for $C_{18}H_{30}O_6SiNa$ [M + Na]$^+$: 393.1704 found 393.1696.

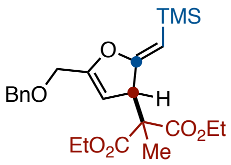

Diethyl (*Z*)-2-(5-((benzyloxy)methyl)-2-((trimethylsilyl)methylene)-2,3-dihydrofuran-3-yl)-2-methylmalonate (6Ia)

^{1}H NMR yield of **6Ia** was 70% (^{1}H NMR peak at 5.40 ppm (s, 1H) was used). Purification by Isolera® with a basic alumina column cartridge (hexane/EtOAc = 99:1 to 9:1) afforded **6Ia** (54.3 mg, 122 µmol, 61% yield) as a colorless oil. A part of product decomposed during purification (rearomatization). ^{1}H NMR (400 MHz, $CDCl_3$) δ 7.37–7.28 (m, 5H), 5.12 (d, J = 2.4 Hz, 1H), 4.57 (s, 2H), 4.37–4.35 (m, 2H), 4.27–4.14 (m, 4H), 4.11 (s, 2H), 1.31 (s, 3H), 1.30–1.23 (m, 6H), 0.10 (s,

9H); ^{13}C NMR (101 MHz, $CDCl_3$) δ 170.6, 170.0, 166.1, 154.9, 137.8, 128.4, 127.8, 101.5, 98.1, 72.4, 64.2, 61.5, 61.4, 57.9, 51.0, 15.3, 14.0, –0.4 (two peaks are missing due to overlapping); HRMS (ESI) *m/z* calcd for $C_{24}H_{34}O_6SiNa$ [M + Na]$^+$: 469.2017 found 469.2011.

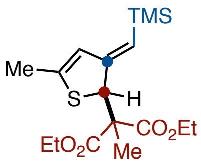

Diethyl (*E*)-2-methyl-2-(5-methyl-3-((trimethylsilyl)methylene)-2,3-dihydrothiophen-2-yl)malonate (6Ja)

^{1}H NMR yield of **6Ja** was 64% (^{1}H NMR peak at 5.89 ppm (s, 1H) was used). Purification by Isolera® with a basic alumina column cartridge (hexane/EtOAc = 99:1 to 9:1) afforded **6Ja** (37.5 mg, 105 μmol, 53% yield) as a colorless oil. A part of product decomposed during purification (rearomatization). ^{1}H NMR (400 MHz, $CDCl_3$) δ 5.89 (s, 1H), 5.03 (s, 1H), 4.94 (s, 1H), 4.26–4.12 (m, 4H), 2.05 (s, 3H), 1.40 (s, 3H), 1.31–1.24 (m, 6H), 0.10 (s, 9H); ^{13}C NMR (101 MHz, $CDCl_3$) δ 170.8, 169.0, 158.4, 149.5, 122.8, 121.4, 61.8, 61.5, 61.0, 58.8, 17.2, 13.99, 13.97, 12.7, –0.1; HRMS (ESI) *m/z* calcd for $C_{17}H_{29}O_4SSi$ [M + H]$^+$: 357.1550 found 357.1547.

5. Pd-Catalyzed three-component C–C bond forming dearomatization using *N*-tosylhydrazones

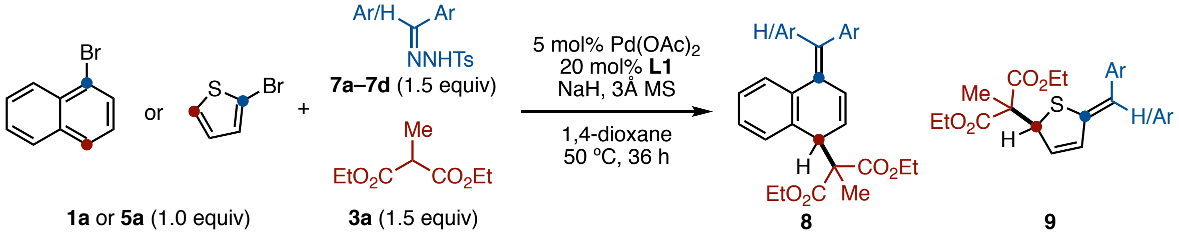

General Procedure C

To an 8-mL glass vessel equipped with a screw cap containing a magnetic stirring bar and 3Å MS (50.0 mg) was dried with a heat-gun *in vacuo* and filled with N_2 after cooling to room temperature. To this vessel were added bromoarene **1a** or **5a** (0.20 mmol, 1.0 equiv), $Pd(OAc)_2$ (2.3 mg, 0.010 mmol, 5.0 mol%), 4-(dimethylamino)phenyldiphenylphosphine (**L1**: 12.2 mg, 0.040 mmol, 20 mol%), sodium hydride (60%, dispersion in paraffin liquid: 24.0 mg, 0.60 mmol, 3.0 equiv), tosylhydrazones **7** (0.30 mmol, 1.5 equiv) and malonate **3a** (52.3 mg, 0.30 mmol, 1.5 equiv). The vessel was placed under vacuum and refilled N_2 gas three times, and then added toluene (1.0 mL). The vessel was sealed with a screw cap and then heated at 50 °C for 36 h with stirring. After cooling the reaction mixture to room temperature, the mixture was passed through a short alumina pad with hexane/EtOAc (1:1) as an eluent. The filtrate was concentrated *in vacuo*. The yield of **8** or **9** was determined by ^{1}H NMR analysis using CH_2Br_2 as an internal standard.

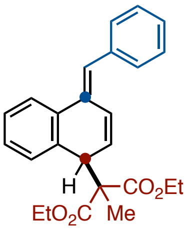

Diethyl (*E*)-2-(4-benzylidene-1,4-dihydronaphthalen-1-yl)-2-methylmalonate (8Aa) [12]

^{1}H NMR yield of **8Aa** was 43% (^{1}H NMR peak at 4.64 ppm (d, *J* = 5.2 Hz, 1H) was used). Purification by PTLC (hexane/EtOAc = 19:1) afforded **8Aa** (28.8 mg, 73.8 µmol, 37% yield) as a colorless oil. A part of product decomposed during purification (rearomatization). ^{1}H NMR (400 MHz, $CDCl_3$) δ 7.83 (d, *J* = 8.0 Hz, 1H), 7.43–7.37 (m, 4H), 7.34–7.28 (m, 2H), 7.23–7.21 (m, 2H), 7.17 (s, 1H), 7.02 (d, *J* = 10.4 Hz, 1H), 6.33–6.29 (m, 1H), 4.64 (d, *J* = 5.2 Hz, 1H), 4.30–4.11 (m, 4H), 1.28 (t, *J* = 7.2 Hz, 3H), 1.24 (t, *J* = 7.2 Hz, 3H), 1.19 (s, 3H); ^{13}C NMR (101 MHz, $CDCl_3$) δ 170.8, 137.2, 136.5, 134.1, 132.2, 129.5, 129.2, 128.9, 128.8, 128.2, 127.0, 126.8, 124.0, 123.1, 61.5, 61.4, 45.1, 14.9, 14.0, 13.9 (three peaks are missing due to overlapping); HRMS (ESI) *m/z* calcd for $C_{25}H_{26}O_4Na$ $[M + Na]^+$: 413.1723 found 413.1719.

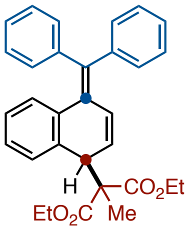

Diethyl 2-(4-(diphenylmethylene)-1,4-dihydronaphthalen-1-yl)-2-methylmalonate (8Ab)

^{1}H NMR yield of **8Ab** was 45% (^{1}H NMR peak at 4.59 ppm (d, *J* = 5.2 Hz, 1H) was used). Purification by PTLC (hexane/EtOAc = 19:1) afforded **8Ab** (35.8 mg, 76.7 µmol, 38% yield) as a white solid. A part of product decomposed during purification (rearomatization). ^{1}H NMR (400 MHz, $CDCl_3$) δ 7.37–7.33 (m, 2H), 7.30–7.26 (m, 1H), 7.24–7.17 (m, 7H), 7.15–7.13 (m, 1H), 7.07–7.04 (m, 2H), 6.88–6.83 (m, 2H), 6.09 (dd, *J* = 10.0, 5.2 Hz, 1H), 4.59 (d, *J* = 5.2 Hz, 1H), 4.29–4.14 (m, 4H), 1.39 (s, 3H), 1.30–1.24 (m, 6H); ^{13}C NMR (101 MHz, $CDCl_3$) δ 170.8, 170.6, 143.3, 143.2, 138.7, 136.4, 136.1, 133.2, 131.0, 130.2, 130.0, 128.9, 128.4, 128.3, 127.2, 127.1, 127.0, 126.4, 125.7, 61.49, 61.47, 61.2, 46.1, 15.0, 14.02, 13.98 (one peak is missing due to overlapping); HRMS (ESI) *m/z* calcd for $C_{31}H_{30}O_4Na$ $[M + Na]^+$: 489.2036 found 489.2035.

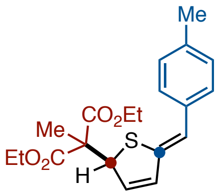

Diethyl (*Z*)-2-methyl-2-(5-(4-methylbenzylidene)-2,5-dihydrothiophen-2-yl)malonate (9Ac)

^{1}H NMR yield of **9Ac** was 44%, *E*/*Z* = 7:93 (^{1}H NMR peak at 5.33 ppm (s, 0.93H) and 5.23 ppm (s, 0.07H) was used). Purification by PTLC (hexane/EtOAc = 19:1) afforded **9Ac** (29.4 mg, 81.6 µmol, 41% yield) as a colorless oil. ^{1}H NMR (400 MHz, $CDCl_3$) δ 7.38 (d, *J* = 8.0 Hz, 2H), 7.16 (d, *J* = 8.0 Hz, 2H), 6.50–6.47 (m, 2H), 5.99–5.97 (m, 1H), 5.33 (s, 1H), 4.31–4.18 (m, 4H), 2.34 (s, 3H), 1.41 (s, 3H), 1.30–1.26 (m, 6H); ^{13}C NMR (101 MHz, $CDCl_3$) δ 170.7, 170.3, 142.6, 137.2, 136.2, 134.2, 131.0, 129.1, 127.8, 119.1, 61.9, 61.7, 58.1, 21.3, 15.1, 14.04, 14.01 (one peak is missing due to overlapping); HRMS (ESI) *m*/*z* calcd for $C_{20}H_{24}O_4SNa$ $[M + Na]^+$: 383.1288 found 383.1288.

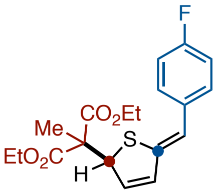

9Ad

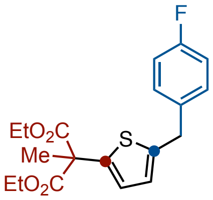

9Ad'

Diethyl (*Z*)-2-(5-(4-fluorobenzylidene)-2,5-dihydrothiophen-2-yl)-2-methylmalonate (9Ad)

^{1}H NMR yield of **9Ad** was 50%, *E*/*Z* = 6:94 (^{1}H NMR peak at 5.34 ppm (s, 0.94H) and 5.23 ppm (s, 0.06H) was used). Purification by PTLC (hexane/EtOAc = 19:1) afforded a mixture of **9Ad** and rearomatized compound **9Ad'** (31.0 mg, 85.1 µmol, 43% yield, **9Ad**:**9Ad'** = 86:14) and the yield of **9Ad** was determined as 37%. Further purification by GPC was performed to give pure **9Ad** as a colorless oil for the characterization. A part of product decomposed during purification (rearomatization). ^{1}H NMR (400 MHz, $CDCl_3$) δ 7.47–7.42 (m, 2H), 7.07–7.01 (m, 2H), 6.49–6.47 (m, 2H), 6.01 (dd, *J* = 6.4, 2.8 Hz, 1H), 5.34 (s, 1H), 4.32–4.19 (m, 4H), 1.41 (s, 3H), 1.28 (t, *J* = 7.2 Hz, 6H); ^{13}C NMR (101 MHz, $CDCl_3$) δ 170.7, 170.2, 161.1 (d, $J_{C\text{–}F}$ = 248 Hz), 143.2, 137.1, 133.3 (d, $J_{C\text{–}F}$ = 2.9 Hz), 131.5, 129.4 (d, $J_{C\text{–}F}$ = 7.7 Hz), 117.9, 115.4 (d, $J_{C\text{–}F}$ = 22.2 Hz), 61.9, 61.7, 58.0, 15.2, 14.04, 14.02 (one peak is missing due to overlapping); HRMS (ESI) *m*/*z* calcd for $C_{19}H_{22}FO_4S$ $[M + H]^+$: 365.1217 found 365.1214.

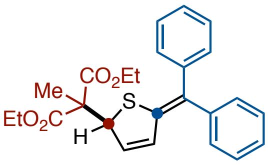

Diethyl 2-(5-(diphenylmethylene)-2,5-dihydrothiophen-2-yl)-2-methylmalonate (9Ab)

^{1}H NMR yield of **9Ab** was 56% (^{1}H NMR peak at 5.26–5.24 ppm (m, 1H) was used). Purification by PTLC (hexane/EtOAc = 19:1) afforded **9Ab** (32.0 mg, 75.7 µmol, 38% yield) as a white solid. A part of product decomposed during purification (rearomatization). ^{1}H NMR (400 MHz, $CDCl_3$) δ 7.38–7.35 (m, 2H), 7.33–7.25 (m, 5H), 7.23–7.19 (m, 1H), 7.16–7.14 (m, 2H), 6.53 (dd, *J* = 6.4, 2.0 Hz, 1H), 6.01 (dd, *J* = 6.4, 3.2 Hz, 1H), 5.26–5.24 (m, 1H), 4.25–4.13 (m, 4H), 1.46 (s, 3H), 1.28–1.22 (m, 6H); ^{13}C NMR (101 MHz, $CDCl_3$) δ 170.5, 170.3, 142.6, 142.3, 142.0, 134.0, 133.4, 130.6, 130.2, 129.1, 128.15, 128.07, 127.0, 126.9, 61.8, 59.6, 58.1, 15.3, 14.03, 13.97 (one peak is missing due to overlapping); HRMS (ESI) *m/z* calcd for $C_{25}H_{26}O_4SNa$ $[M + Na]^+$: 445.1444 found 445.1442.

6. Derivatization of products

Diimide reduction of 4Aa for the synthesis of diethyl 2-methyl-2-((1*R,4*S**)-4-((trimethylsilyl) methyl)-1,4-dihydronaphthalen-1-yl)malonate (10)**

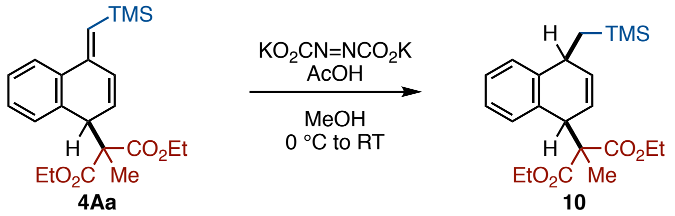

To a 20-mL glass drum-vial containing magnetic stirring bar and the solution of **4Aa** (190 mg, 491 μmol, 1.0 equiv) in MeOH (6.0 mL) was added potassium diazocarboxylate (381 mg, 2.0 mmol, 4.0 equiv). The mixture was cooled at 0 °C, and then AcOH (223 μL, 3.9 mmol, 8.0 equiv) was slowly added. After stirring 1 h at room temperature, potassium diazocarboxylate (381 mg, 2.0 mmol, 4.0 equiv) and AcOH (223 μL, 3.9 mmol, 8.0 equiv) were added again in this order at 0 °C. The mixture was further stirred at room temperature until the color of suspension turned to white from yellow. The mixture was slowly quenched with $NaHCO_3$ aq. and extracted three times with EtOAc. Combined organic layer was dried over Na_2SO_4, filtrated, and concentrated *in vacuo*. The mixture was purified by Isolera® with basic alumina column cartridge (hexane/EtOAc = 99:1 to 4:1) to afford **10** (189 mg, 485 μmol, 99% yield) as a colorless oil. ^{1}H NMR (400 MHz, $CDCl_3$) δ 7.21–7.14 (m, 2H), 7.09–7.03 (m, 2H), 6.09 (dd, *J* = 10.4, 4.4 Hz, 1H), 5.87 (dd, *J* = 10.4, 4.4 Hz, 1H), 4.56 (t, *J* = 4.4 Hz, 1H), 4.29–4.16 (m, 4H), 3.45–3.42 (m, 1H), 1.31–1.22 (m, 7H), 1.17 (s, 3H), 0.94 (dd, *J* = 14.4, 12.0 Hz, 1H), 0.13 (s, 9H); ^{13}C NMR (101 MHz, $CDCl_3$) δ 171.3, 170.9, 143.5, 133.8, 132.7, 128.3, 128.2, 126.9, 125.3, 124.1, 61.43, 61.39, 59.2, 43.2, 36.0, 27.9, 16.2, 14.0, 13.9, –0.7; HRMS (ESI) *m/z* calcd for $C_{22}H_{32}O_4NaSi$ $[M + Na]^+$: 411.1962 found 411.1958.

Bromolactonization of 10 for the synthesis of ethyl (1*R,3a*S**,4*S**,5*S**,9b*R**)-4-bromo-1-methyl -2-oxo-5-((trimethylsilyl)methyl)-1,2,3a,4,5,9b-hexahydronaphtho[2,1-*b*]furan-1-carboxylate (11)**

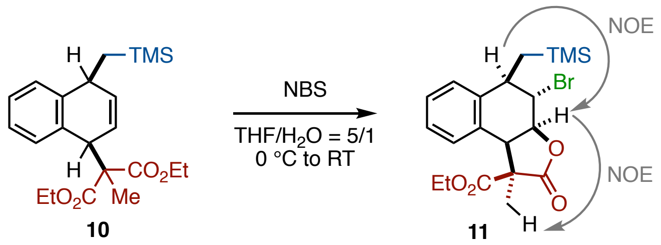

To an 8-mL glass tube containing magnetic stirring bar was added **10** (18.2 mg, 46.8 μmol, 1.0 equiv), THF (0.50 mL), water (0.10 mL), and then *N*-bromosuccinimide (NBS: 10.0 mg, 56.2 μmol, 1.2 equiv) at 0 °C. After warming the mixture to room temperature and stirring for 2 h, to the mixture were slowly added $NaHCO_3$ aq. and $Na_2S_2O_3$ aq. The mixture was extracted three times with EtOAc. Combined organic layer was dried over $MgSO_4$, filtrated, and concentrated *in vacuo*. The mixture was purified by PTLC (hexane/EtOAc = 9:1) to afford **11** (13.6 mg, 31.0 μmol, 66% yield) as a colorless

oil. ^{1}H NMR (400 MHz, $CDCl_3$) δ 7.29–7.26 (m, 2H), 7.18–7.15 (m, 1H), 7.05–7.03 (m, 1H), 5.32 (dd, J = 8.8, 4.0 Hz, 1H), 4.65 (t, J = 4.0 Hz, 1H), 4.36–4.30 (m, 3H), 3.38 (td, J = 7.2, 4.0 Hz, 1H), 1.36 (t, J = 7.2 Hz, 3H), 1.22–1.16 (m, 4H), 0.93 (dd, J = 15.2, 6.8 Hz, 1H), 0.09 (s, 9H); ^{13}C NMR (101 MHz, $CDCl_3$) δ 174.2, 170.8, 139.3, 130.8, 129.5, 128.6, 127.8, 126.9, 79.8, 62.7, 55.3, 51.4, 44.5, 42.3, 25.5, 18.6, 14.0, –0.6; HRMS (ESI) *m/z* calcd for $C_{20}H_{27}BrO_4NaSi$ $[M + Na]^+$: 461.0754 found 461.0750.

Epoxidation of 10 for the synthesis of diethyl 2-methyl-2-((1a*R,2*R**,7*R**,7a*S**)-7-((trimethylsilyl)methyl)-1a,2,7,7a-tetrahydronaphtho[2,3-*b*]oxiren-2-yl)malonate (12)**

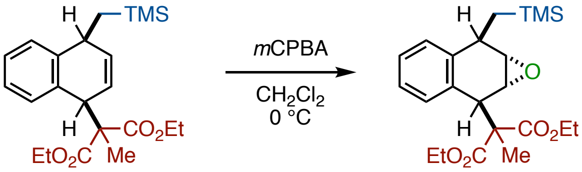

10 **12**

To an 8-mL glass tube containing magnetic stirring bar were added **10** (28.2 mg, 72.6 µmol, 1.0 equiv), CH_2Cl_2 (1.0 mL), and then *m*-chloroperbenzoic acid (*m*CPBA, 77% purity: 12.5 mg, 72.6 µmol, 1.0 equiv) at 0 °C. After stirring at 0 °C overnight, to the mixture were slowly added $NaHCO_3$ aq. and then $Na_2S_2O_3$ aq. The mixture was extracted three times with CH_2Cl_2. Combined organic layer was dried over Na_2SO_4, filtrated, and concentrated *in vacuo*. The mixture was purified by PTLC (hexane/EtOAc = 3:1) to afford **12** (18.0 mg, 44.5 µmol, 61% yield) as a colorless oil. ^{1}H NMR (400 MHz, $CDCl_3$) δ 7.18 (t, J = 8.0 Hz, 1H), 7.04 (t, J = 8.0 Hz, 1H), 7.02 (d, J = 8.0 Hz, 1H), 6.94 (d, J = 8.0 Hz, 1H), 4.43 (s, 1H), 4.31–4.15 (m, 4H), 3.53 (dt, J = 4.0, 1.2 Hz, 1H), 3.42 (dd, J = 7.6, 6.0 Hz, 1H), 3.22 (d, J = 4.0 Hz, 1H), 1.34 (s, 3H), 1.30 (t, J = 7.2 Hz, 3H), 1.26 (t, J = 7.2 Hz, 3H), 1.12–1.03 (m, 2H), 0.16 (s, 9H); ^{13}C NMR (101 MHz, $CDCl_3$) δ 170.9, 170.6, 140.0, 130.1, 129.9, 129.1, 127.9, 126.0, 61.8, 61.7, 57.8, 55.6, 54.1, 42.9, 35.1, 23.5, 17.8, 14.0, 13.8, –1.0; HRMS (ESI) *m/z* calcd for $C_{22}H_{33}O_5Si$ $[M + H]^+$: 405.2092 found 405.2089.

Lactonization of 12 for the synthesis of hydroxy lactone (13)

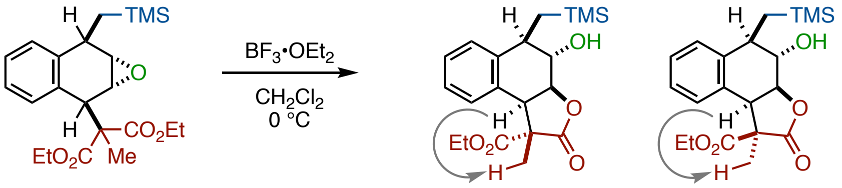

12 **13** **13'**

To an 8-mL glass tube containing magnetic stirring bar were added **12** (33.4 mg, 82.6 µmol, 1.0 equiv), CH_2Cl_2 (1.0 mL), and $BF_3{\cdot}OEt_2$ (12.5 mg, 165 µmol, 2.0 equiv) at 0 °C. After stirring at 0 °C for 5 min, the mixture was diluted with Et_2O and added $NaHCO_3$ aq. to quench the reaction. The mixture was extracted three times with CH_2Cl_2. Combined organic layer was dried over Na_2SO_4,

filtrated, and concentrated *in vacuo*. The mixture was purified by PTLC (hexane/EtOAc = 5:1) to afford a mixture of **13** and **13’** (20.9 mg, 55.5 μmol, 67% yield, **13**:**13’** = 71:29) as a yellow oil.

Ethyl (1*S,3a*S**,4*S**,5*R**,9b*R**)-4-hydroxy-1-methyl-2-oxo-5-((trimethylsilyl)methyl)-1,2,3a,4,5,9b-hexahydronaphtho[2,1-*b*]furan-1-carboxylate (13)**: ^{1}H NMR (400 MHz, $CDCl_3$) δ 7.31–7.28 (m, 2H), 7.24–7.20 (m, 1H), 6.94 (d, *J* = 8.0 Hz, 1H), 4.83 (t, *J* = 4.8 Hz, 1H), 4.53 (d, *J* = 8.8 Hz, 1H), 4.36 (q, *J* = 7.2 Hz, 2H), 3.83 (dt, *J* = 8.8, 3.2 Hz, 1H), 2.99–2.94 (m, 1H), 2.29–2.28 (m, 1H), 1.36 (t, *J* = 7.2 Hz, 3H), 1.28 (dd, *J* = 15.6, 6.0 Hz, 1H), 1.20 (s, 3H), 1.16 (dd, *J* = 15.6, 6.0 Hz, 1H), 0.04 (s, 9H); ^{13}C NMR (101 MHz, $CDCl_3$) δ 175.3, 171.1, 139.3, 129.9, 129.3, 128.4, 127.7, 126.9, 81.8, 73.0, 62.7, 54.3, 45.6, 39.4, 18.2, 18.1, 14.0, –0.1; HRMS (ESI) *m/z* calcd for $C_{20}H_{28}O_5NaSi$ $[M + Na]^+$: 399.1598 found 399.1596.

Ethyl (1*R,3a*S**,4*S**,5*R**,9b*R**)-4-hydroxy-1-methyl-2-oxo-5-((trimethylsilyl)methyl)-1,2,3a,4,5,9b-hexahydronaphtho[2,1-*b*]furan-1-carboxylate (13’)**: ^{1}H NMR (400 MHz, $CDCl_3$) δ 7.29–7.27 (m, 1H), 7.26–7.23 (m, 1H), 7.20–7.15 (m, 2H), 4.72 (dd, *J* = 9.6, 8.8 Hz, 1H), 4.14 (dt, *J* = 9.6, 3.2 Hz, 1H), 3.97 (d, *J* = 9.6 Hz, 1H), 3.76 (q, *J* = 7.2 Hz, 2H), 2.86–2.81 (m, 1H), 2.39 (d, *J* = 3.2 Hz, 1H), 1.87 (s, 3H), 1.34 (dd, *J* = 15.2, 5.2 Hz, 1H), 1.15 (dd, *J* = 15.2, 5.2 Hz, 1H), 0.78 (t, *J* = 7.2 Hz, 3H), 0.08 (s, 9H); ^{13}C NMR (101 MHz, $CDCl_3$) δ 175.6, 168.6, 139.4, 130.8, 128.1, 127.9, 127.8, 126.5, 82.3, 72.5, 61.9, 56.1, 49.2, 38.8, 24.4, 17.7, 13.6, –0.1; HRMS (ESI) *m/z* calcd for $C_{20}H_{28}O_5NaSi$ $[M + Na]^+$: 399.1598 found 399.1598.

Diimide reduction of 4Ie for the synthesis of diethyl 2-((1*S,2*S**)-2-((trimethylsilyl)methyl)-1,2-dihydronaphthalen-1-yl)malonate (14)**

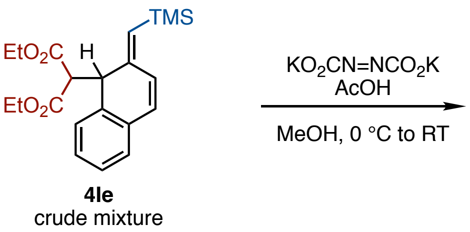

To a crude mixture of **4Ie** (NMR yield was 61%, 0.20 mmol scale) in a 20 mL drum-vial with a magnetic stirring bar were added potassium diazocarboxylate (155 mg, 0.80 mmol, 4.0 equiv) and MeOH (2.0 mL). The mixture was cooled at 0 °C, and then AcOH (90.9 μL, 1.6 mmol, 8.0 equiv) was slowly added. After stirring 1 h at room temperature, potassium diazocarboxylate (155 mg, 0.80 mmol, 4.0 equiv) and AcOH (90.9 μL, 1.6 mmol, 8.0 equiv) were added again in this order at 0 °C. The mixture was further stirred at room temperature until the color of suspension turned to white from yellow. The mixture was slowly quenched with $NaHCO_3$ aq. and extracted three times with EtOAc. Combined organic layer was dried over Na_2SO_4, filtrated, and concentrated *in vacuo*. The mixture was purified by PTLC (hexane/EtOAc = 19:1, three times) to afford **14** (34.3 mg, 46% yield in 2 steps (for this reduction, 75% yield)) as a colorless oil. ^{1}H NMR (400 MHz, $CDCl_3$) δ 7.17 (td, *J* = 7.2, 2.0 Hz,

1H), 7.12–7.07 (m, 2H), 7.03 (d, J = 7.2 Hz, 1H), 6.47 (dd, J = 9.6, 2.8 Hz, 1H), 5.78 (dd, J = 9.6, 2.8 Hz, 1H), 4.28–4.15 (m, 2H), 3.90 (d, J = 8.8 Hz, 1H), 3.86–3.69 (m, 2H), 3.65 (dd, J = 8.8, 5.2 Hz, 1H), 2.85–2.73 (m, 1H), 1.28 (t, J = 7.2 Hz, 3H), 0.97 (t, J = 7.2 Hz, 3H), 0.68 (dd, J = 14.0, 3.2 Hz, 1H), 0.59 (t, J = 14.0 Hz, 1H), 0.01 (s, 9H); ^{13}C NMR (101 MHz, $CDCl_3$) δ 169.3, 168.6, 135.6, 134.2, 133.3, 127.8, 127.3, 126.8, 125.7, 61.5, 61.2, 50.8, 44.3, 34.3, 16.9, 14.0, 13.7, –0.8 (one peak is missing due to overlapping); HRMS (DART) *m/z* calcd for $C_{21}H_{30}O_4SiNa$ $[M + Na]^+$: 397.1806 found 397.1809.

Epoxidation of 14 for the synthesis of diethyl 2-((1a*S,2*R**,3*S**,7b*R**)-2-((trimethylsilyl)methyl)-1a,2,3,7b-tetrahydronaphtho[1,2-*b*]oxiren-3-yl)malonate (15)**

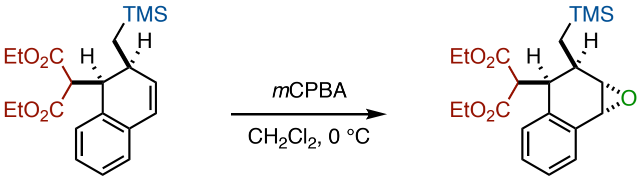

To a solution of **14** (13.0 mg, 34.7 µmol, 1.0 equiv) in CH_2Cl_2 (1.0 mL) was added *m*-chloroperbenzoic acid (*m*CPBA, 77% purity: 8.6 mg, 38.2 µmol, 1.1 equiv) at 0 °C. After stirring the solution at 0 °C for several hours with monitoring reaction progress by TLC, $NaHCO_3$ aq. and $Na_2S_2O_3$ aq. were added to quench the reaction. The mixture was extracted three times with CH_2Cl_2, dried over Na_2SO_4, filtrated, and then concentrated *in vacuo*. Purification by PTLC (hexane/EtOAc = 9:1) afforded **15** (9.3 mg, 23.8 µmol, 69% yield) as a colorless oil. ^{1}H NMR (400 MHz, $CDCl_3$) δ 7.41 (dd, J = 7.2, 2.0 Hz, 1H), 7.29–7.21 (m, 2H), 6.84 (d, J = 7.2 Hz, 1H), 4.32–4.17 (m, 4H), 3.92 (dd, J = 12.0, 4.0 Hz, 1H), 3.87 (d, J = 4.0 Hz, 1H), 3.84 (d, J = 12.0 Hz, 1H), 3.60 (t, J = 4.0 Hz, 1H), 2.50–2.44 (m, 1H), 1.33 (t, J = 7.2 Hz, 3H), 1.25 (t, J = 7.2 Hz, 3H), 0.61 (dd, J = 14.8, 2.8 Hz, 1H), 0.02 (s, 9H), –0.13 (dd, J = 14.8, 12.0 Hz, 1H); ^{13}C NMR (101 MHz, $CDCl_3$) δ 168.5, 168.2, 135.9, 132.5, 129.4, 128.6, 126.4, 125.4, 61.8, 57.6, 53.8, 53.1, 37.8, 31.5, 14.1, 14.0, 12.9, –0.5 (one peak is missing due to overlapping); HRMS (ESI) *m/z* calcd for $C_{21}H_{30}O_5SiNa$ $[M + Na]^+$: 413.1755 found 413.1751.

Bromohydrin formation (synthesis of 16)

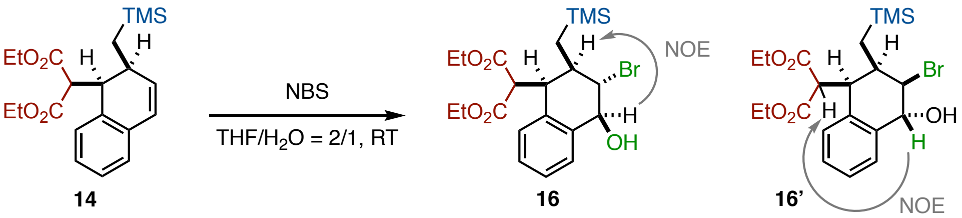

To a solution of **14** (19.0 mg, 50.7 µmol, 1.0 equiv) in THF (0.80 mL) and H_2O (0.40 mL) was added *N*-bromosuccinimide (NBS: 9.9 mg 55.8 µmol, 1.1 equiv) at 0 °C. After stirring the solution at

room temperature for several hours with monitoring reaction progress by TLC, water was added to quench the reaction. The mixture was extracted three times with EtOAc, dried over $MgSO_4$, filtrated, and then concentrated *in vacuo*. Purification by PTLC (hexane/EtOAc = 9:1, two times) afforded **16** (13.1 mg, 27.8 μmol, 55% yield) as a colorless oil and diastereo isomer **16'** (4.0 mg, 8.5 μmol, 17% yield) as a colorless oil.

Diethyl 2-((1*S,2*S**,3*S**,4*S**)-3-bromo-4-hydroxy-2-((trimethylsilyl)methyl)-1,2,3,4-tetrahydronaphthalen-1-yl)malonate (16)**: ^{1}H NMR (400 MHz, $CDCl_3$) δ 7.58 (d, J = 7.6 Hz, 1H), 7.31 (t, J = 7.6 Hz, 1H), 7.24 (t, J = 7.6 Hz, 1H), 7.09 (d, J = 7.6 Hz, 1H), 5.03 (dd, J = 7.6, 4.0 Hz, 1H), 4.38–4.30 (m, 1H), 4.26–4.18 (m, 1H), 4.03–3.87 (m, 5H), 2.85 (d, J = 4.0 Hz, 1H), 2.59–2.53 (m, 1H), 1.33 (t, J = 7.2 Hz, 3H), 1.10 (t, J = 7.2 Hz, 3H), 0.40 (dd, J = 14.8, 5.6 Hz, 1H), 0.35–0.25 (m, 1H), 0.03 (s, 9H); ^{13}C NMR (101 MHz, $CDCl_3$) δ 168.32, 168.28, 136.8, 136.0, 127.2, 125.8, 125.2, 75.5, 64.7, 62.0, 61.9, 52.8, 42.5, 41.4, 18.9, 14.1, 13.8, –1.0 (one peak is missing due to overlapping); HRMS (ESI) *m/z* calcd for $C_{21}H_{31}BrO_5SiNa$ $[M + Na]^+$: 493.1016 found 493.1016.

Diethyl 2-((1*S,2*S**,3*R**,4*R**)-3-bromo-4-hydroxy-2-((trimethylsilyl)methyl)-1,2,3,4-tetrahydronaphthalen-1-yl)malonate (16')**: ^{1}H NMR (400 MHz, $CDCl_3$) δ 7.51 (d, J = 7.6 Hz, 1H), 7.30 (t, J = 7.6 Hz, 1H), 7.21 (t, J = 7.6 Hz, 1H), 7.08 (d, J = 7.6 Hz, 1H), 5.27 (t, J = 5.2 Hz, 1H), 4.49 (t, J = 5.2 Hz, 1H), 4.39 (d, J = 8.8 Hz, 1H), 4.35–4.22 (m, 2H), 3.95 (dd, J = 8.8, 4.0 Hz, 1H), 3.87–3.78 (m, 1H), 3.75–3.66 (m, 1H), 2.52–2.46 (m, 2H), 1.32 (t, J = 7.2 Hz, 3H), 0.98 (dd, J = 14.4, 10.8 Hz, 1H), 0.88 (t, J = 7.2 Hz, 3H), 0.69 (dd, J = 14.4, 3.2 Hz, 1H), 0.07 (s, 9H); ^{13}C NMR (101 MHz, $CDCl_3$) δ 169.5, 168.5, 137.0, 135.8, 128.5, 127.7, 127.6, 127.1, 75.0, 62.5, 61.8, 61.3, 51.7, 45.1, 36.7, 18.3, 14.1, 13.5, –0.6; HRMS (ESI) *m/z* calcd for $C_{21}H_{32}BrO_5Si$ $[M + H]^+$: 471.1197 found 471.1202.

Hydrogenation of 14 for the synthesis of diethyl 2-((1*S,2*S**)-2-((trimethylsilyl)methyl)-1,2,3,4-tetrahydronaphthalen-1-yl)malonate (17)**

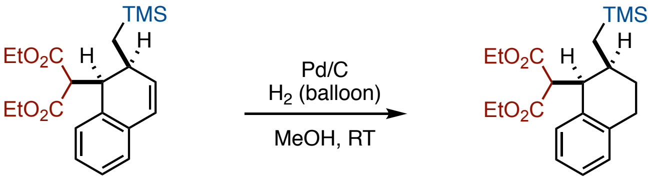

14 **17**

To a solution of **14** (15.6 mg, 41.6 μmol, 1.0 equiv) in MeOH (0.40 mL) was added Pd/C (10 wt%, 1.6 mg, 1.5 μmol, 3.6 mol%) and equipped with H_2 balloon (1 atm) then stirred for overnight at room temperature. The mixture was passed through a pad of Celite® and filtrate was concentrated *in vacuo* to afford **17** (14.0 mg, 37.2 μmol, 89% yield) as a colorless oil. ^{1}H NMR (400 MHz, $CDCl_3$) δ 7.12–7.09 (m, 2H), 7.06–7.02 (m, 2H), 4.26 (q, J = 7.2 Hz, 2H), 3.74–3.66 (m, 3H), 3.62–3.54 (m, 1H), 2.90–2.77 (m, 2H), 2.12–2.04 (m, 1H), 1.87–1.80 (m, 1H), 1.75–1.64 (m, 1H), 1.30 (t, J = 7.2 Hz, 3H),

0.88 (t, J = 7.2 Hz, 3H), 0.57 (dd, J = 14.4, 2.4 Hz, 1H), 0.41 (dd, J = 14.4, 11.2 Hz, 1H), 0.01 (s, 9H); ^{13}C NMR (100 MHz, $CDCl_3$) δ 170.0, 169.0, 137.6, 136.9, 129.5, 128.4, 126.7, 124.9, 61.5, 61.0, 53.3, 44.7, 34.0, 28.3, 26.0, 20.4, 14.0, 13.5, –0.7; HRMS (ESI) *m/z* calcd for $C_{21}H_{32}O_4Na$ $[M + Na]^+$: 399.1962 found 399.1964.

***m*CPBA Oxidation of 6Aa for the synthesis of diethyl (*E*)-2-(1,1-dioxido-5-((trimethylsilyl) methylene)-2,5-dihydrothiophen-2-yl)-2-methylmalonate (18)**

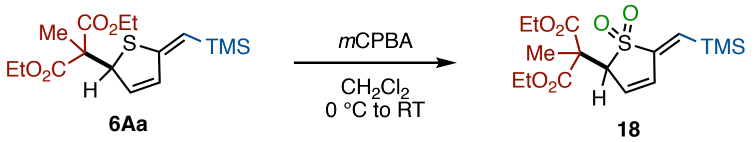

To a solution of **6Aa** (35.0 mg, 102 μmol, 1.0 equiv, *E*/*Z* = 84:16) in a 20 mL drum-vial with a magnetic stirring bar was added CH_2Cl_2 (1.0 mL). The mixture was cooled to 0 °C, and then *m*-chloroperbenzoic acid (*m*CPBA, 77% purity: 28.6 mg, 128 μmol, 1.25 equiv) was added. After stirring 1 h at 0 °C, *m*CPBA (77% purity: 28.6 mg, 128 μmol, 1.25 equiv) was added again at 0 °C. The mixture was stirred for 4 h at room temperature. The mixture was quenched with $NaHCO_3$ aq. at 0 °C and extracted three times with CH_2Cl_2. Combined organic layer was dried over Na_2SO_4, filtrated, and concentrated *in vacuo*. The mixture was purified by PTLC (hexane/EtOAc = 9:1) to afford **6Aa** (31.1 mg, 83.0 μmol, 81% yield, *E*/*Z* = 85:15) as a colorless oil. ^{1}H NMR (400 MHz, $CDCl_3$) δ 6.84–6.82 (m, 0.85H), 6.55–6.52 (m, 0.15H), 6.44 (s, 0.85H), 6.42–6.39 (m, 0.85H), 6.23–6.21 (m, 0.30H), 4.61 (t, J = 2.8 Hz, 0.15H), 4.58 (t, J = 2.8 Hz, 0.85H), 4.37–4.17 (m, 4H), 1.56 (s, 0.45H), 1.55 (s, 2.55H), 1.35–1.30 (m, 3H), 1.29–1.25 (m, 3H), 0.28 (s, 1.35H), 0.23 (s, 7.65H); ^{13}C NMR (101 MHz, $CDCl_3$) δ 169.2, 169.1, 168.73, 168.69, 153.5, 152.8, 134.5, 131.8, 131.0, 130.9, 128.4, 127.8, 67.3, 65.1, 62.6, 62.5, 62.4, 55.3, 55.2, 16.2, 16.1, 14.0, 13.9, –0.4, –0.6 (several peaks are missing due to overlapping); HRMS (ESI) *m/z* calcd for $C_{16}H_{27}O_6SSi$ $[M + H]^+$: 375.1292 found 375.1289.

7. Attempts toward asymmetric dearomatization

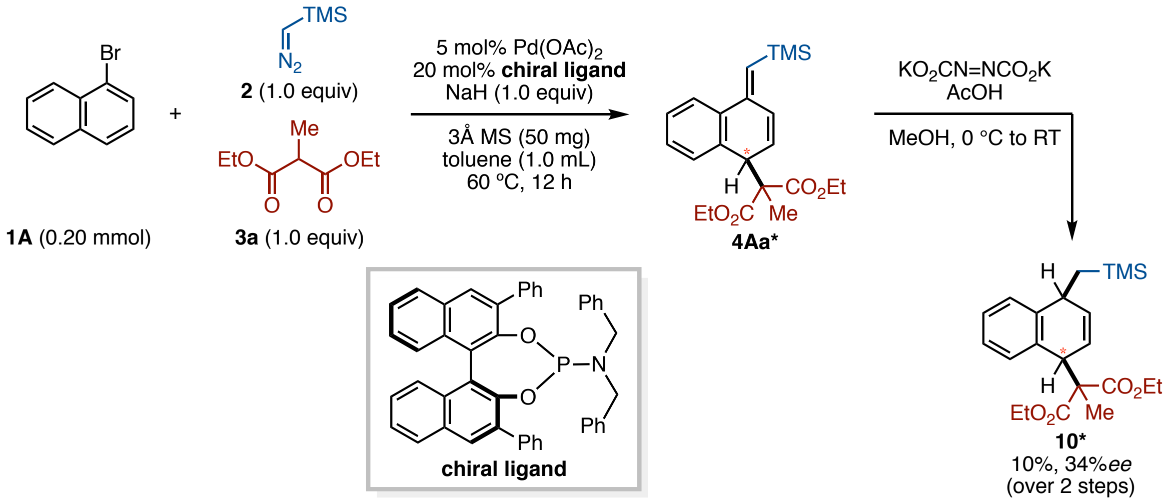

Following the General Procedure A, the reaction of **1A**, **2**, and **3a** was conducted by using (11b*S*)-*N*,*N*-dibenzyl-2,6-diphenyldinaphtho[2,1-*d*:1',2'-*f*][1,3,2]dioxaphosphepin-4-amine (26.6 mg, 0.040 mmol, 20 mol%) as a chiral ligand, ^{1}H NMR yield of **4Aa*** was 13% (^{1}H NMR peak at 4.60 ppm (d, J = 5.6 Hz, 1H) was used). The crude material was used next step without further purification.

To a crude mixture of **4Aa*** (NMR yield was 13%, 0.026 mmol) in a 20 mL drum-vial with a magnetic stirring bar were added potassium diazocarboxylate (51.8 mg, 0.27 mmol, 10.3 equiv, based on the amount of **4Aa***) and MeOH (2.0 mL). The mixture was cooled to 0 °C, and then AcOH (30.3 µL, 0.53 mmol, 20.4 equiv) was slowly added. After stirring 1 h at room temperature, potassium diazocarboxylate (51.8 mg, 0.27 mmol, 10.3 equiv) and AcOH (30.3 µL, 0.53 mmol, 20.4 equiv) were added again in this order at 0 °C. After stirring 1 h at room temperature, potassium diazocarboxylate (51.8 mg, 0.27 mmol, 10.3 equiv) and AcOH (30.3 µL, 0.53 mmol, 20.4 equiv) were added again in this order at 0 °C (total three portions). The mixture was further stirred at room temperature until the color of suspension turned to white from yellow. The mixture was slowly quenched with $NaHCO_3$ aq. and extracted three times with EtOAc. Combined organic layer was dried over Na_2SO_4, filtrated, and concentrated *in vacuo*. The mixture was purified by PTLC (hexane/EtOAc = 19:1, three times) to afford pure **10*** (7.7 mg, 10% yield, 34% *ee* in 2 steps) as a colorless oil. Enantiomeric excess 34% was determined by chiral HPLC analysis: Chiralpak OD-3, hexane/IPA 99:1, 1.0 mL/min, 40 °C, detection at 220 nm, retention time (min): 5.67 (minor) and 6.00 (major).

Chromatogram Report

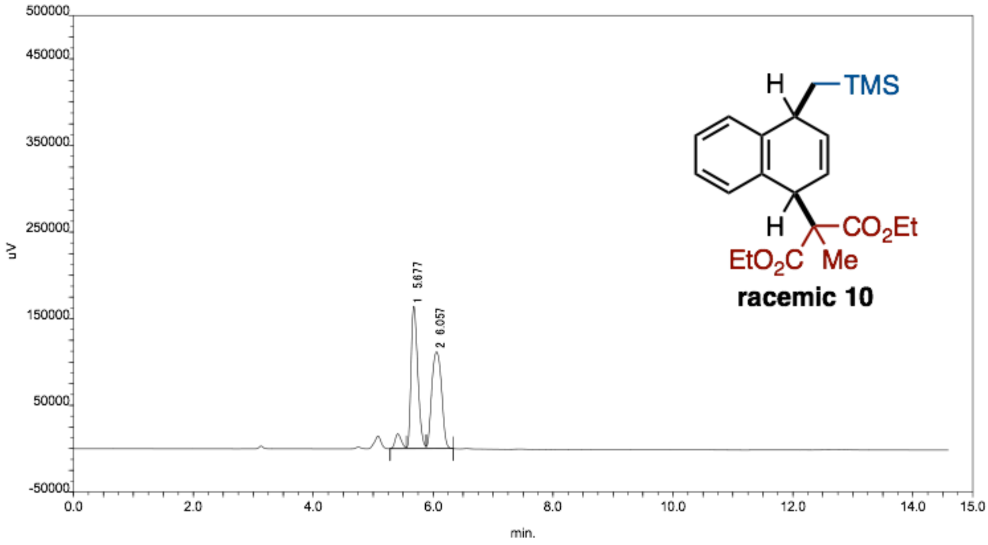

Result

No.	Rt (min)	Area	Area (%)	height
1	5.677	1278753.4	50.1	164276
2	6.057	1273451.0	49.9	111696
		2552204.4	100.0	275972

Chromatogram Report

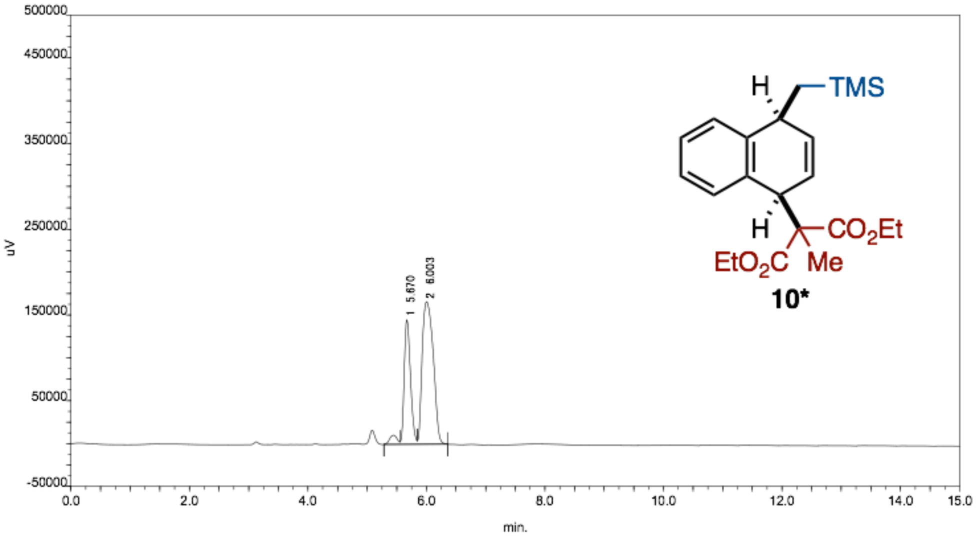

Result

No.	Rt (min)	Area	Area (%)	height
1	5.670	1075320.0	33.2	145238
2	6.003	2162662.3	66.8	166420
		3237982.3	100.0	311658

8. Effect of parameters

Effect of ligand

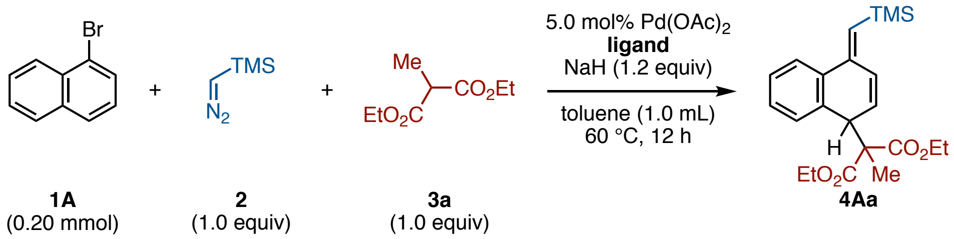

entry	**ligand (X mol%)**	recovery of **1A**[a] (%)	yield of **4Aa**[a] (%)
**1**	**L1 (20)**	**44**	**56**
2	PPh_3 (20)	51	44
3[b]	$Pd(PPh_3)_4$ (5)	52	39
4	**L2** (20)	46	2
5	**L3** (20)	56	44
6	**L4** (20)	45	15
7	**L5** (20)	96	0
8	**L6** (20)	100	0
9	**L7** (20)	10	1
10	**L8** (20)	84	15
11	DPEphos (10)	64	32
12	Xantphos (10)	100	0
13	XPhos (20)	94	6
14	(±) BINAP (10)	94	0
15	PPh_2Me (20)	72	0
16	PPh_2Et (20)	69	6
17	P^nBu_3 (20)	96	4
18	$P(OPh)_3$ (20)	92	0
19	$P(C_6F_5)_3$ (20)	76	0
20	none	93	0

[a] Recoveries and yields were determined by ^{1}H NMR using CH_2Br_2 as an internal standard.
[b] Without $Pd(OAc)_2$.

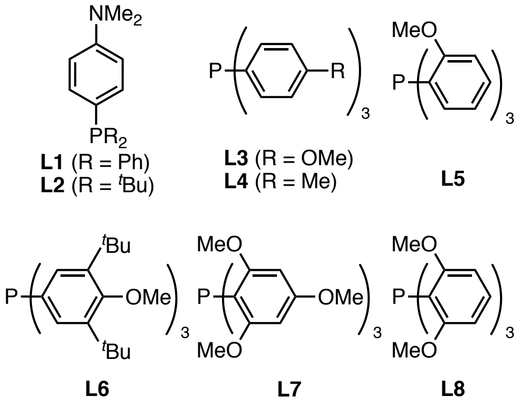

Effect of base

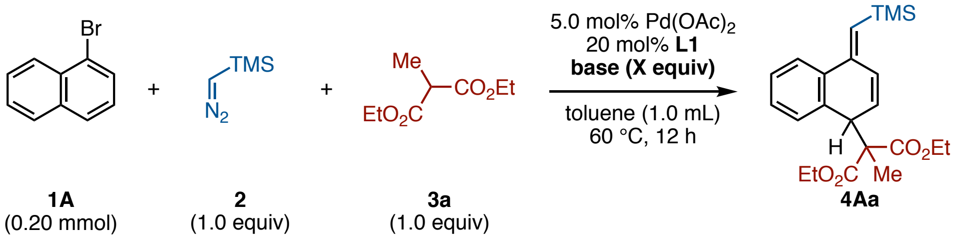

entry	**base**	**X**	recovery of **1A**[a] (%)	yield of **4Aa**[a] (%)
1	Cs_2CO_3	2.0	100	6
2	Na_2CO_3	2.0	95	0
3	NaO^tBu	2.0	87	0
4	LiO^tBu	2.0	100	3
5	K_3PO_4	2.0	98	0
6	NaH	1.2	44	56
**7**	**NaH**	**1.0**	**12**	**71**
8	NaH	2.0	15	20

[a] Recoveries and yields were determined by ^{1}H NMR using CH_2Br_2 as an internal standard.

Effect of additive

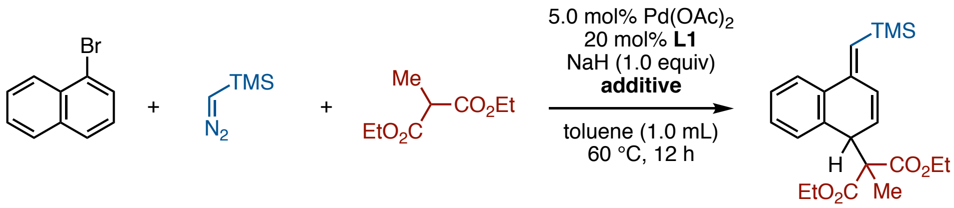

1A (0.20 mmol) **2** (1.0 equiv) **3a** (1.0 equiv) **4Aa**

entry	**additive (X equiv)**	recovery of **1A**[a] (%)	yield of **4Aa**[a] (%)
1	none	12	71
2	H_2O (1.0)	81	19
**3**	**3Å MS (50 mg)**	**12**	**88**
4	KF (2.0) with 3Å MS	64	36
5	KCl (2.0) with 3Å MS	69	31
6	KBr (2.0) with 3Å MS	41	48

[a] Recoveries and yields were determined by ^1H NMR using CH_2Br_2 as an internal standard.

Effect of Pd source

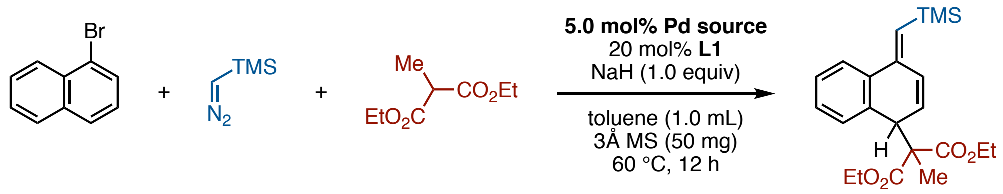

1A (0.20 mmol) **2** (1.0 equiv) **3a** (1.0 equiv) **4Aa**

entry	**Pd source**	recovery of **1A**[a] (%)	yield of **4Aa**[a] (%)
**1**	**$Pd(OAc)_2$**	**12**	**88**
2	$PdBr_2$	52	48
3	$Pd(COOCF_3)_2$	54	46
4	$Pd(cod)Cl_2$	52	48
5	$Pd(acac)_2$	65	28
6[b]	$Pd_2(allyl)_2Cl_2$	68	28
7[b]	$Pd_2(dba)_3$	69	25
8	$Pd(MeCN)_4(BF_4)_2$	100	0

[a] Recoveries and yields were determined by ^1H NMR using CH_2Br_2 as an internal standard.
[b] 2.5 mol% of Pd source

Effect of temperature

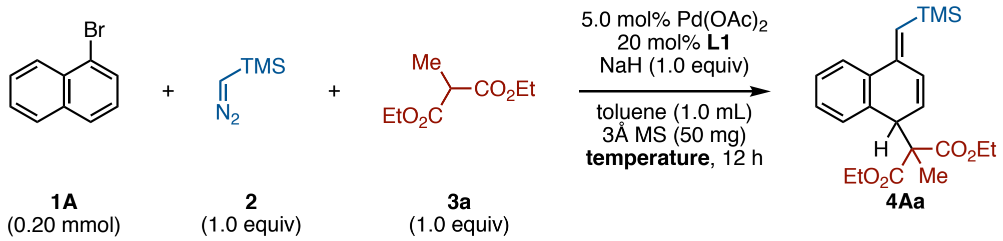

entry	**temperature** (°C)	recovery of **1A**[a] (%)	yield of **4Aa**[a] (%)
1	50	48	52
**2**	**60**	**12**	**88**
3	70	54	24
4	80	49	30

[a] Recoveries and yields were determined by ^{1}H NMR using CH_2Br_2 as an internal standard.

Effect of solvent

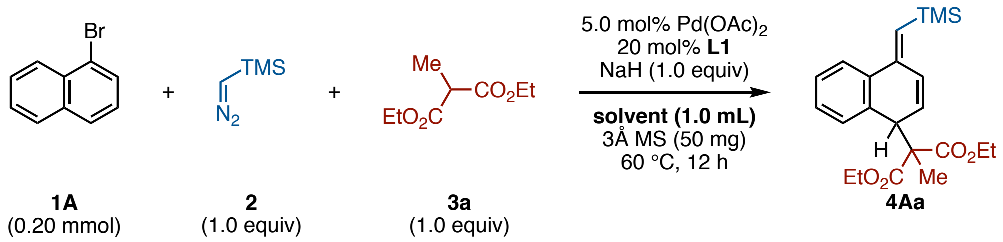

entry	**solvent**	recovery of **1A**[a] (%)	yield of **4Aa**[a] (%)
**1**	**toluene**	**12**	**88**
2	cyclohexane	32	54
3	1,4-dioxane	60	40
4	CH_2Cl_2	68	32
5	DCE	82	18
6	Et_2O	54	26
7	^{t}AmylOH	78	4
8	^{t}BuOH	91	0

[a] Recoveries and yields were determined by ^{1}H NMR using CH_2Br_2 as an internal standard.

Condition screening for dearomatization of bromobenzene (1F)

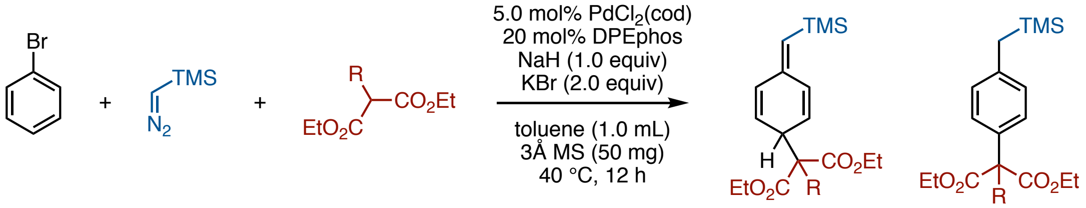

1F (0.20 mmol) **2** (1.0 equiv) **3a** (R = Me) or **3e** (R = H) (1.0 equiv) **4Fa** or **4Fe** **4Fa'** or **4Fe'**

entry	malonate	**variation from above conditions**	yield of **4Fa** or **4Fe** (%)[a]	yield of **4Fa'** or **4Fe'** (%)[a]
1	**3e**	$Pd(OAc)_2$ and **L1** were used w/o KBr at 60 °C	0	0
2	**3e**	$Pd(OAc)_2$ was used w/o KBr at 60 °C	6	0
3	**3e**	$Pd_2(allyl)_2Cl_2$ was used w/o KBr at 60 °C	8	0
4	**3e**	w/o KBr at 70 °C	10	0
5	**3e**	at 70 °C	17	0
6	**3a**	at 70 °C	37	21
7	**3a**	cyclohexane at 70 °C	32	37
8	**3a**	cyclohexane at 70 °C, 6 h	45	25
9	**3a**	at 50 °C	54	18
10	**3a**	6 h	57	12
**11**	**3a**	**none**	**65**	**26**

[a] Yields were determined by ^{1}H NMR using CH_2Br_2 as an internal standard.

Solvent effect for dearomatization using benzaldehyde *p*-toluenesulfonylhydrazone (7a)

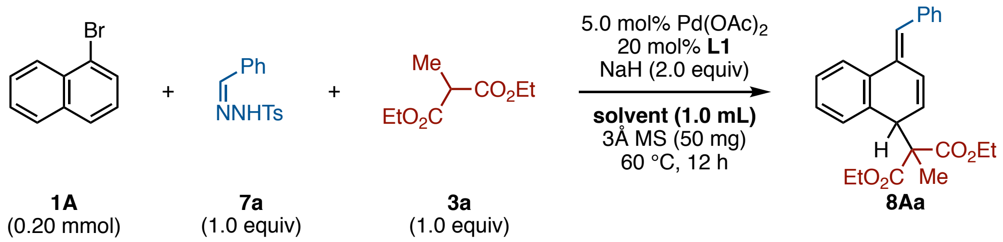

entry	**solvent**	recovery of **1A**[a] (%)	yield of **8Aa**[a] (%)
1	toluene	69	13
2	THF	46	1
3	1,4-dioxane	40	20
4	DME	31	3
5	CH_2Cl_2	48	8
6	DCE	73	1
7	*m*-xylene	75	8
8	benzene	45	12
9	$PhCF_3$	40	2
10	**1,4-dioxane**	**38**	**31**
11	Et_2O	54	9
12	$CHCl_3$	97	0

[a] Recoveries and yields were determined by ^{1}H NMR using CH_2Br_2 as an internal standard.

Base effect for dearomatization using benzaldehyde *p*-toluenesulfonylhydrazone (7a)

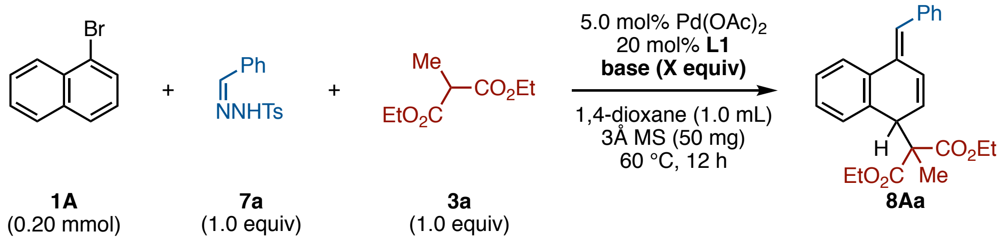

entry	**base**	**X**	recovery of **1A**[a] (%)	yield of **8Aa**[a] (%)
**1**	**NaH**	**2.0**	**38**	**31**
2	K_2CO_3	2.0	100	0
3	NaO*ᵗ*Bu	2.0	65	7
4	KO*ᵗ*Bu	2.0	52	6
5	LiO*ᵗ*Bu	2.0	77	0
6	NaH	2.5	22	17
7	NaH	3.0	13	11

[a] Recoveries and yields were determined by ^{1}H NMR using CH_2Br_2 as an internal standard.

Equivalent effect for dearomatization using benzaldehyde *p*-toluenesulfonylhydrazone (7a)

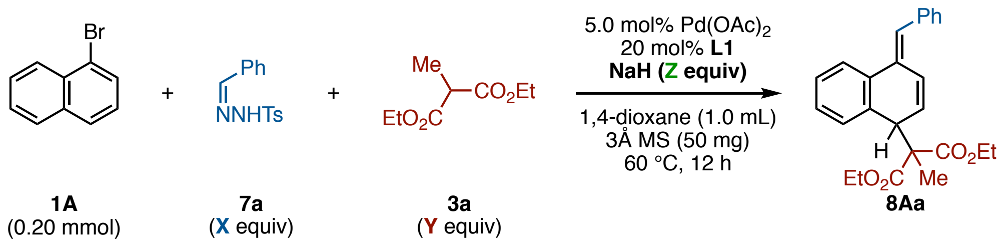

entry	X	Y	Z	recovery of **1A**[a] (%)	yield of **8Aa**[a] (%)
1	1.0	1.0	2.0	38	31
2	1.5	1.0	2.0	54	18
3	2.0	1.0	2.0	87	0
4	1.0	2.0	3.0	31	32
**5**	**1.5**	**1.5**	**3.0**	**22**	**41**
6	2.0	2.0	4.0	0	38

[a] Recoveries and yields were determined by ^{1}H NMR using CH_2Br_2 as an internal standard.

Temperature and reaction time effects for dearomatization using benzaldehyde *p*-toluenesulfonylhydrazone (7a)

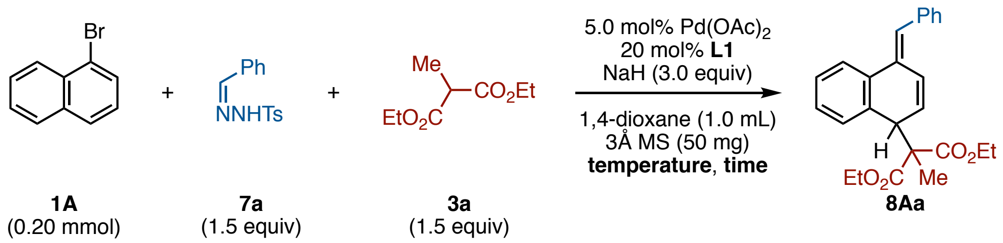

entry	**temperature**	**time**	recovery of **1A**[a] (%)	yield of **8Aa**[a] (%)
1	60 °C	12 h	22	41
2	40 °C	24 h	63	11
3	50 °C	24 h	21	35
**4**	**50 °C**	**36 h**	**16**	**43**

[a] Recoveries and yields were determined by ^{1}H NMR using CH_2Br_2 as an internal standard.

Limitation (Ar–Br)

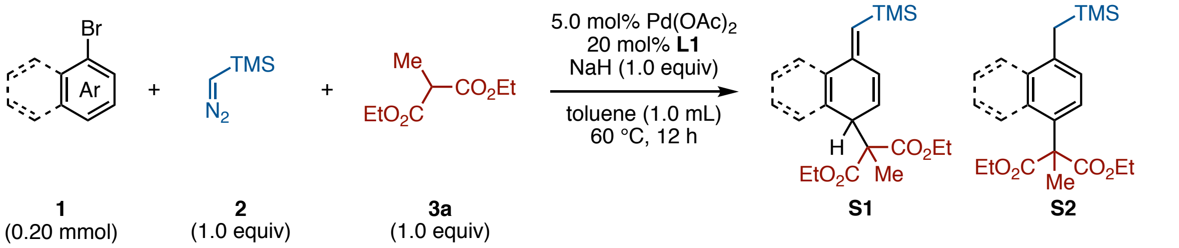

^{1}H NMR yield[a]

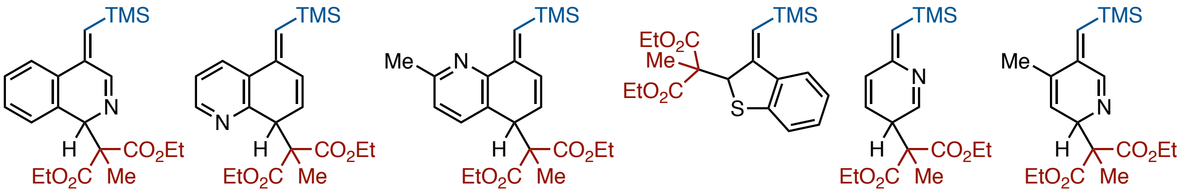

**S1a** : 19%	**S1b** : 8%	**S1c** : 0%	**S1d** : 0%	**S1e**[b] : 0%	**S1f**[b] : 0%
**S2a'** : 59%	**S2b'** : 74%	**S2c'** : 0%	**S2d'** : 0%	**S2e'** : 0%	**S2f'** : 68%

[a] Yields were determined by ^{1}H NMR using CH_2Br_2 as an internal standard.
[b] $Pd(cod)Cl_2$ (5.0 mol %), DPEphos (10 mol %) were used as catalysts and KBr (2.0 equiv) was added at 40 °C.

Limitation (nucleophile)

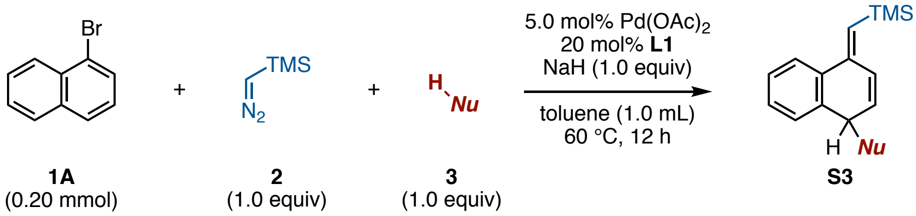

^{1}H NMR yield[a]

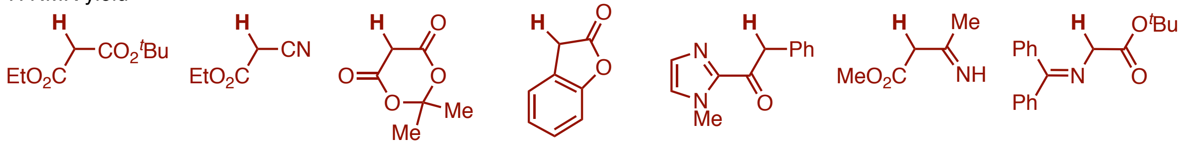

**S3a** : 46%<br>57:43 dr	**S3b** : 0%	**S3c** : 0%	**S3d**: 0%	**S3e** : 0%	**S3f** : 0%	**S3g** : 0%

[a] Yields were determined by ^{1}H NMR using CH_2Br_2 as an internal standard.

Limitation (diazo compound)

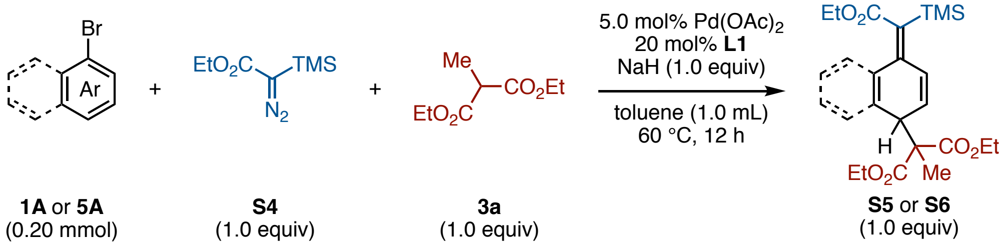

^{1}H NMR yield[a]

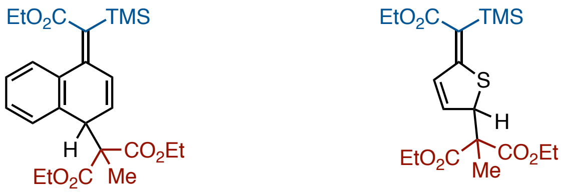

**S5** : 0%	**S6** : 0%

[a] Yields were determined by ^{1}H NMR using CH_2Br_2 as an internal standard.

9. References

[1] Ni, S.; Remaily, M. A. E. A. A. A. E.; Franzén, J. Carbocation Catalyzed Bromination of Alkyl Arenes, a Chemoselective *sp*^{3} *vs. sp*^{2} C–H Functionalization. *Adv. Synth. Catal.* **2018**, *360*, 4197–4204.

[2] Whelligan, D. K.; Solanki, S.; Taylor, D.; Thomson, D. W.; Cheung, K. M.; Boxall, K.; Mas-Droux, C.; Barillari, C.; Burns, S.; Grummitt, C. G.; Collins, I.; van Montfort, R. L.; Aherne, G. W.; Bayliss, R.; Hoelder, S. Aminopyrazine Inhibitors Binding to an Unusual Inactive Conformation of the Mitotic Kinase Nek2: SAR and Structural Characterization. *J. Med. Chem.* **2010**, *53*, 7682–7698.

[3] Gothard, C. M.; Soh, S.; Gothard, N. A.; Kowalczyk, B.; Wei, Y.; Baytekin, B.; Grzybowski, B. A. Rewiring Chemistry: Algorithmic Discovery and Experimental Validation of One-Pot Reactions in the Network of Organic Chemistry. *Angew. Chem., Int. Ed.* **2012**, *51*, 7922–7927.

[4] Colletto, C.; Islam, S.; Juliá-Hernández, F.; Larrosa, I. Room-Temperature Direct β-Arylation of Thiophenes and Benzo[*b*]-Thiophenes and Kinetic Evidence for a Heck-Type Pathway. *J. Am. Chem. Soc*. **2016**, *138*, 1677–1683.

[5] Funaki, K.; Sato, T.; Oi, S. Pd-Catalyzed β-Selective Direct C–H Bond Arylation of Thiophenes with Aryltrimethylsilanes. *Org. Lett*. **2012**, *14*, 6186–6189.

[6] Amb, C. M.; Rasmussen, S. C. Sterics versus Electronics: Regioselective Cross-Coupling of Polybrominated Thiophenes. *Eur. J. Org. Chem.* **2008**, *2008*, 801–804.

[7] Komatsuda, M.; Kato, H.; Muto, K.; Yamaguchi, J. Pd-Catalyzed Dearomative Three-Component Reaction of Bromoarenes with Diazo Compounds and Allylborates. *ACS Catal*. **2019**, *9*, 8991–8995.

[8] Wilent, J.; Petersen, K. S. Enantioselective Desymmetrization of Diesters. *J. Org. Chem*. **2014**, *79*, 2303–2307.

[9] Bosque, I.; Bach, T. 3-Acetoxyquinuclidine as Catalyst in Electron Donor-Acceptor Complex-Mediated Reactions Triggered by Visible Light. *ACS Catal.* **2019**, *9*, 9103–9109.

[10] Bzeih, T.; Zhang, K.; Khalaf, A.; Hachem, A.; Alami, M.; Hamze, A. One-Pot Reaction Between *N*-Tosylhydrazones and 2-Nitrobenzyl Bromide: Route to NH-Free C2-Arylindoles. *J. Org. Chem*. **2019**, *84*, 228–238.

[11] Hashikawa, Y.; Murata, M.; Wakamiya, A.; Murata, Y. Water Entrapped inside Fullerene Cages: A Potential Probe for Evaluation of Bond Polarization. *Angew. Chem., Int. Ed.* **2016**, *55*, 13109–13113.

[12] Peng, B.; Zhang, S.; Yu, X.; Feng, X.; Bao, M. Nucleophilic Dearomatization of Chloromethyl Naphthalene Derivatives via η^3-Benzylpalladium Intermediates: A New Strategy for Catalytic Dearomatization. *Org. Lett*. **2011**, *13*, 5402–5405.

10. ^{1}H and ^{13}C NMR Spectra

^{1}H NMR of **4Aa** (400 MHz, $CDCl_3$)

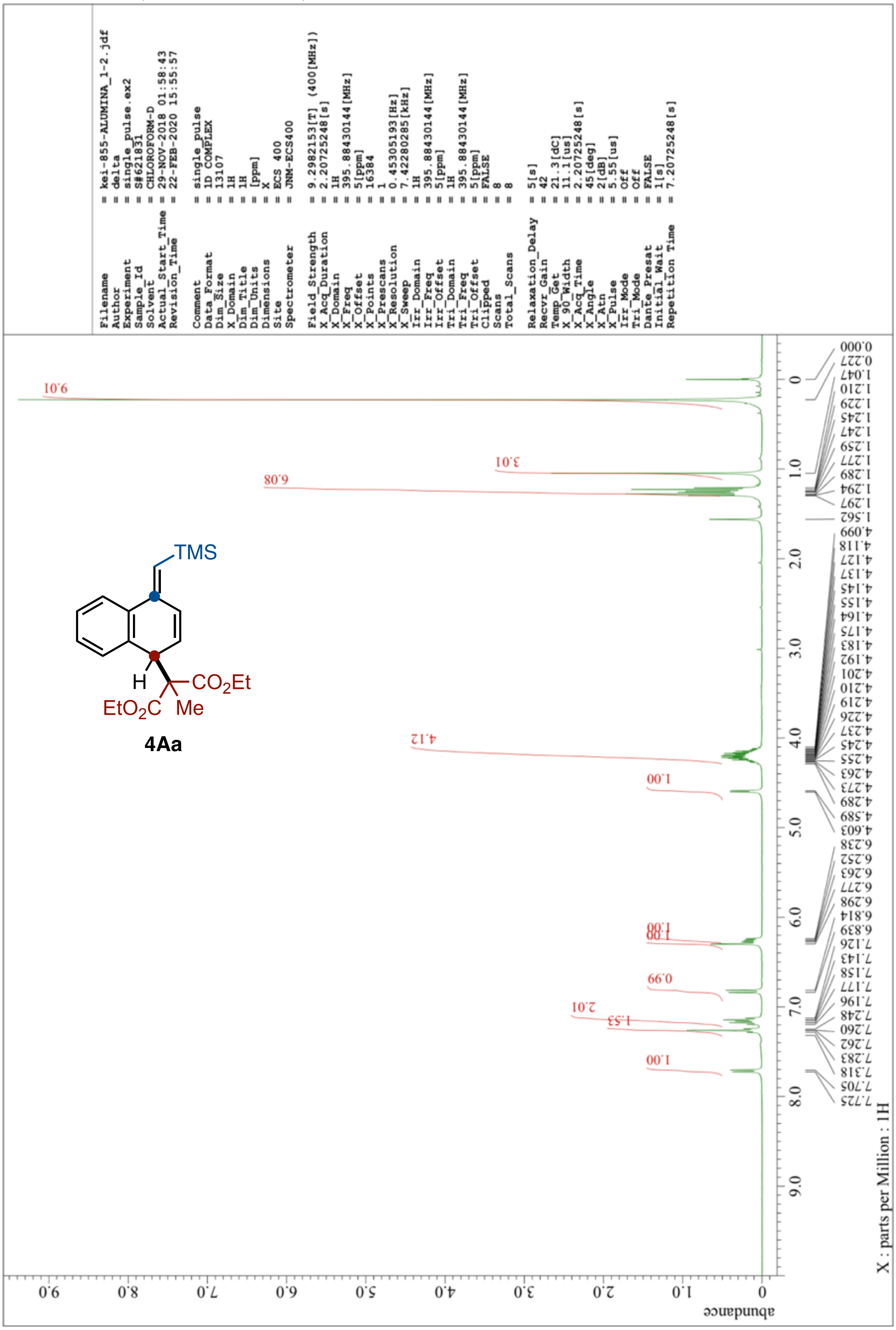

^{13}C NMR of **4Aa** (101 MHz, $CDCl_3$)

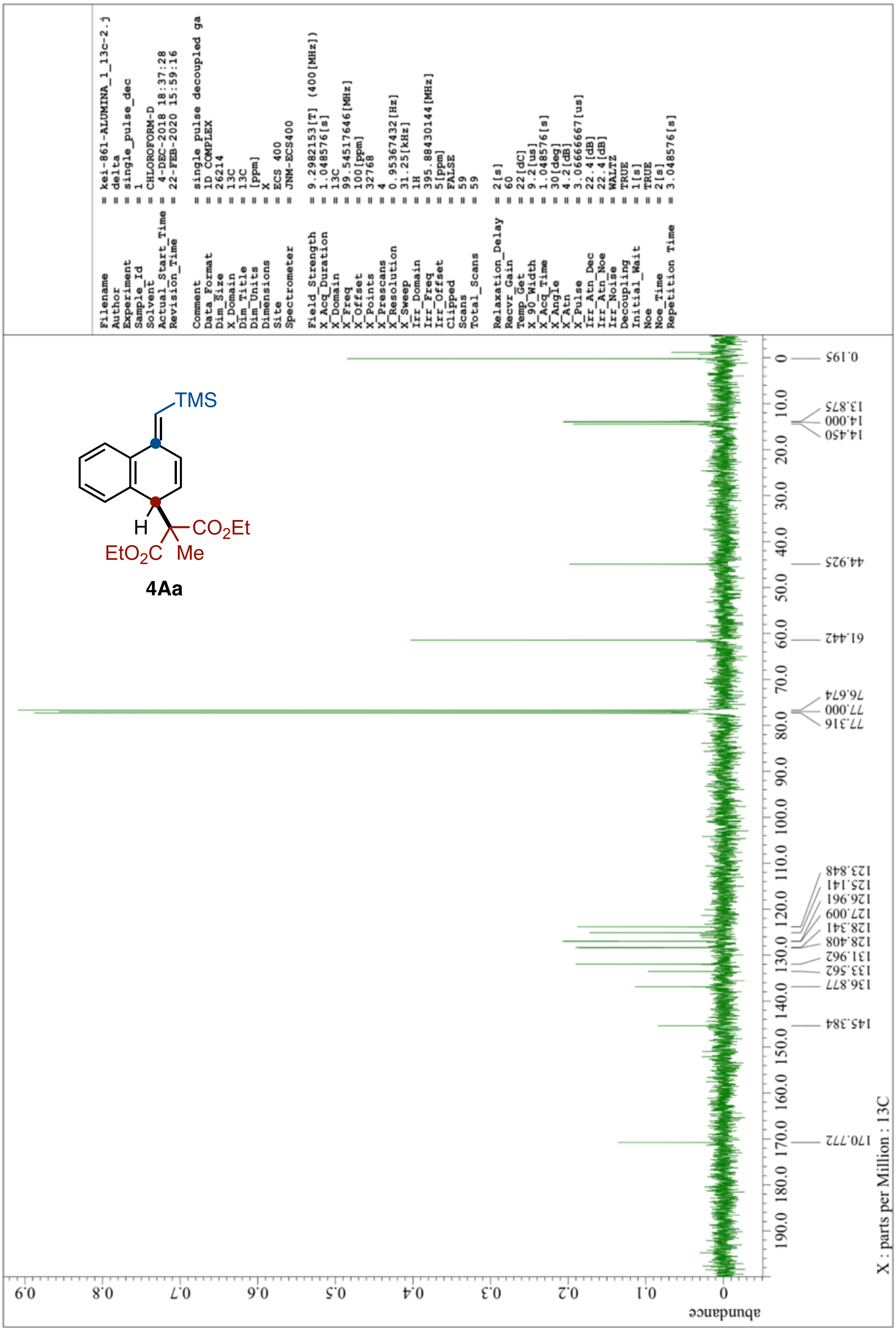

^{1}H NMR of **4Ab** (400 MHz, $CDCl_3$)

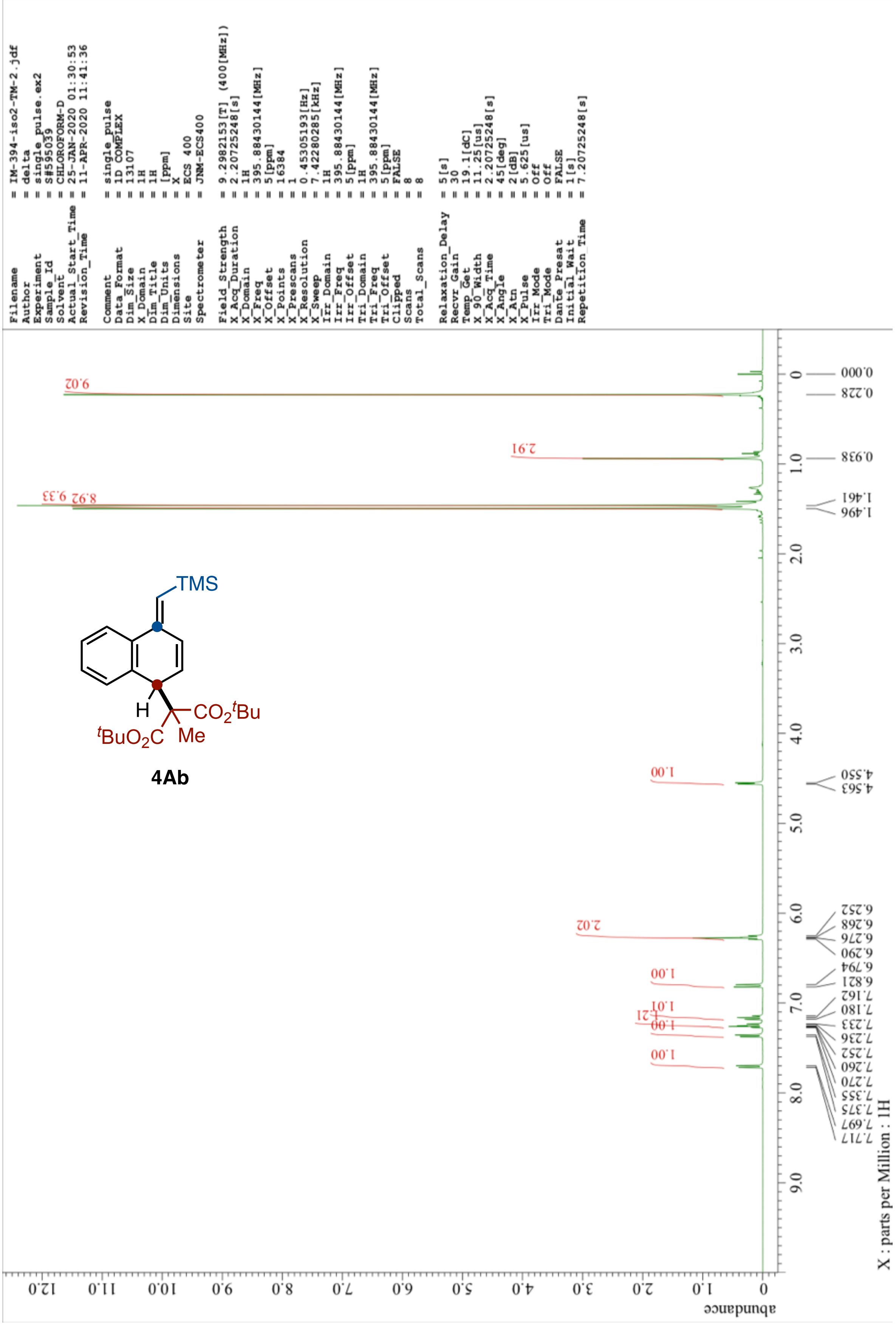

^{13}C NMR of **4Ab** (101 MHz, $CDCl_3$)

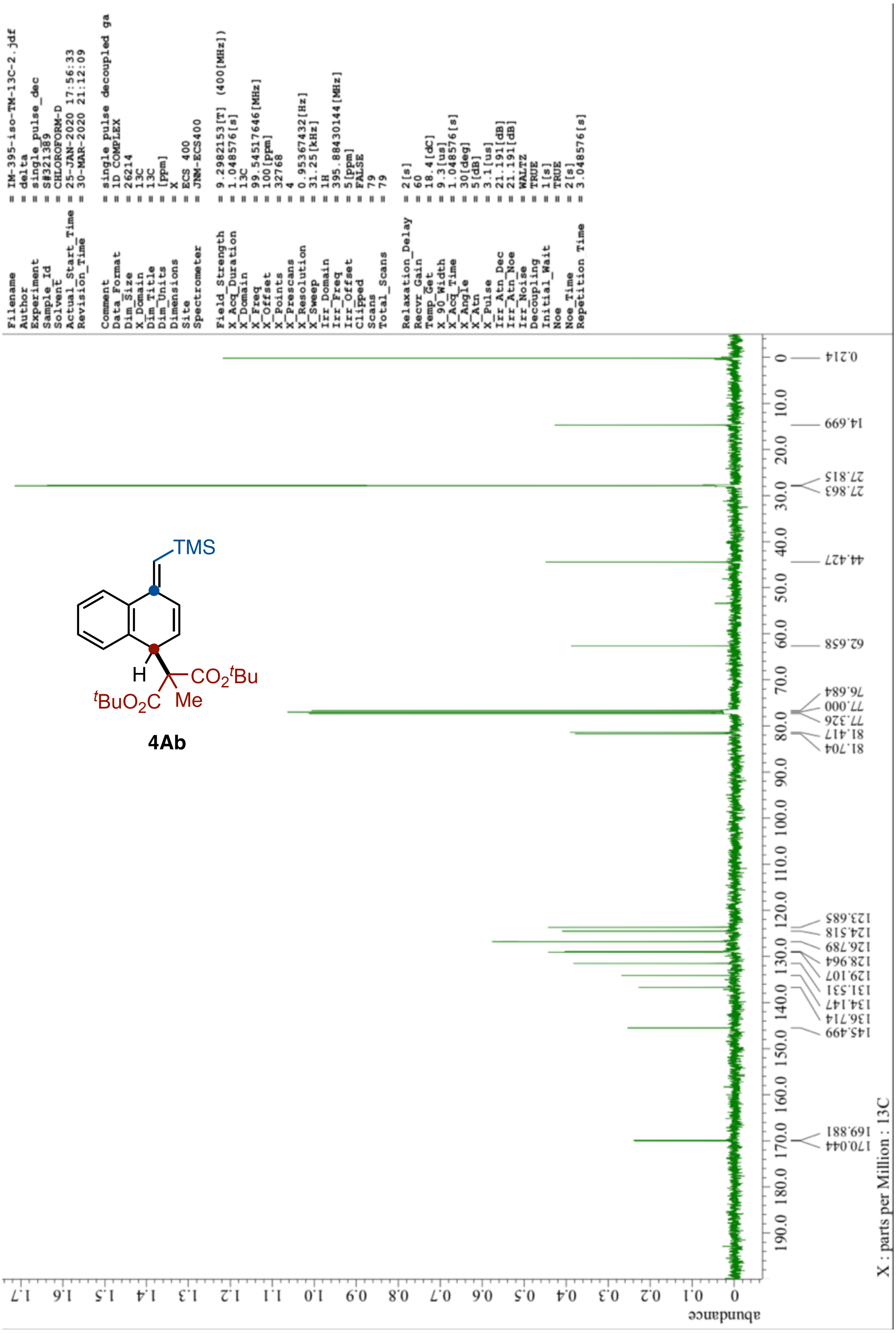

^{1}H NMR of **4Ba** (400 MHz, $CDCl_3$)

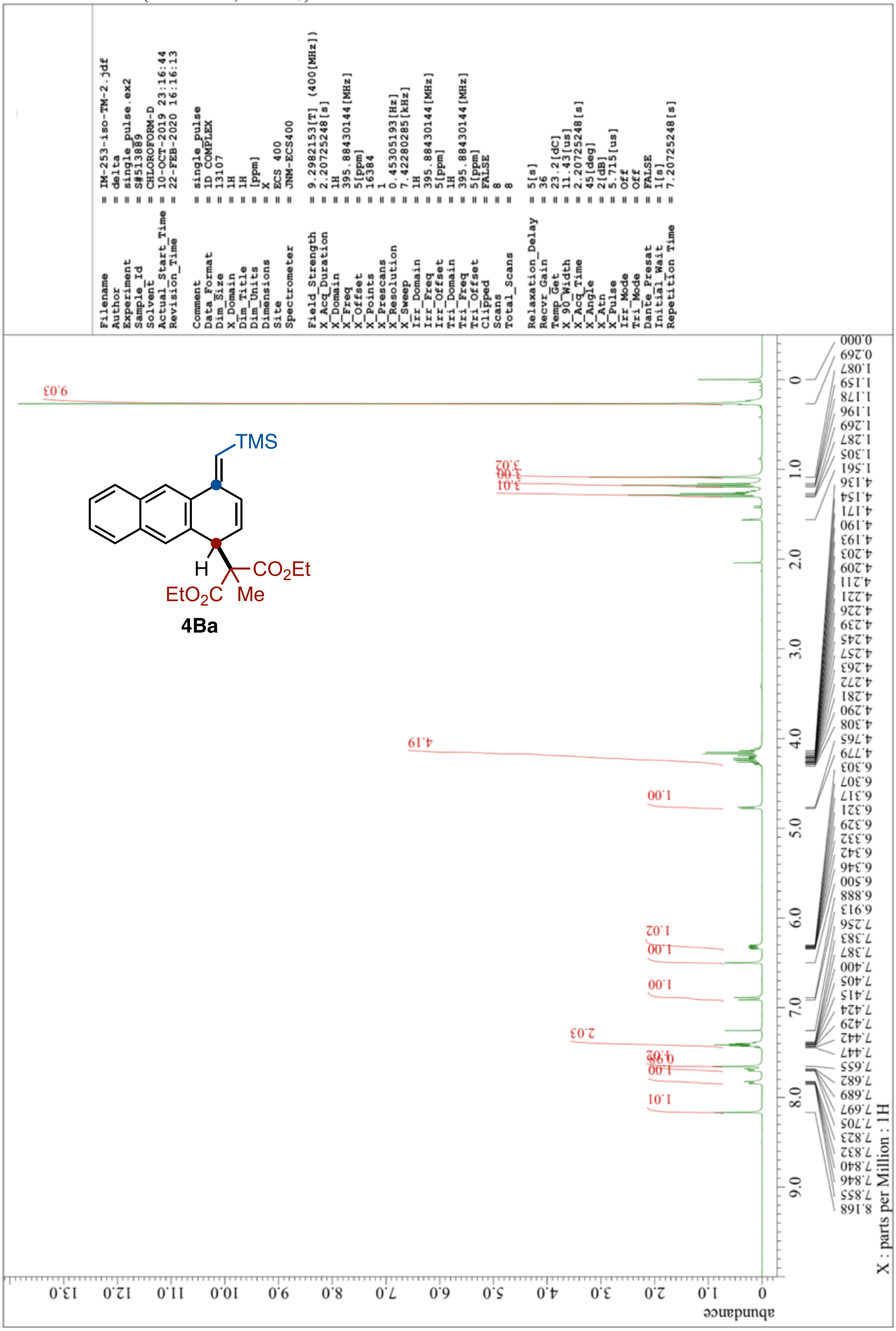

^{13}C NMR of **4Ba** (101 MHz, $CDCl_3$)

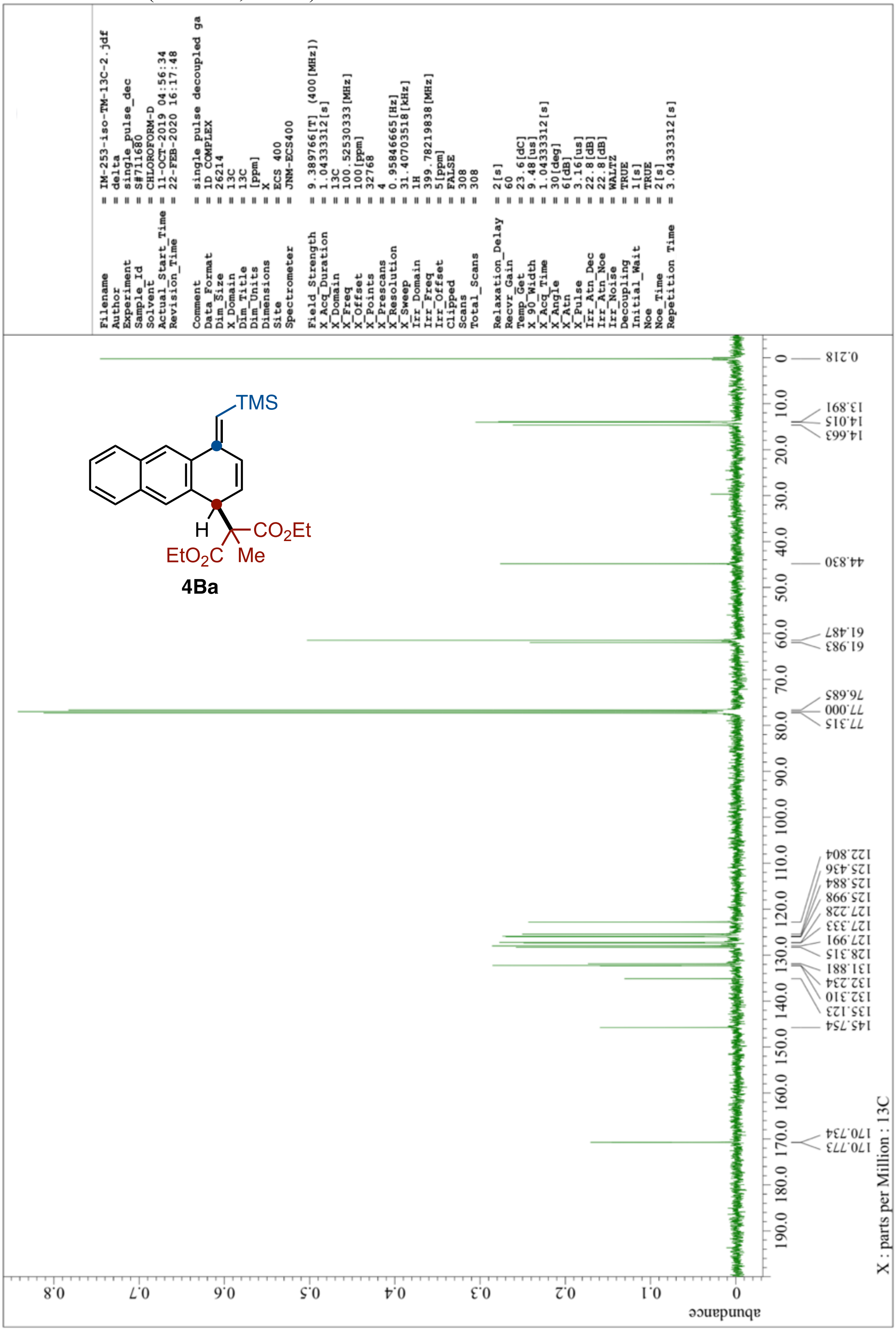

^1H NMR of **4Ca** (400 MHz, $CDCl_3$)

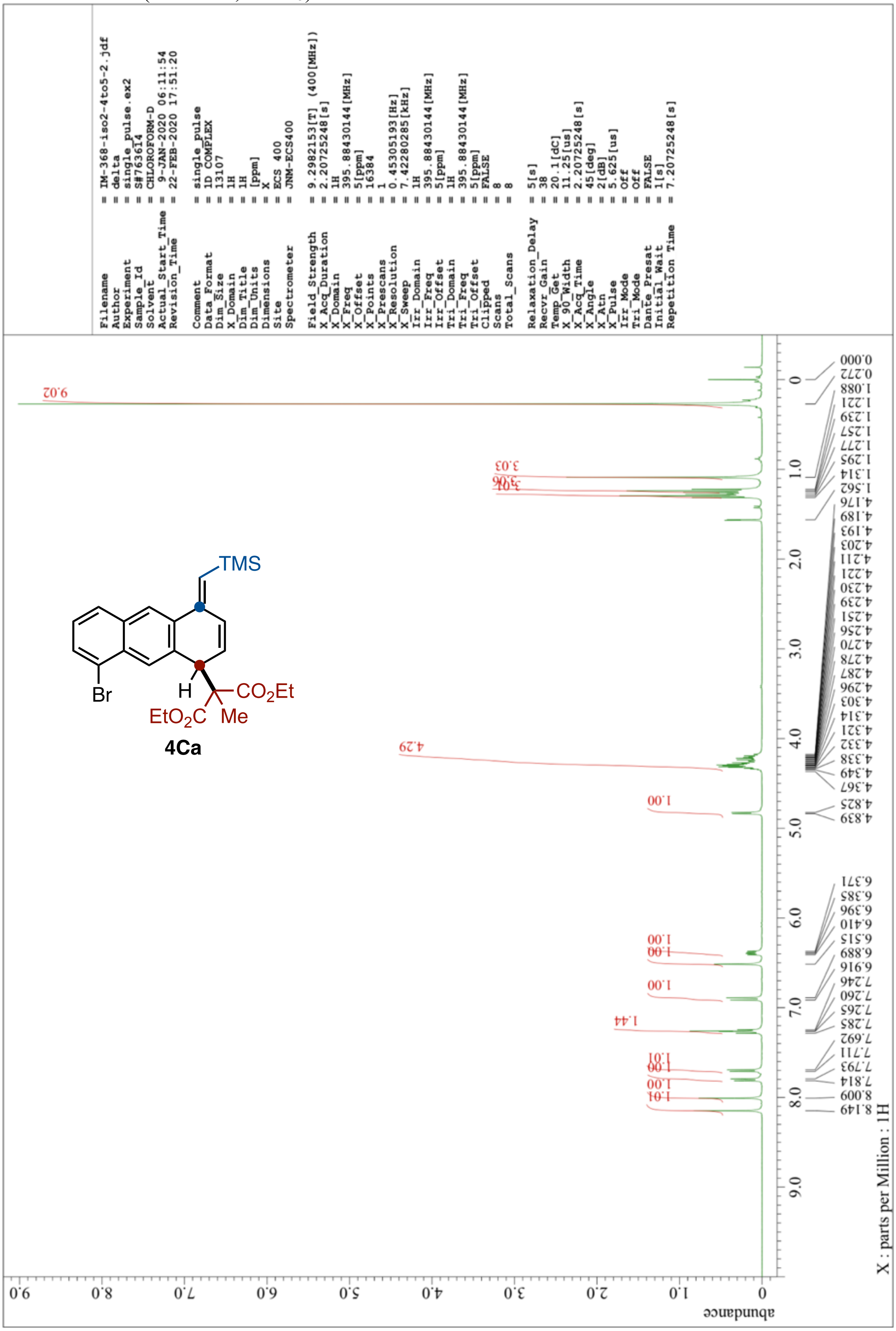

^{13}C NMR of **4Ca** (101 MHz, $CDCl_3$)

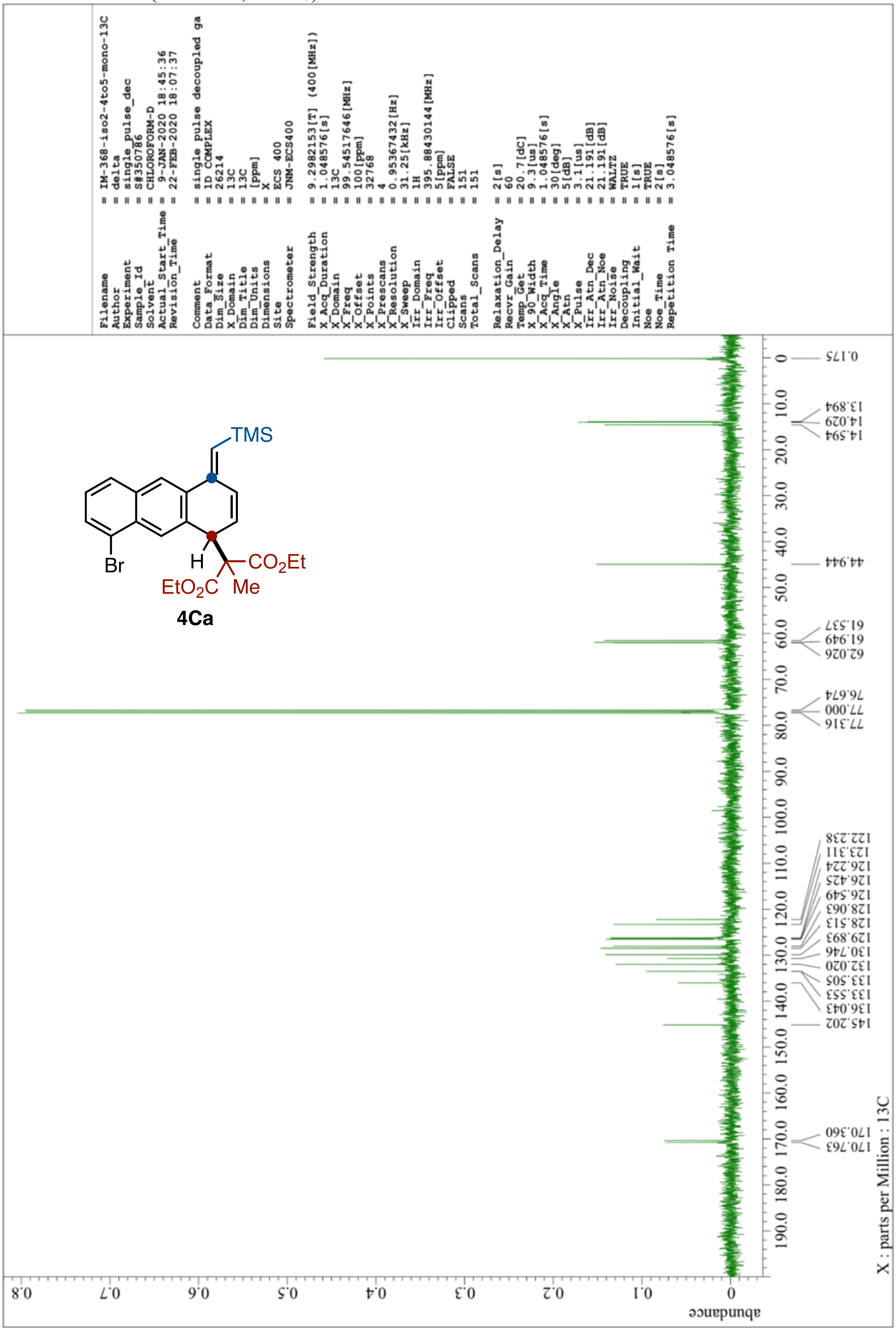

^{1}H NMR of **4Caa** (400 MHz, $CDCl_3$)

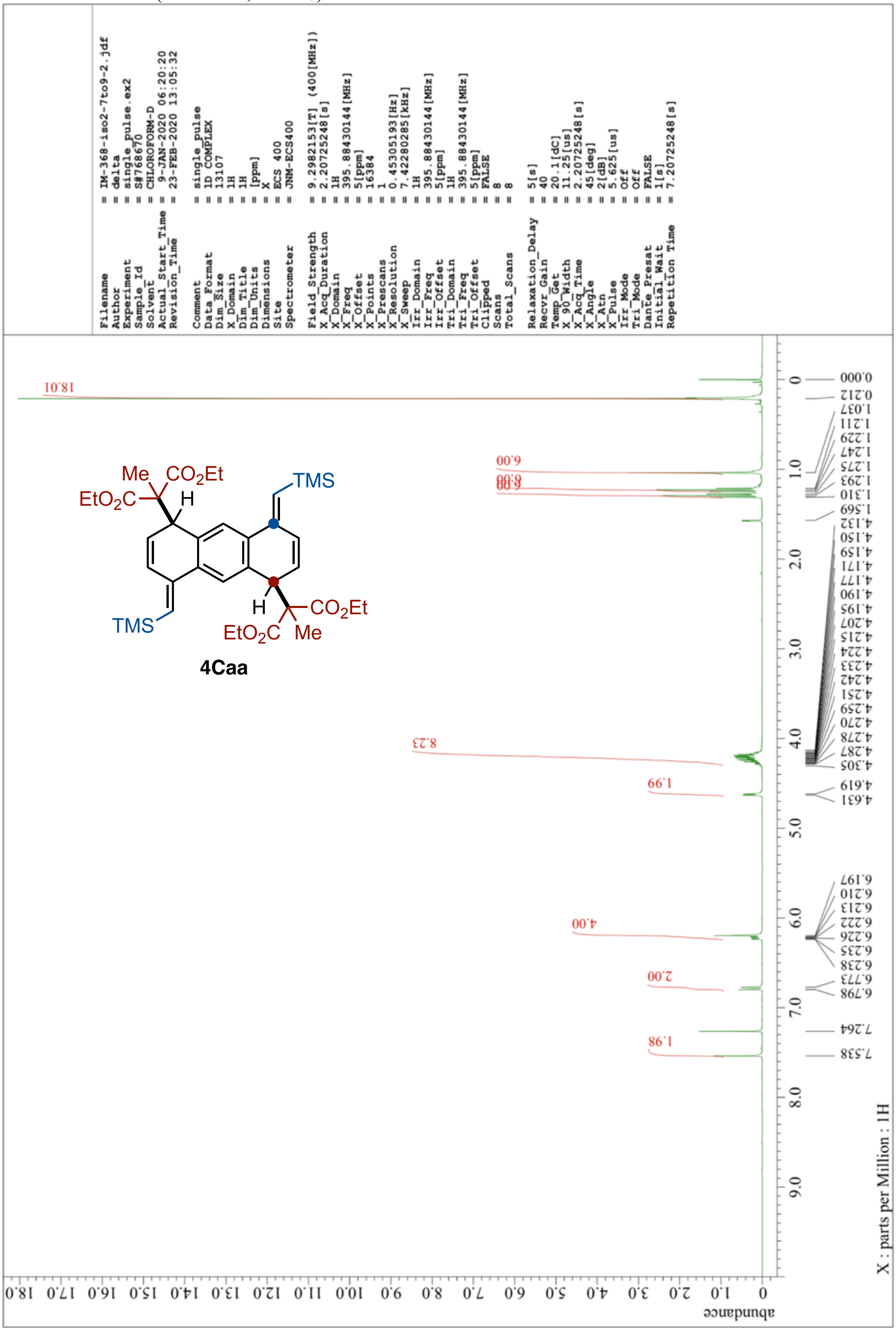

^{13}C NMR of **4Caa** (101 MHz, $CDCl_3$)

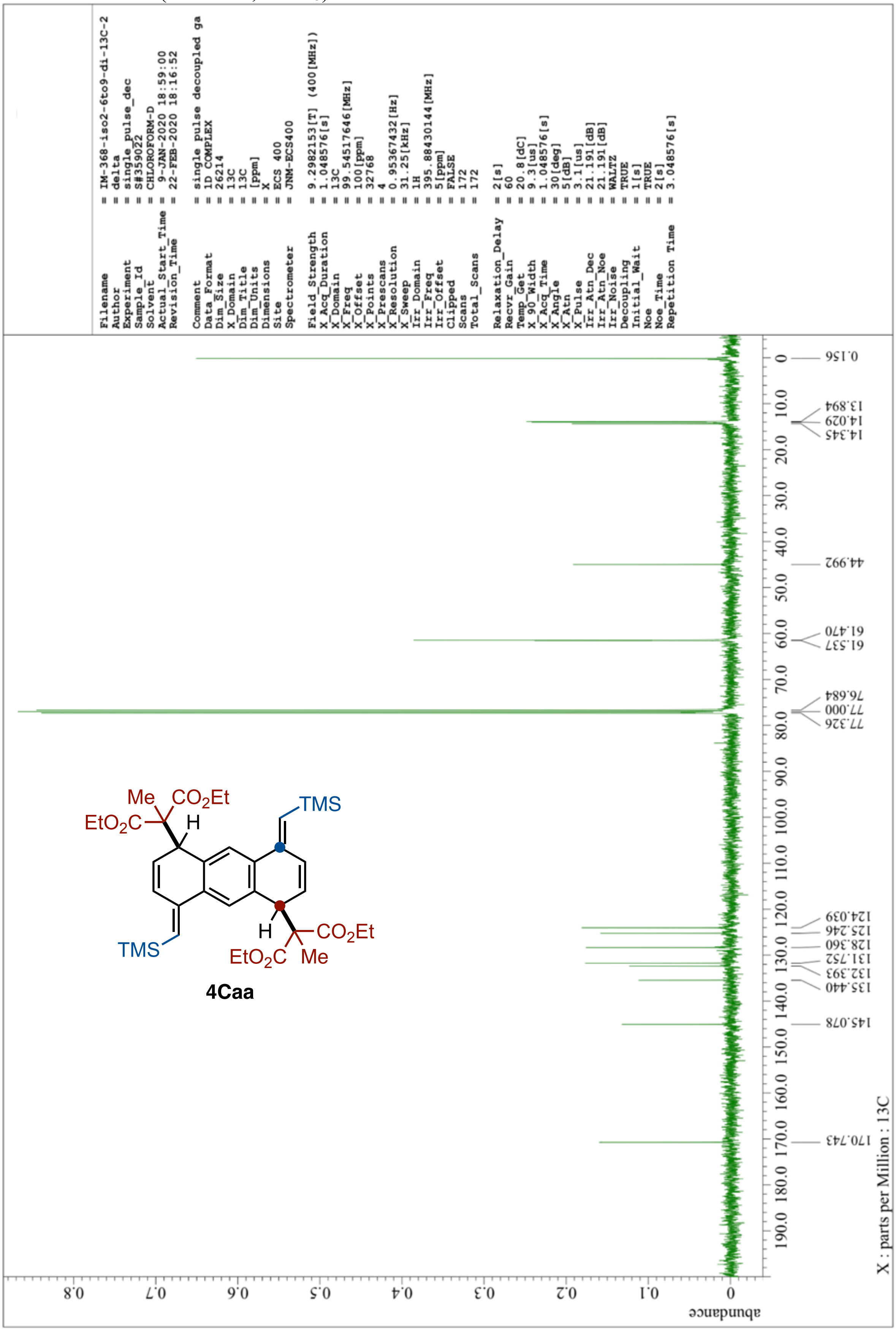

^1H NMR of **4Da** (400 MHz, $CDCl_3$)

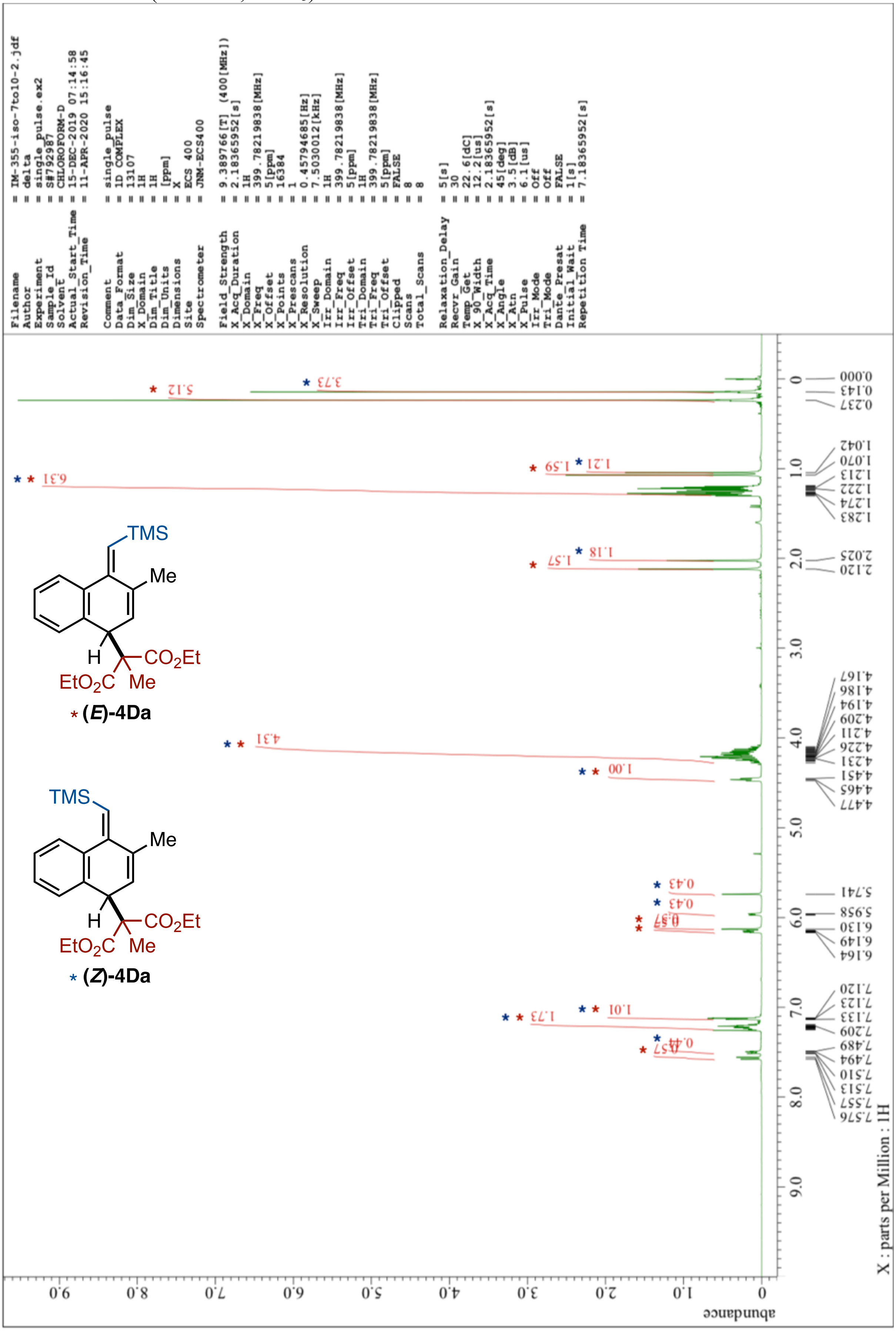

^{13}C NMR of **4Da** (101 MHz, $CDCl_3$)

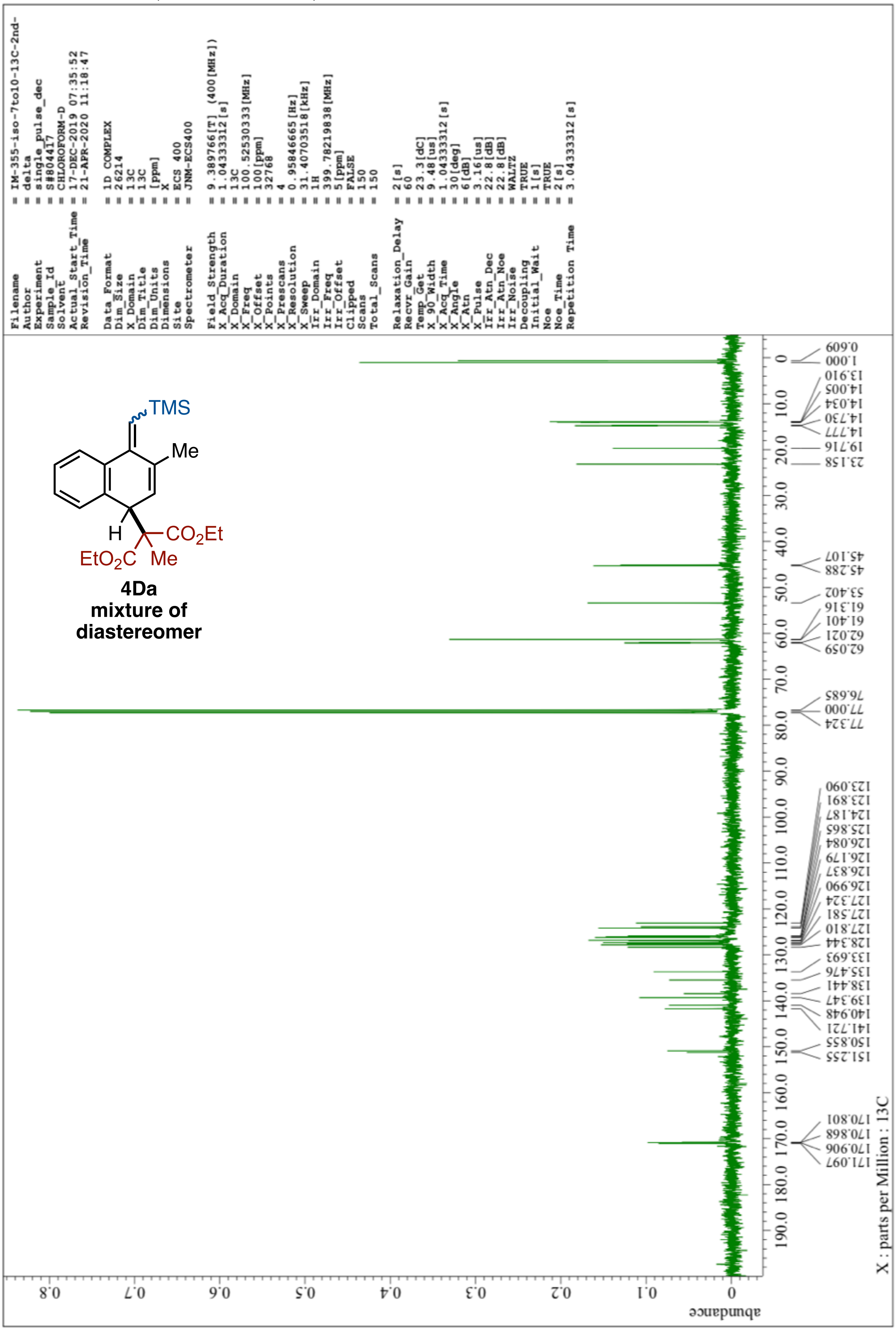

^{1}H NMR of **4Ea** (400 MHz, $CDCl_3$)

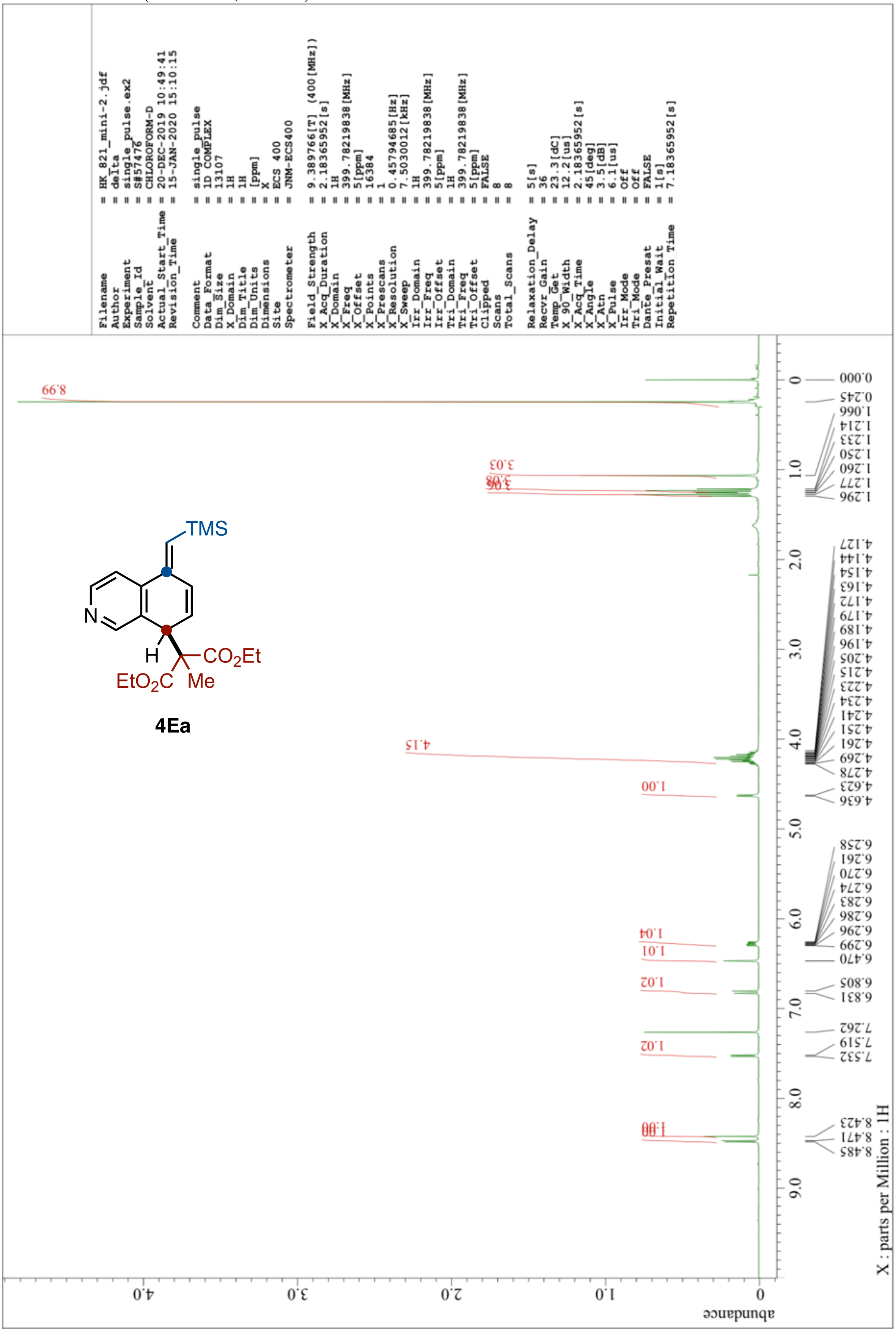

^{13}C NMR of **4Ea** (101 MHz, $CDCl_3$)

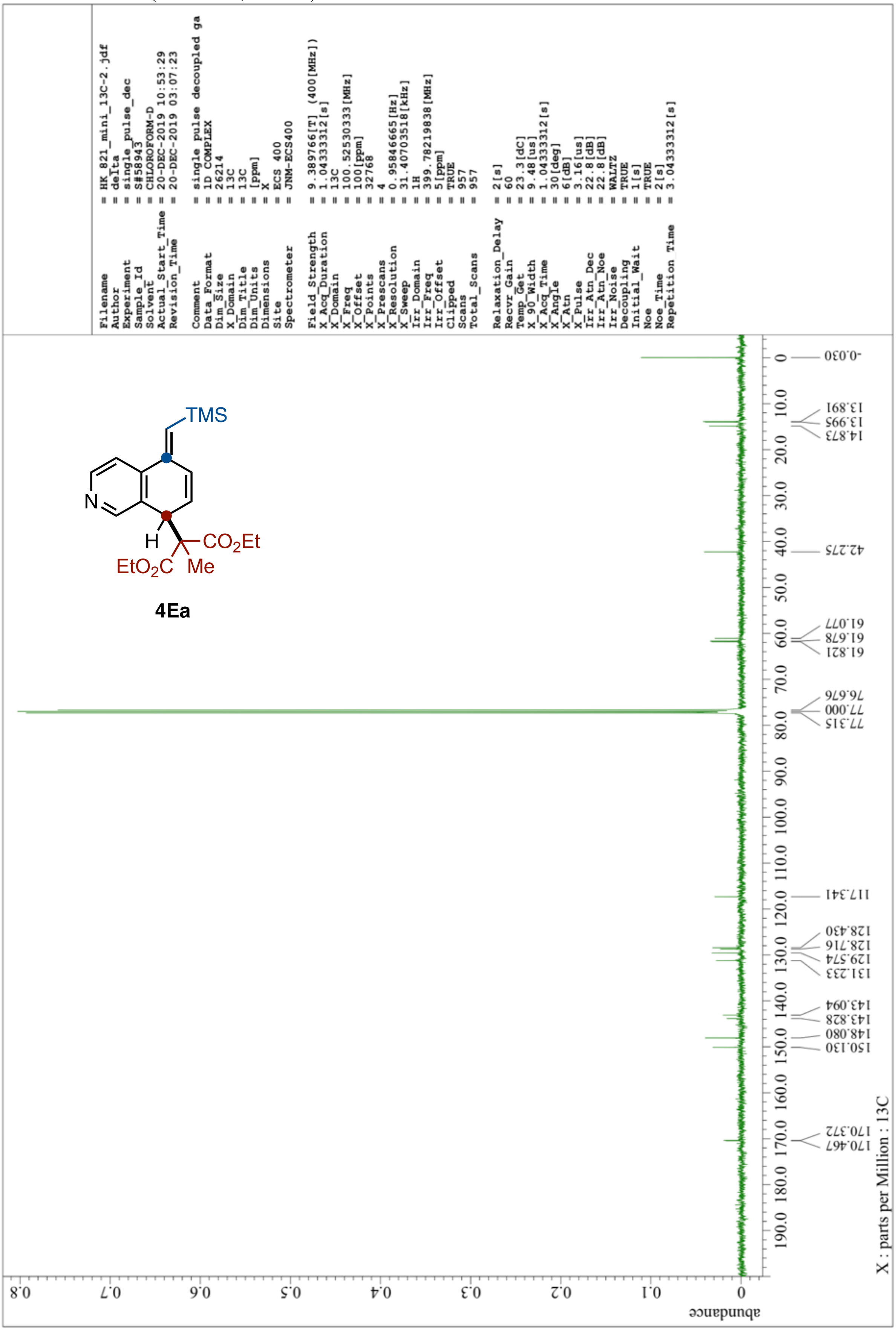

^{1}H NMR of **4Fa** (400 MHz, $CDCl_3$)

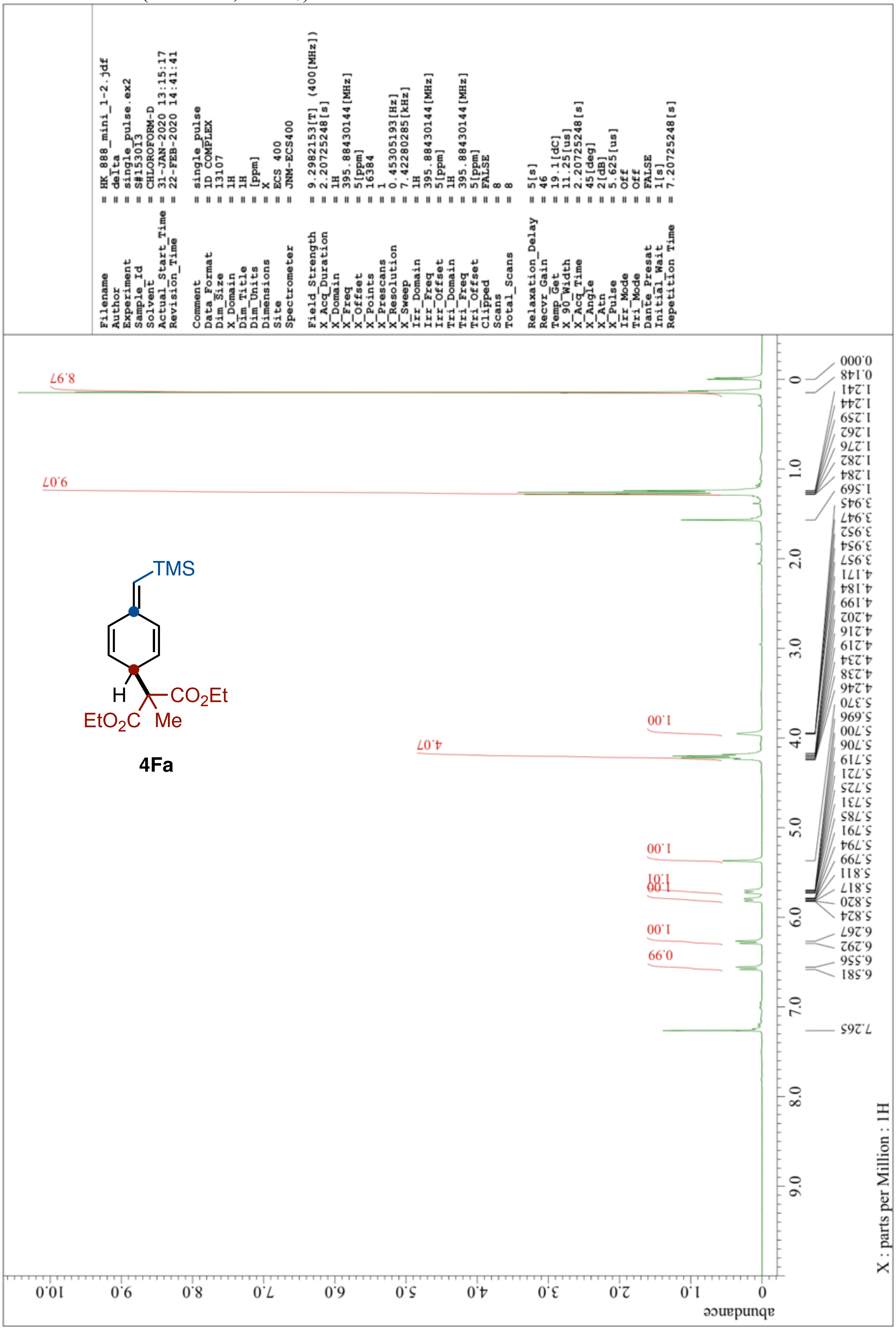

^{13}C NMR of **4Fa** (101 MHz, $CDCl_3$)

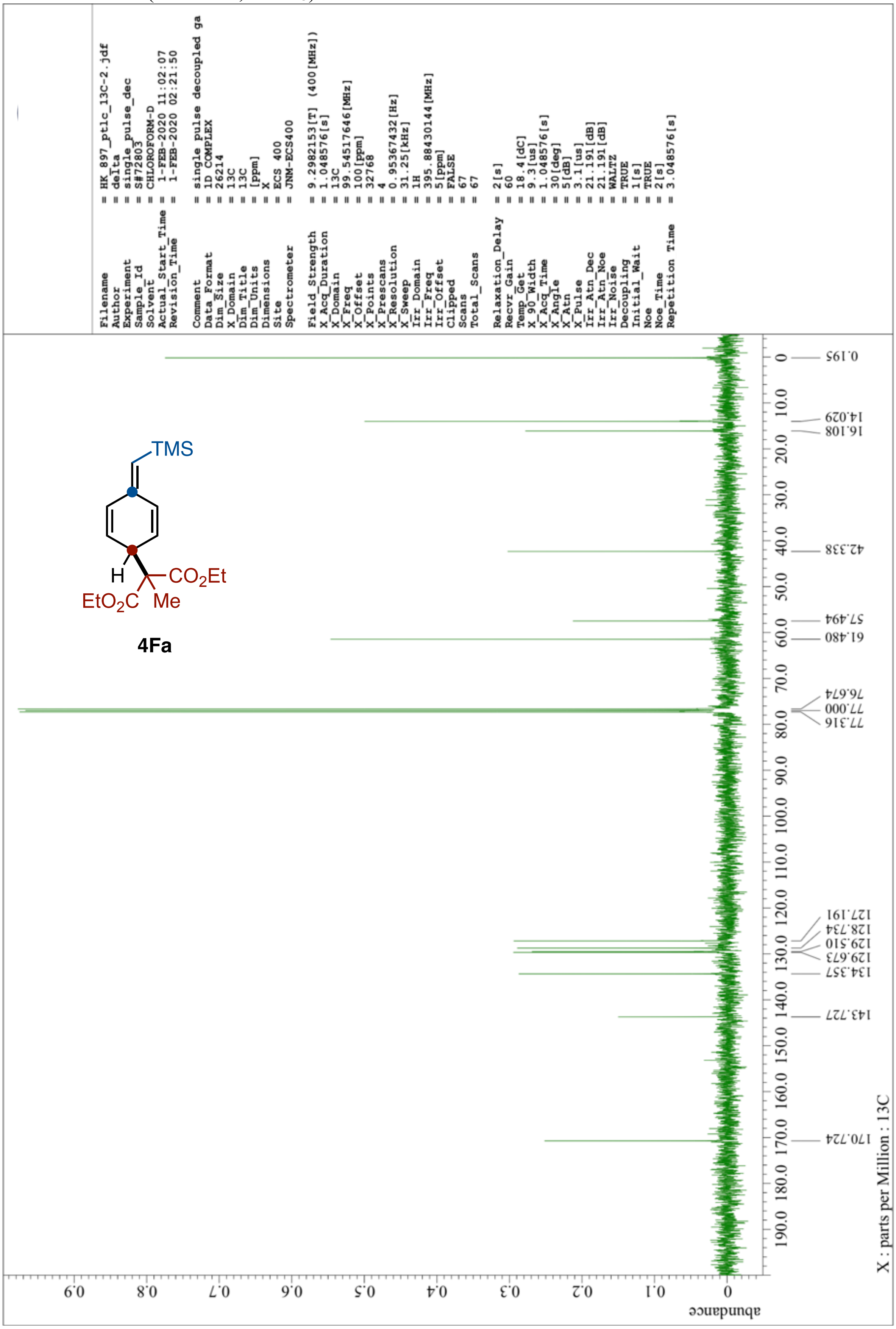

^{1}H NMR of **4Ga** (400 MHz, $CDCl_3$)

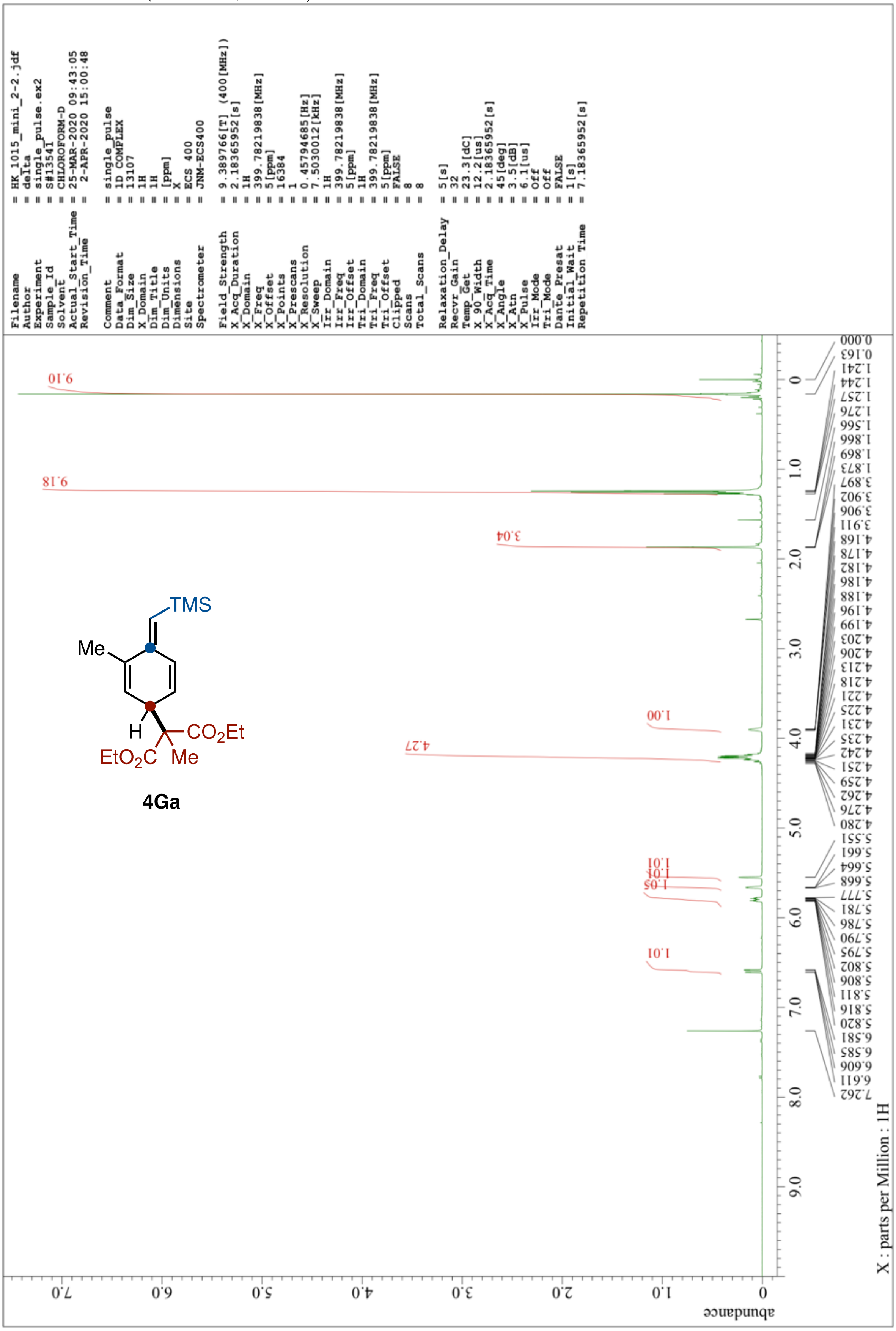

^{13}C NMR of **4Ga** (101 MHz, $CDCl_3$)

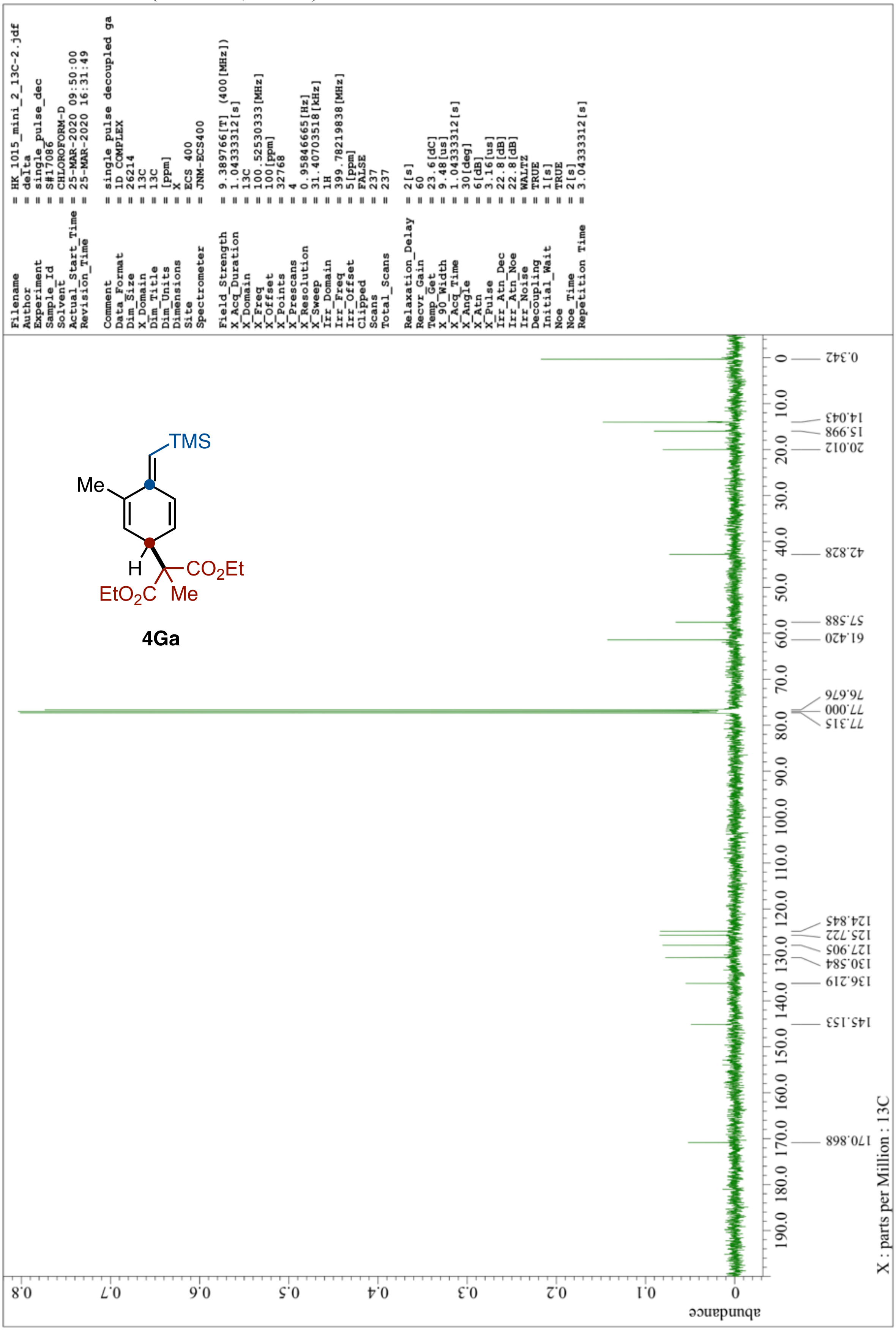

^{1}H NMR of **4Ac** (400 MHz, $CDCl_3$)

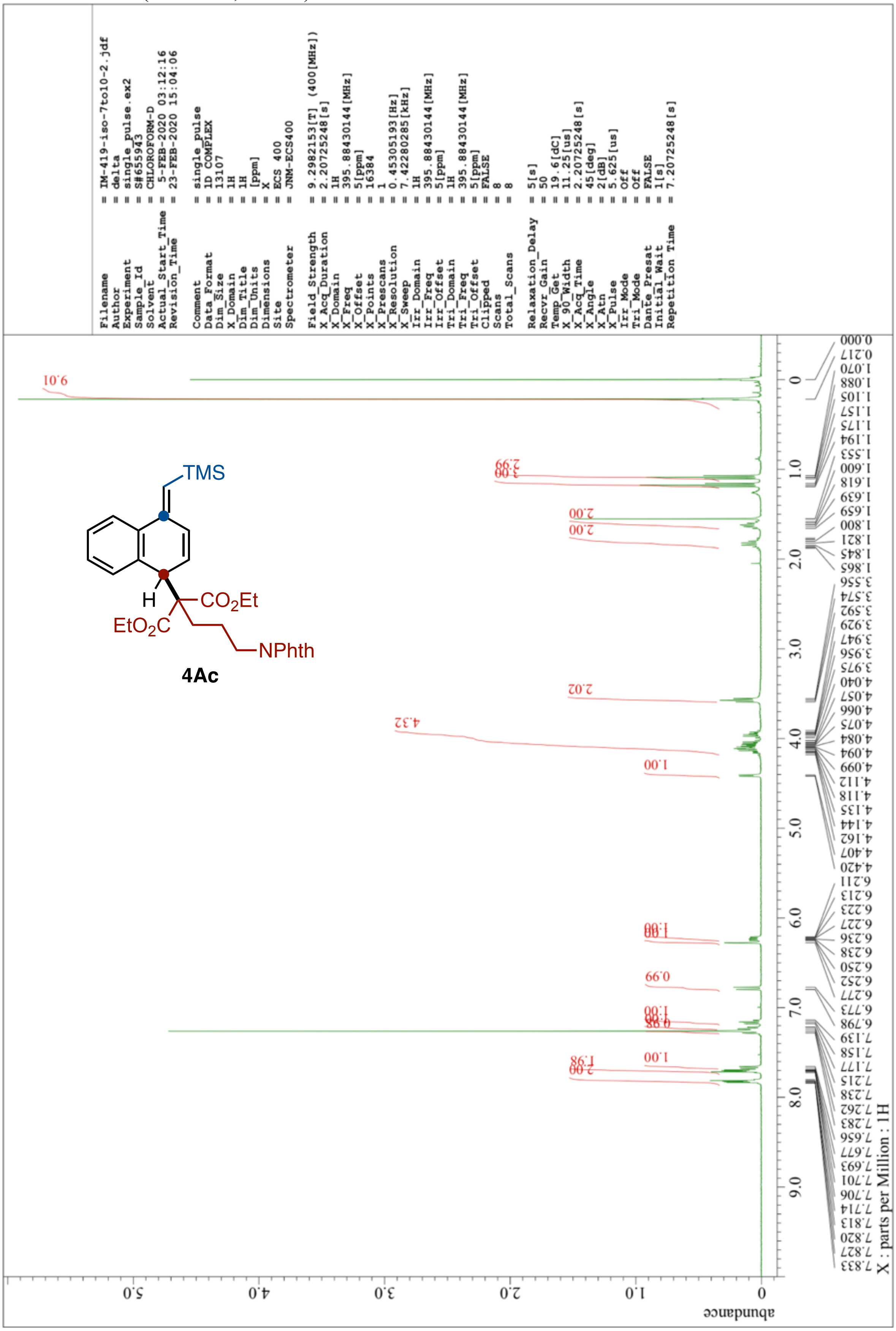

^{13}C NMR of **4Ac** (101 MHz, $CDCl_3$)

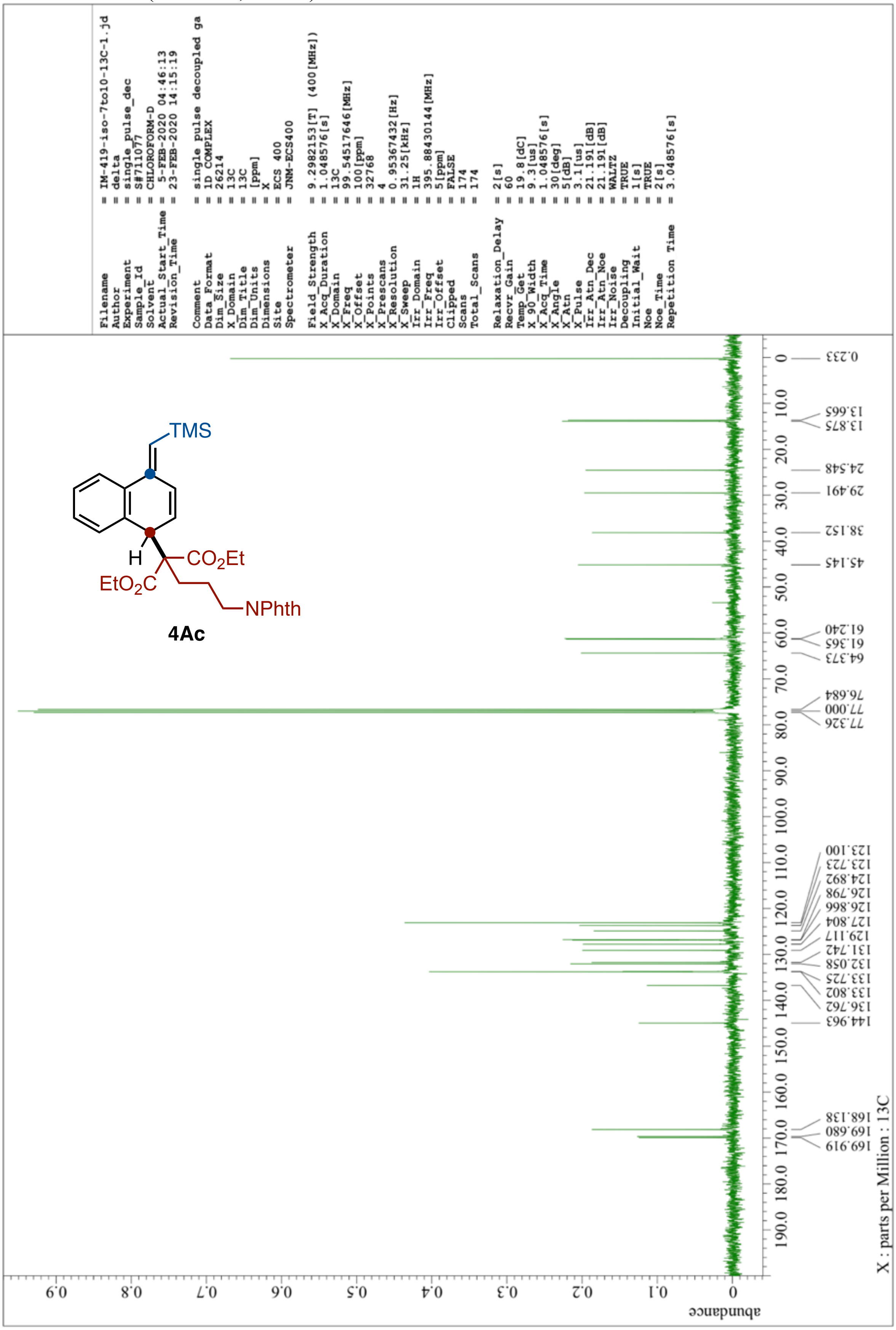

^{1}H NMR of **4Ad** (400 MHz, $CDCl_3$)

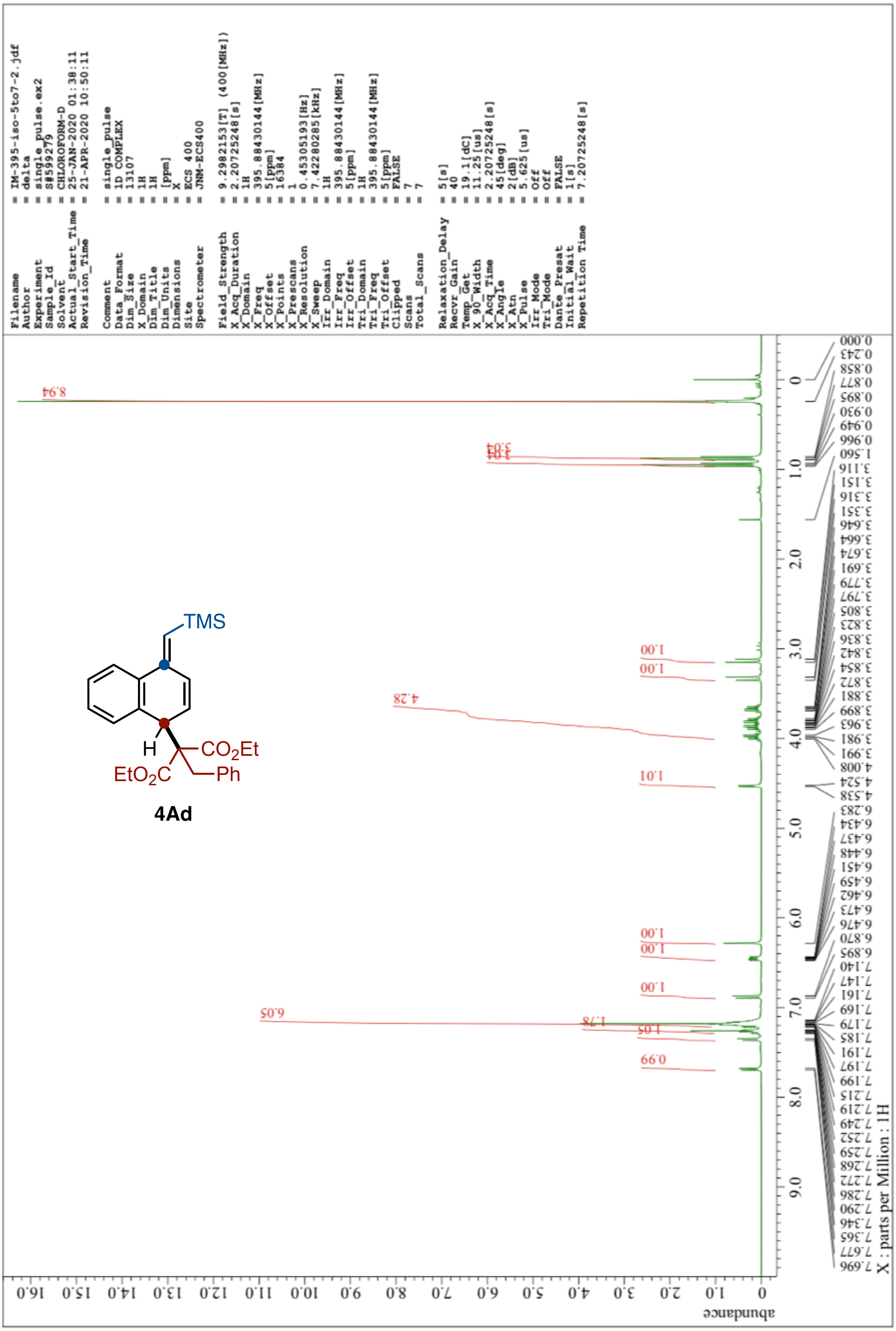

^{13}C NMR of **4Ad** (101 MHz, $CDCl_3$)

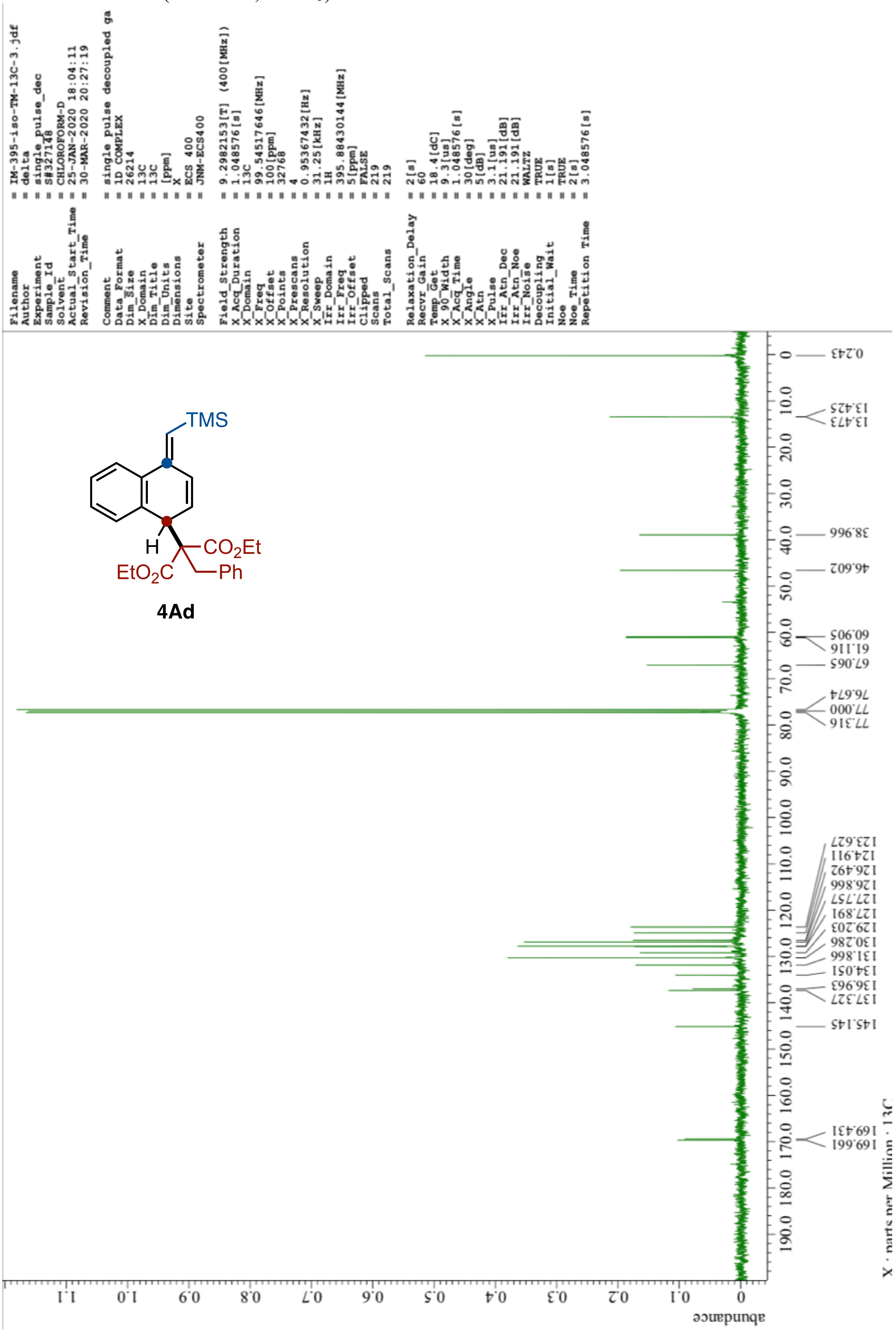

^{1}H NMR of **4He** (400 MHz, $CDCl_3$)

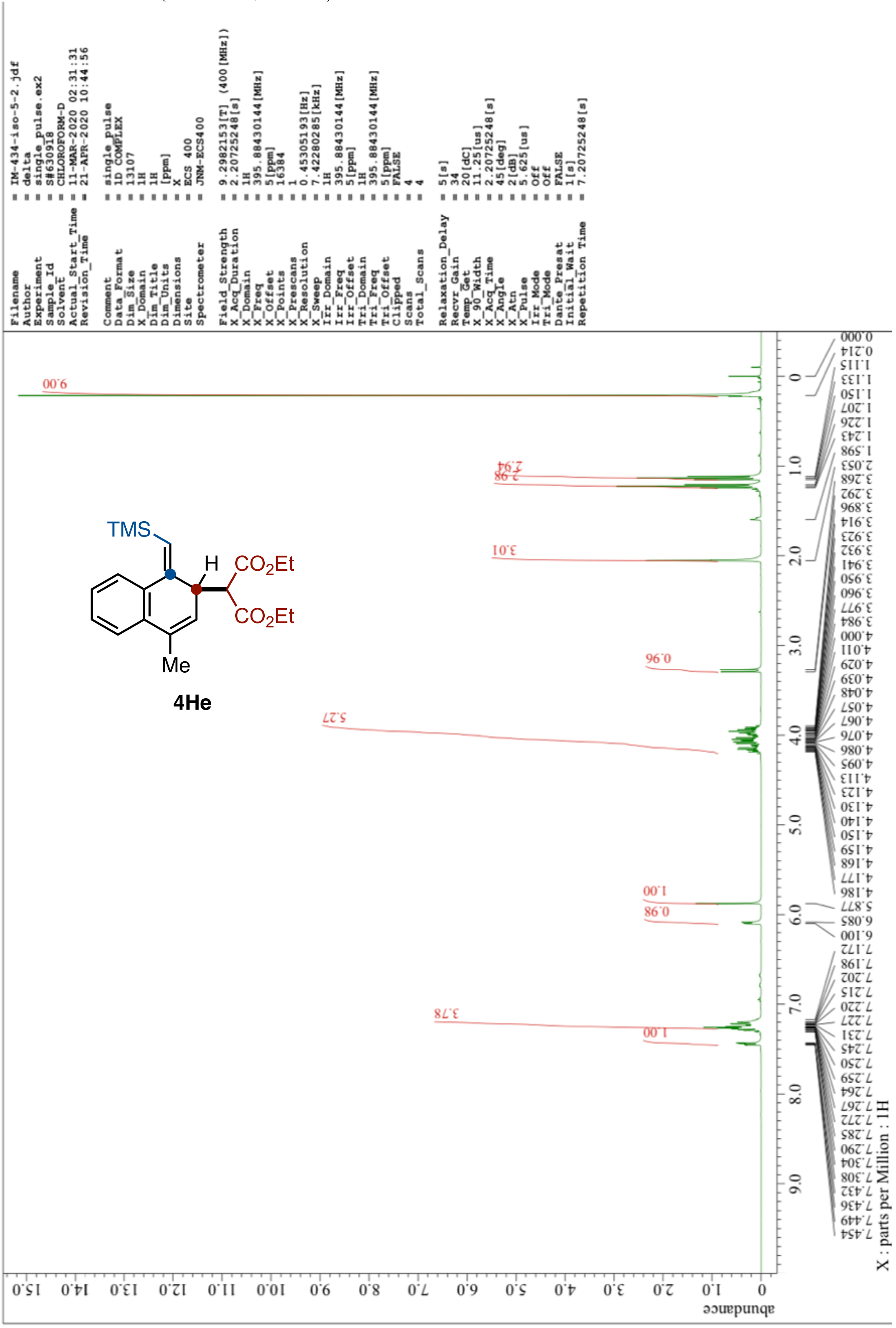

^{13}C NMR of **4He** (101 MHz, $CDCl_3$)

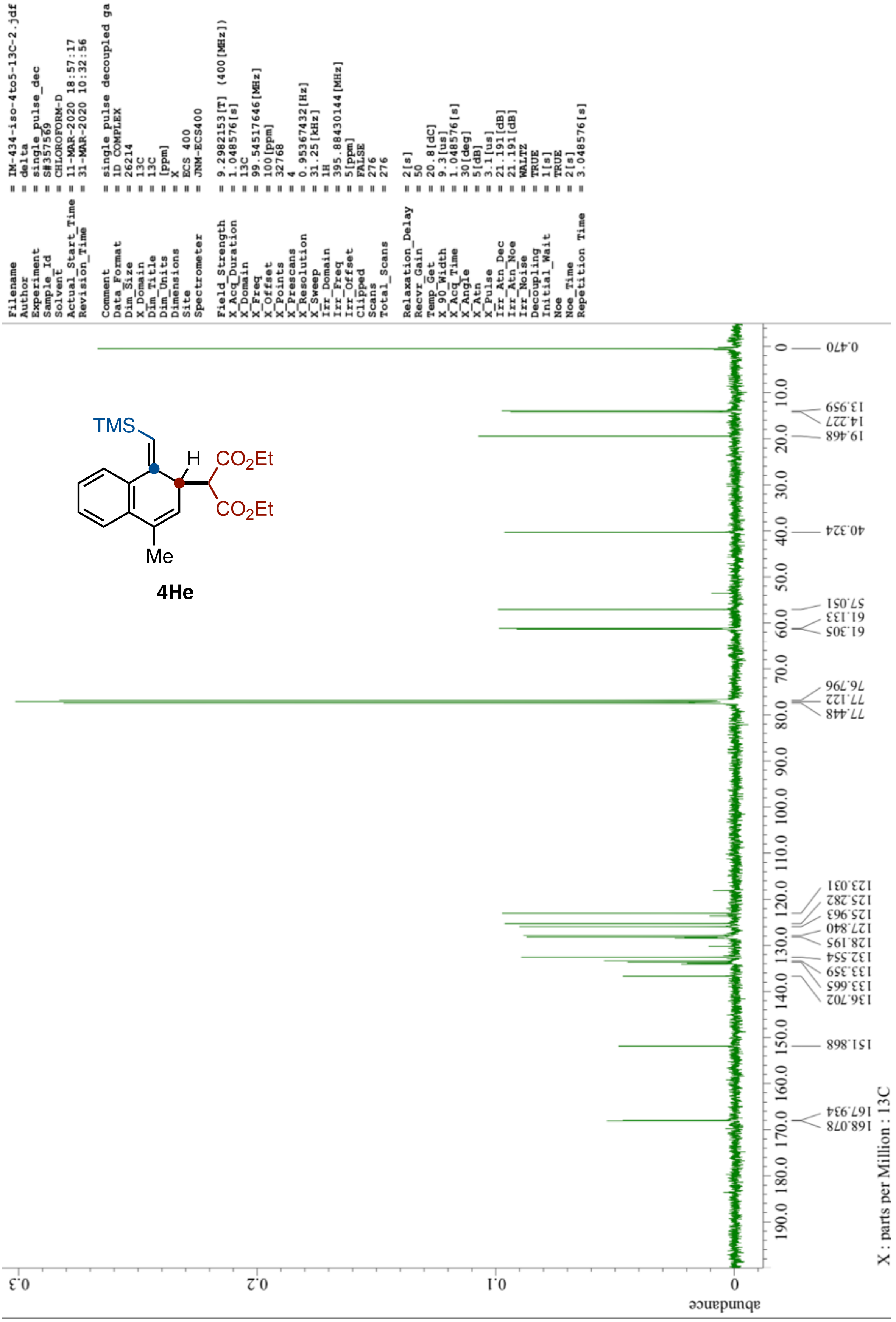

^{1}H NMR of **4Ja** (400 MHz, $CDCl_3$)

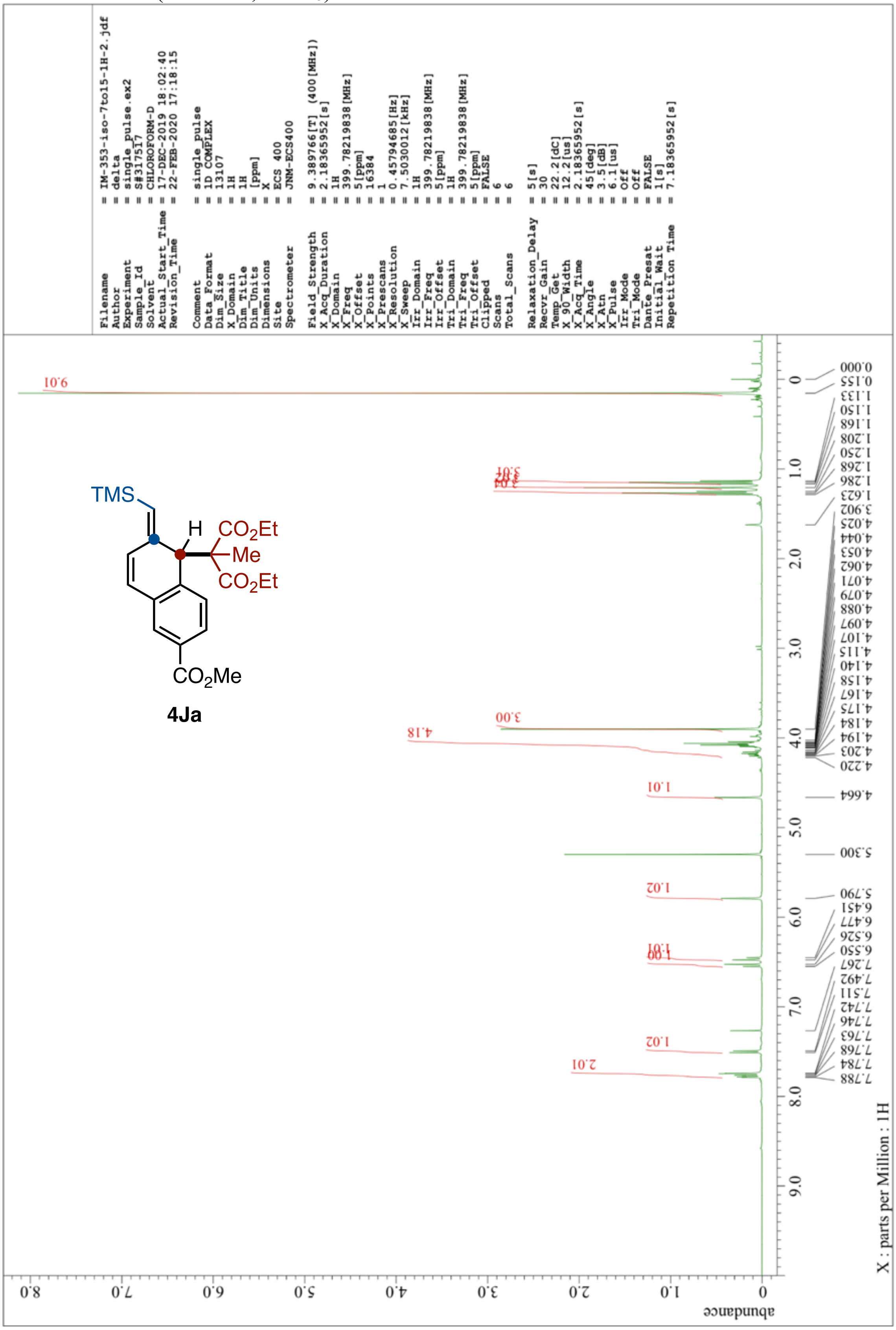

^{13}C NMR of **4Ja** (101 MHz, $CDCl_3$)

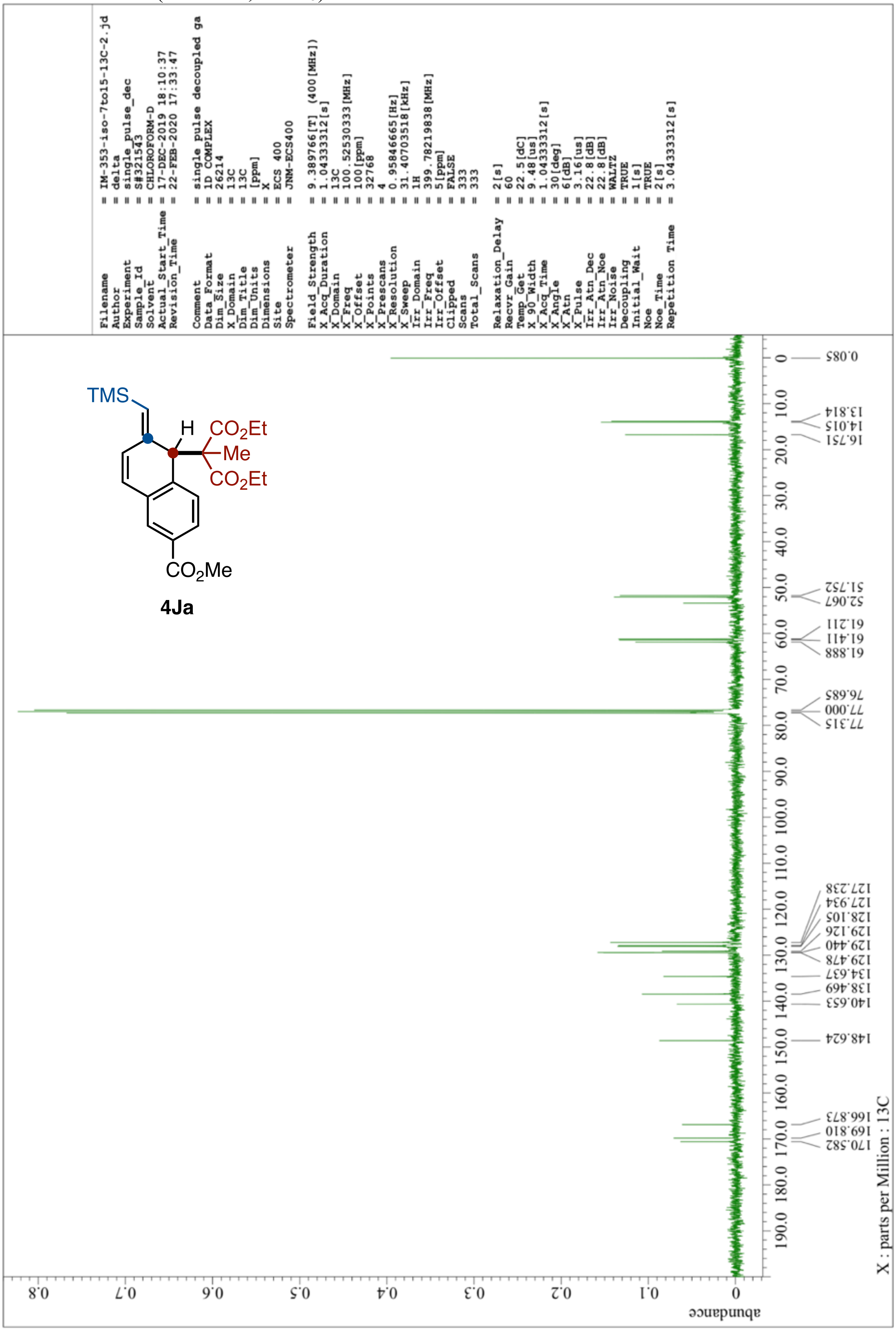

^{1}H NMR of **4Ka** (400 MHz, $CDCl_3$)

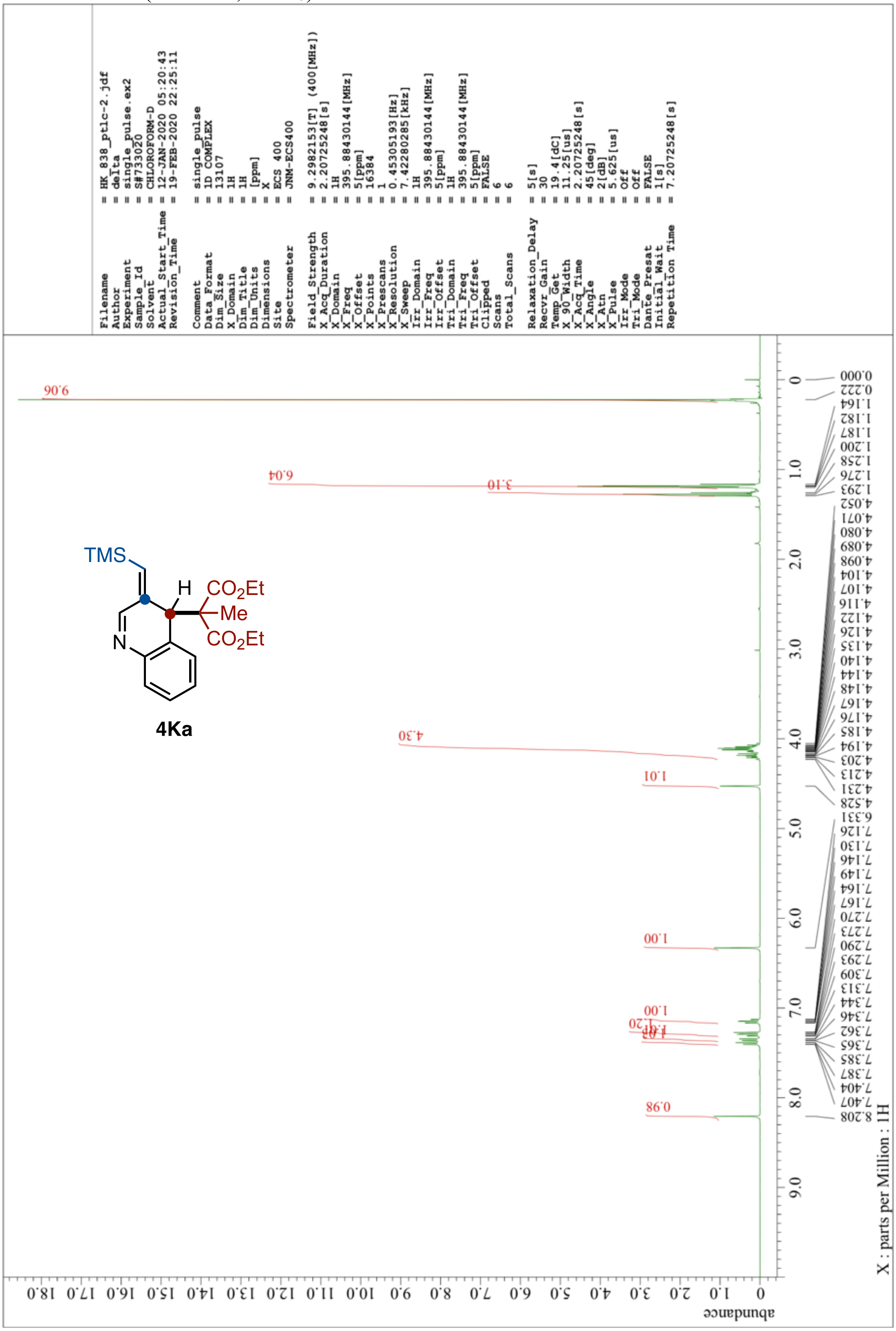

^{13}C NMR of **4Ka** (101 MHz, $CDCl_3$)

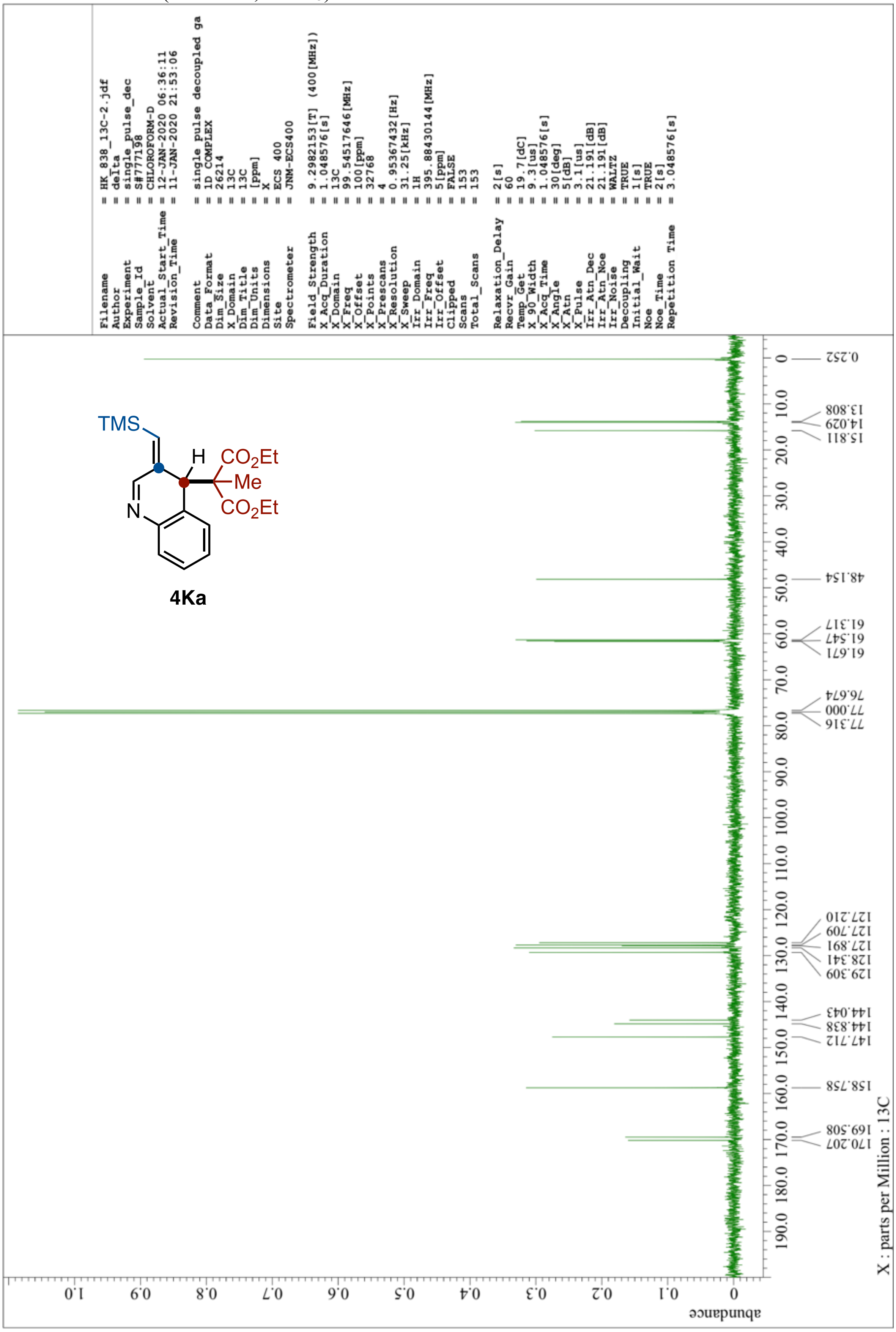

^{1}H NMR of **6Aa** (400 MHz, $CDCl_3$)

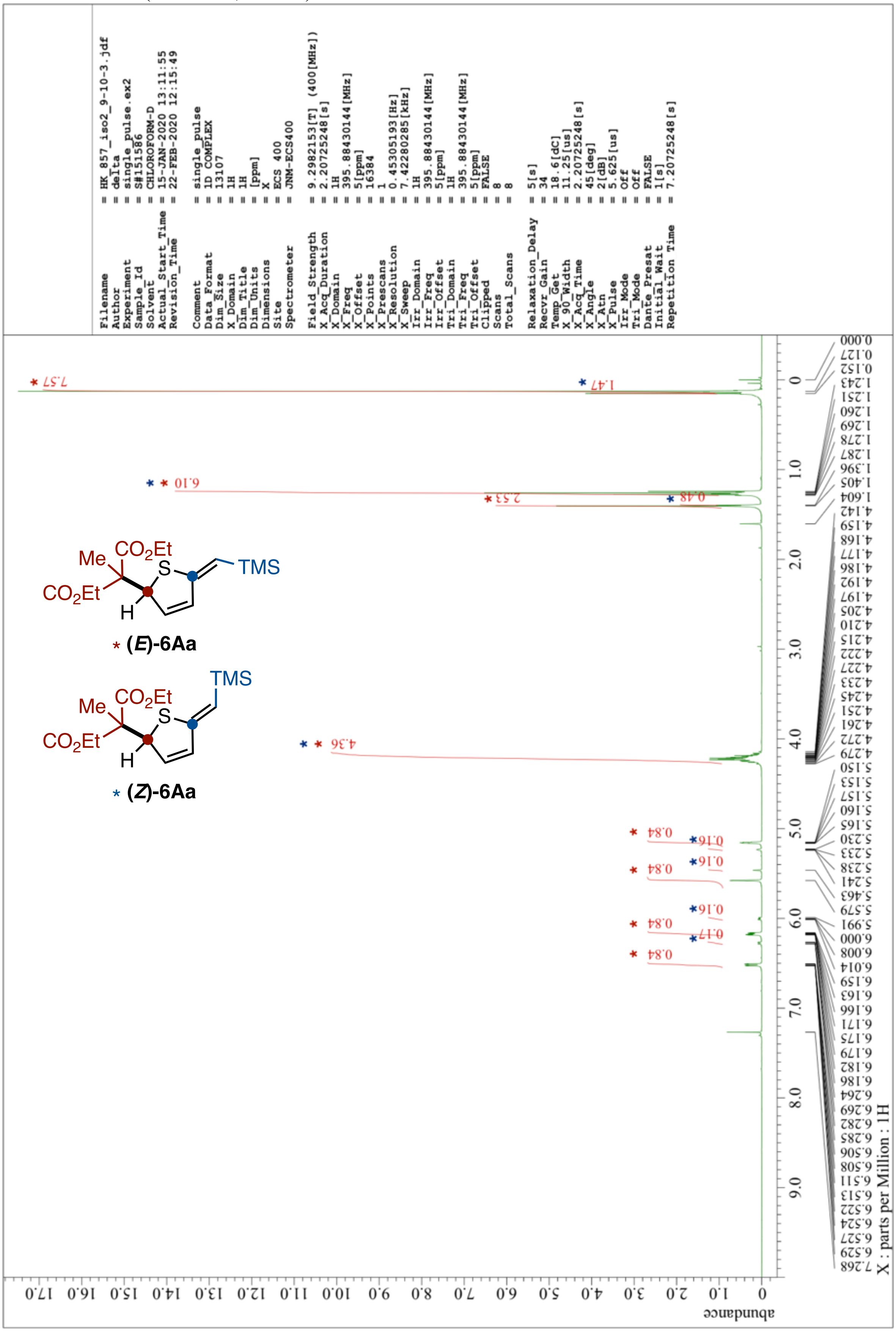

^{13}C NMR of **6Aa** (101 MHz, $CDCl_3$)

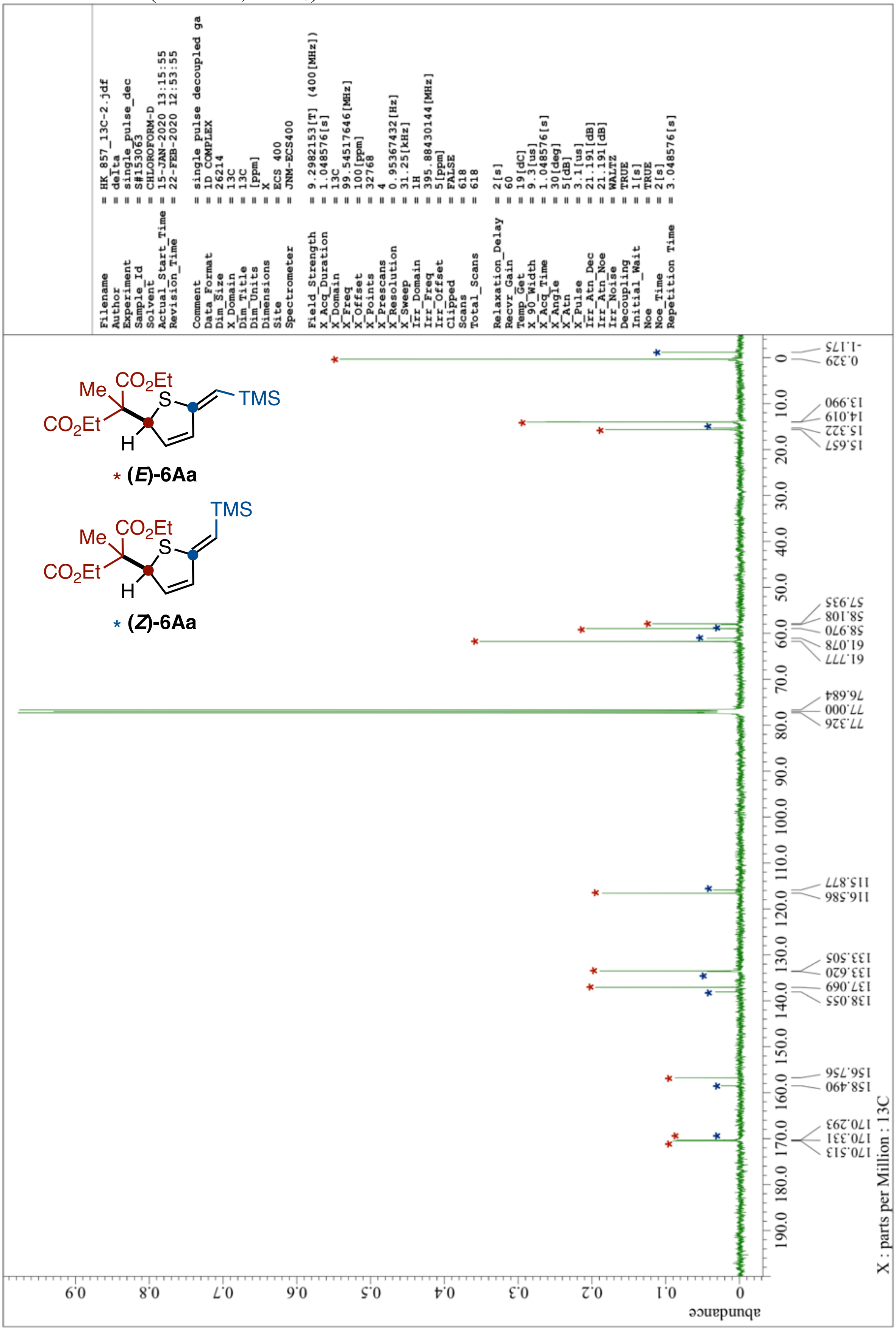

^{1}H NMR of **6Ab** (400 MHz, $CDCl_3$)

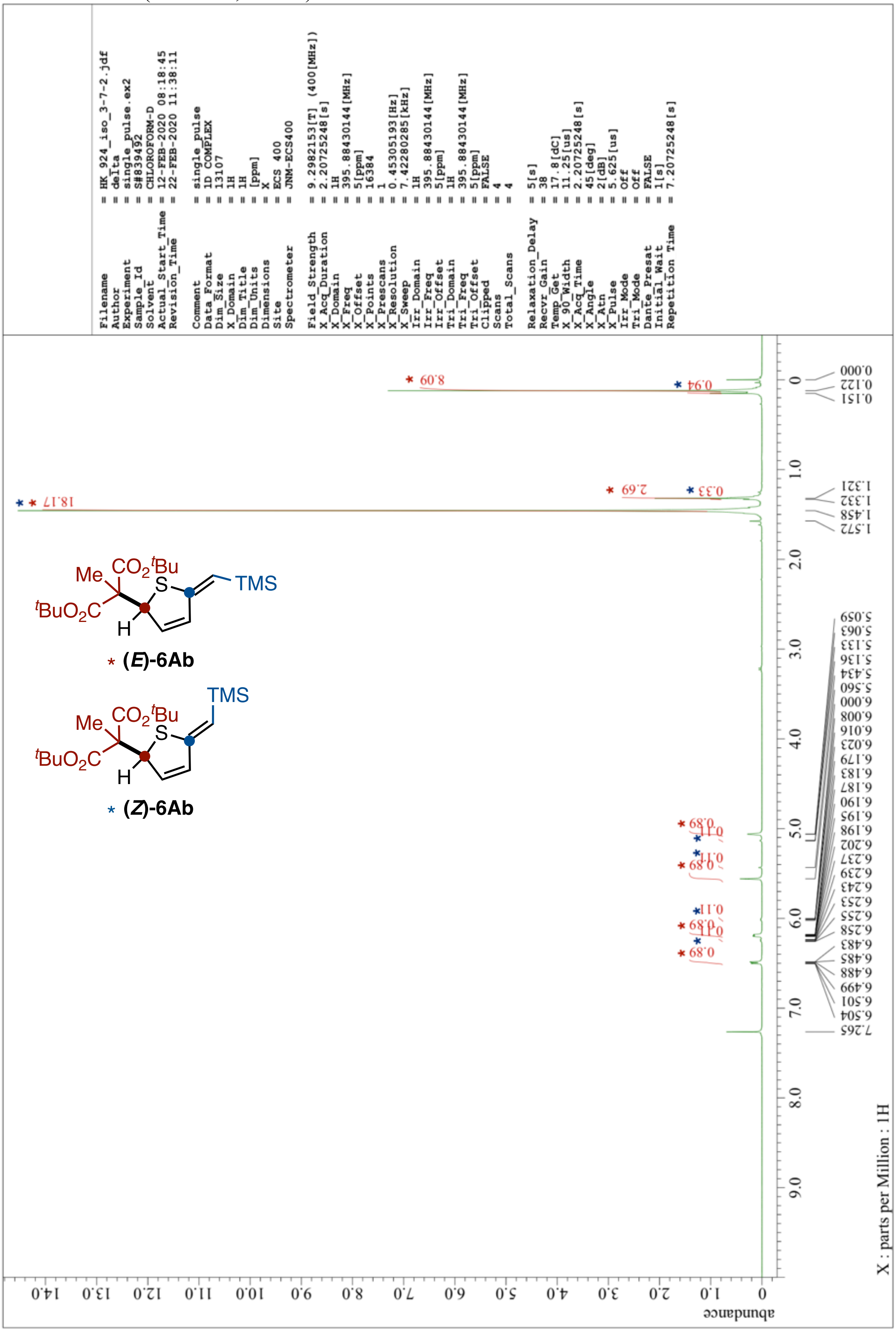

^{13}C NMR of **6Ab** (101 MHz, $CDCl_3$)

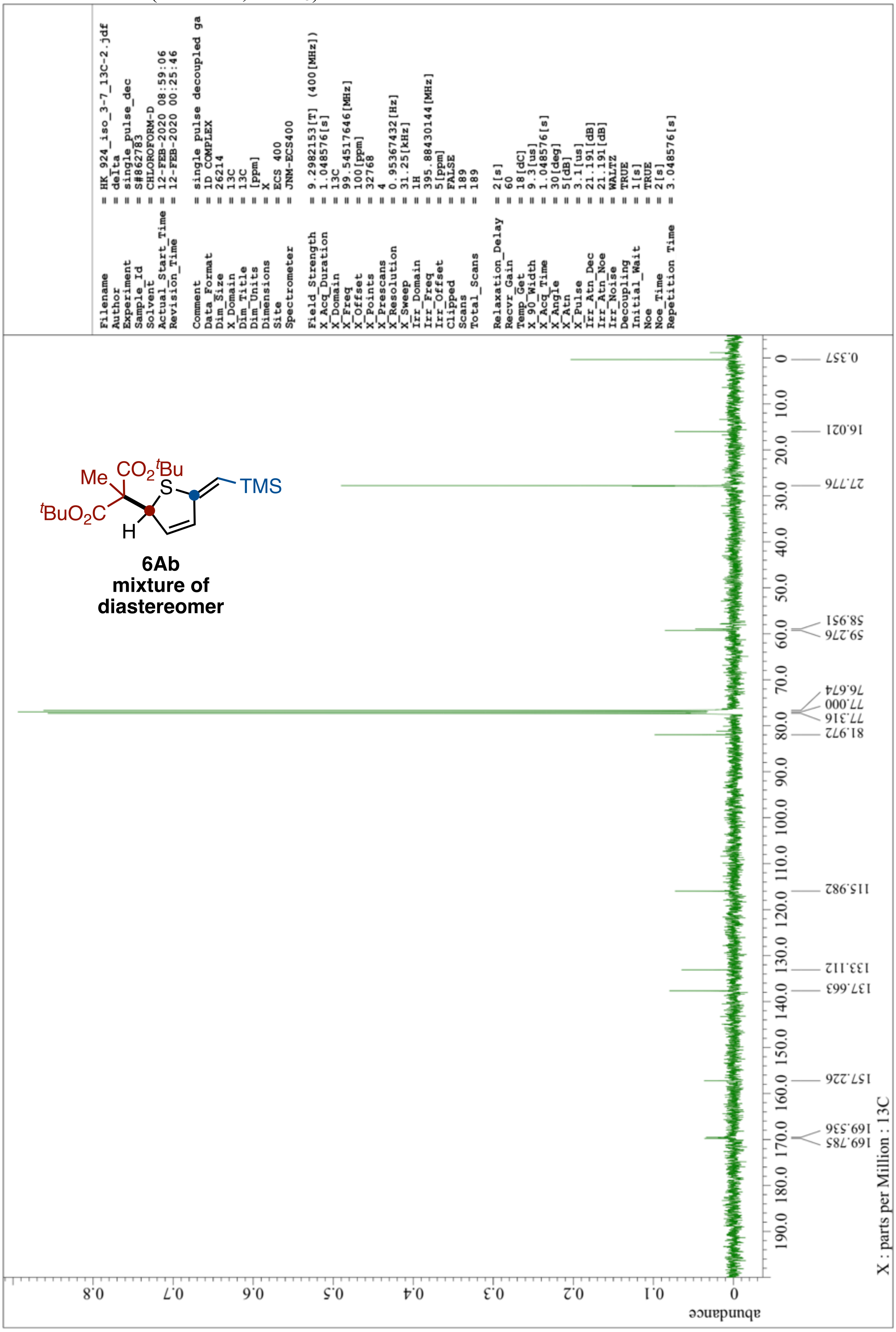

^{1}H NMR of **6Ba** (400 MHz, $CDCl_3$)

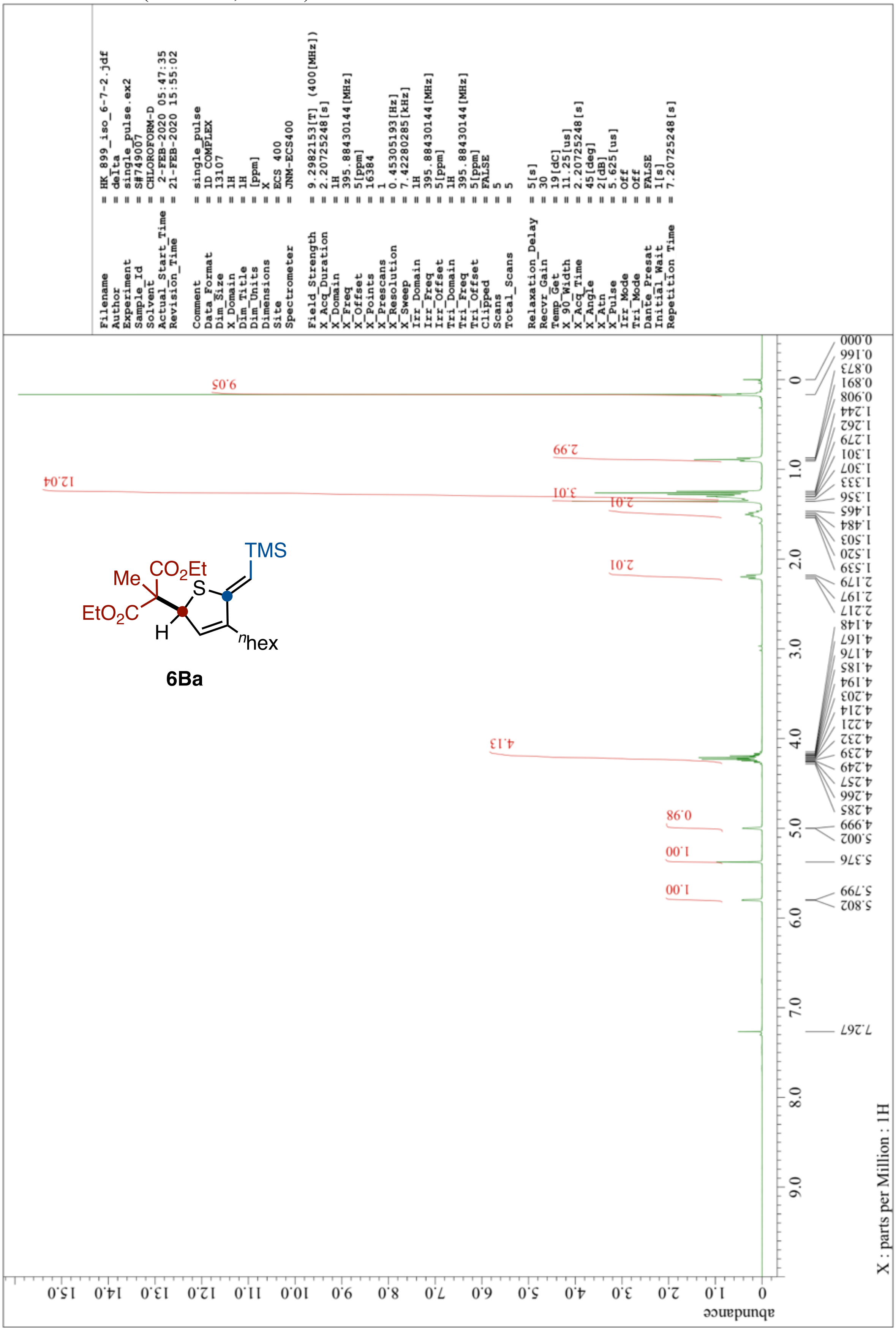

^{13}C NMR of **6Ba** (101 MHz, $CDCl_3$)

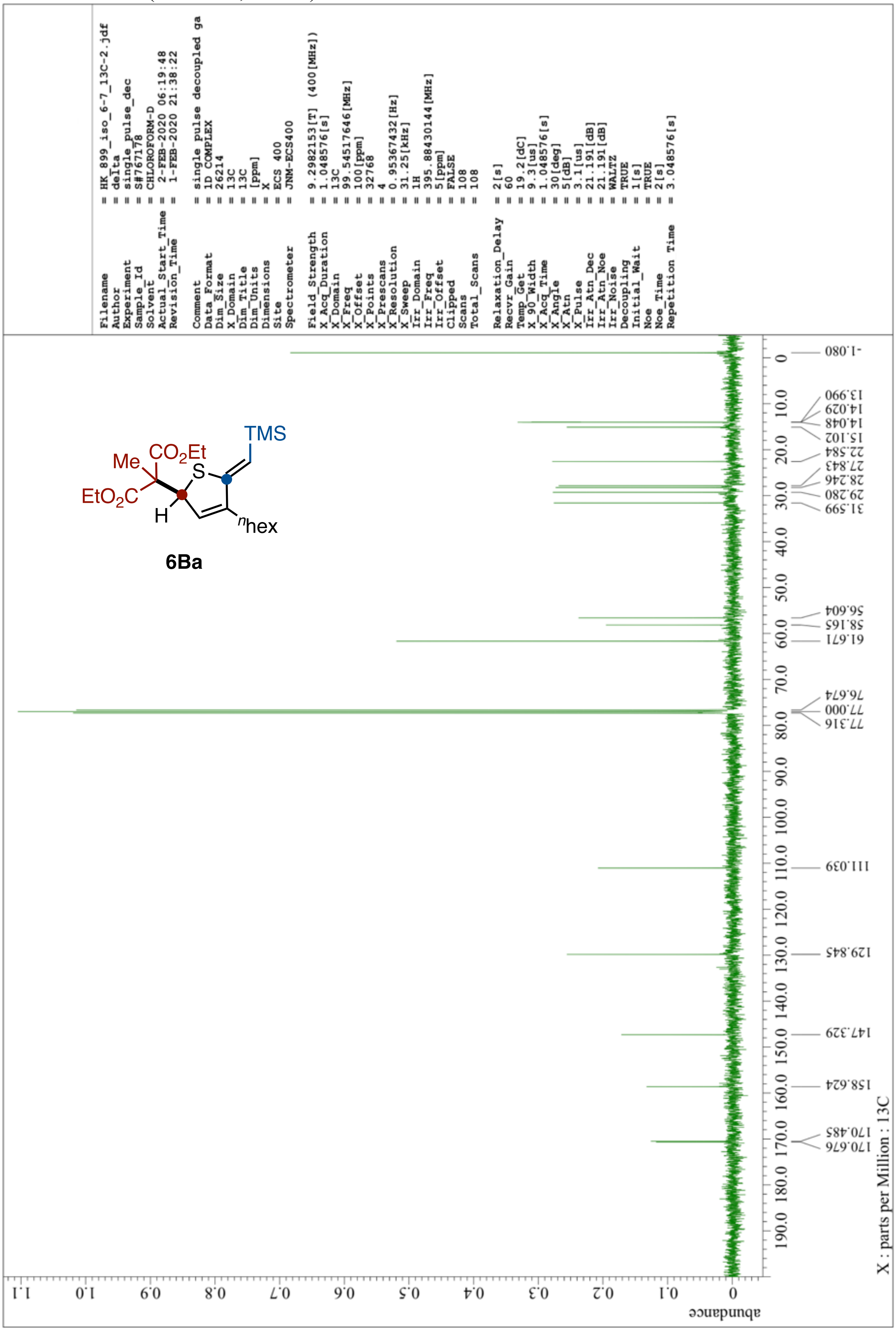

^{1}H NMR of **6Ca** (400 MHz, $CDCl_3$)

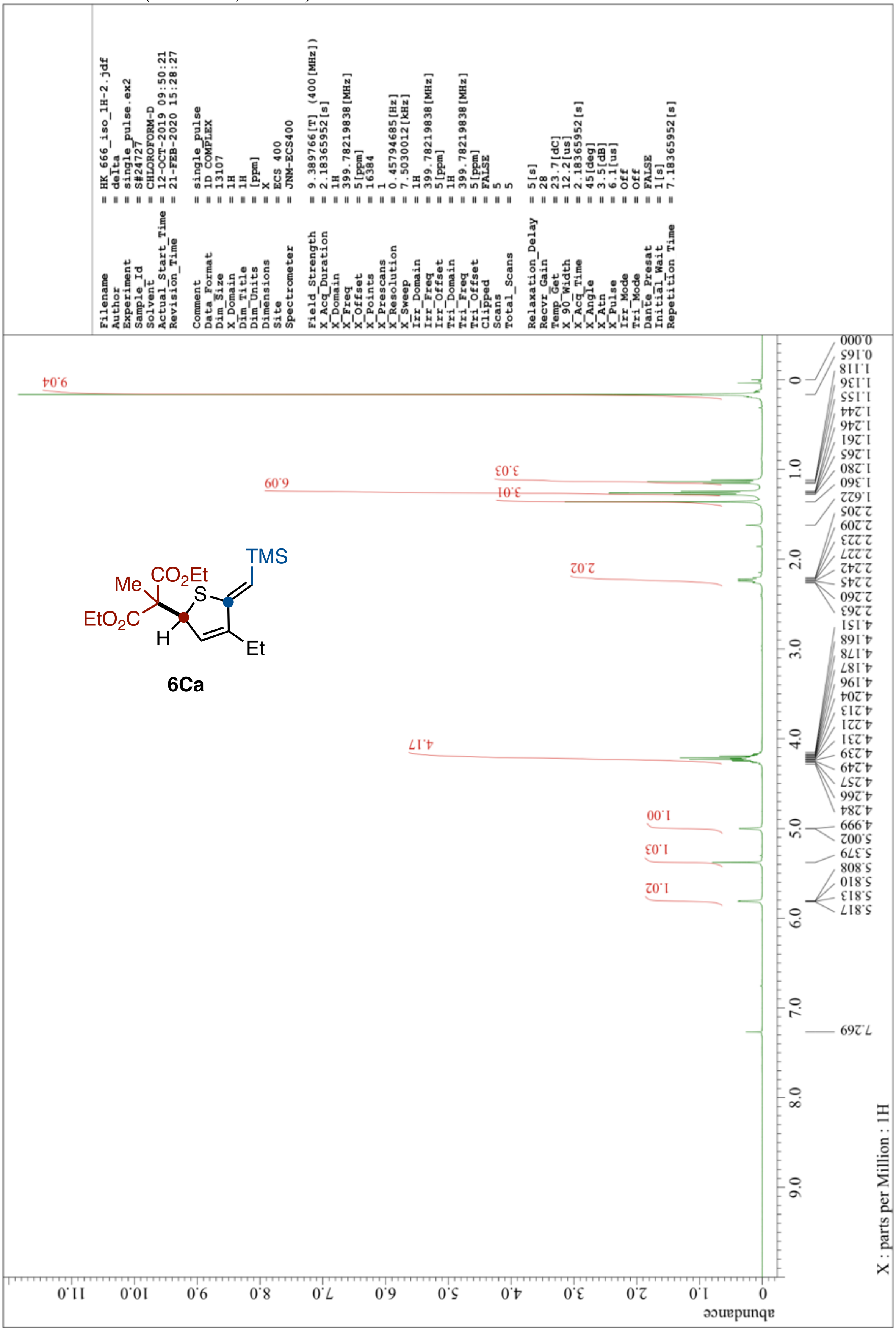

^{13}C NMR of **6Ca** (101 MHz, $CDCl_3$)

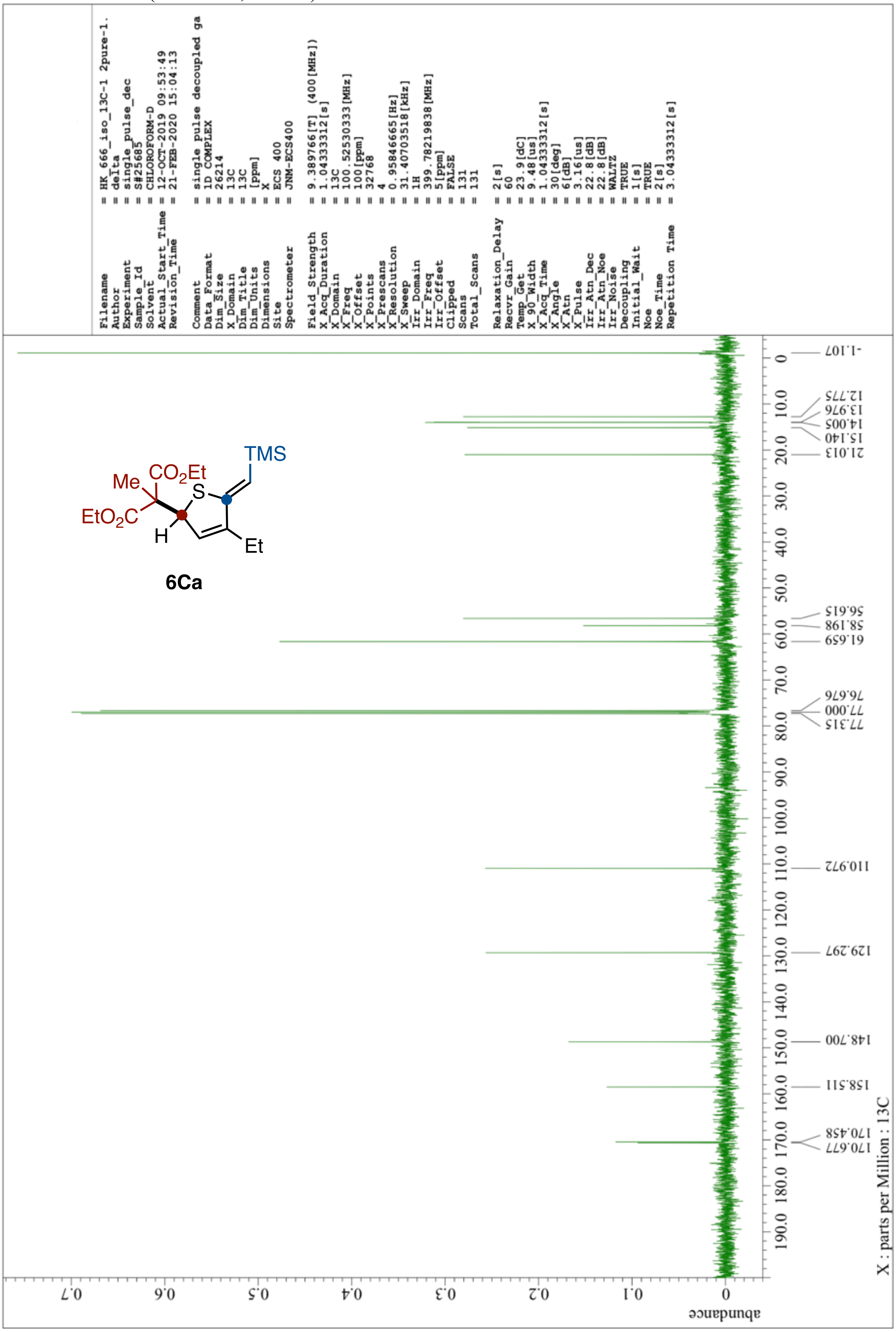

^{1}H NMR of **6Da** (400 MHz, $CDCl_3$)

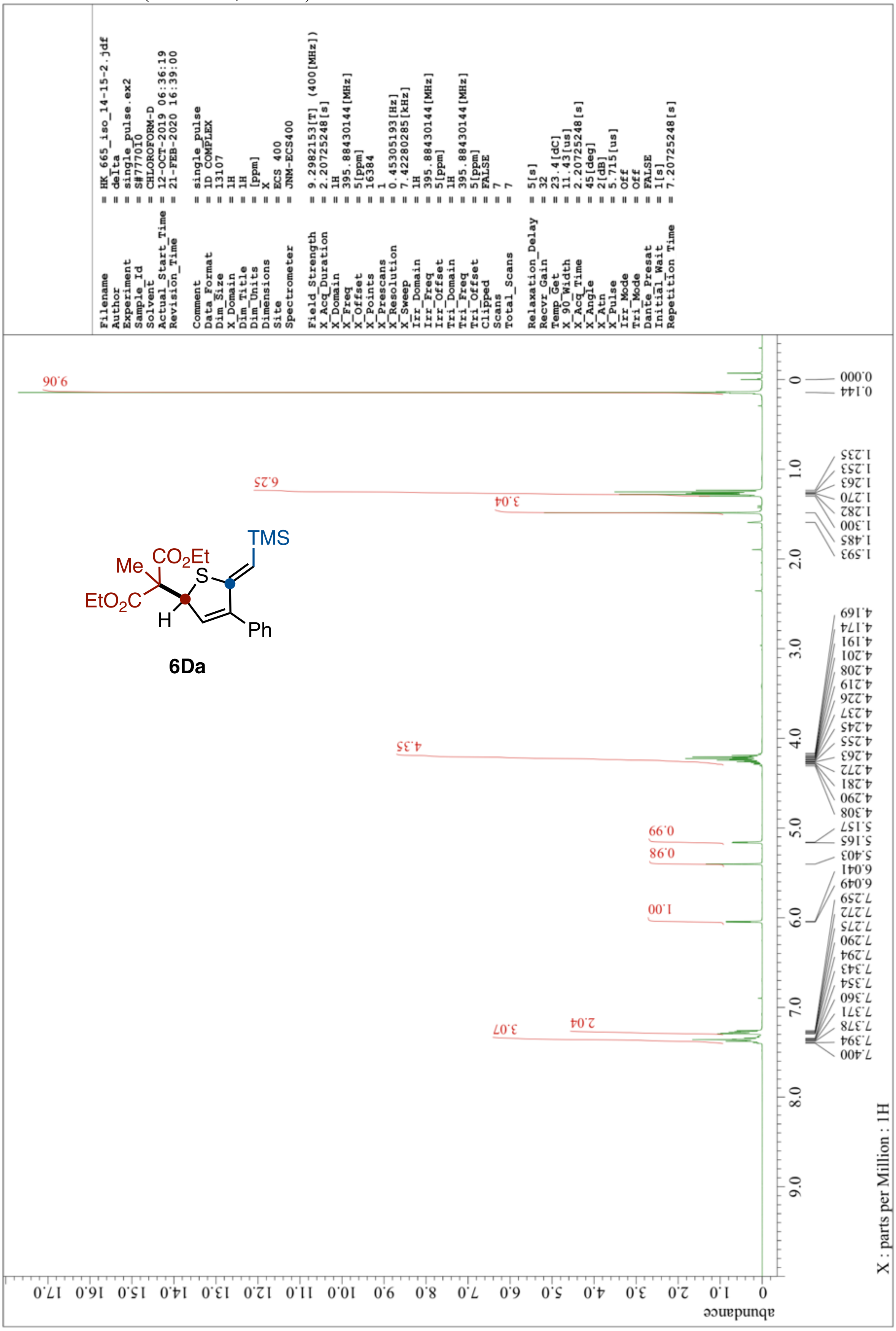

^{13}C NMR of **6Da** (101 MHz, $CDCl_3$)

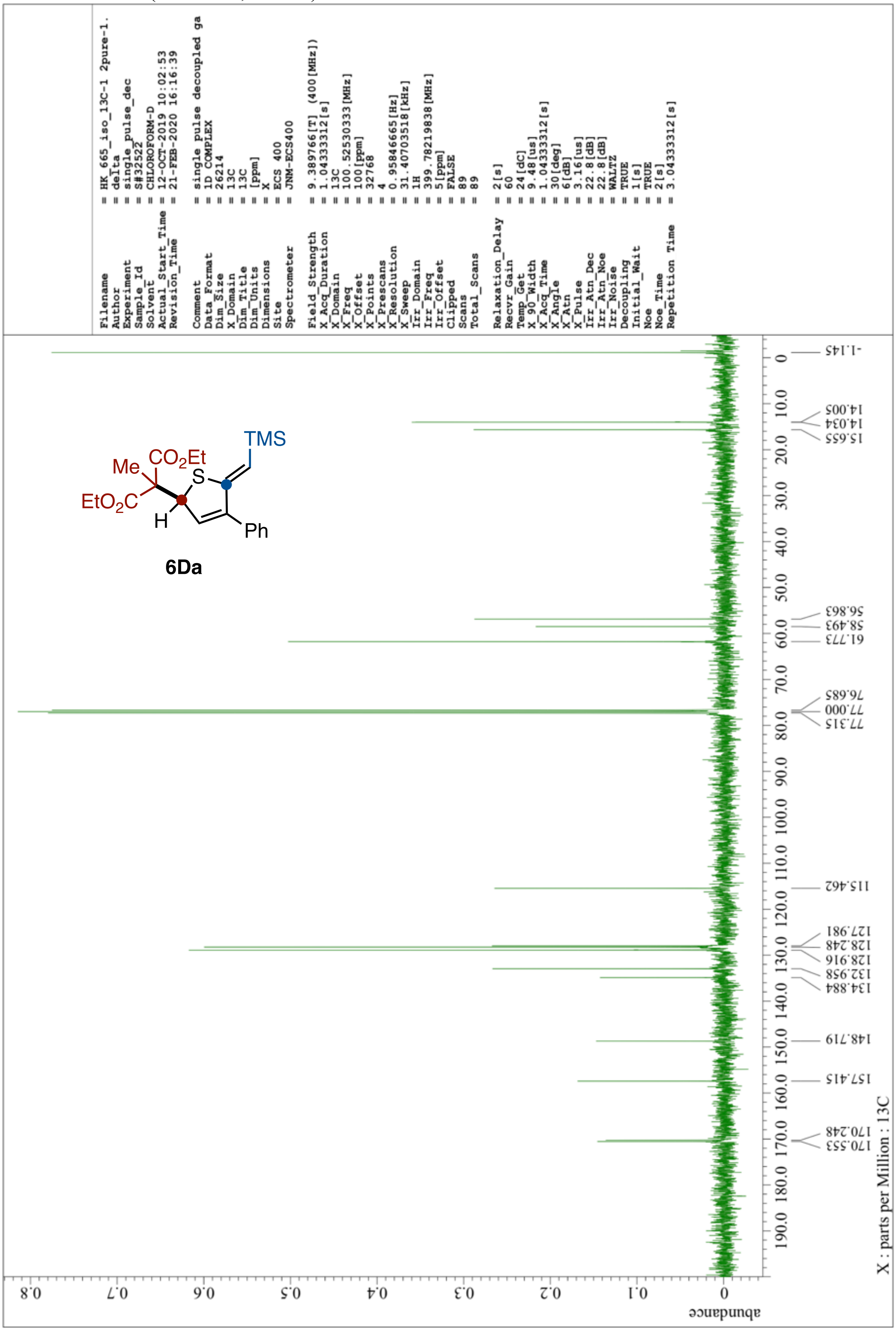

^{1}H NMR of **6Ea** (400 MHz, $CDCl_3$)

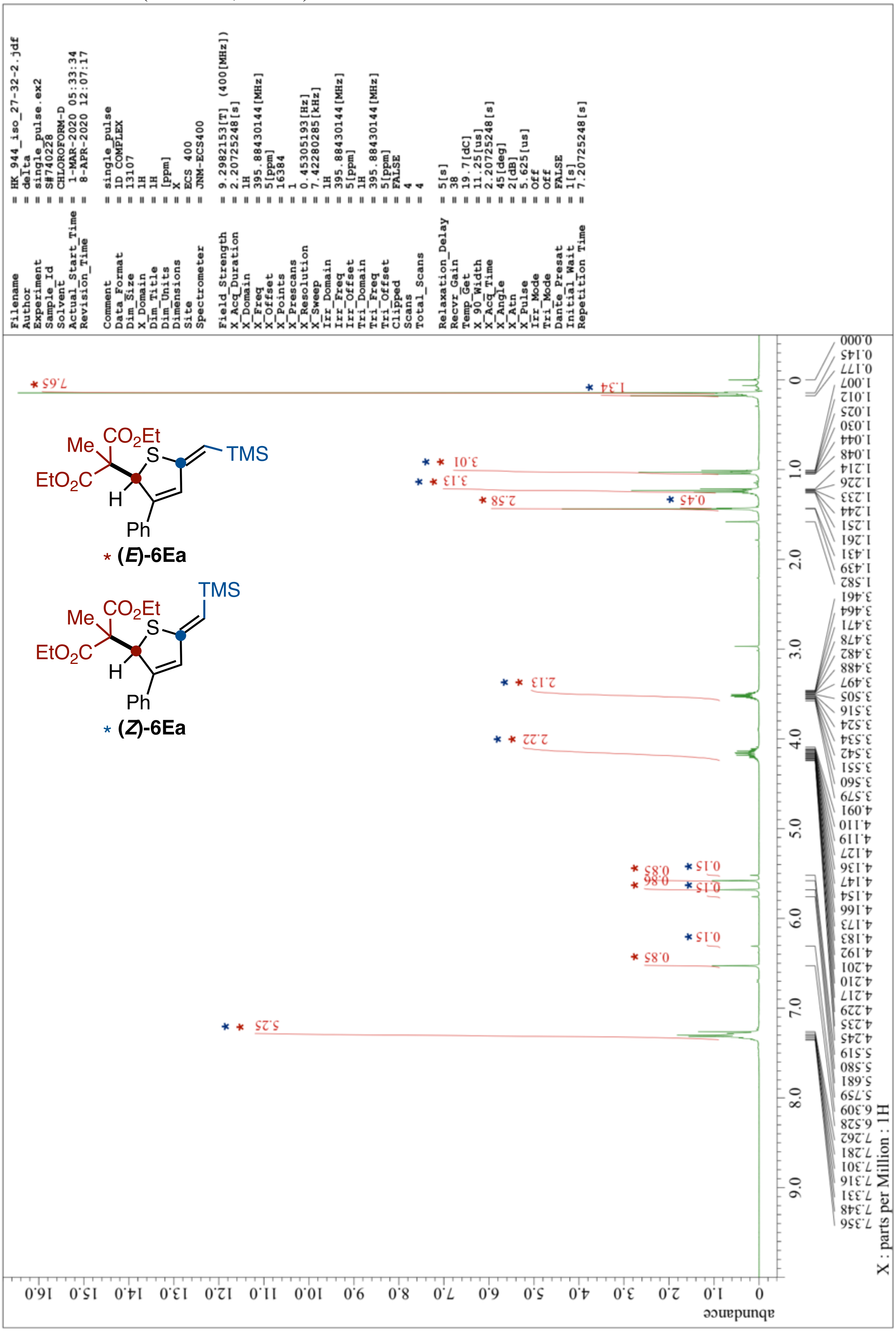

^{13}C NMR of **6Ea** (101 MHz, $CDCl_3$)

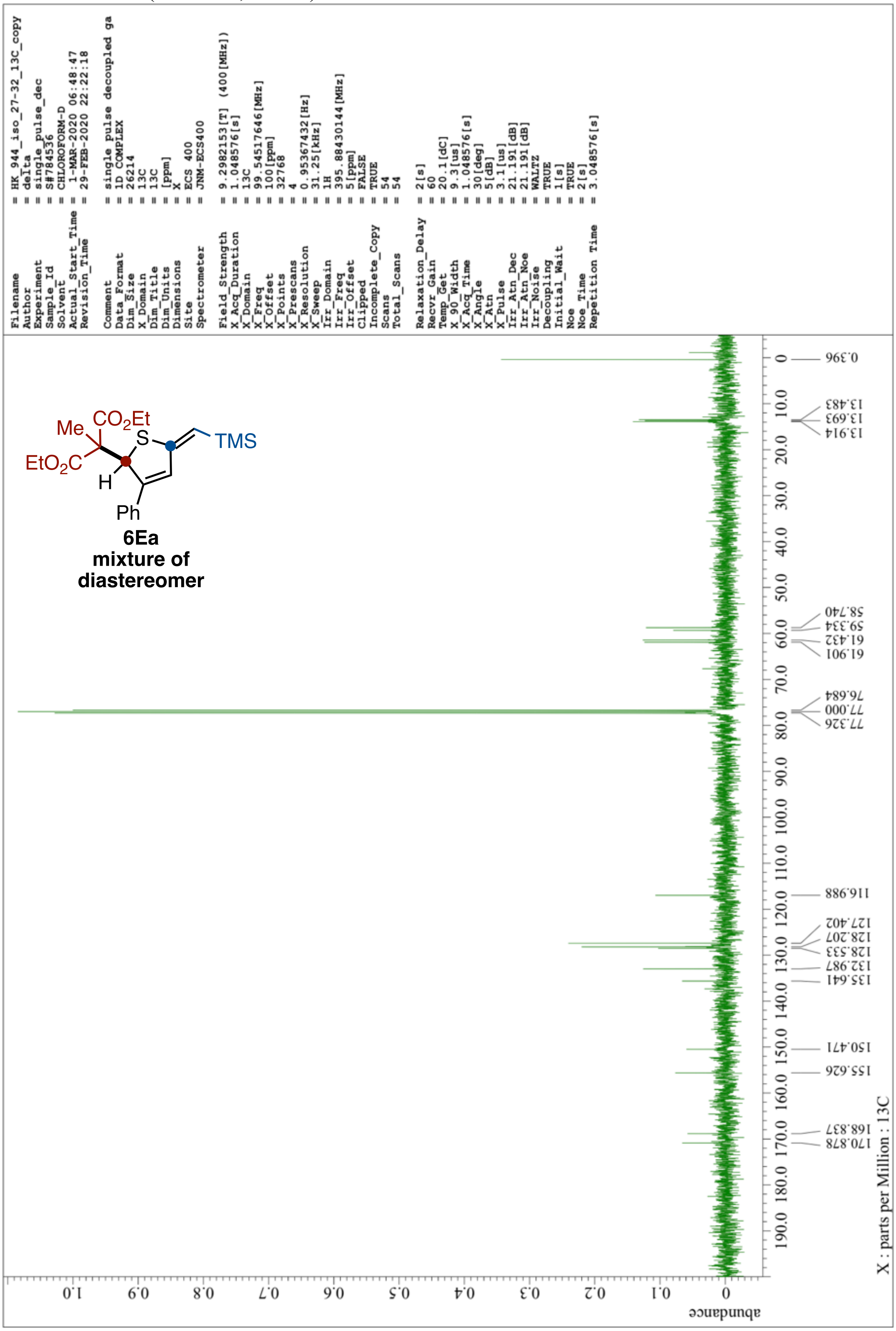

^{1}H NMR of **6Fa** (400 MHz, $CDCl_3$)

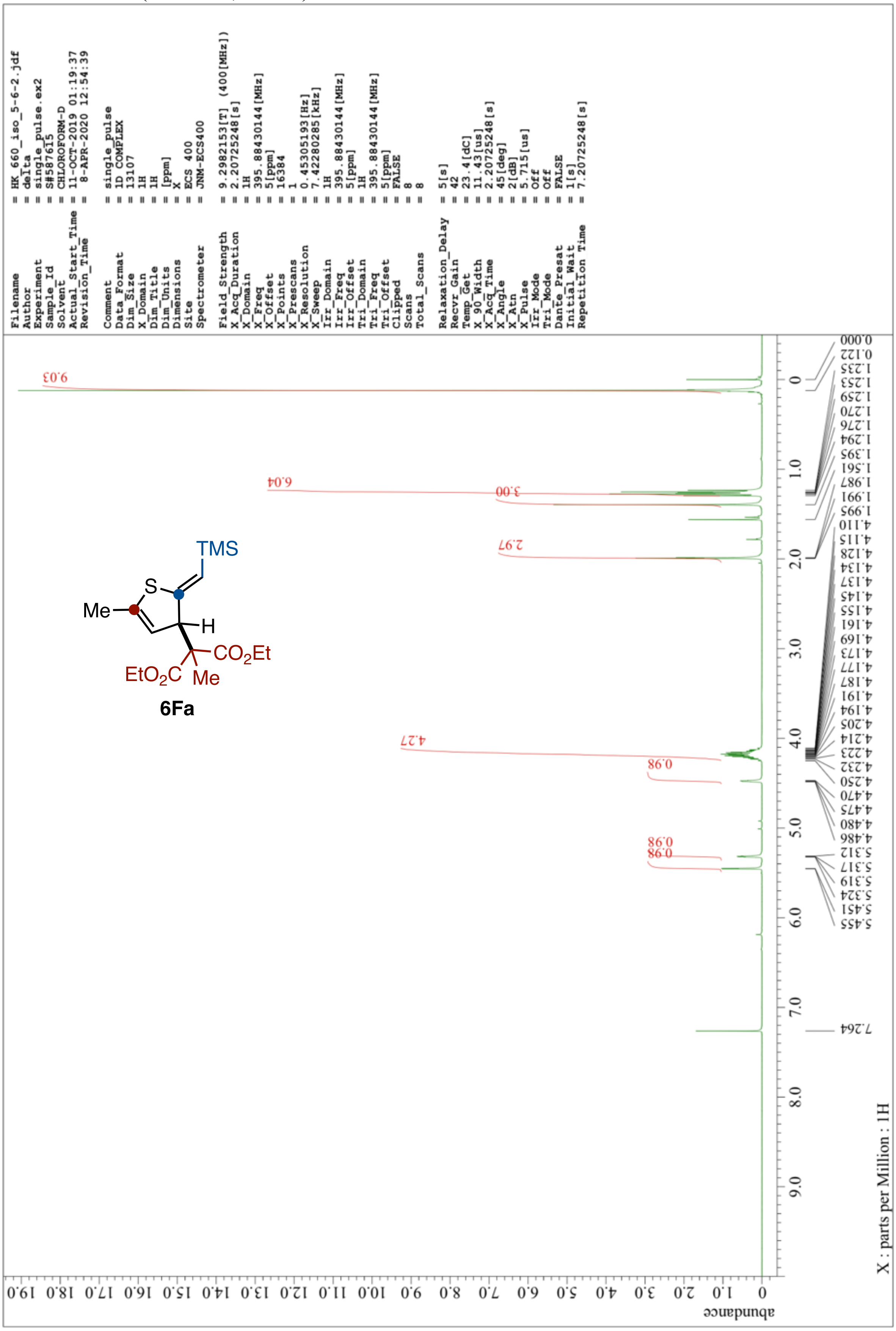

^{13}C NMR of **6Fa** (101 MHz, $CDCl_3$)

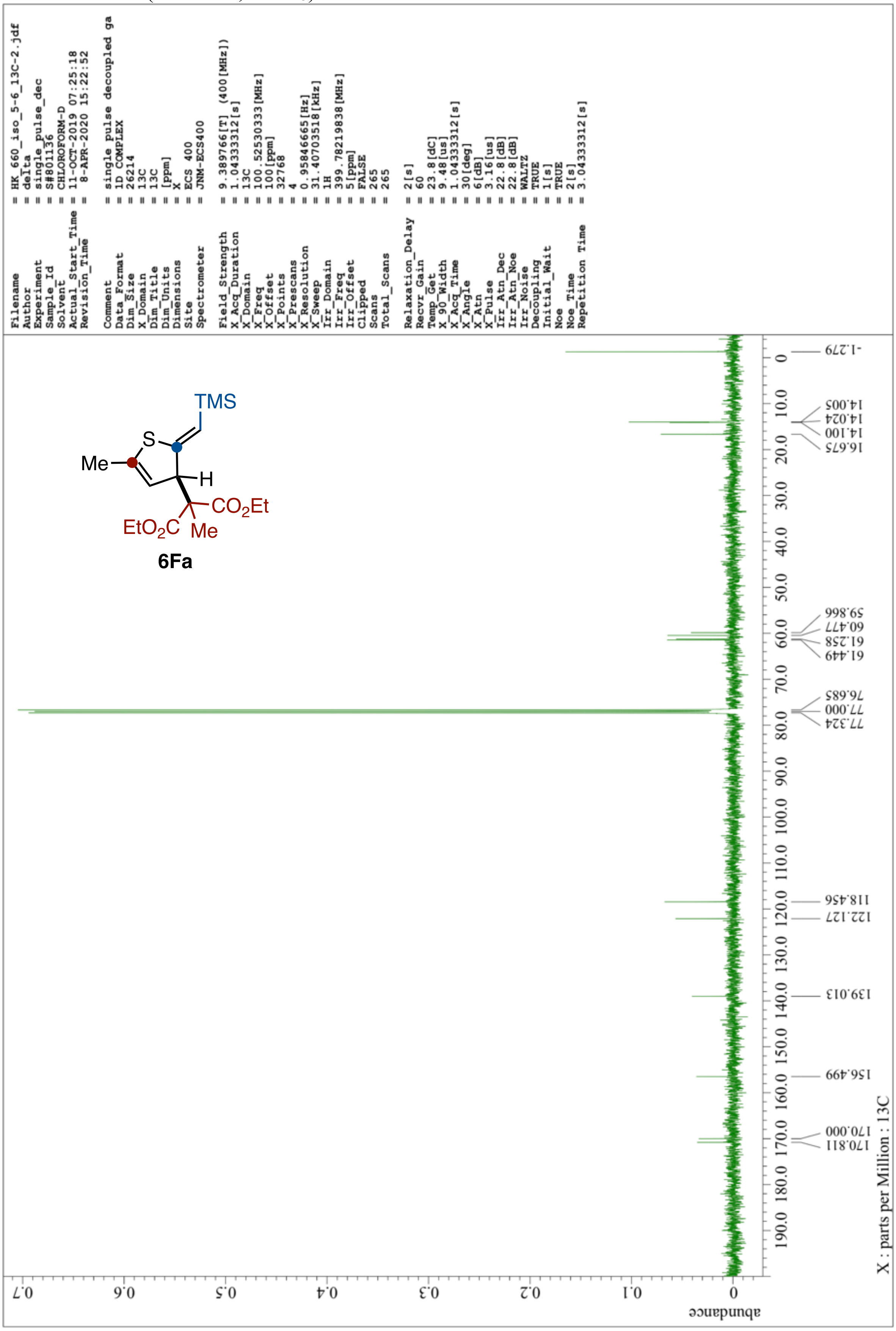

^{1}H NMR of **6Ga** (400 MHz, $CDCl_3$)

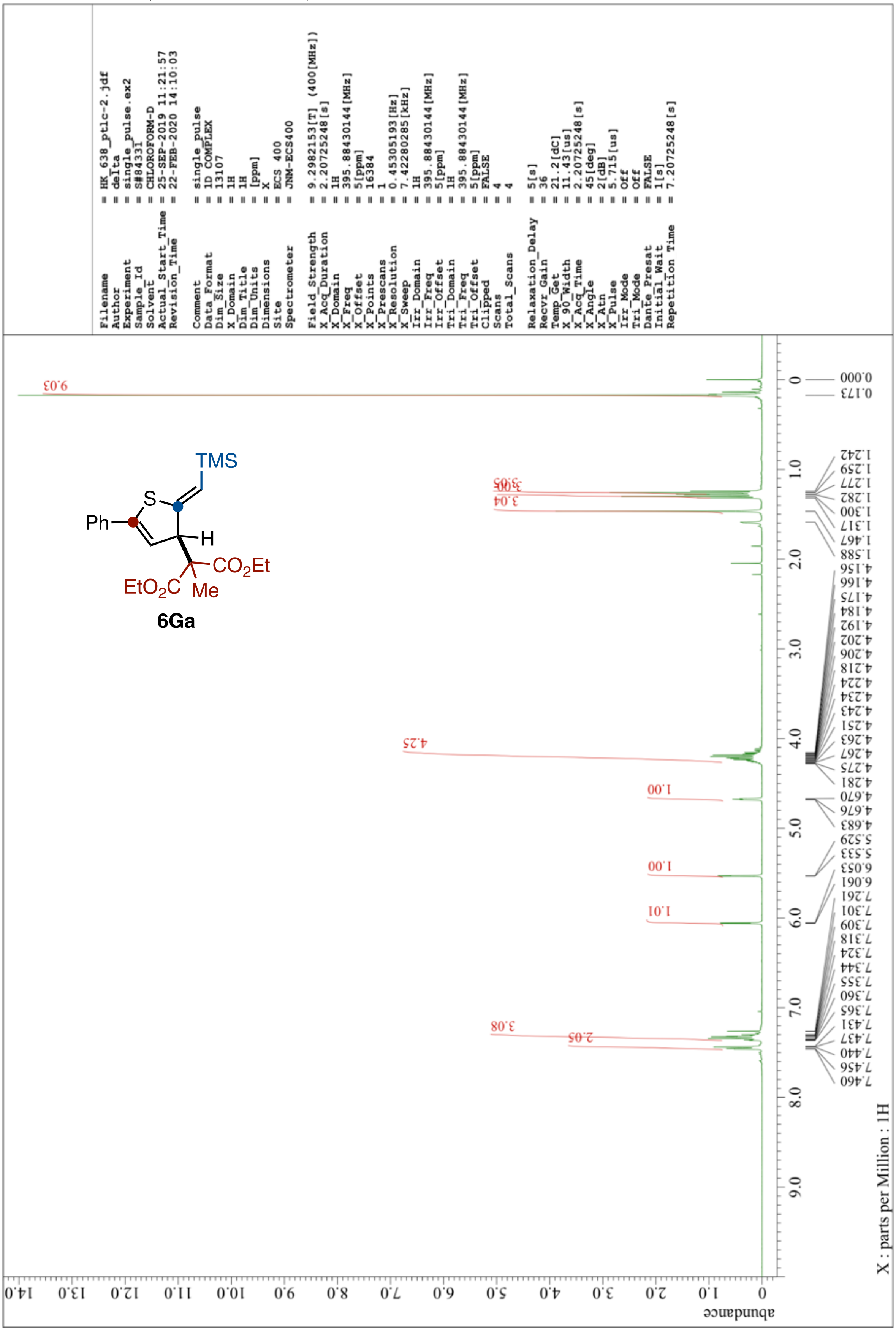

^{13}C NMR of **6Ga** (101 MHz, $CDCl_3$)

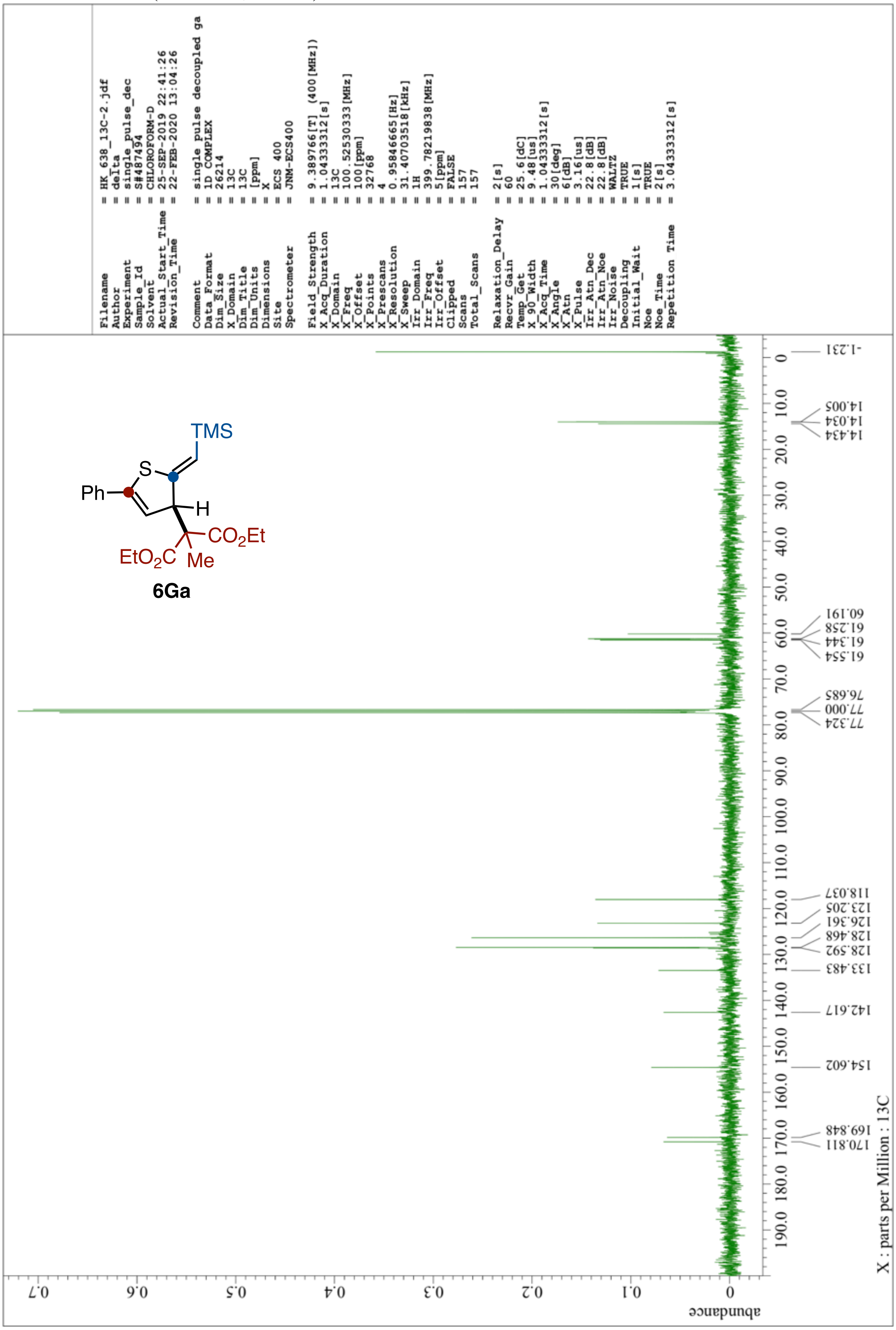

^{1}H NMR of **6Ha** (400 MHz, $CDCl_3$)

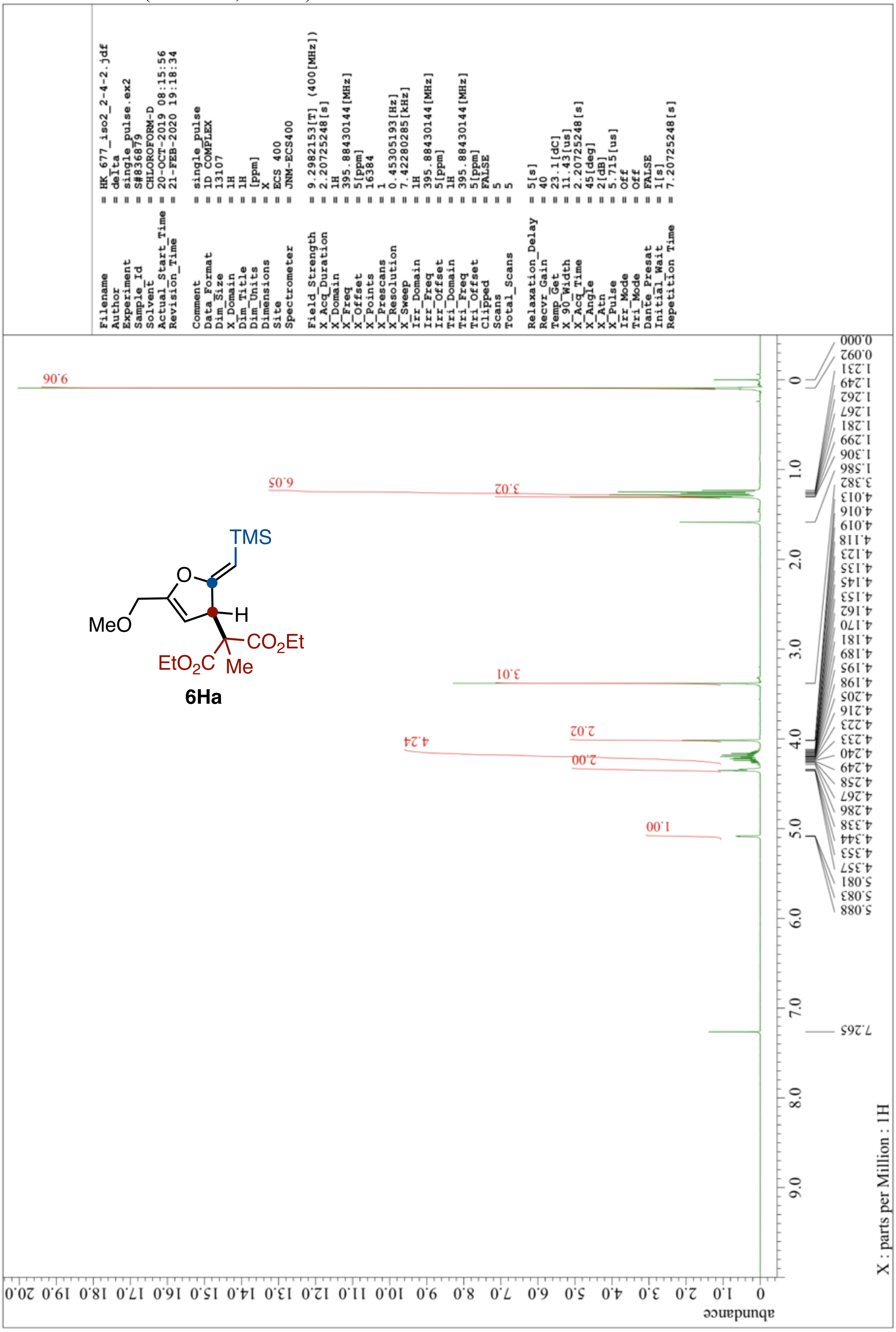

^{13}C NMR of **6Ha** (101 MHz, $CDCl_3$)

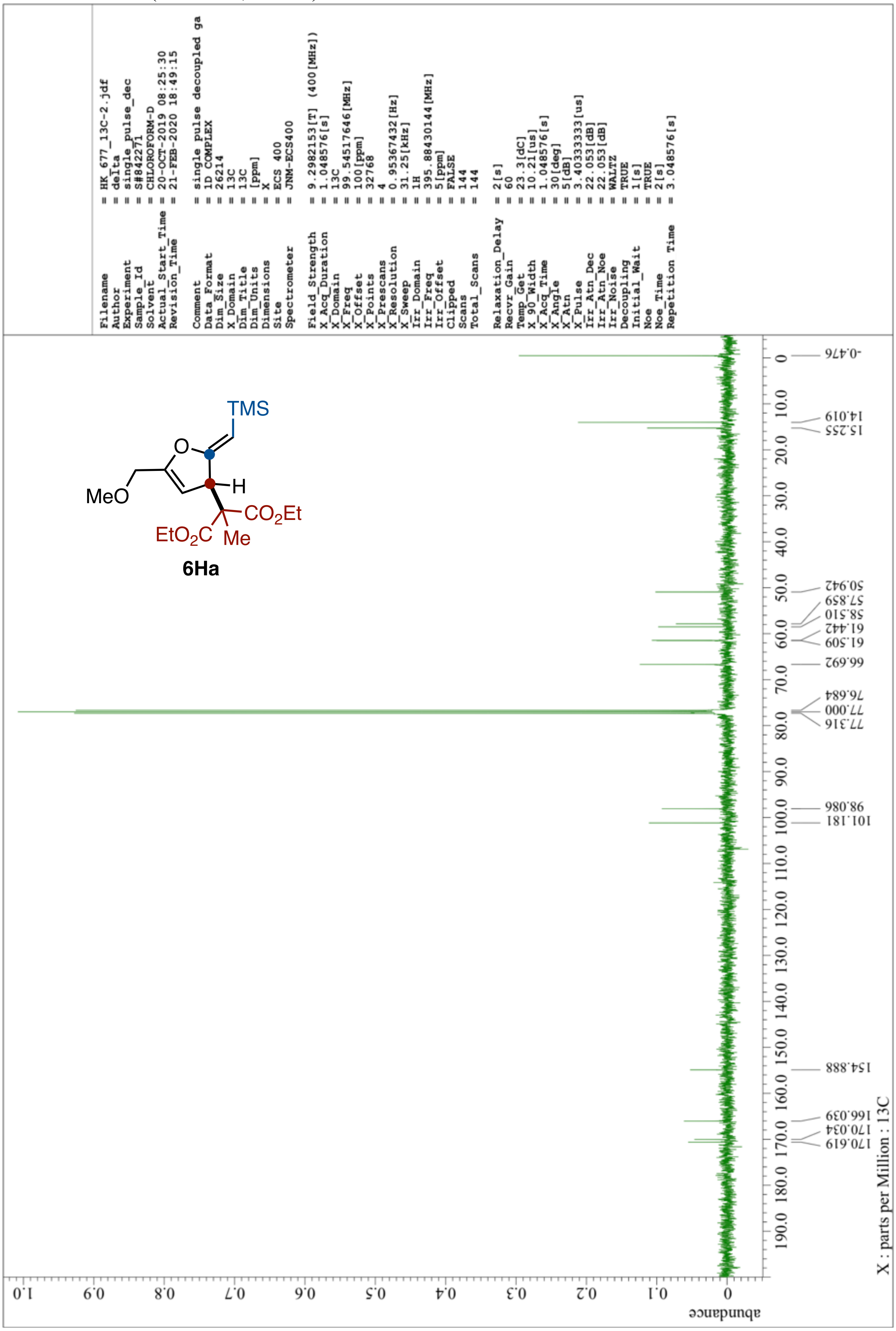

^{1}H NMR of **6Ia** (400 MHz, $CDCl_3$)

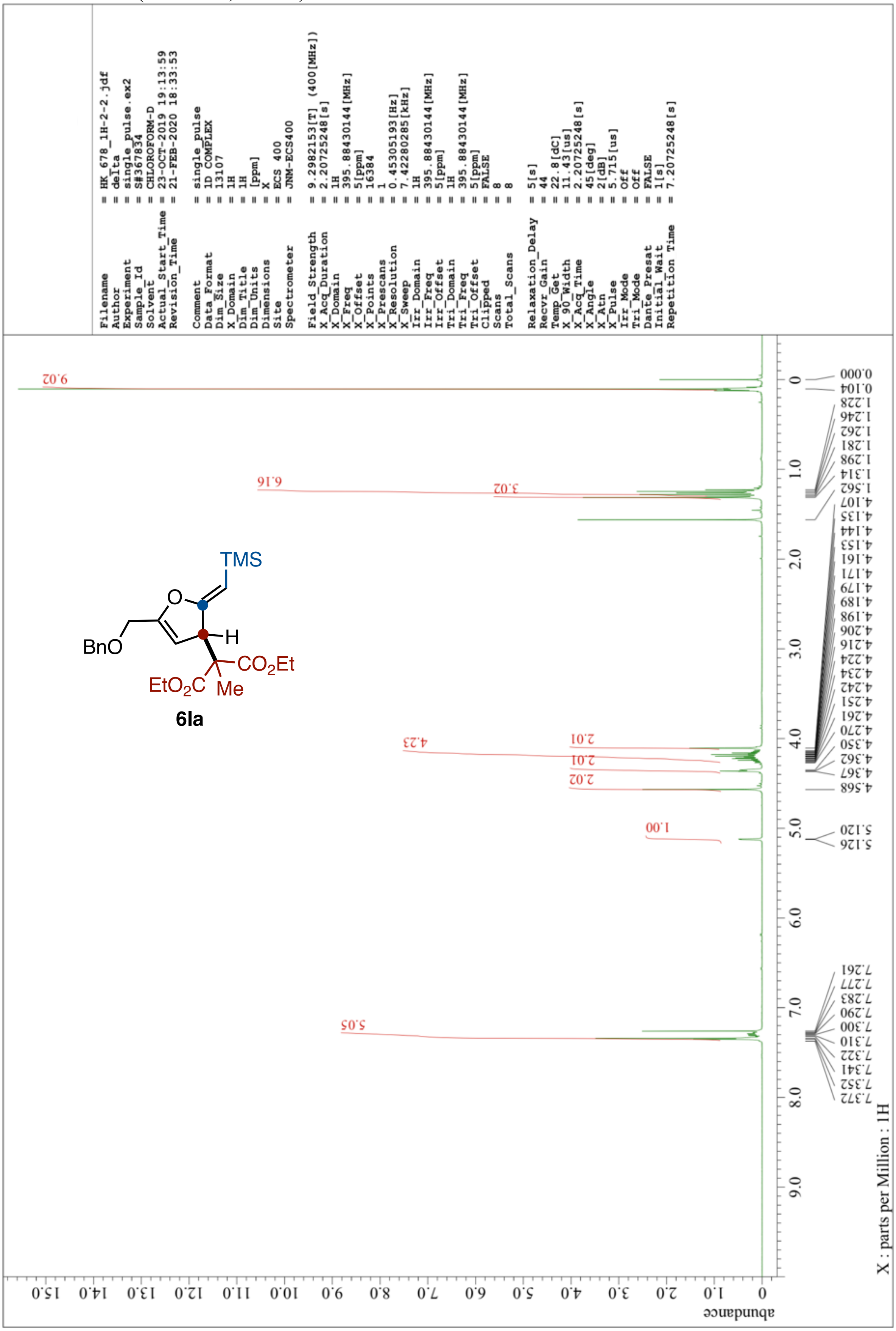

^{13}C NMR of **6Ia** (101 MHz, $CDCl_3$)

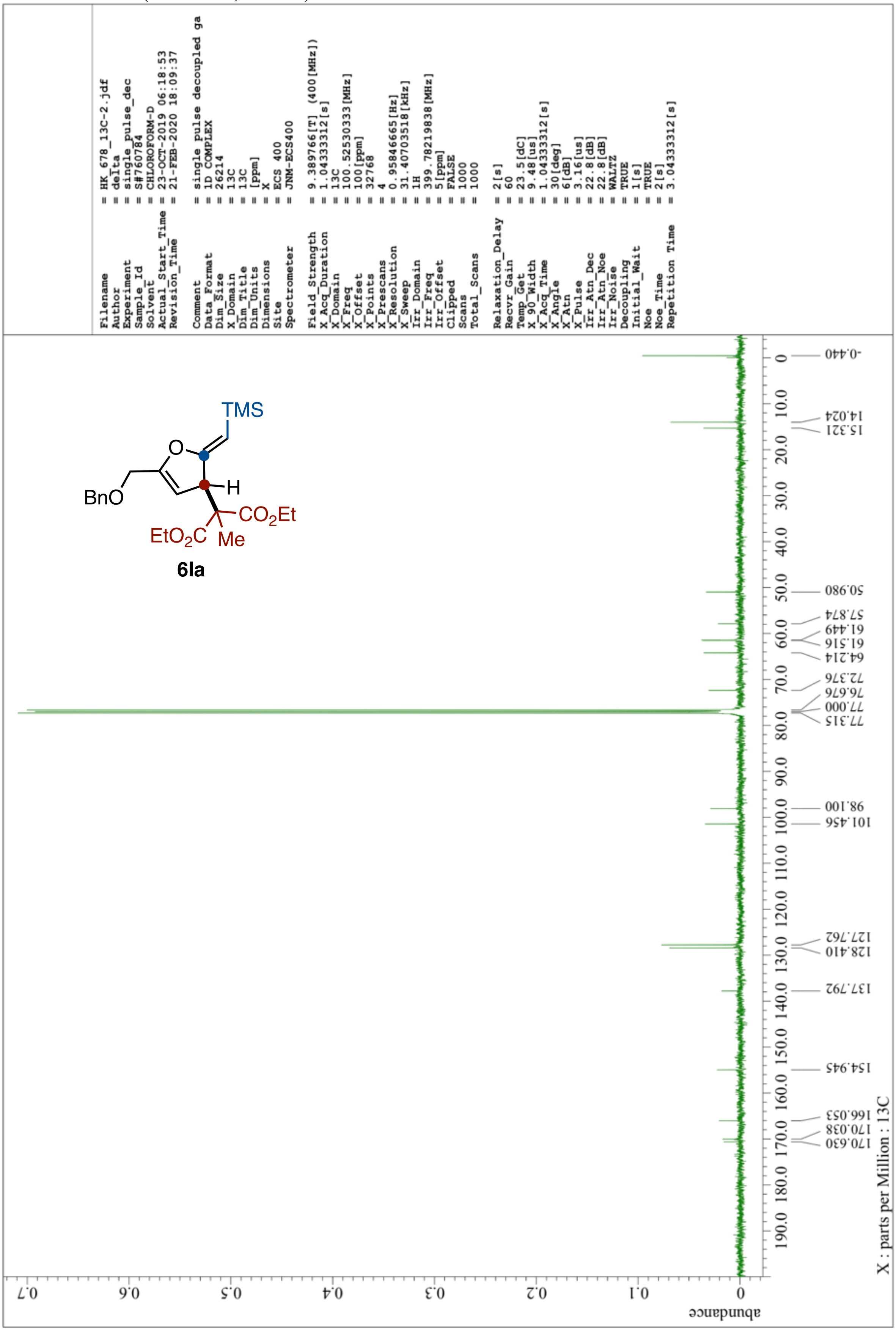

^{1}H NMR of **6Ja** (400 MHz, $CDCl_3$)

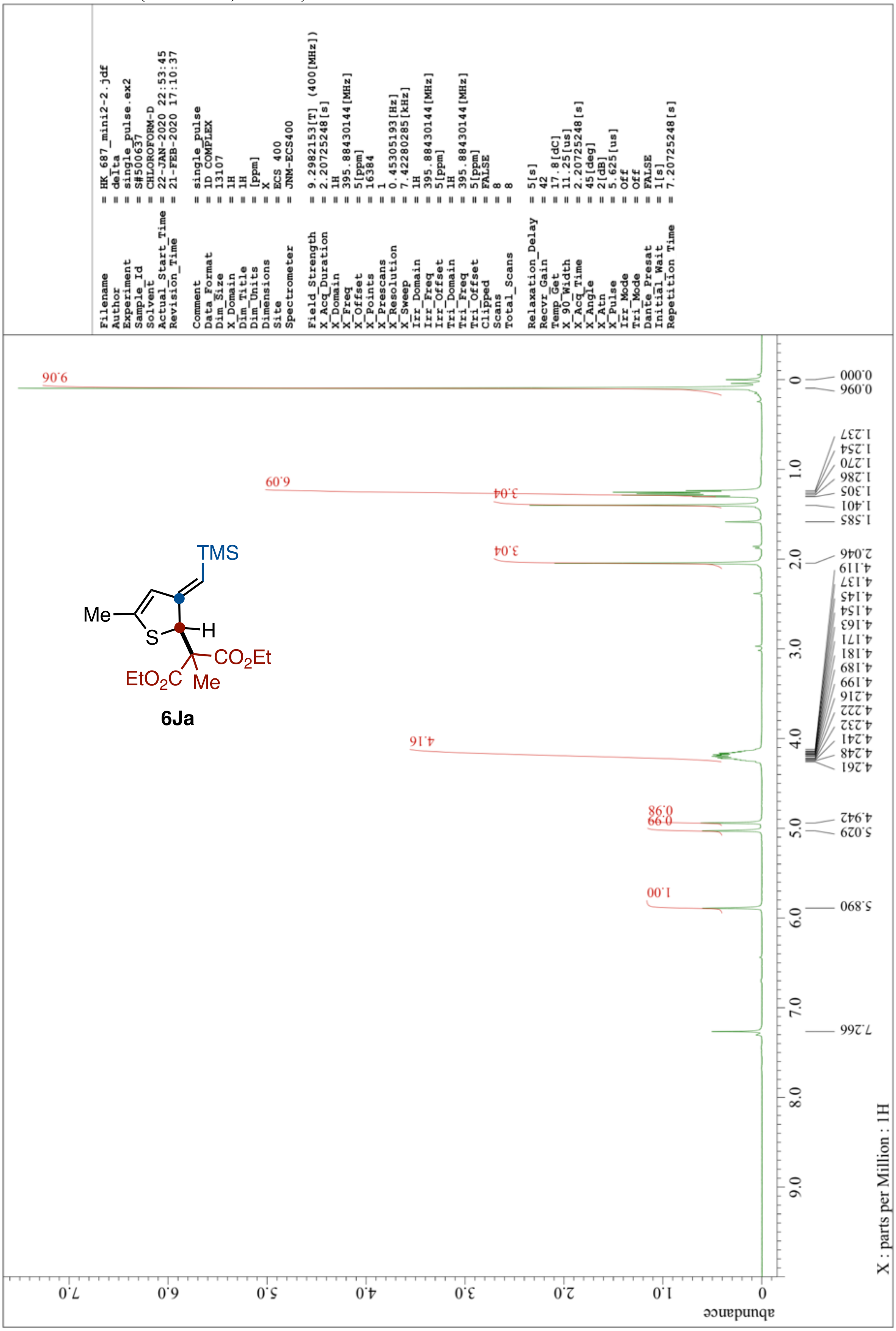

^{13}C NMR of **6Ja** (101 MHz, $CDCl_3$)

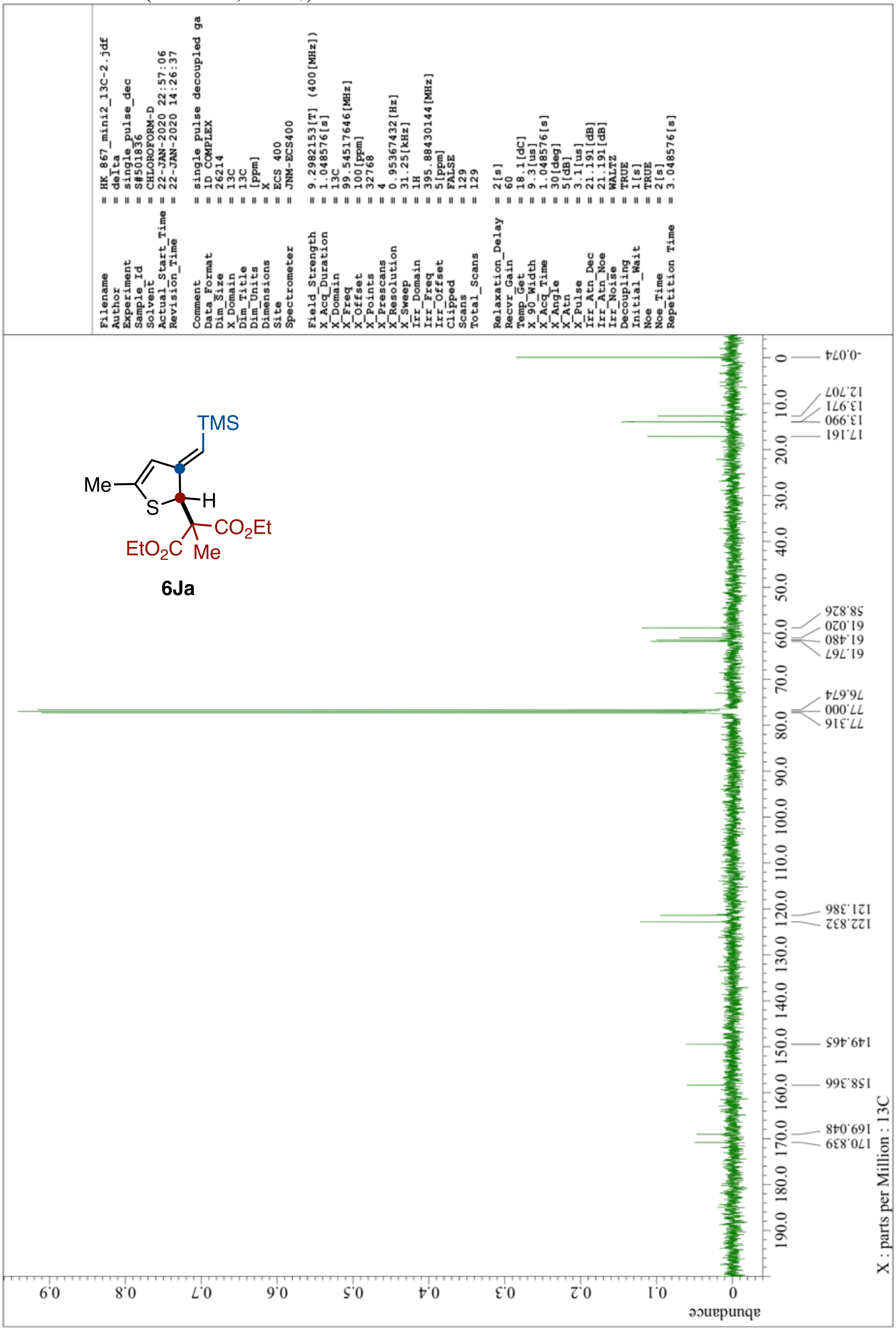

^{1}H NMR of **8Aa** (400 MHz, $CDCl_3$)

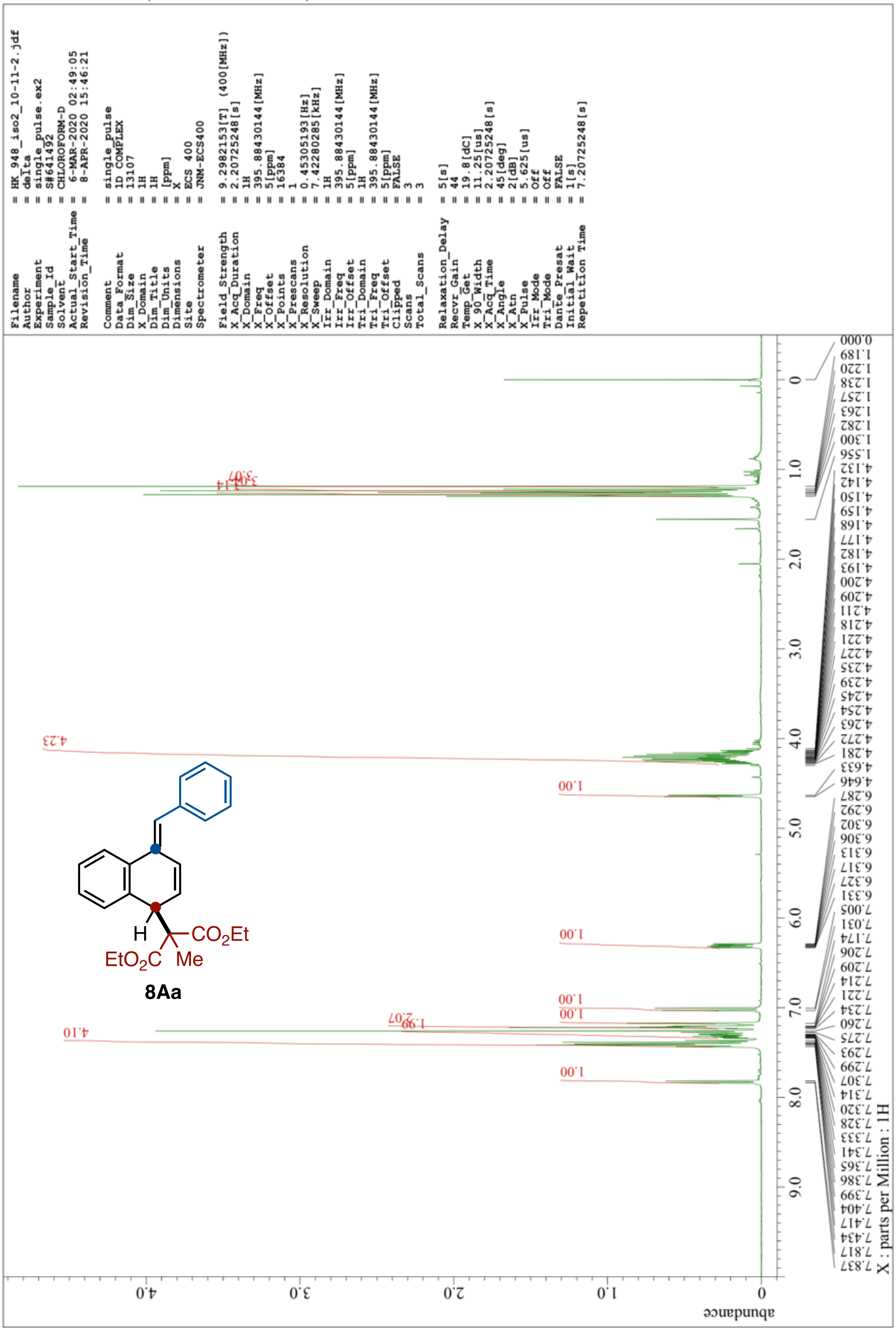

^{13}C NMR of **8Aa** (101 MHz, $CDCl_3$)

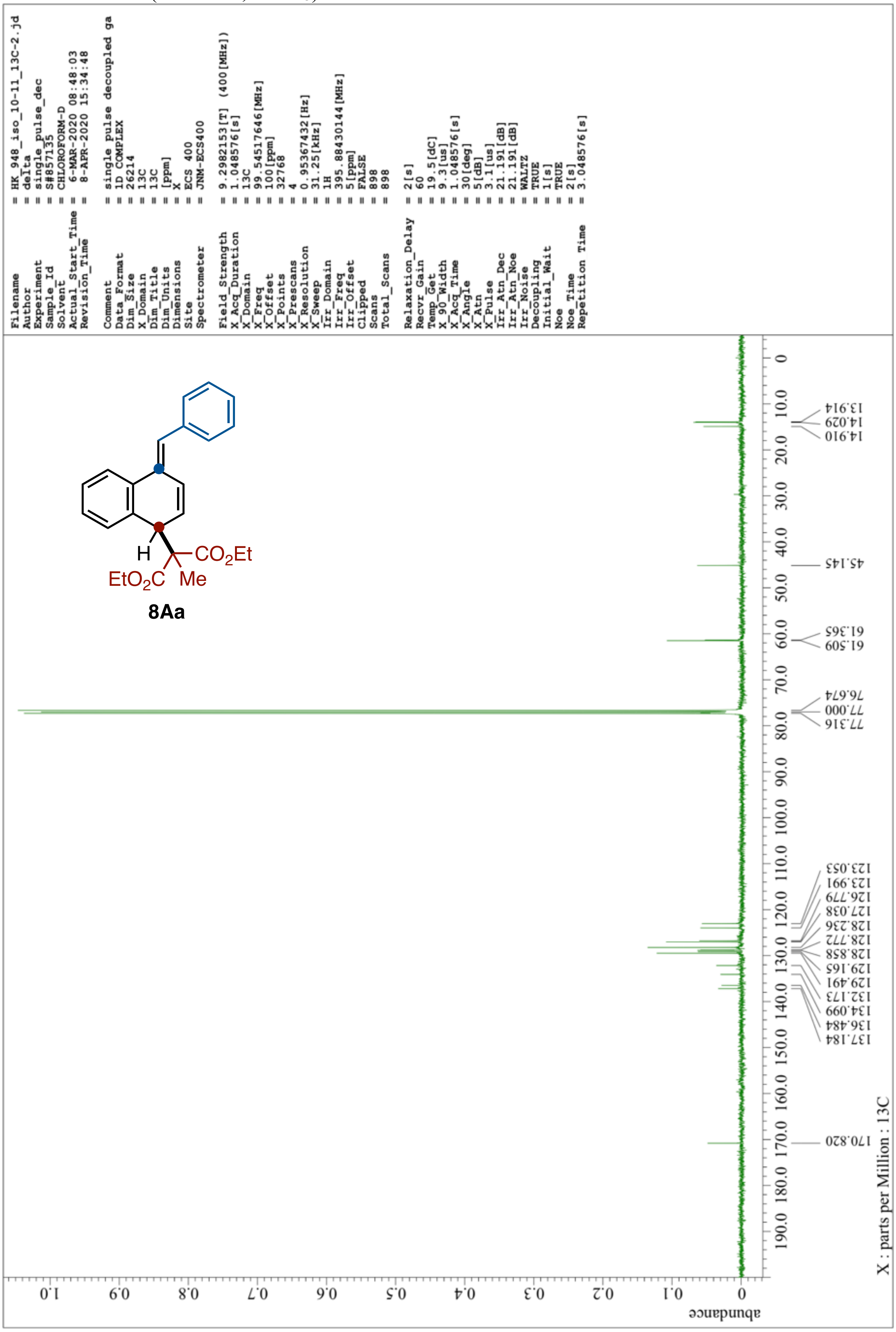

^{1}H NMR of **8Ab** (400 MHz, $CDCl_3$)

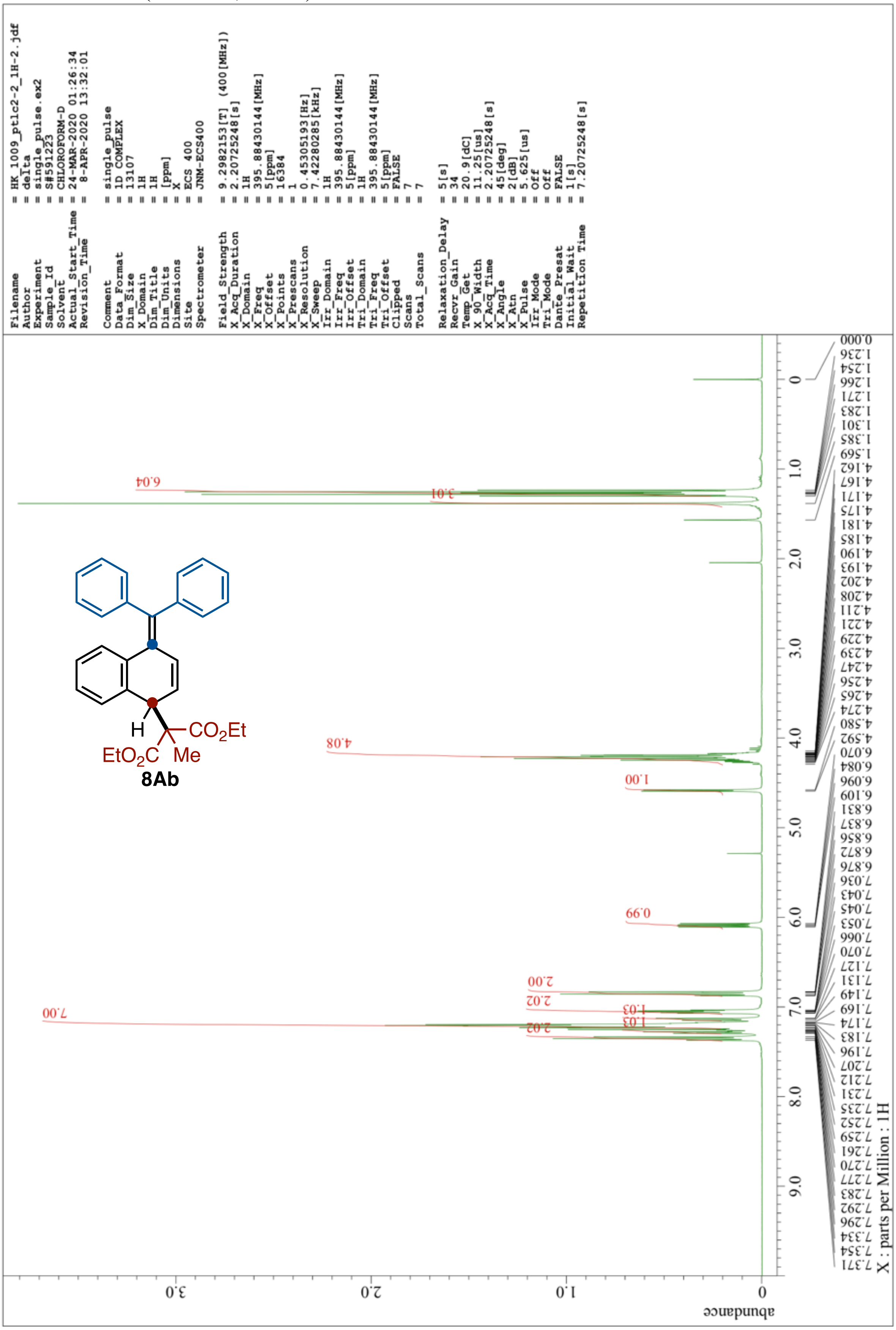

^{13}C NMR of **8Ab** (101 MHz, $CDCl_3$)

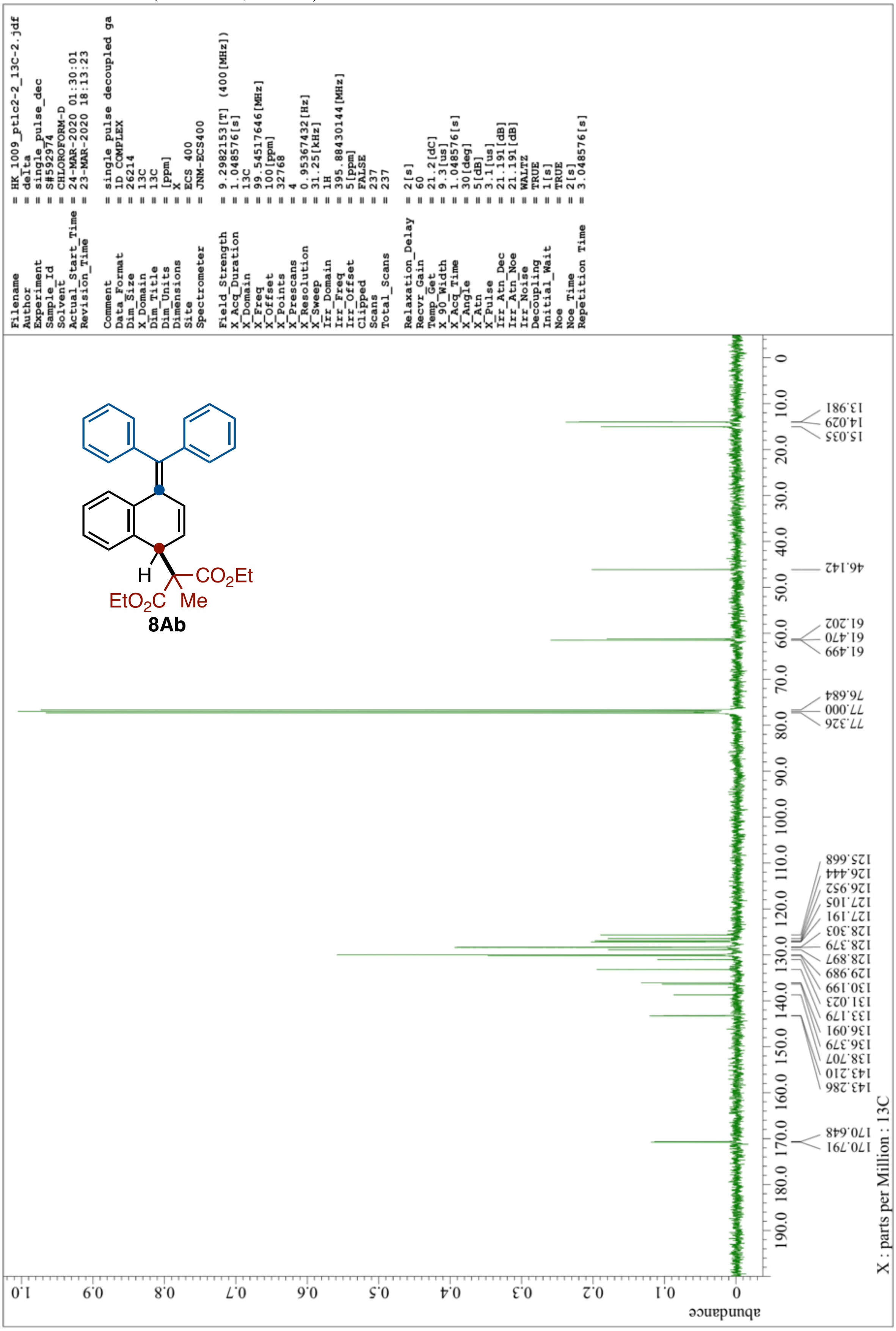

^{1}H NMR of **9Ac** (400 MHz, $CDCl_3$)

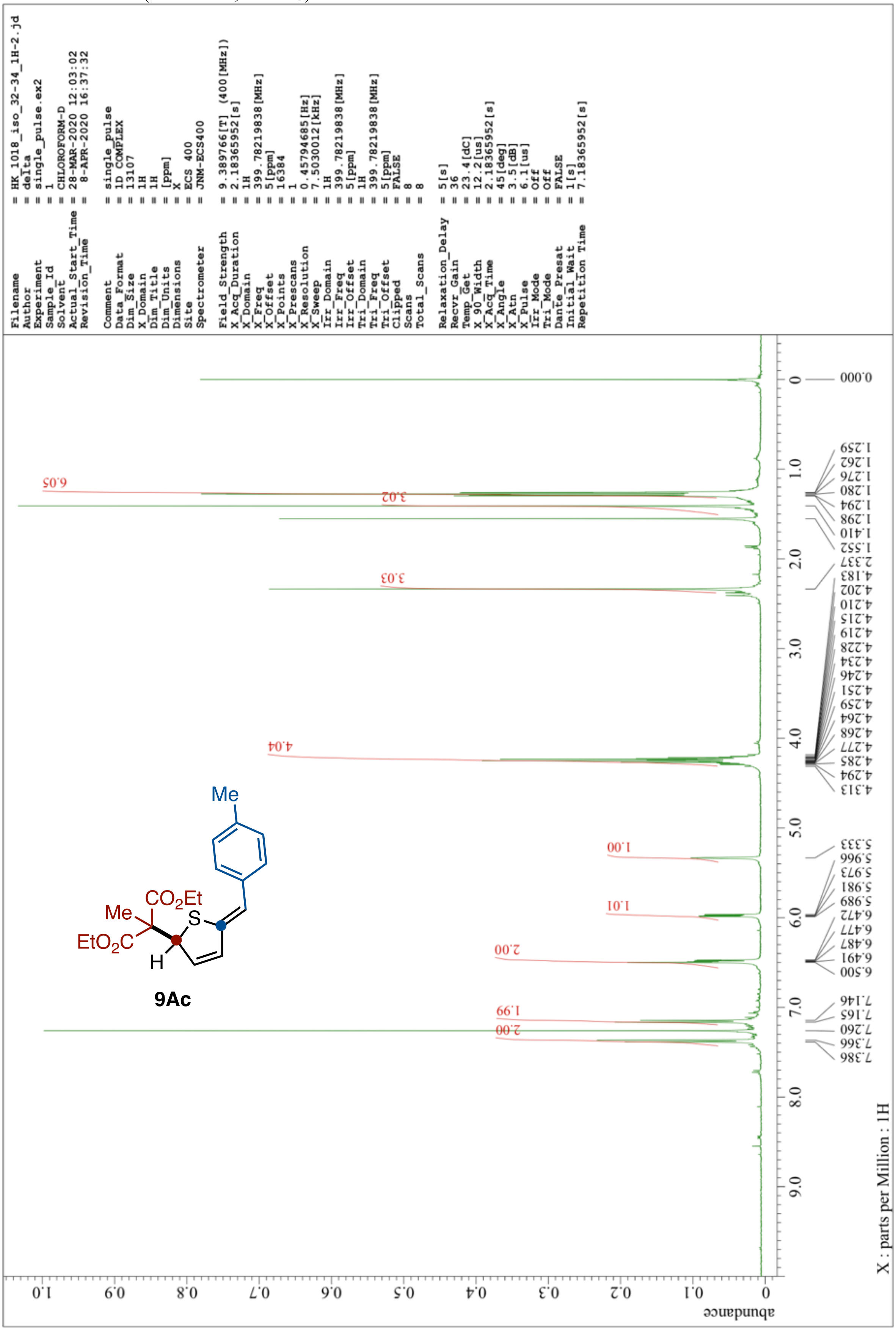

^{13}C NMR of **9Ac** (101 MHz, $CDCl_3$)

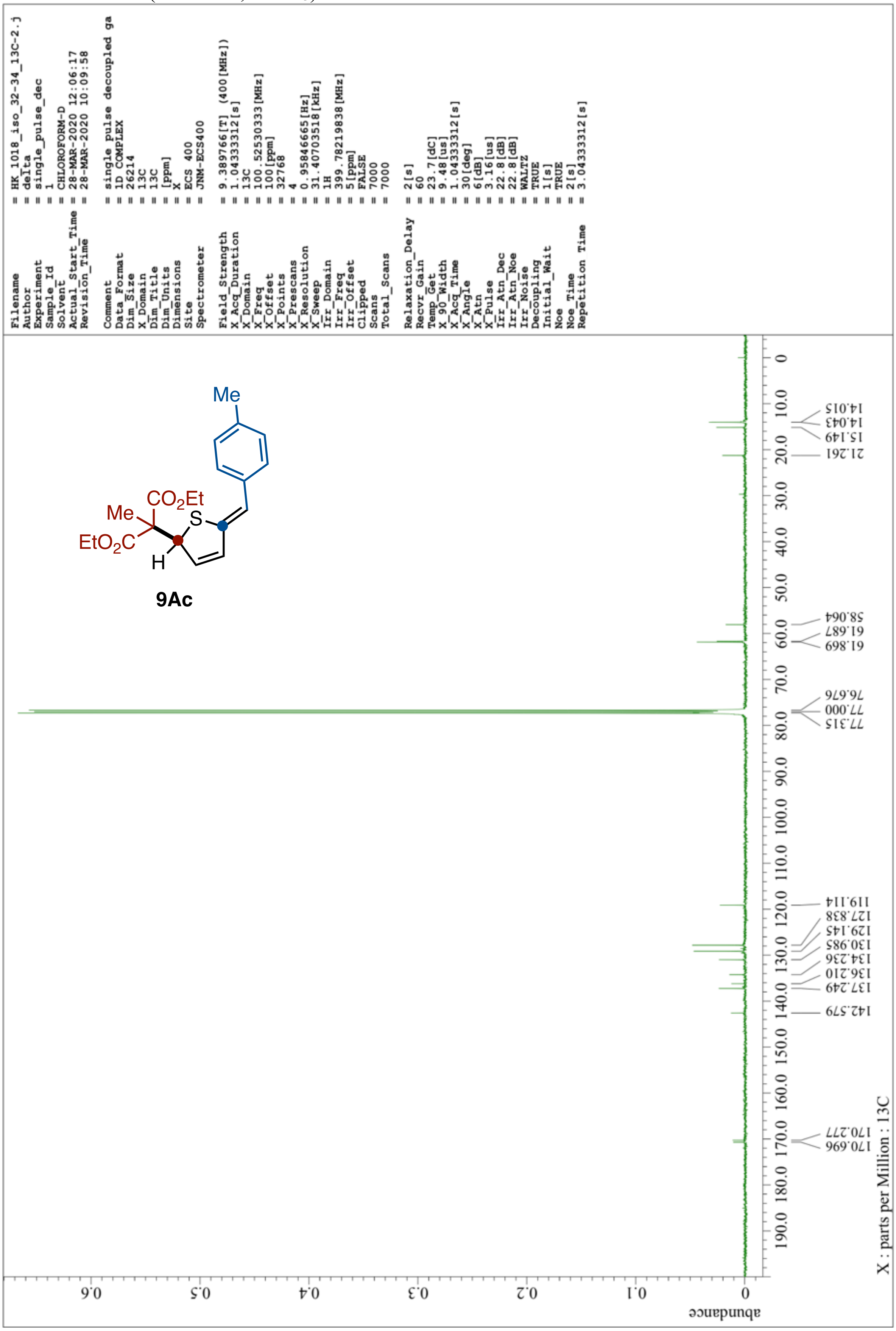

^{1}H NMR of **9Ad** (400 MHz, $CDCl_3$)

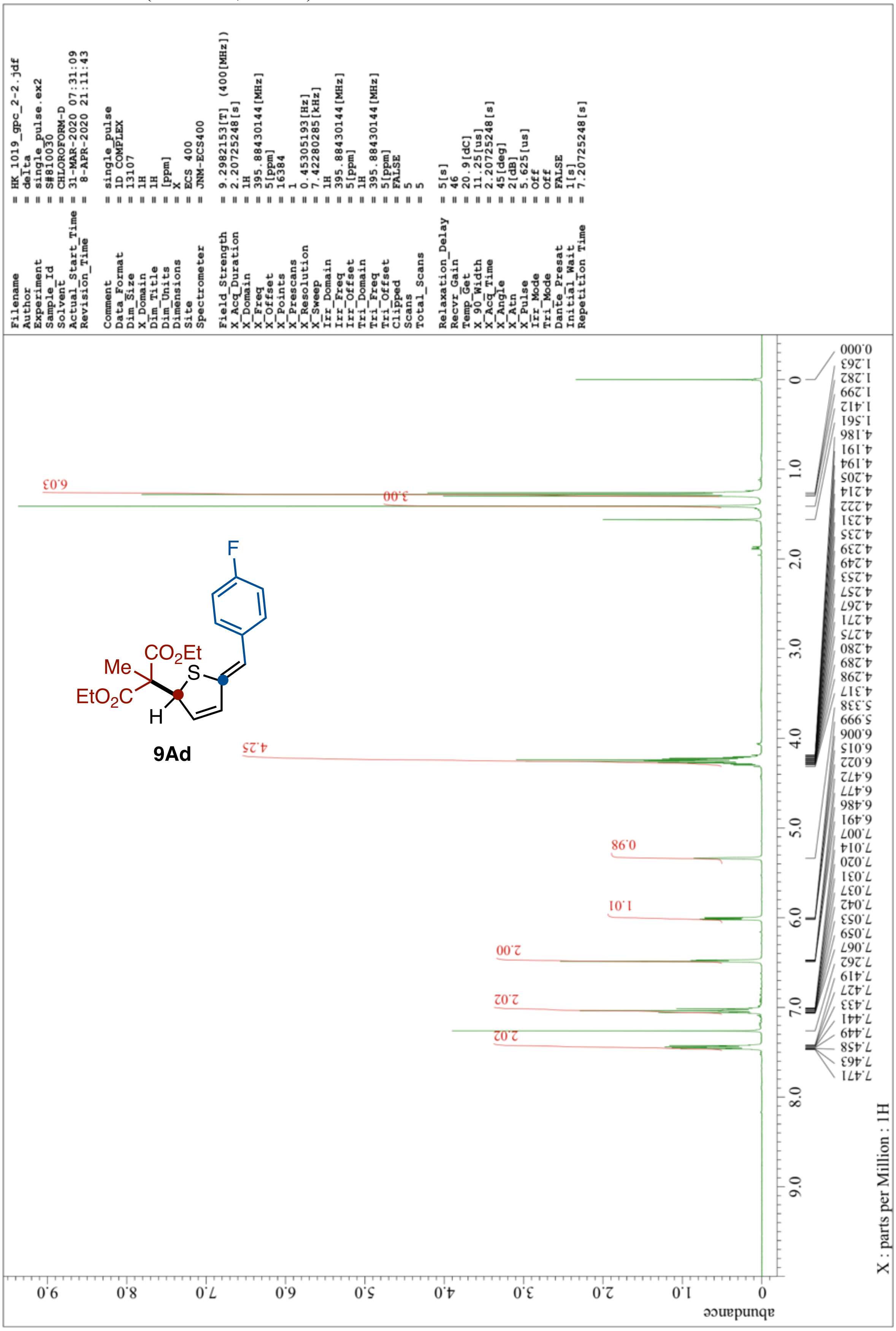

^{13}C NMR of **9Ad** (101 MHz, $CDCl_3$)

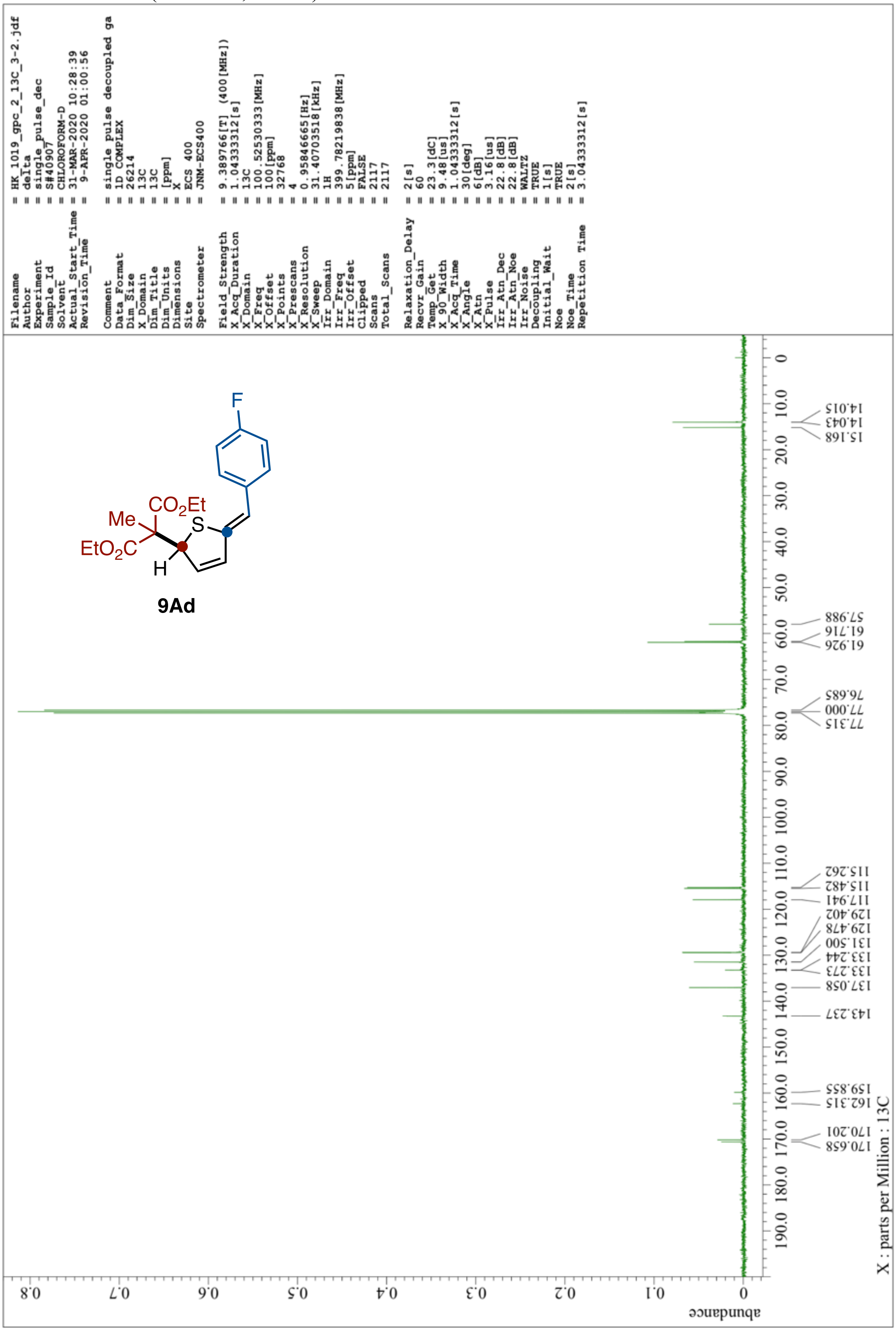

^{1}H NMR of **9Ab** (400 MHz, $CDCl_3$)

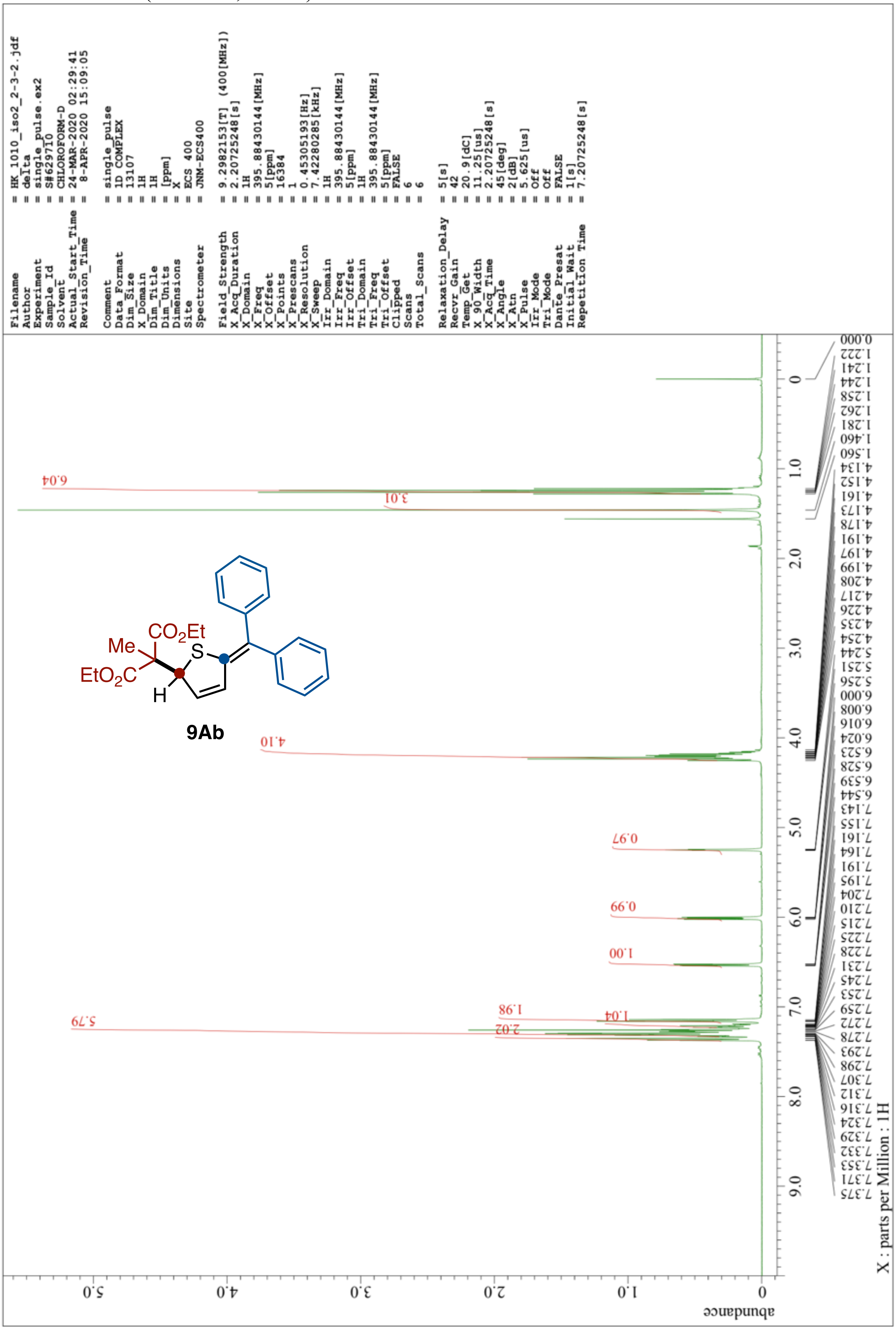

^{13}C NMR of **9Ab** (101 MHz, $CDCl_3$)

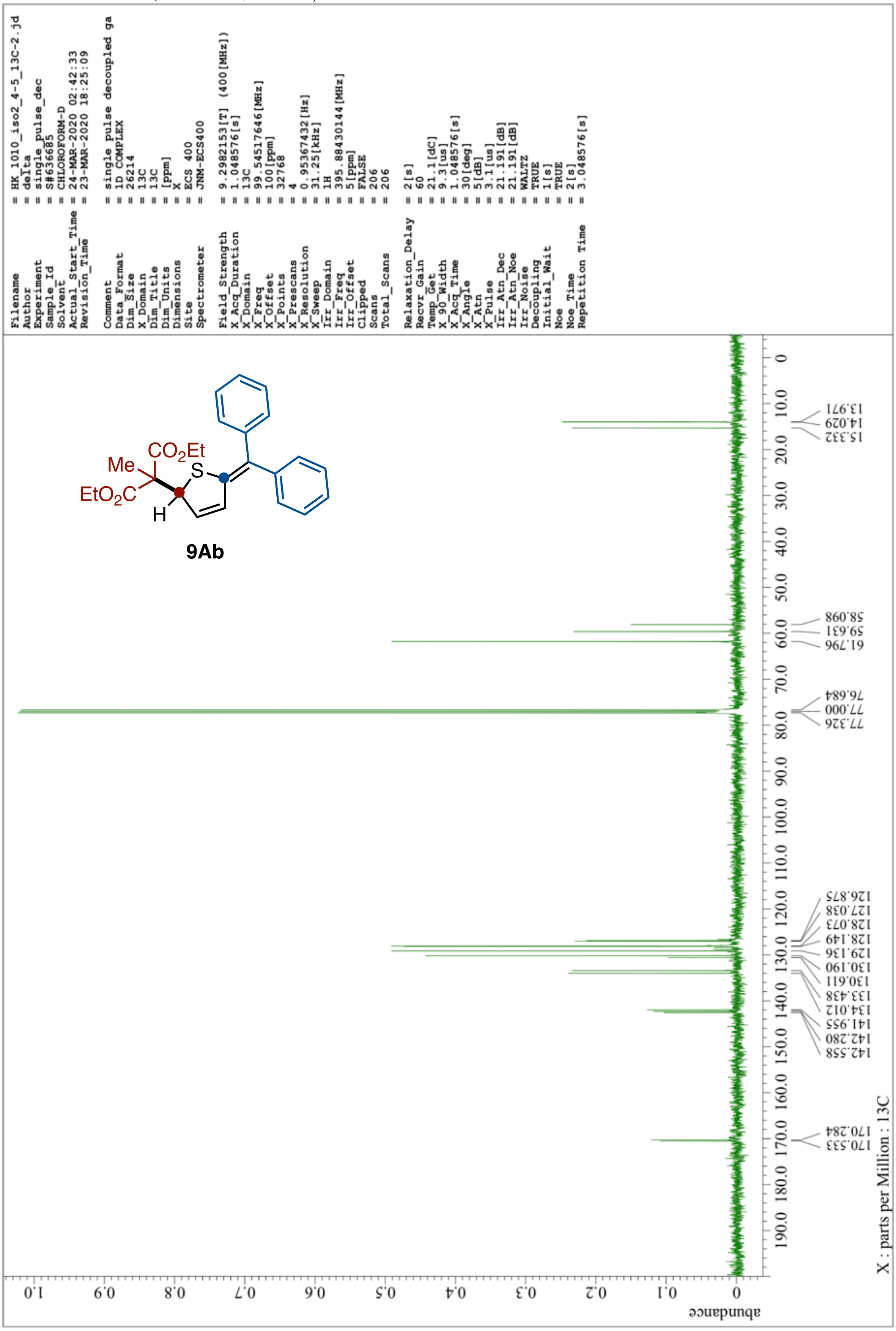

^{1}H NMR of **10** (400 MHz, $CDCl_3$)

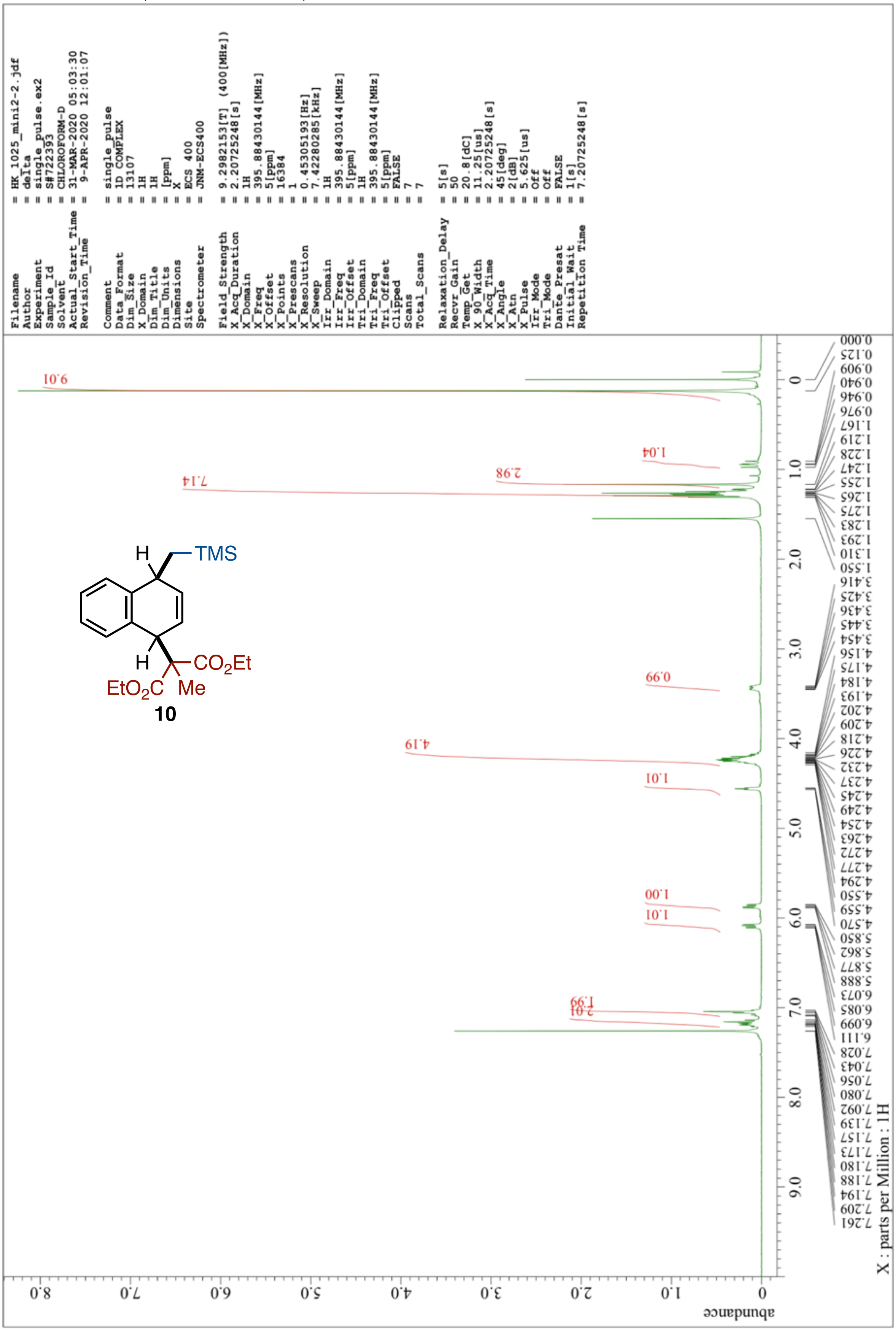

^{13}C NMR of **10** (101 MHz, $CDCl_3$)

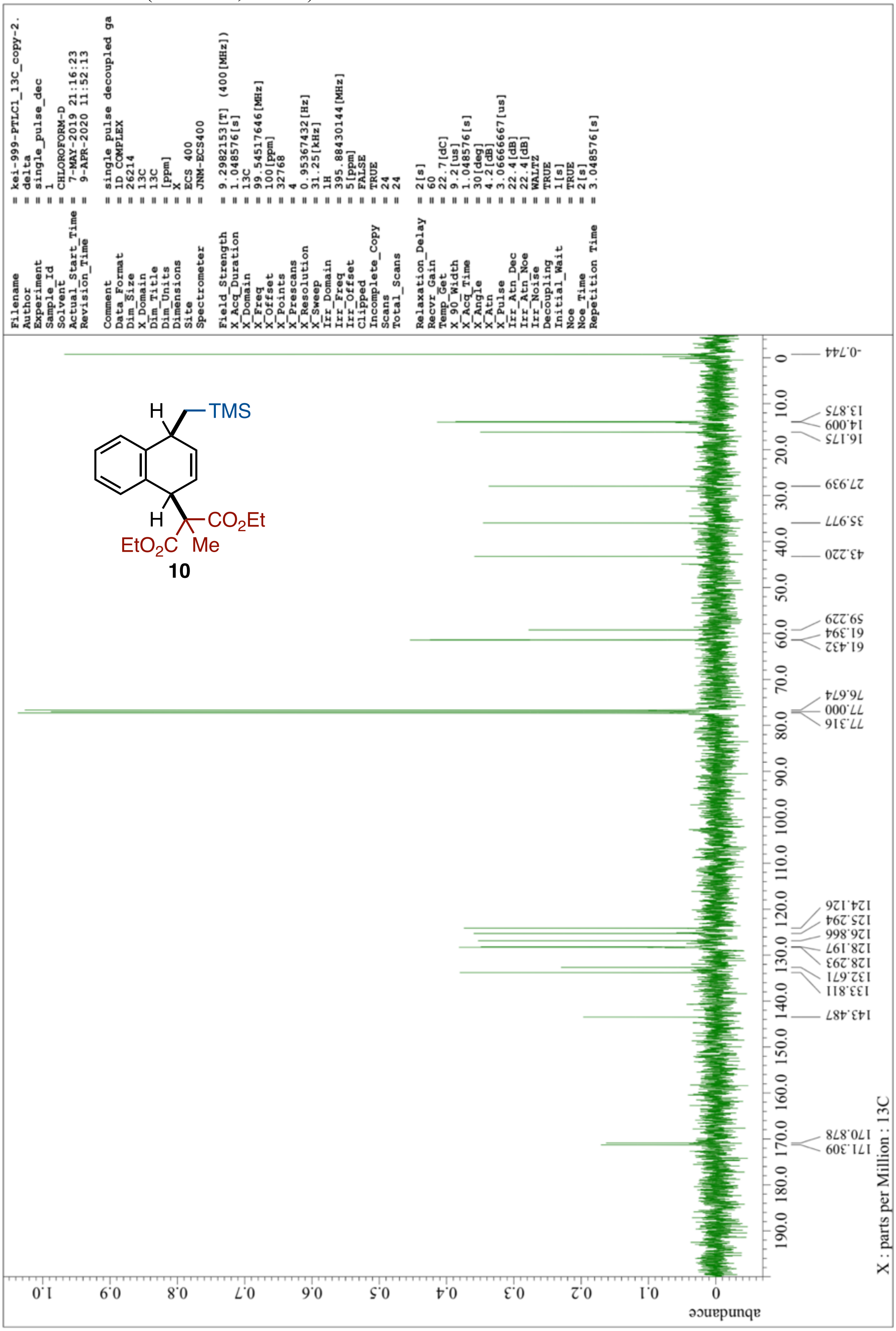

^{1}H NMR of **11** (400 MHz, $CDCl_3$)

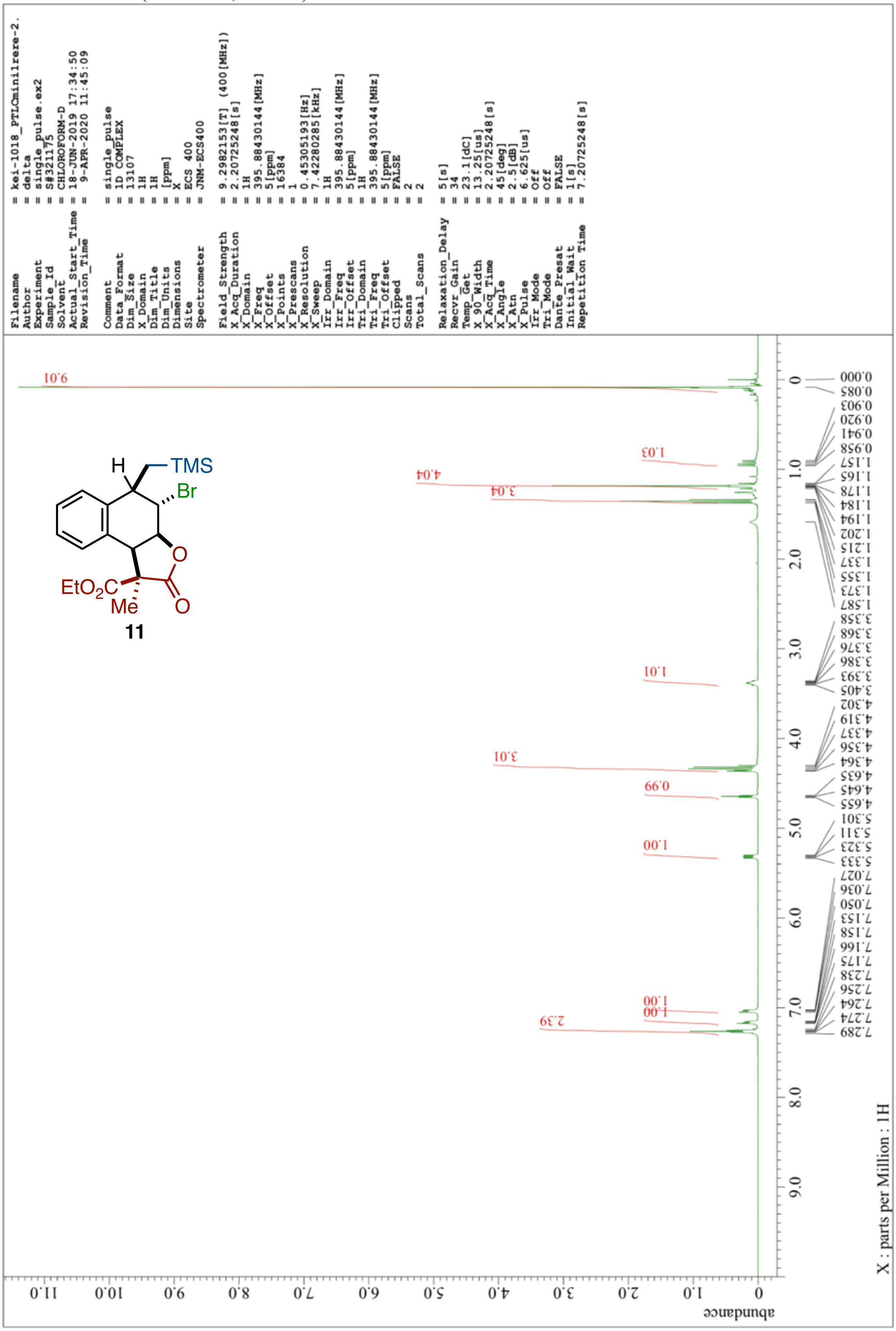

^{13}C NMR of **11** (101 MHz, $CDCl_3$)

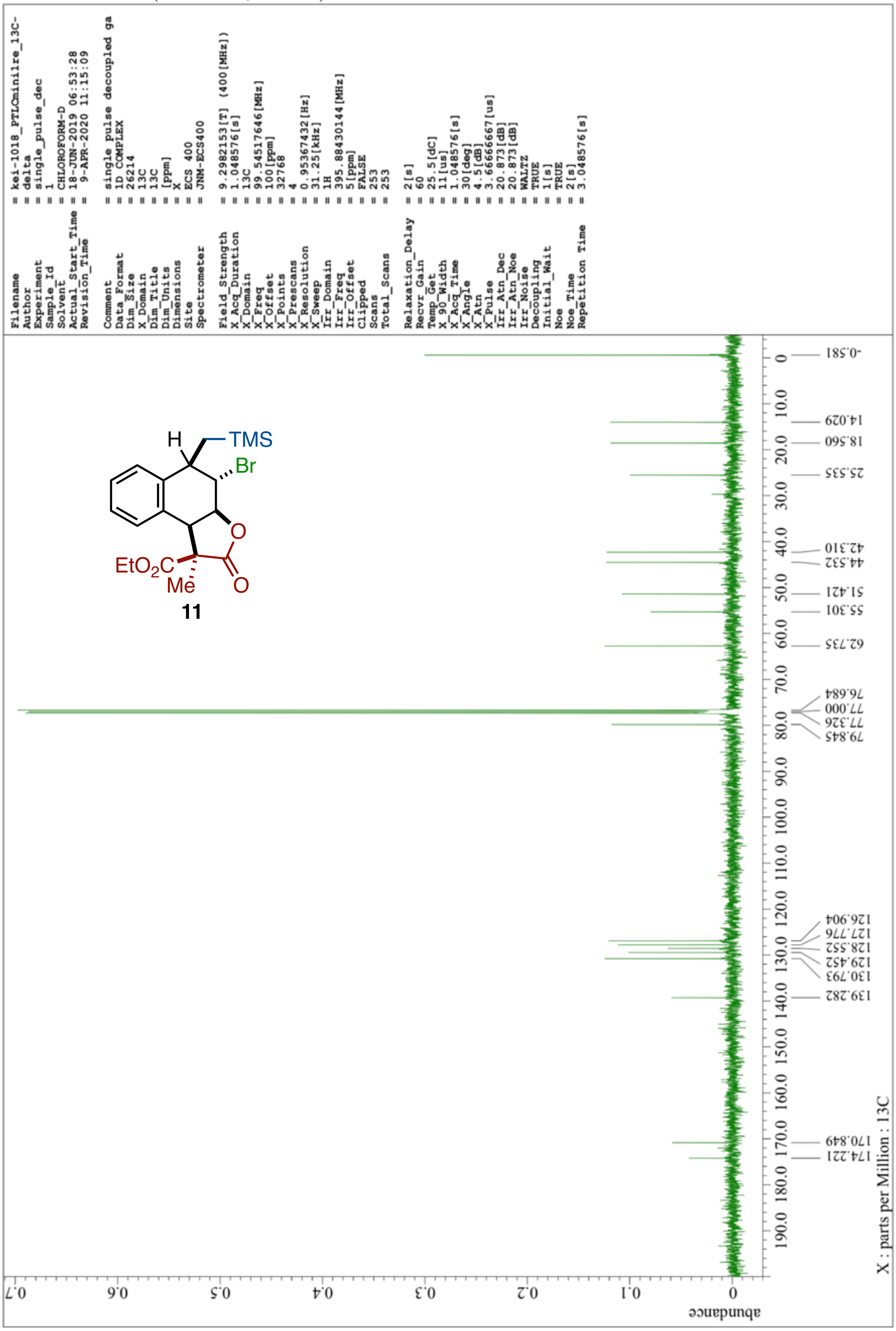

^{1}H NMR of **12** (400 MHz, $CDCl_3$)

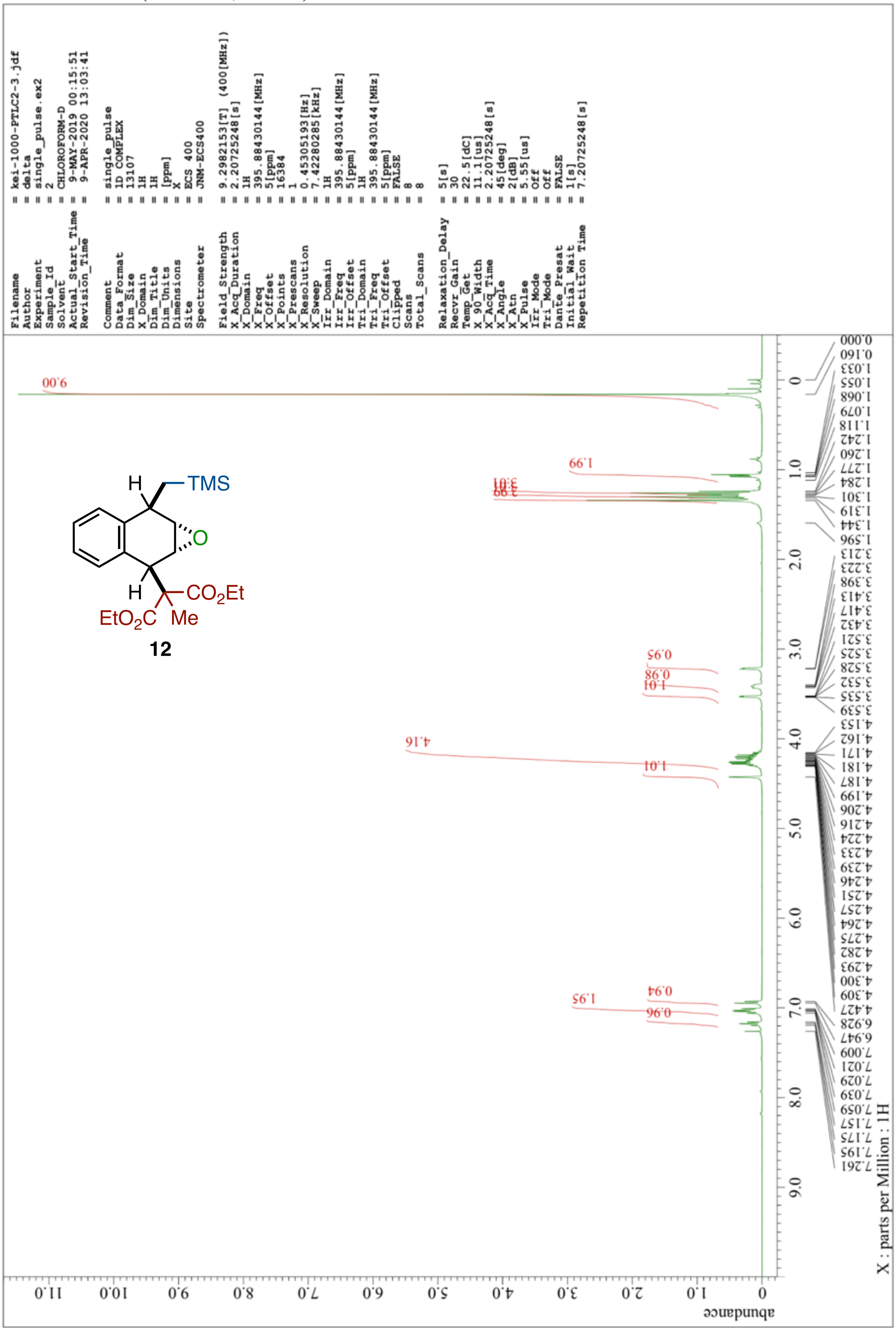

^{13}C NMR of **12** (101 MHz, $CDCl_3$)

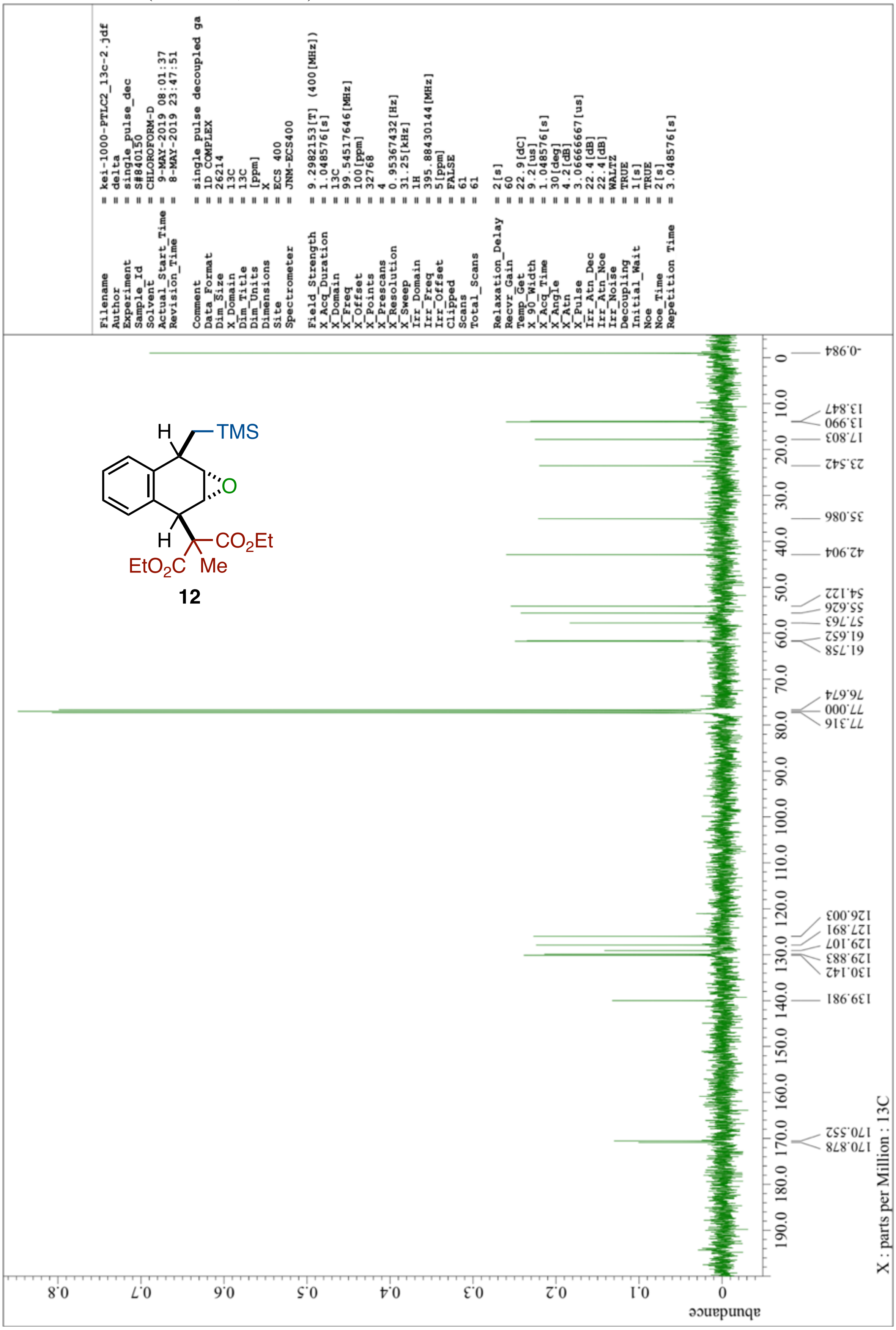

^{1}H NMR of **13** (400 MHz, $CDCl_3$)

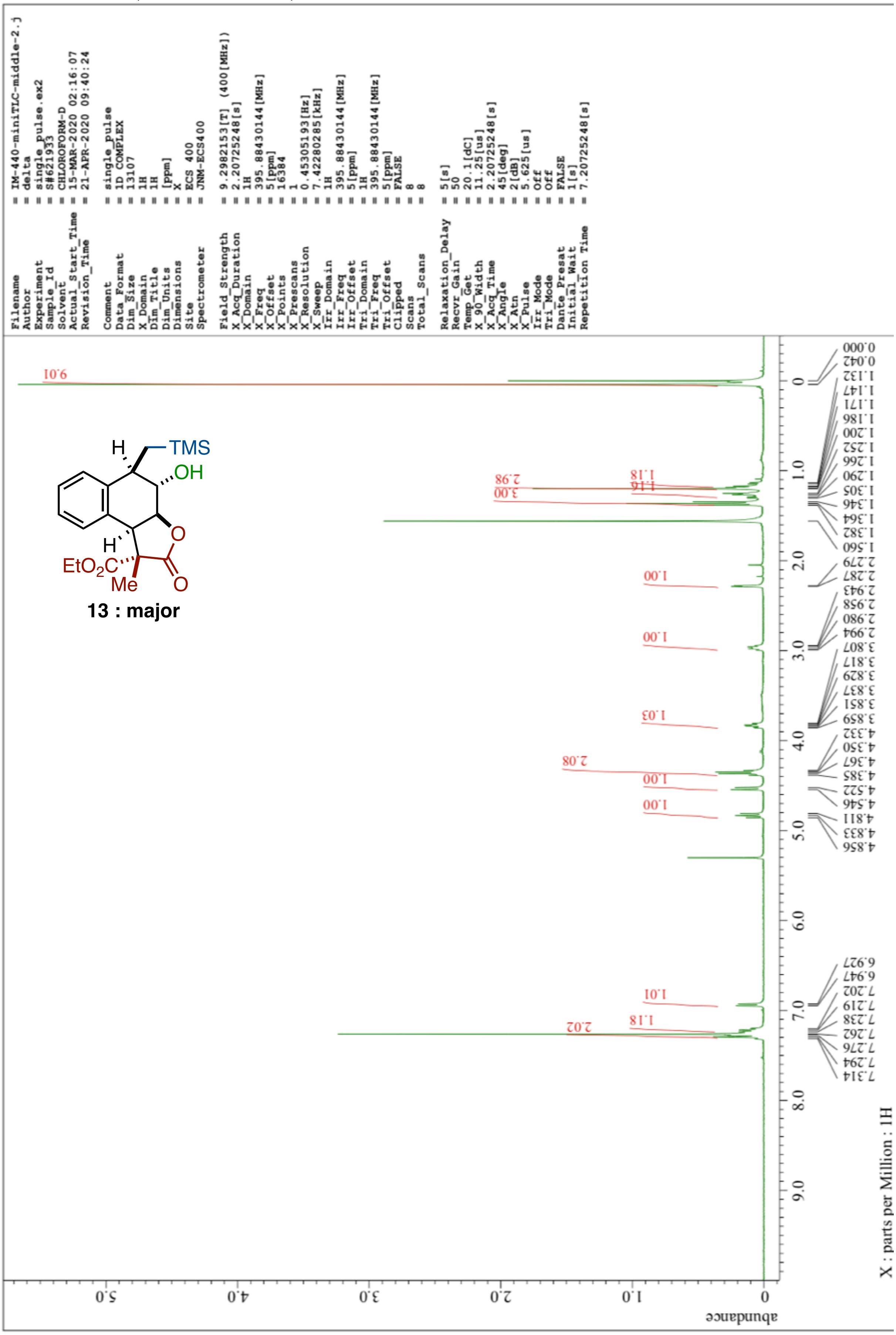

^{13}C NMR of **13** (101 MHz, $CDCl_3$)

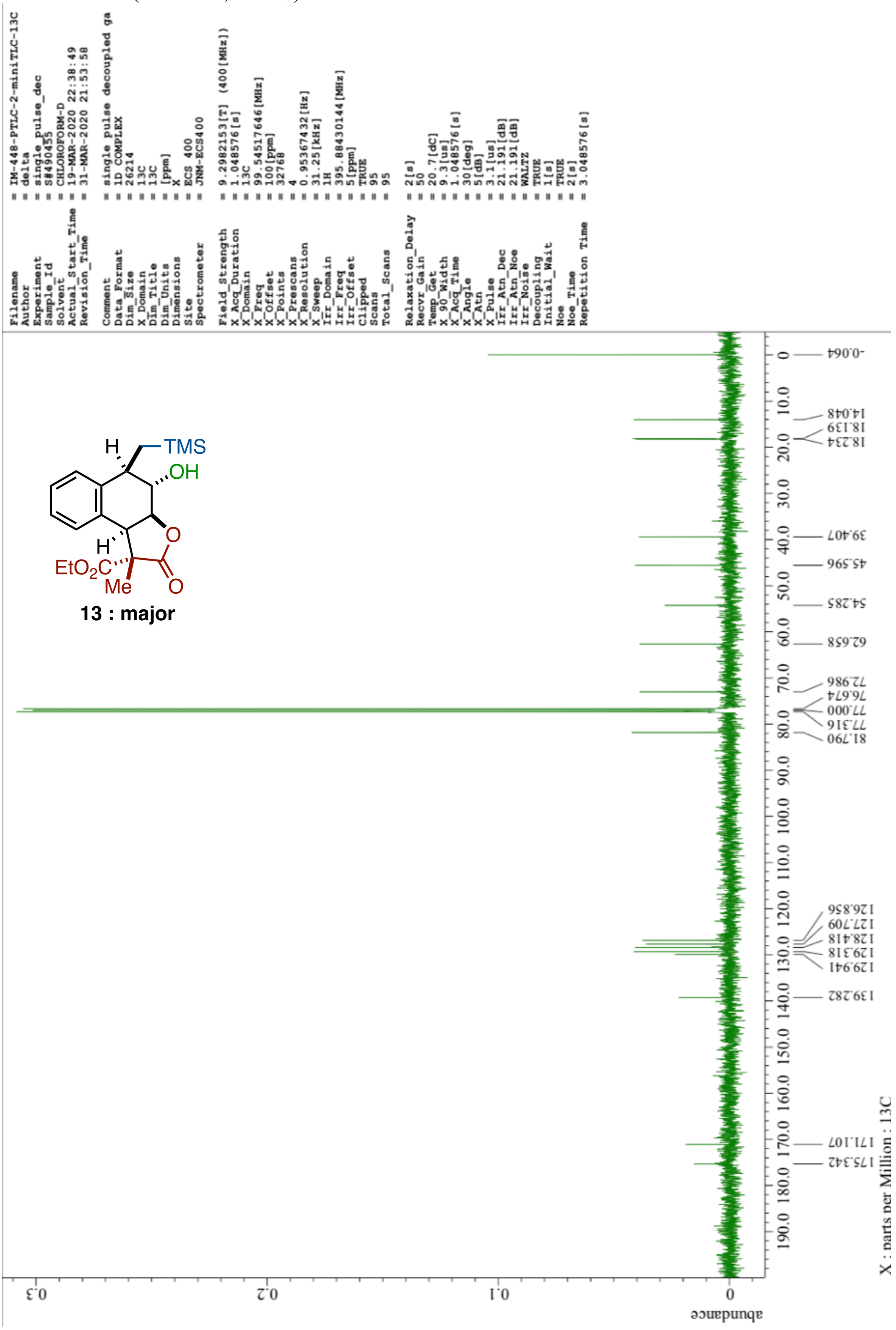

13 : major

^{1}H NMR of **13'** (400 MHz, $CDCl_3$)

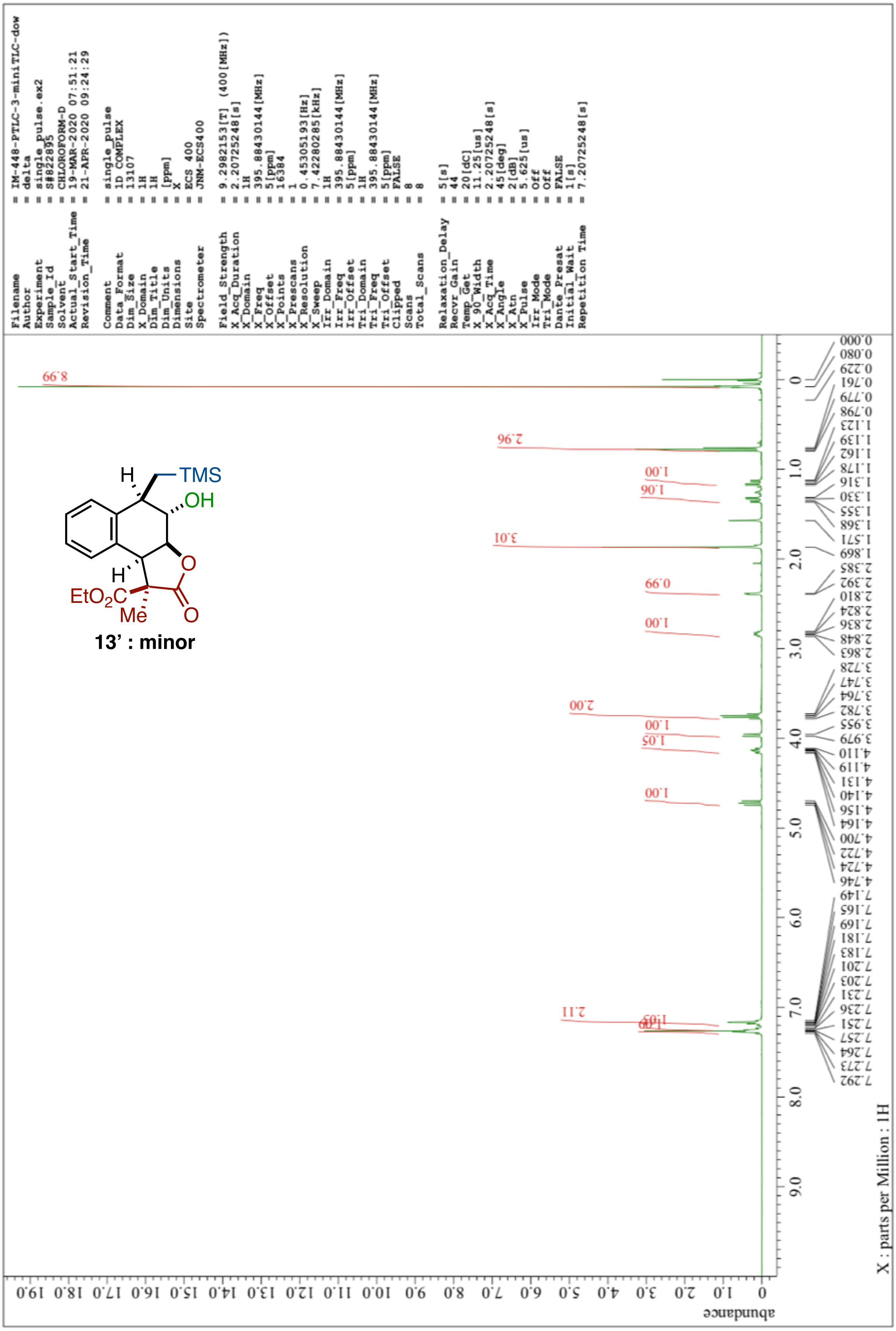

^{13}C NMR of **13’** (101 MHz, $CDCl_3$)

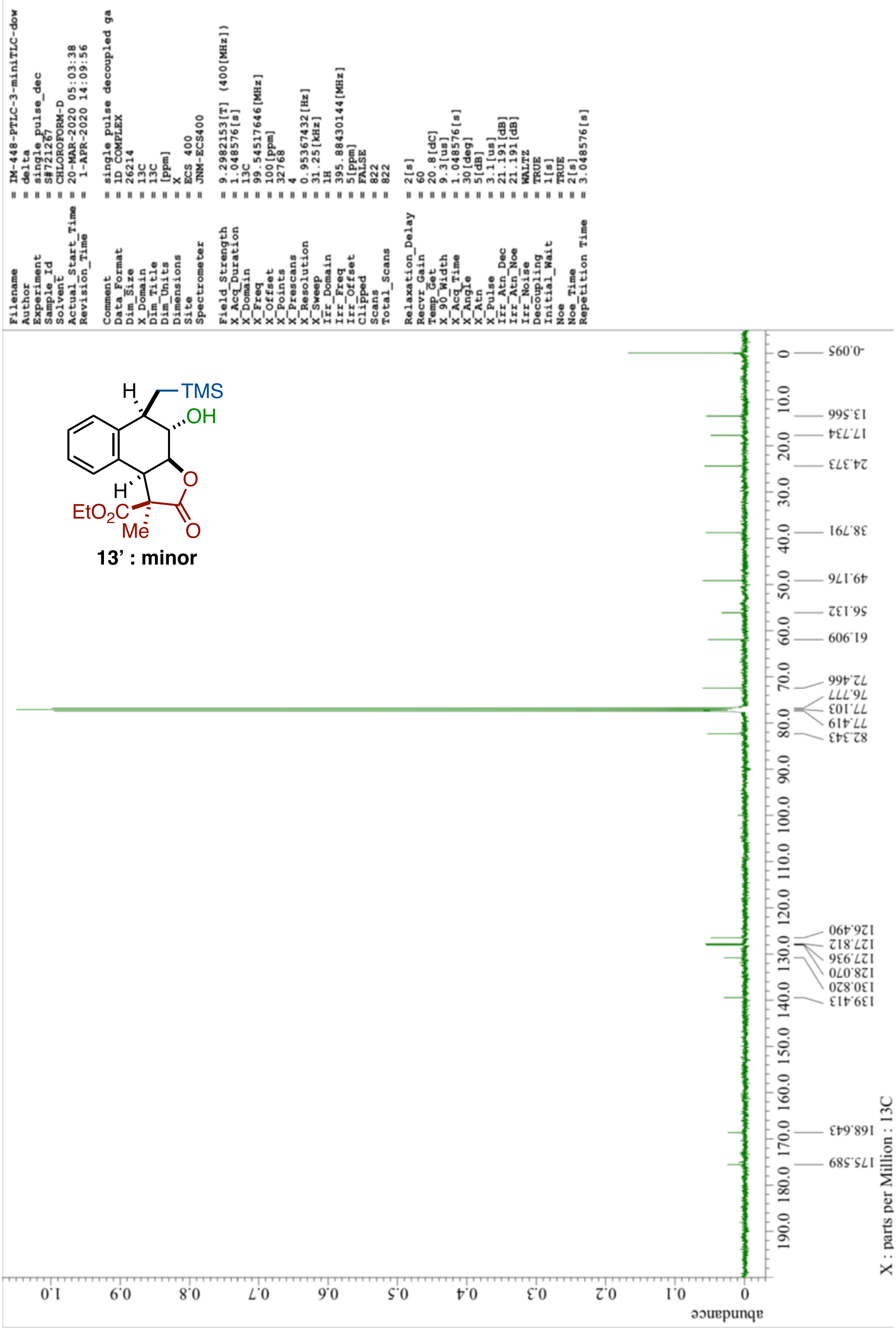

^{1}H NMR of **14** (400 MHz, $CDCl_3$)

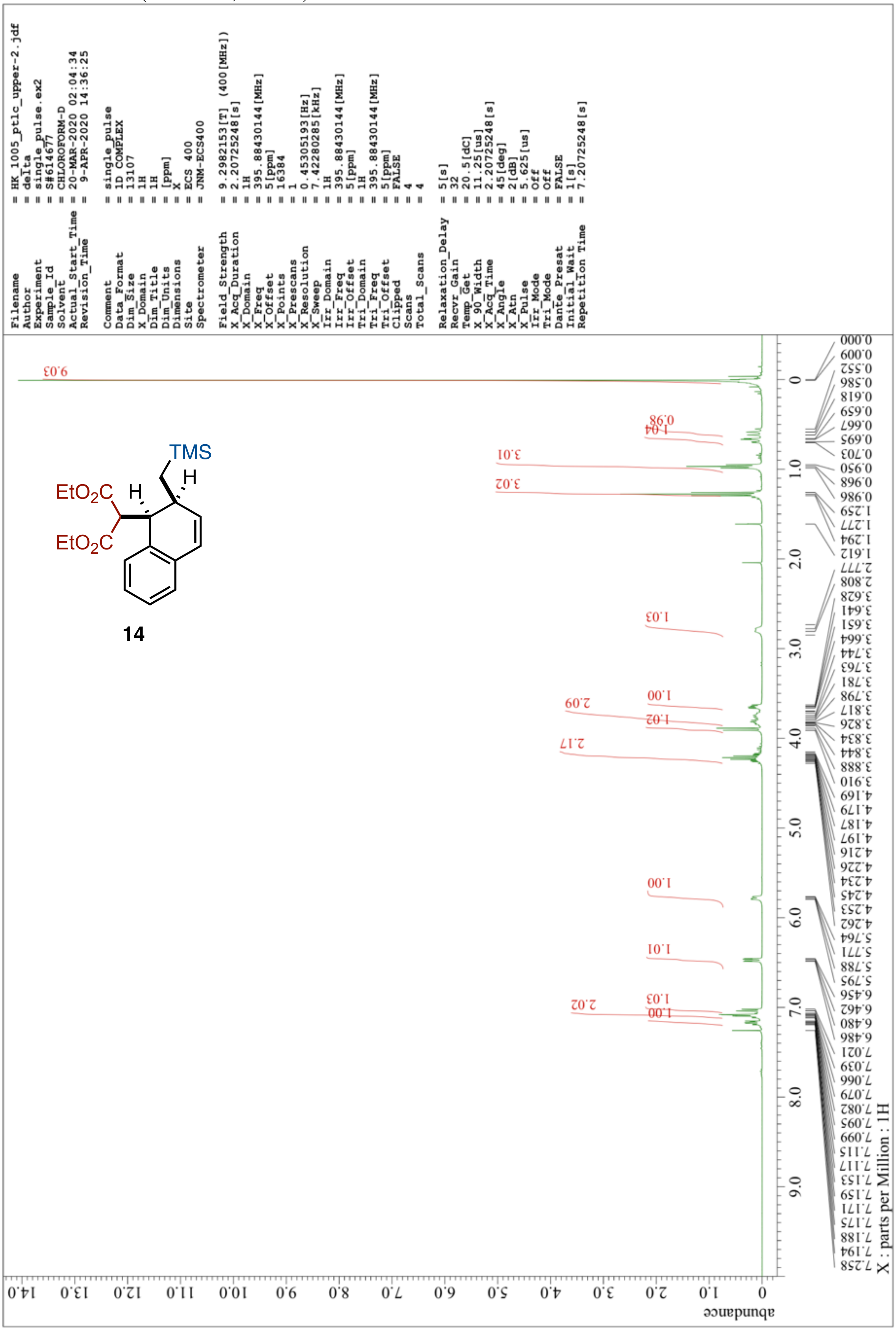

^{13}C NMR of **14** (101 MHz, $CDCl_3$)

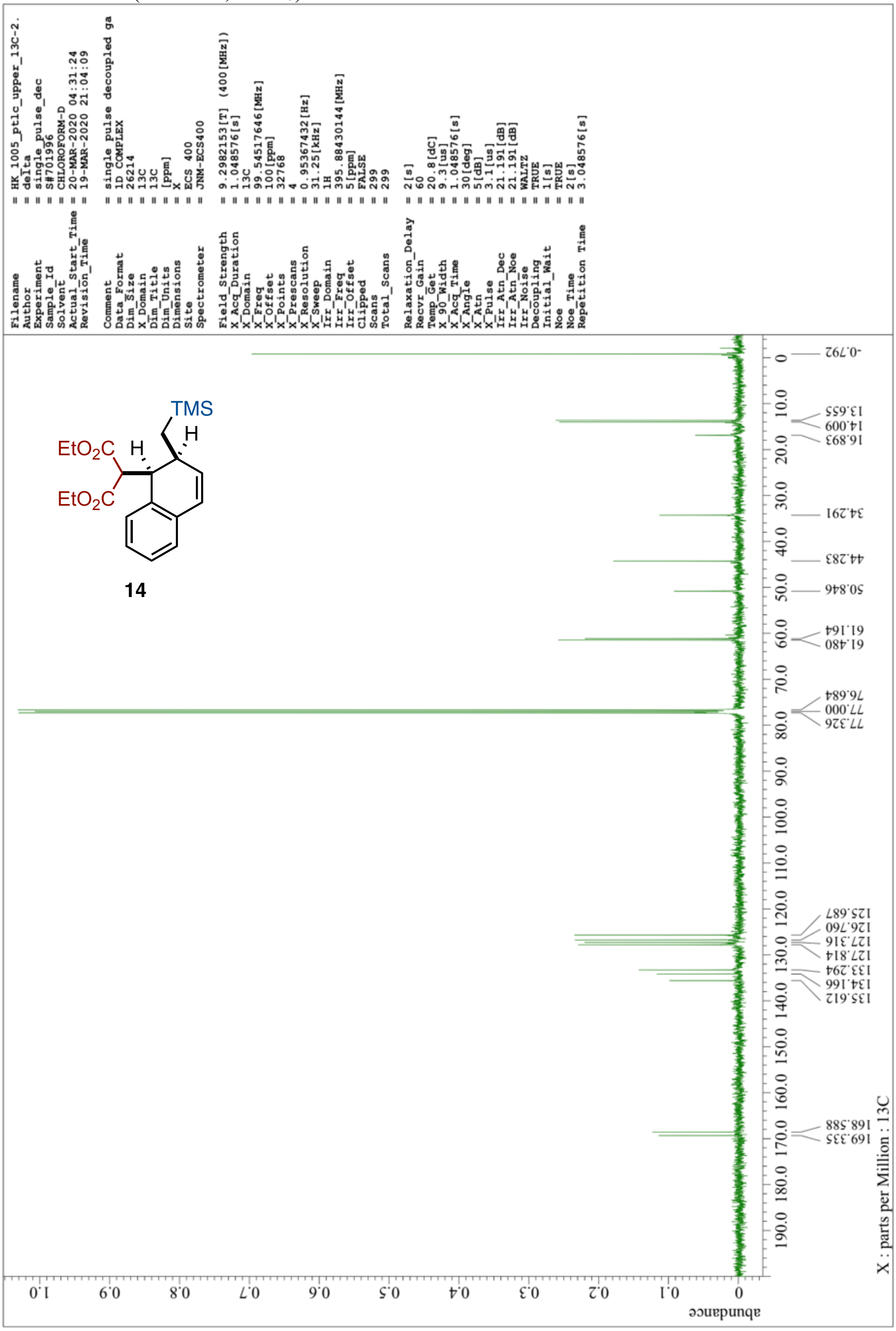

^{1}H NMR of **15** (400 MHz, $CDCl_3$)

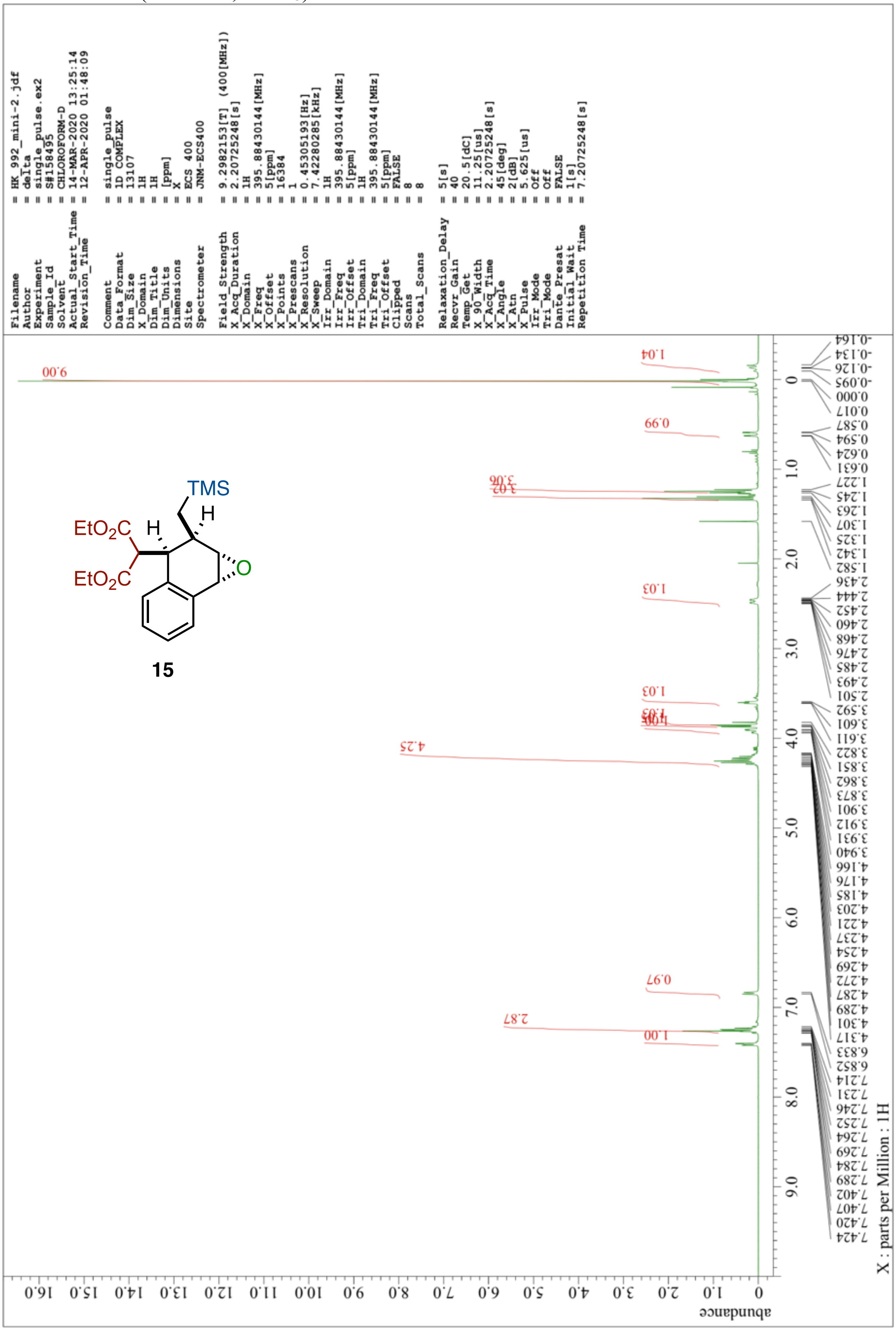

^{13}C NMR of **15** (101 MHz, $CDCl_3$)

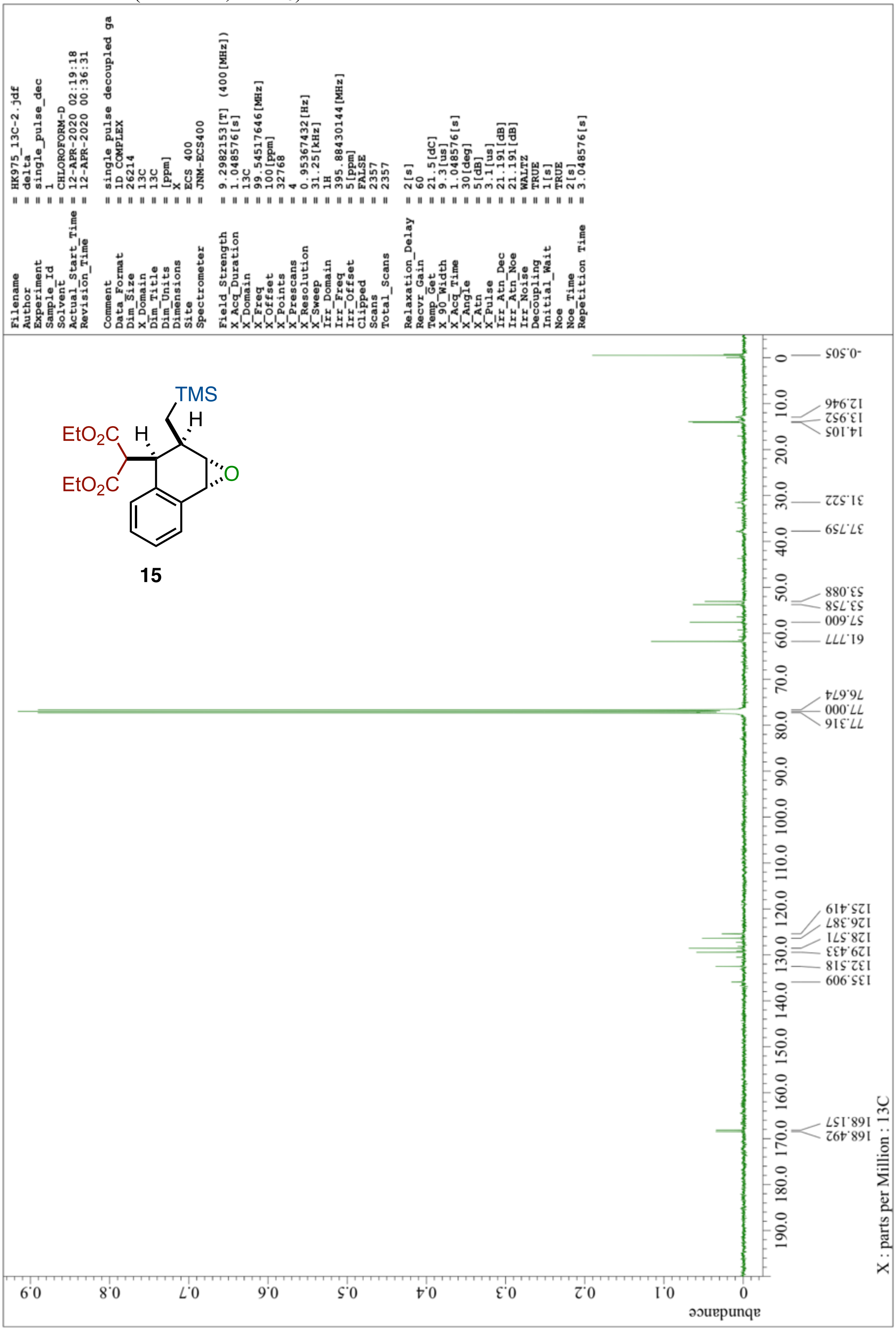

^{13}C NMR of **15** (101 MHz, $CDCl_3$, 323 K)

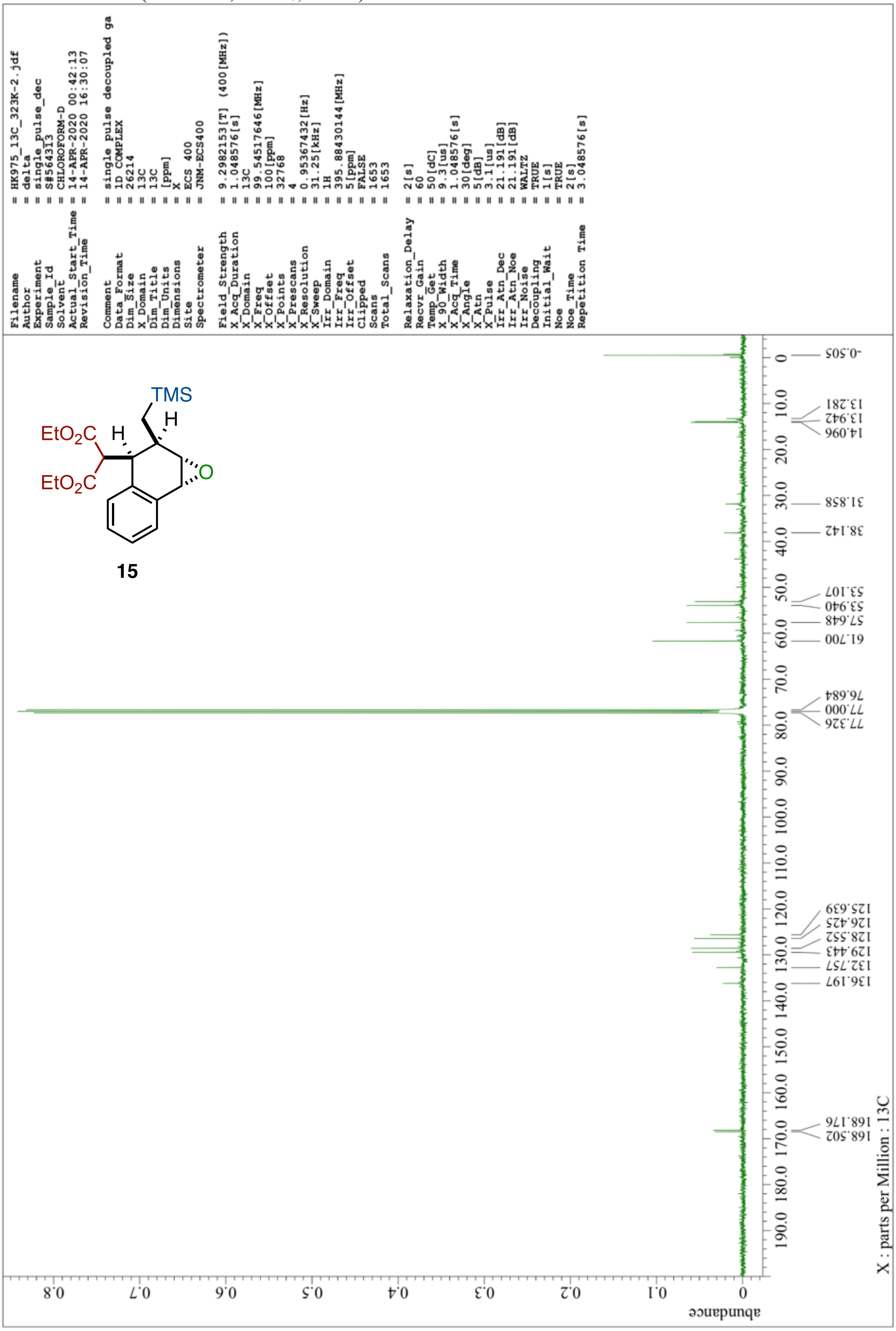

^{1}H NMR of **16** (400 MHz, $CDCl_3$)

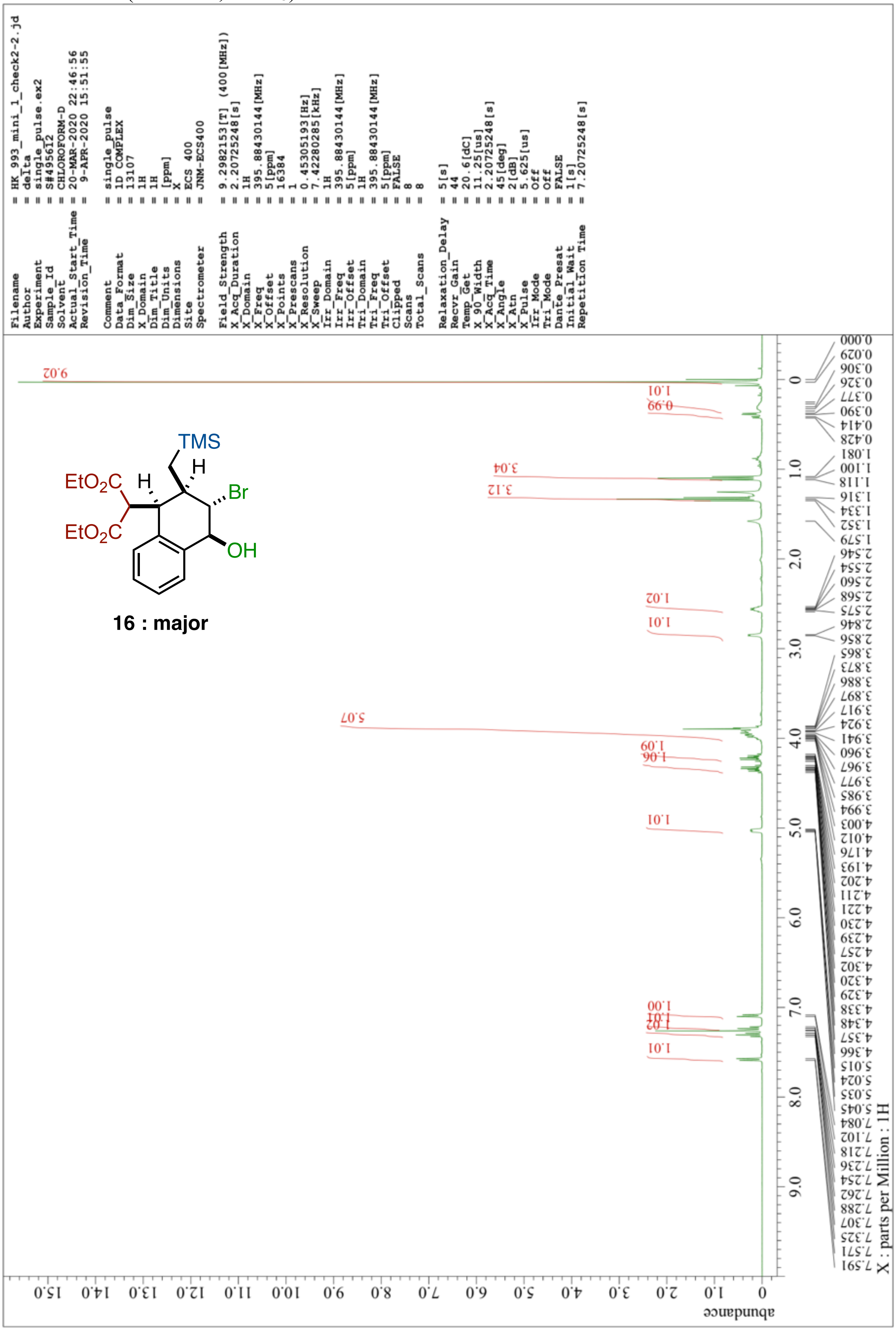

^{13}C NMR of **16** (101 MHz, $CDCl_3$)

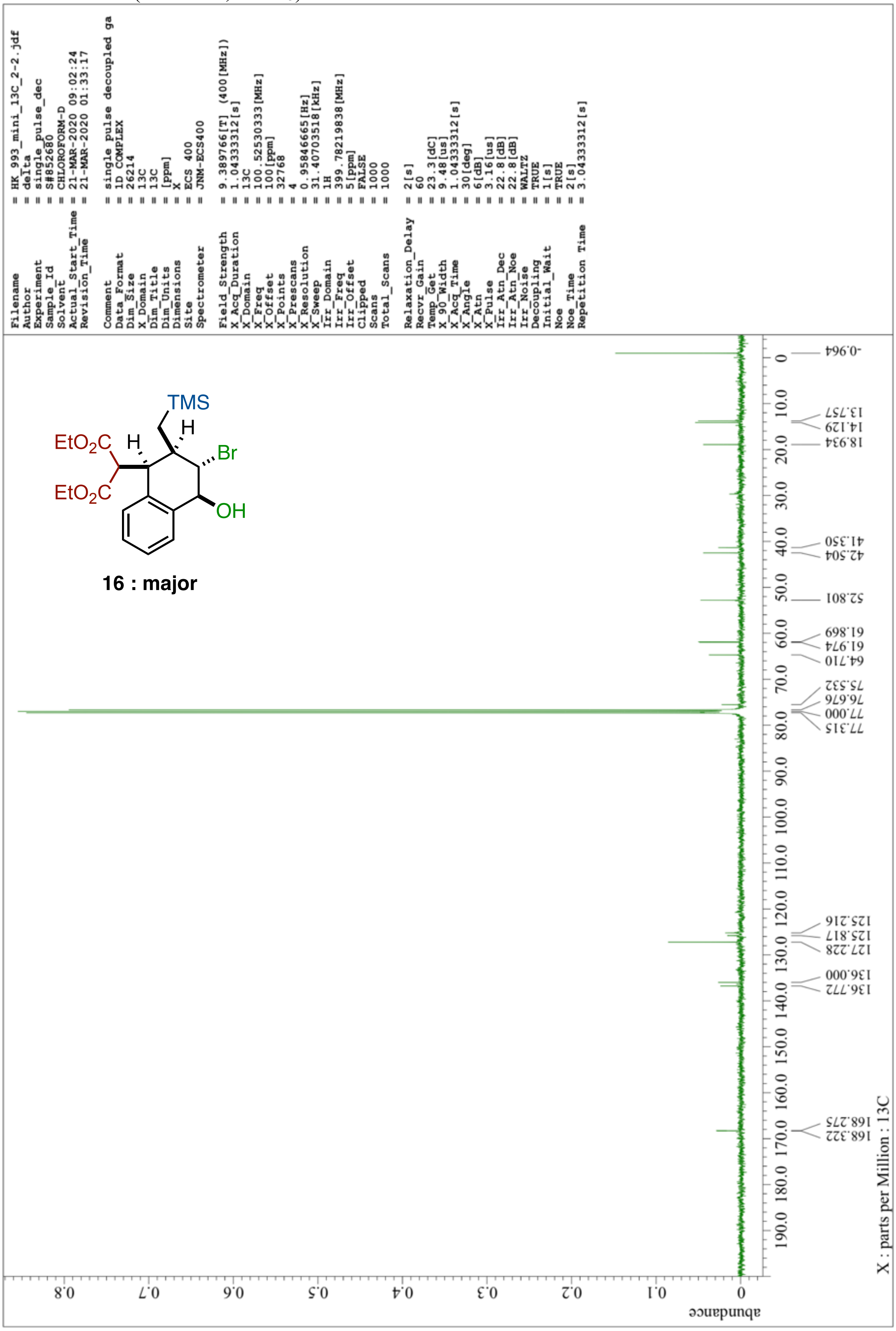

^{1}H NMR of **16’** (400 MHz, $CDCl_3$)

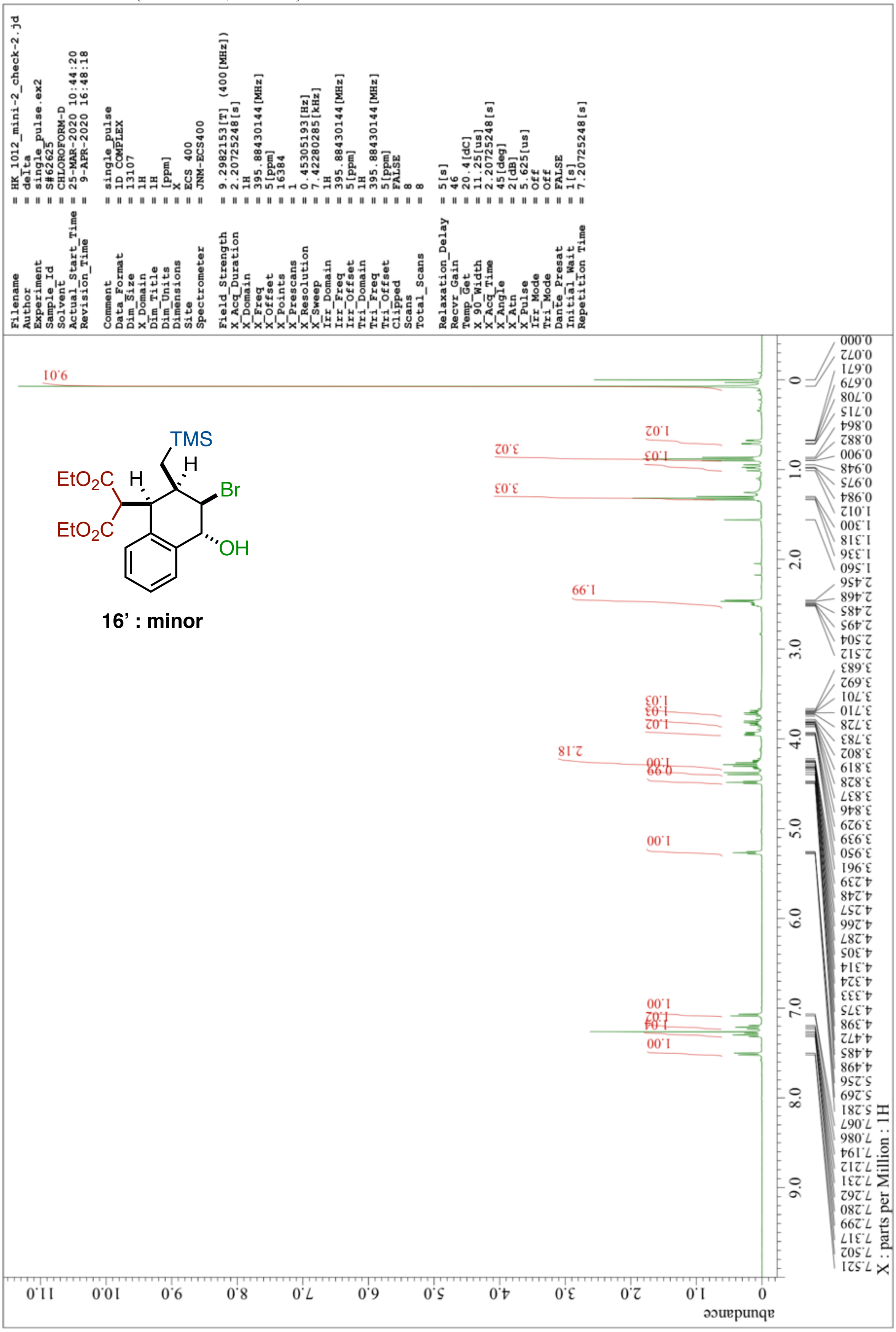

^{13}C NMR of **16’** (101 MHz, $CDCl_3$)

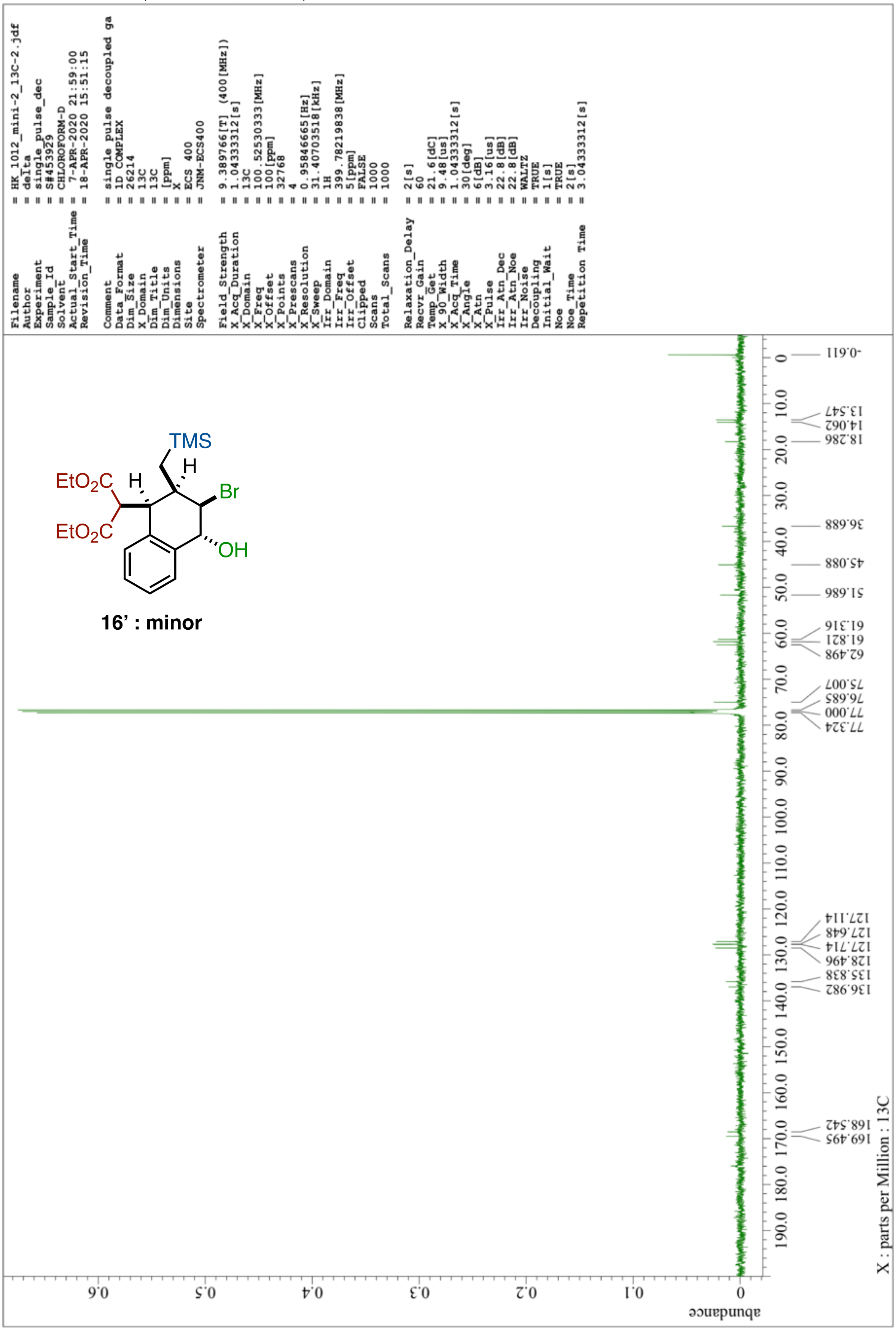

^{1}H NMR of **17** (400 MHz, $CDCl_3$)

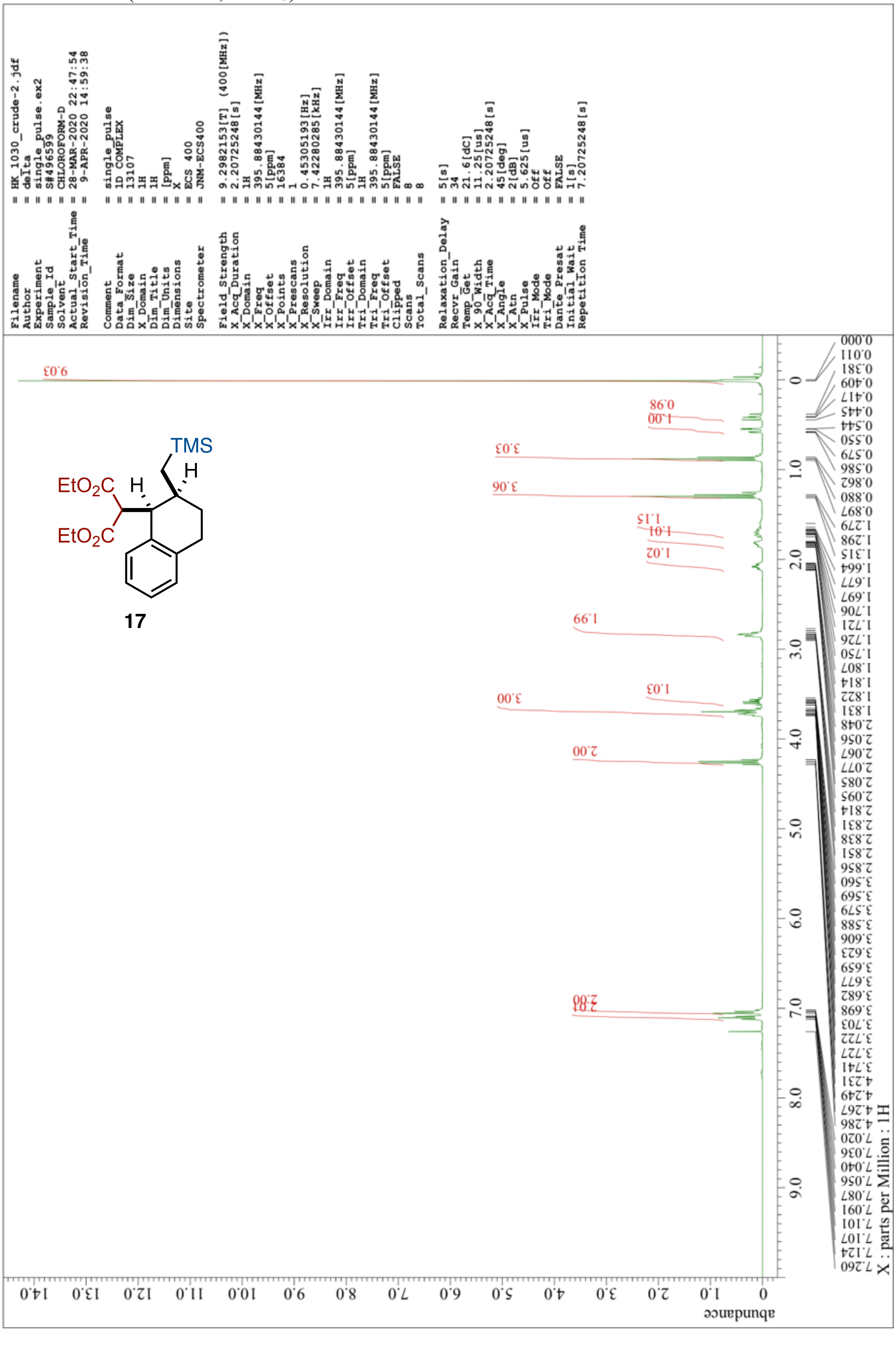

^{13}C NMR of **17** (101 MHz, $CDCl_3$)

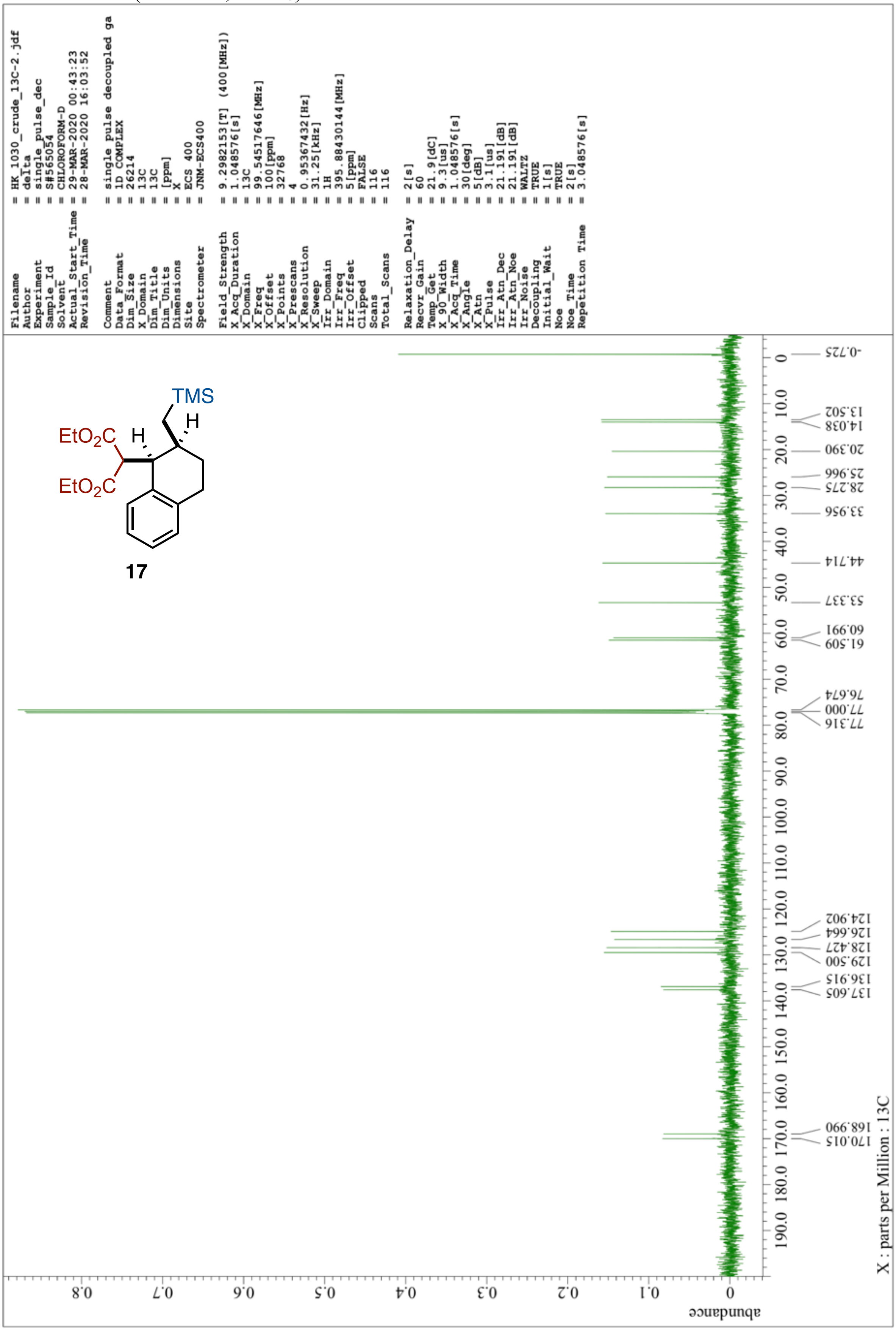

^{1}H NMR of **18** (400 MHz, $CDCl_3$)

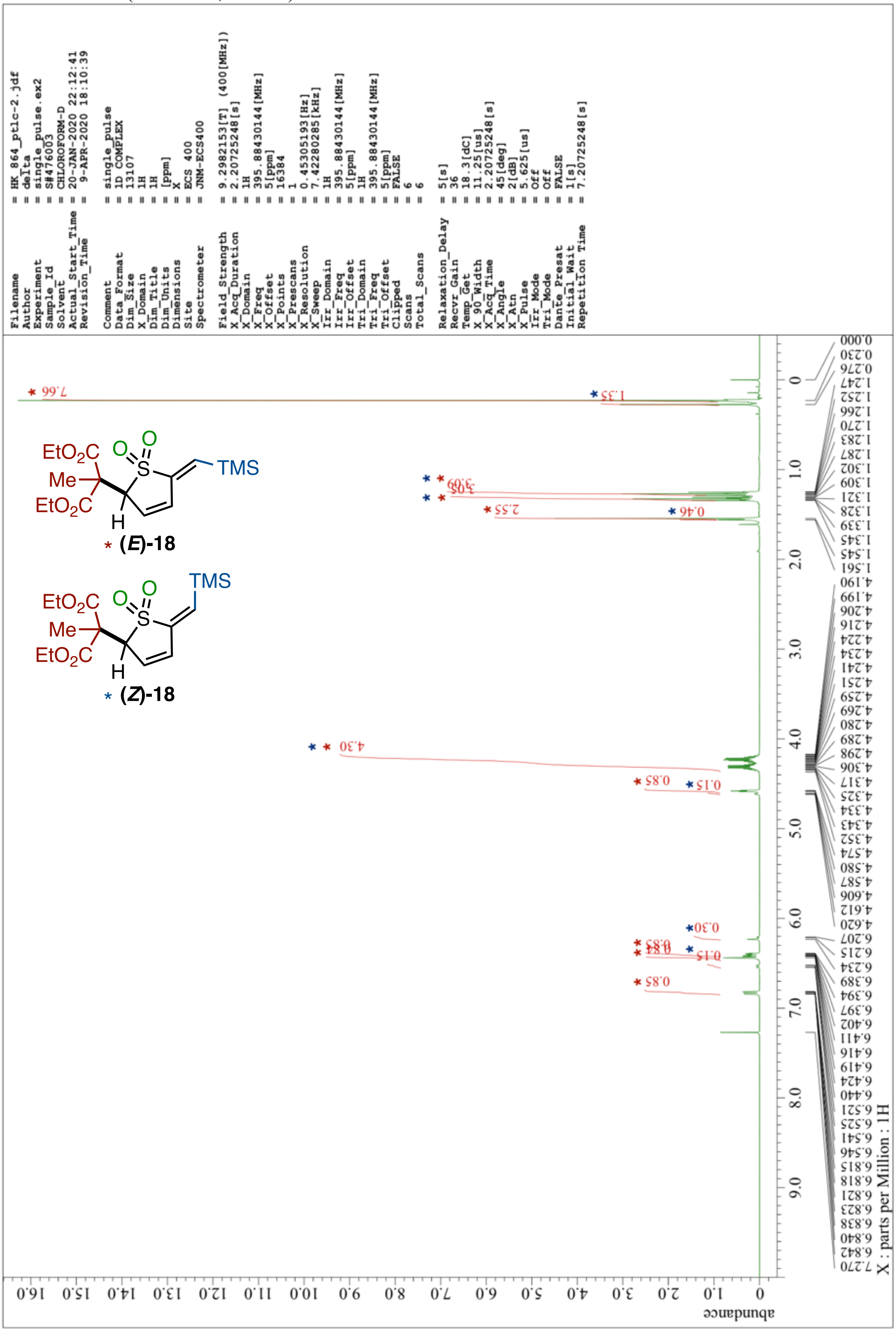

^{13}C NMR of **18** (101 MHz, $CDCl_3$)

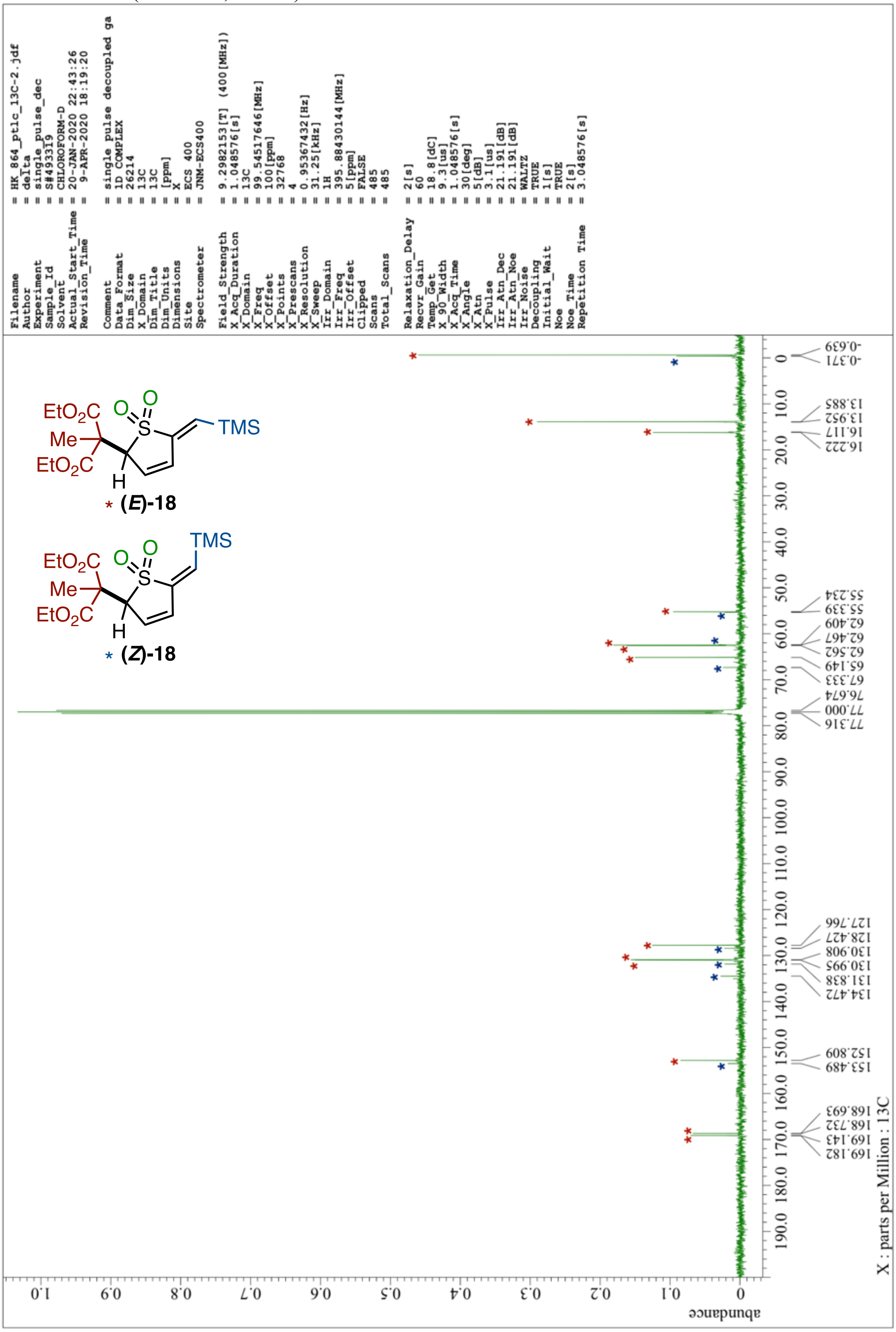

11. Crude ^{1}H NMR spectra of 4, 6, 8 and 9

^{1}H NMR of **4Aa** (400 MHz, $CDCl_3$)

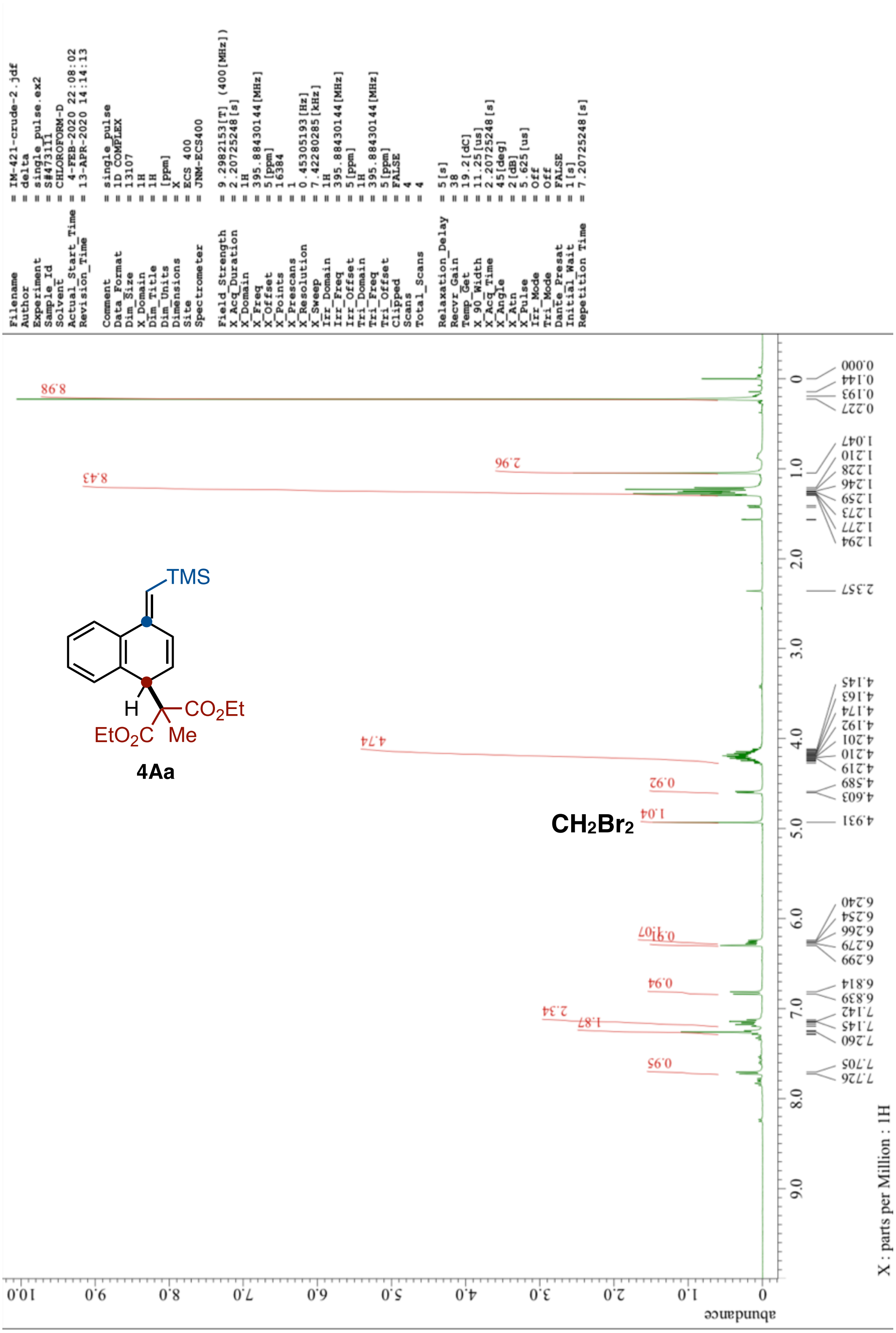

^{1}H NMR of **4Ab** (400 MHz, $CDCl_3$)

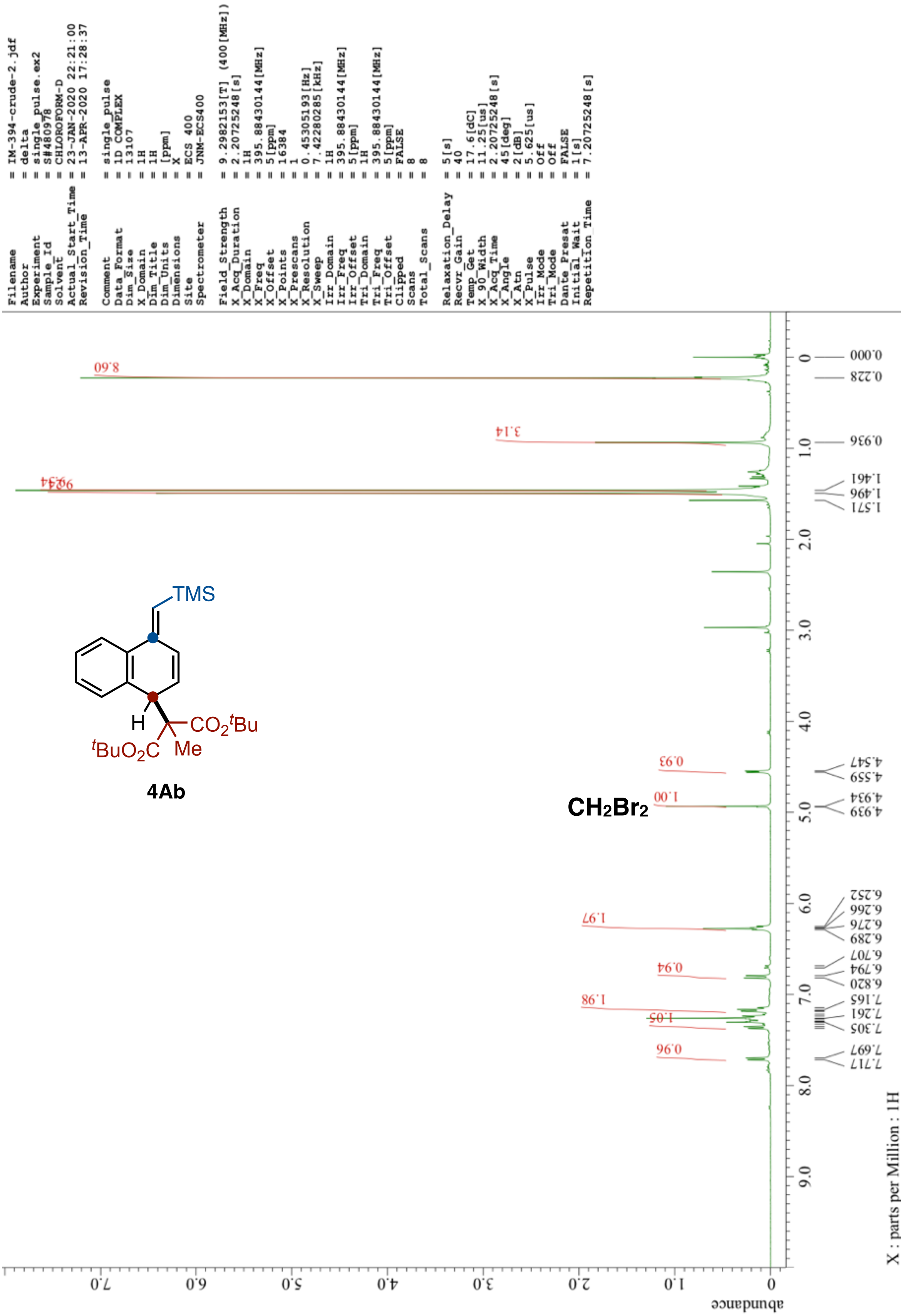

^{1}H NMR of **4Ba** (400 MHz, $CDCl_3$)

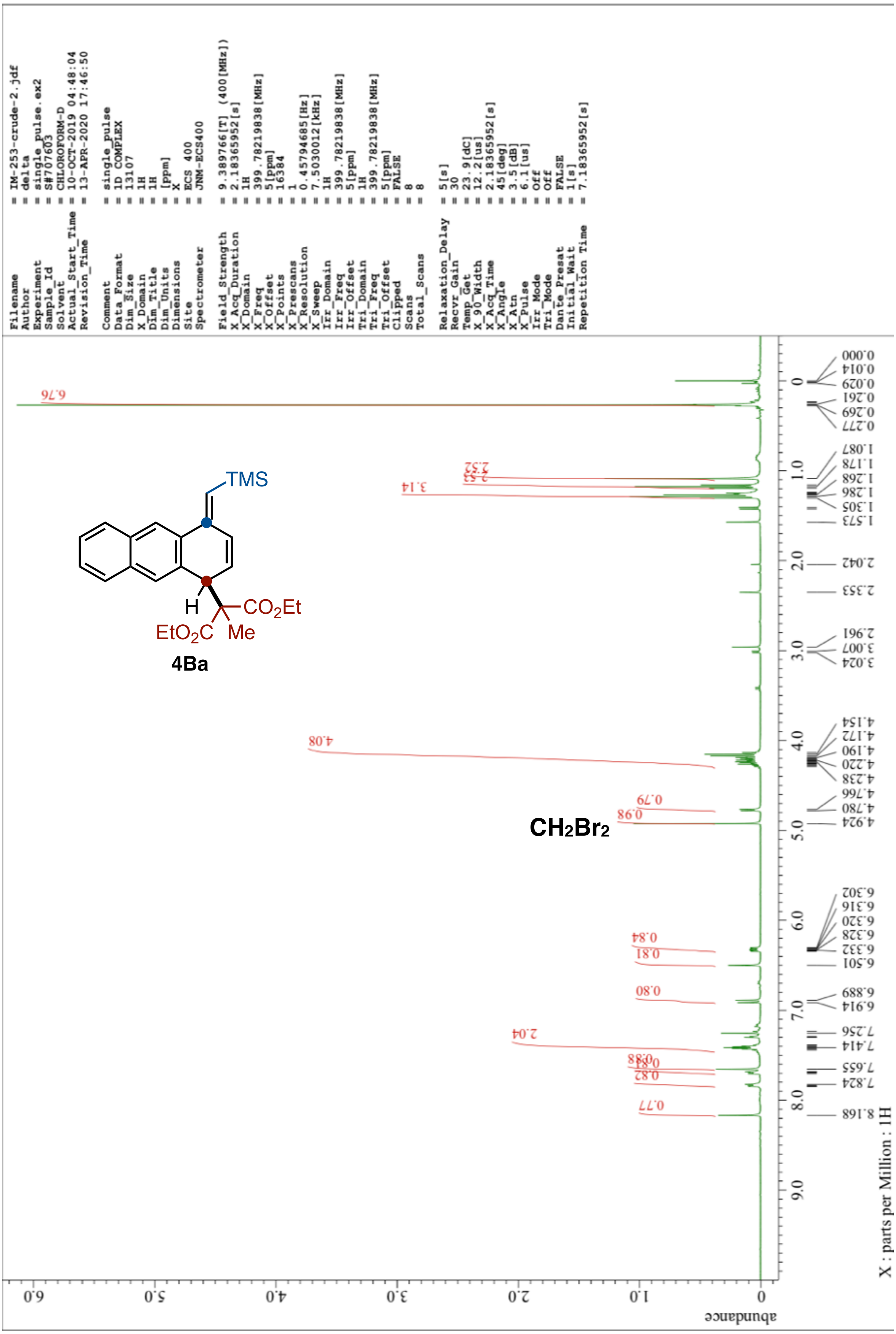

^{1}H NMR of **4Ca** (400 MHz, $CDCl_3$)

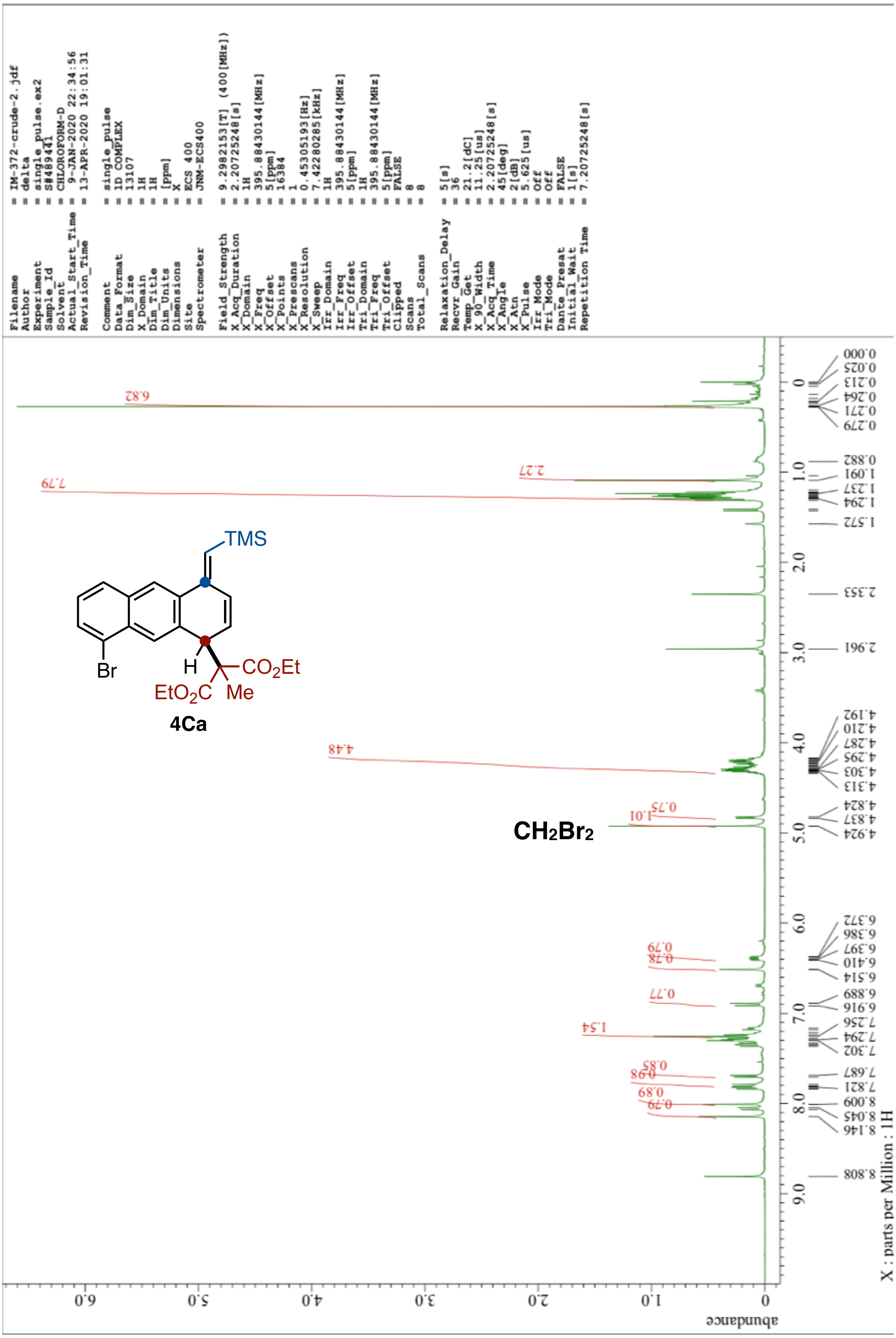

^{1}H NMR of **4Caa** (400 MHz, $CDCl_3$)

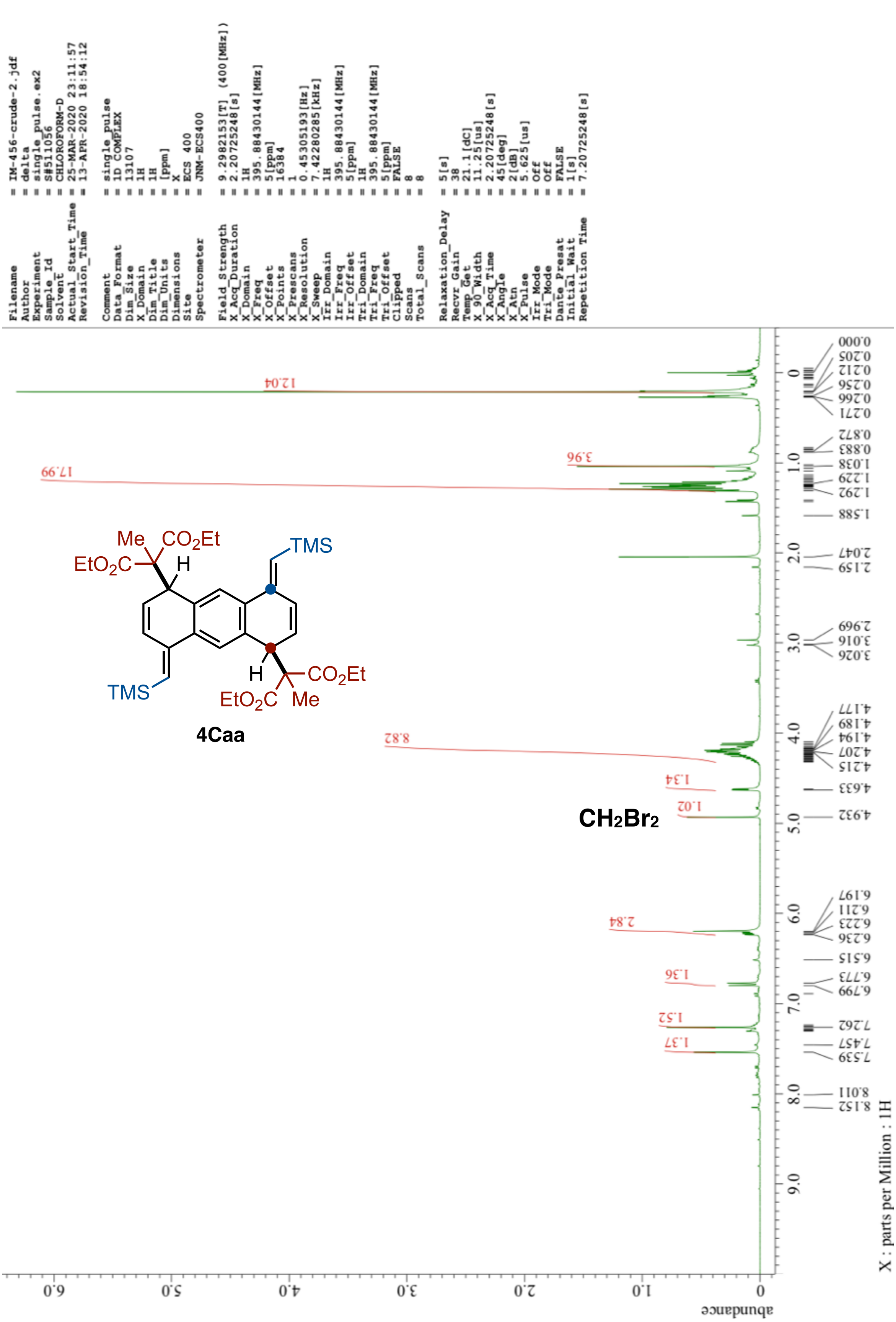

^{1}H NMR of **4Da** (400 MHz, $CDCl_3$)

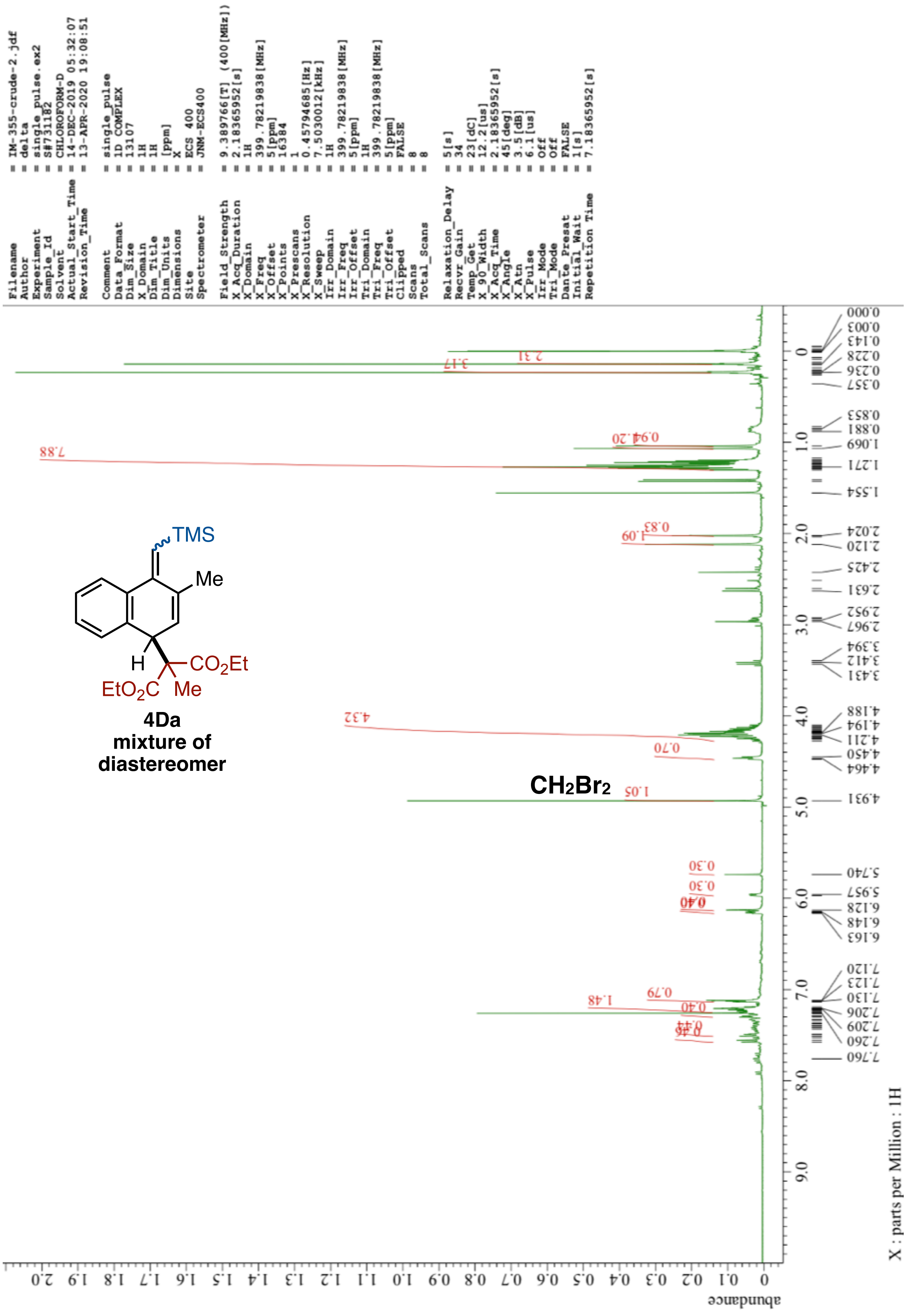

^{1}H NMR of **4Ea** (400 MHz, $CDCl_3$)

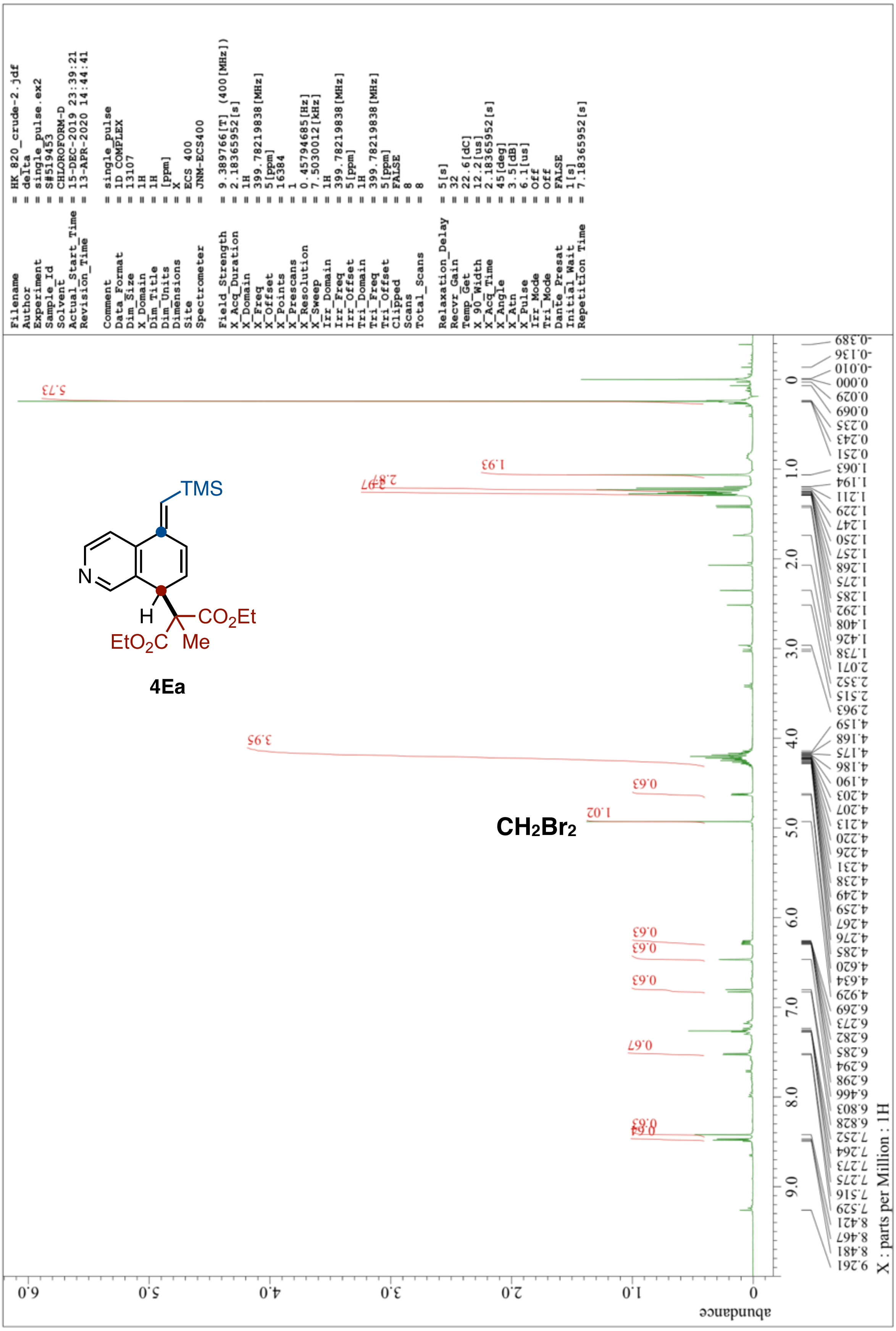

^{1}H NMR of **4Fa** (400 MHz, $CDCl_3$)

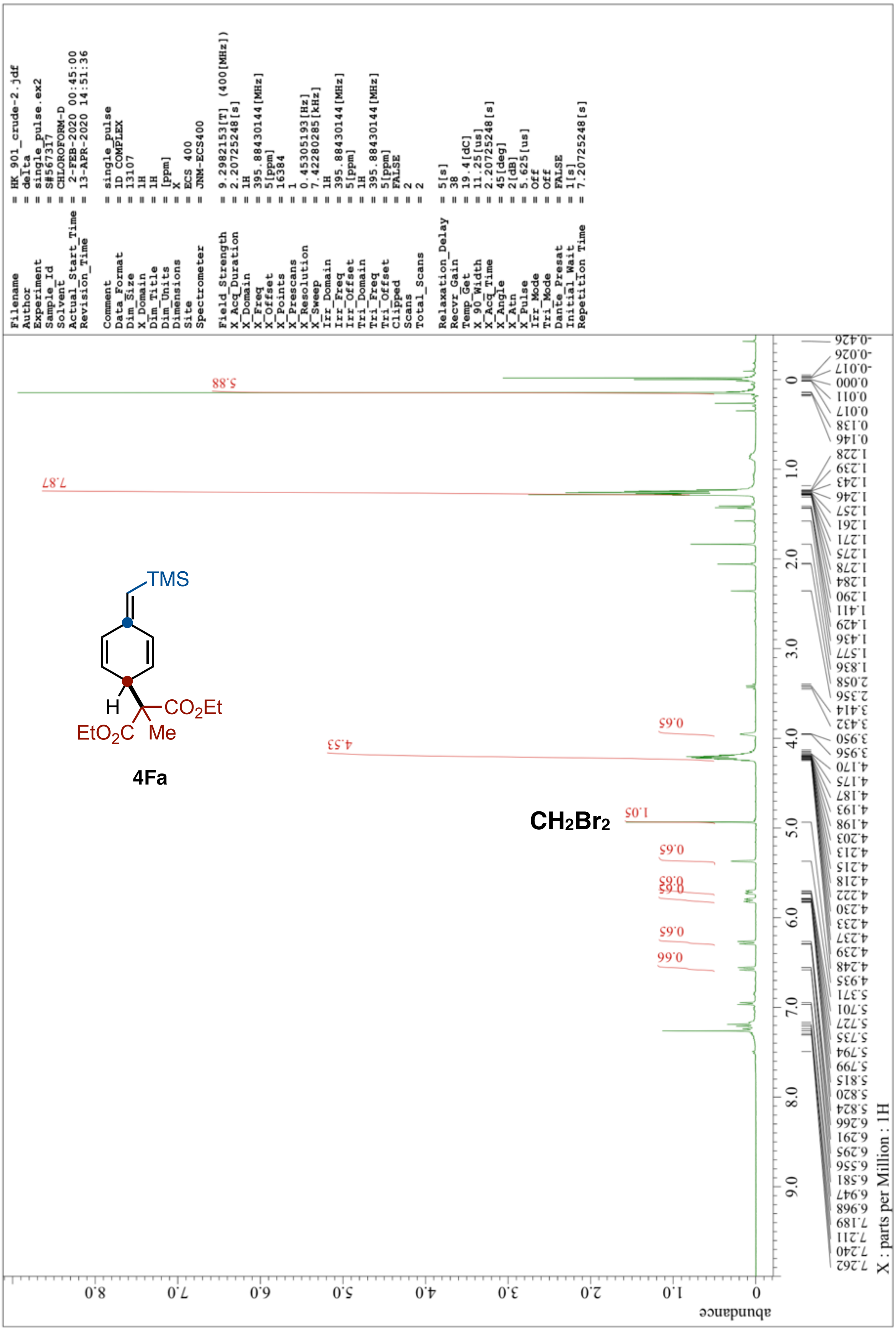

^{1}H NMR of **4Ga** (400 MHz, $CDCl_3$)

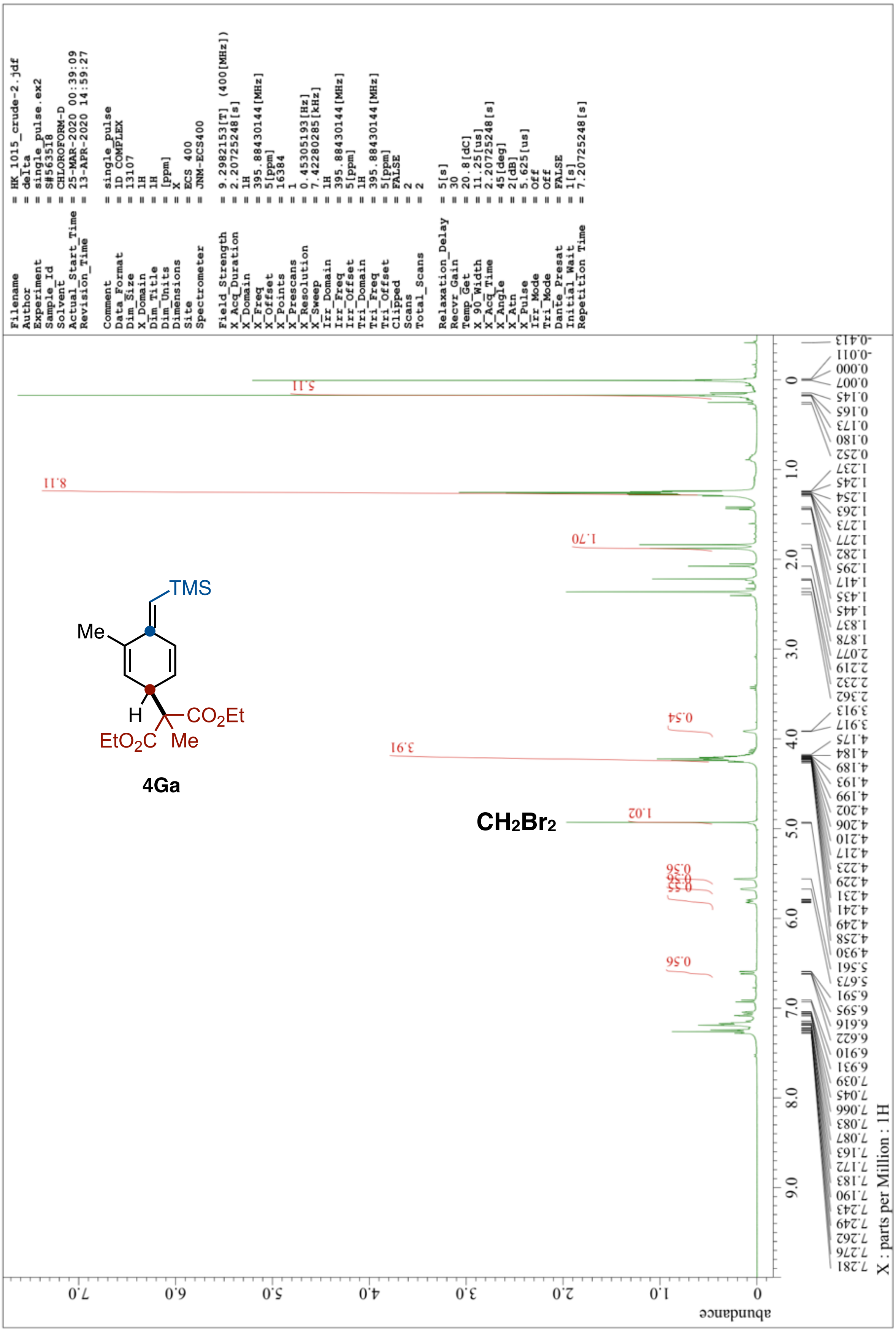

^{1}H NMR of **4Ac** (400 MHz, $CDCl_3$)

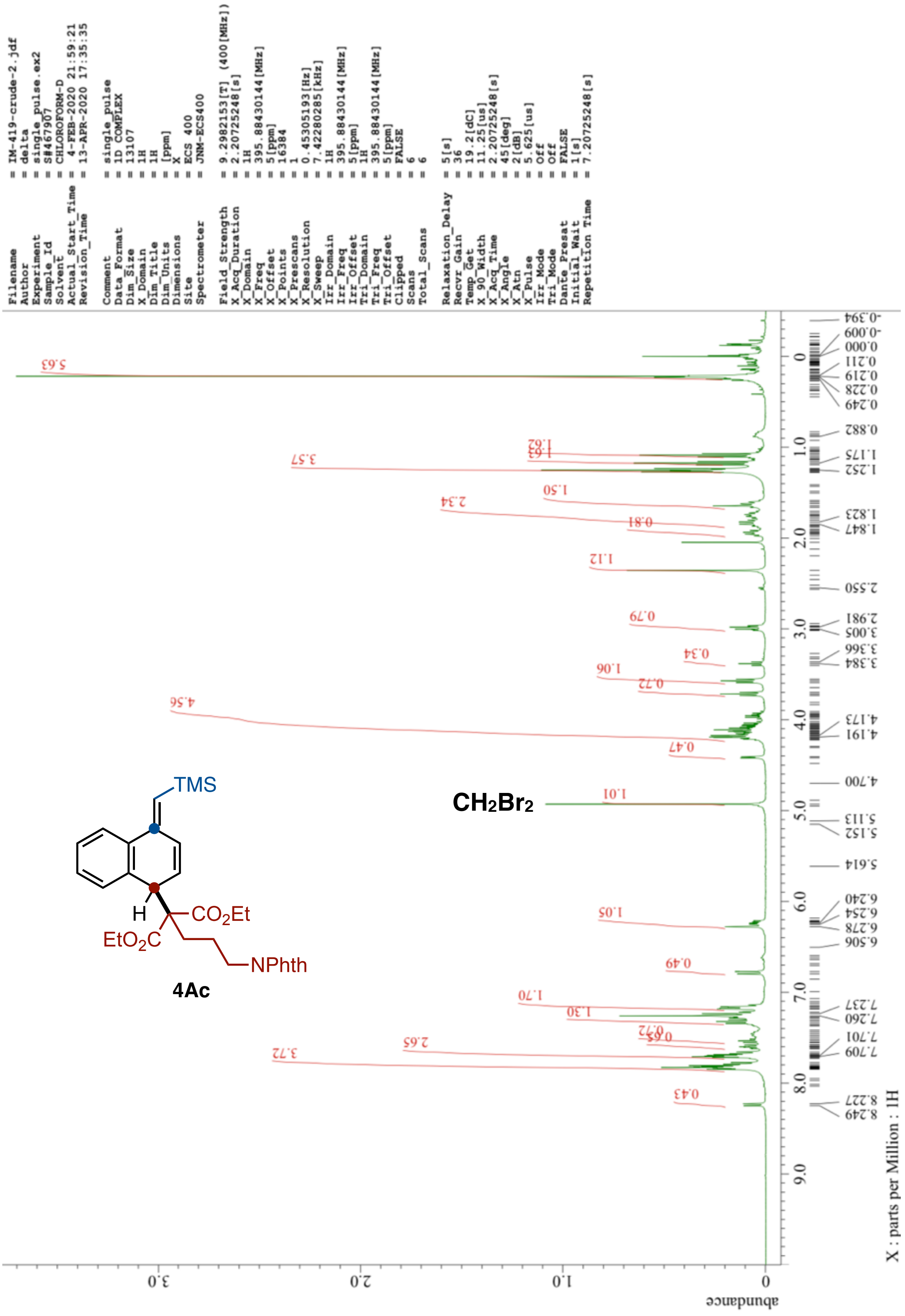

^{1}H NMR of **4Ad** (400 MHz, $CDCl_3$)

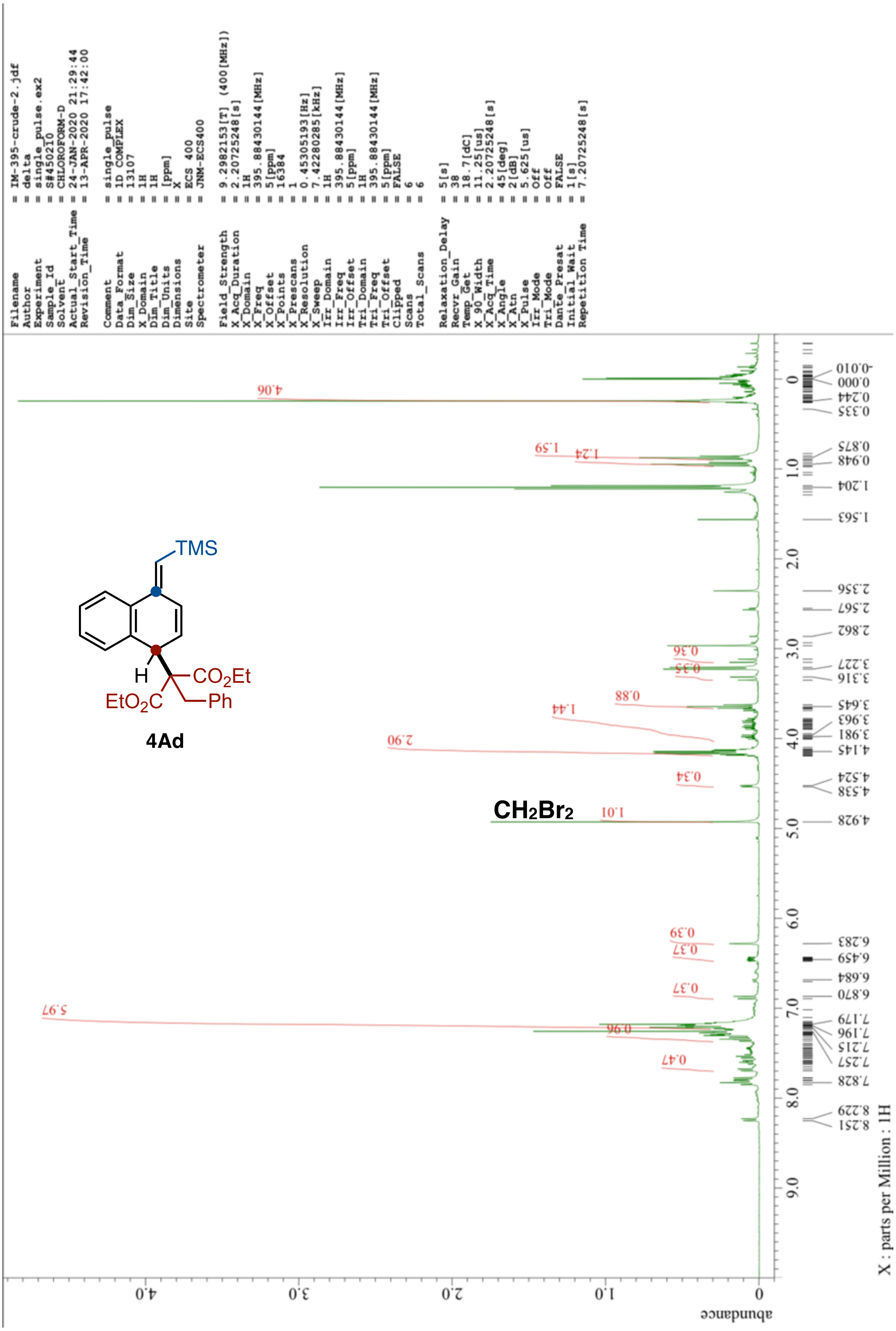

^{1}H NMR of **4He** (400 MHz, $CDCl_3$)

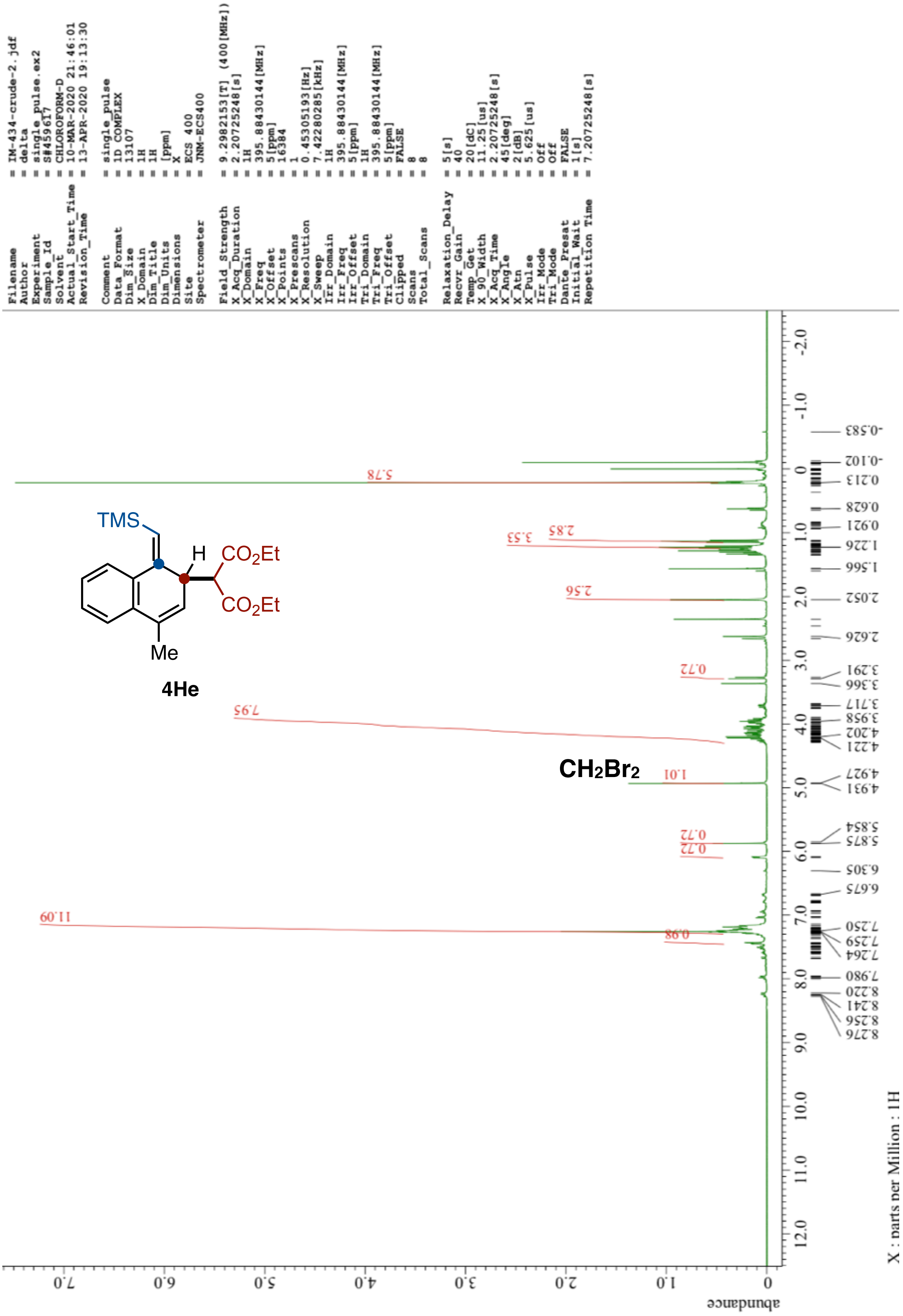

^{1}H NMR of **4Ie** (400 MHz, $CDCl_3$)

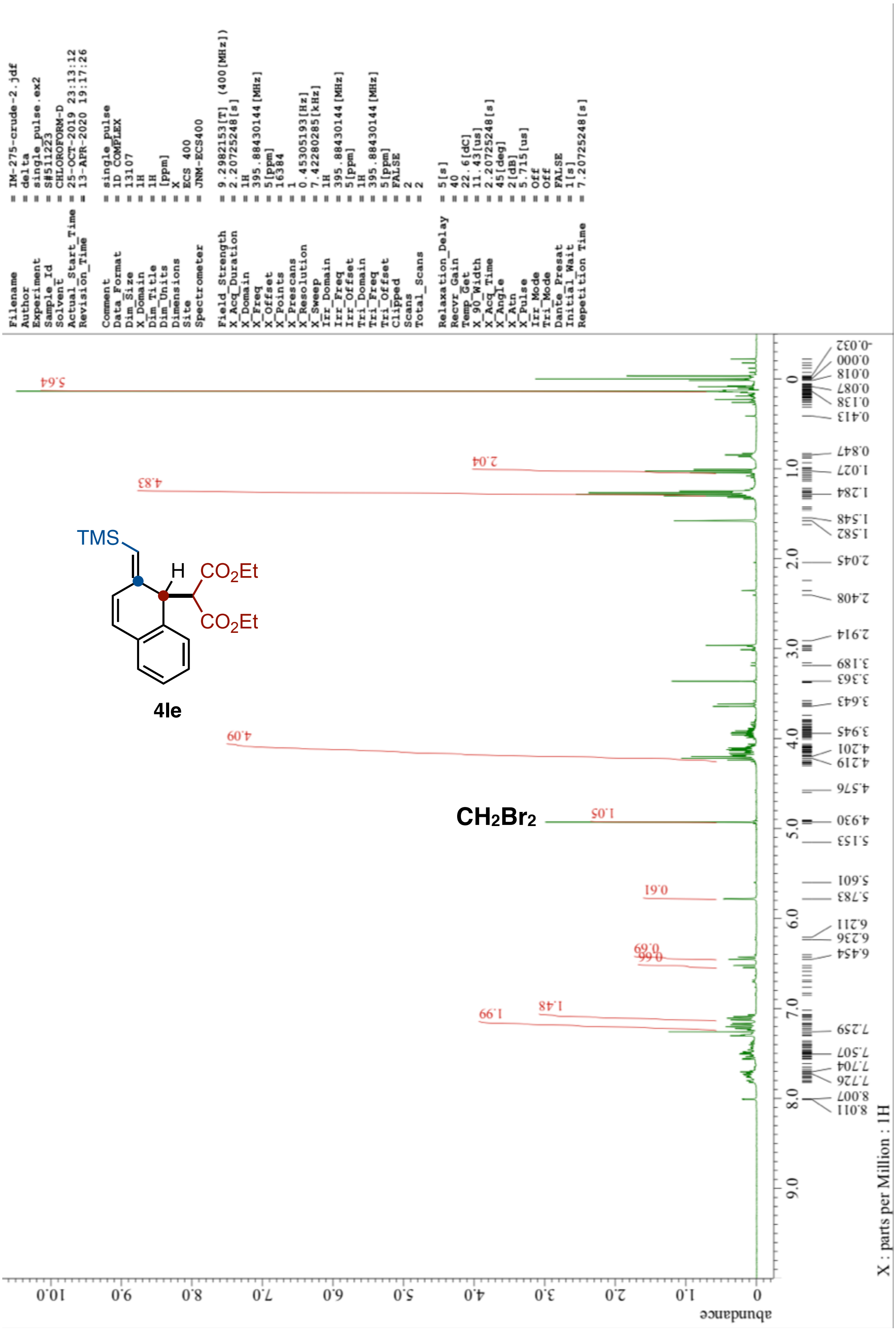

^{1}H NMR of **4Ja** (400 MHz, $CDCl_3$)

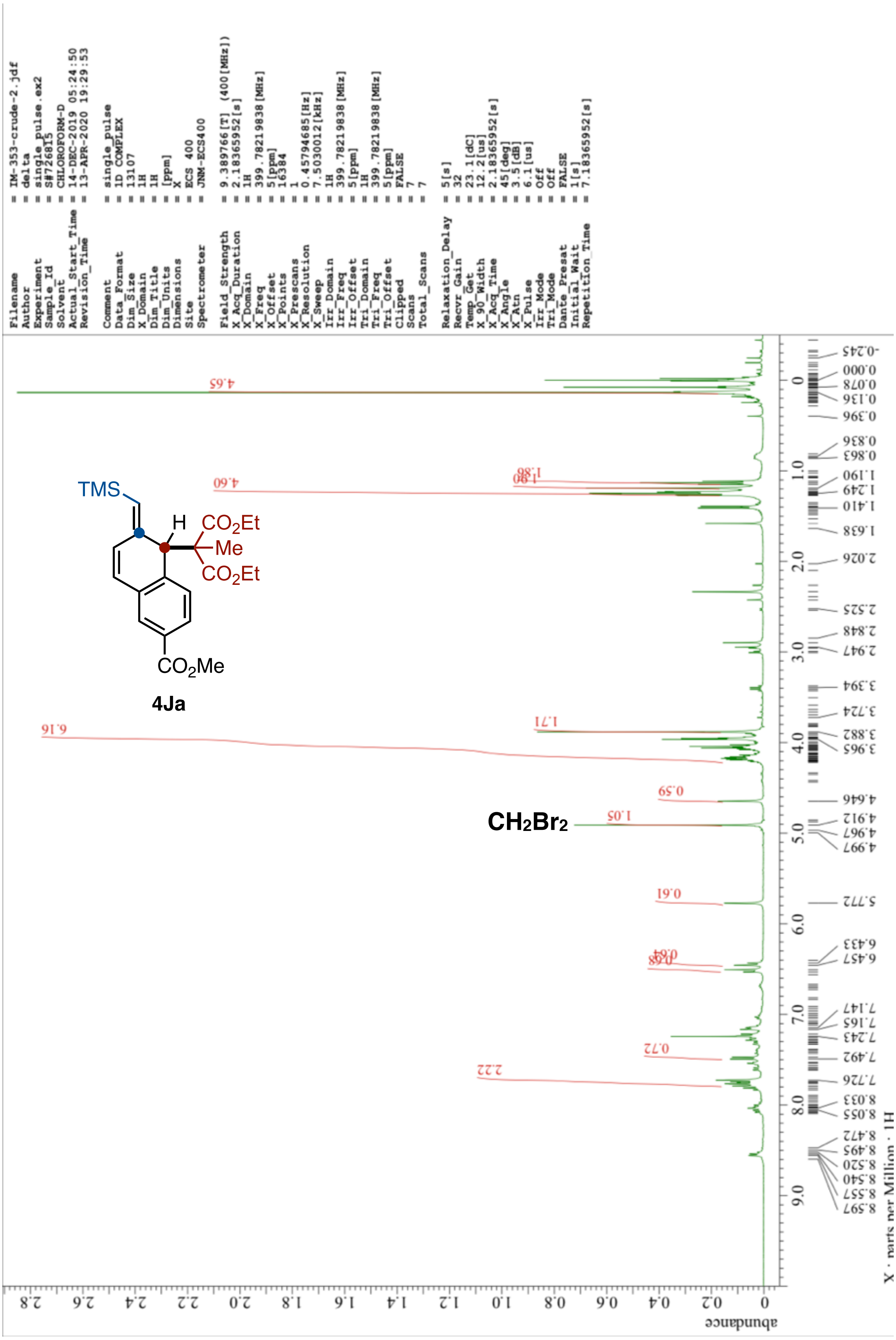

^{1}H NMR of **4Ka** (400 MHz, $CDCl_3$)

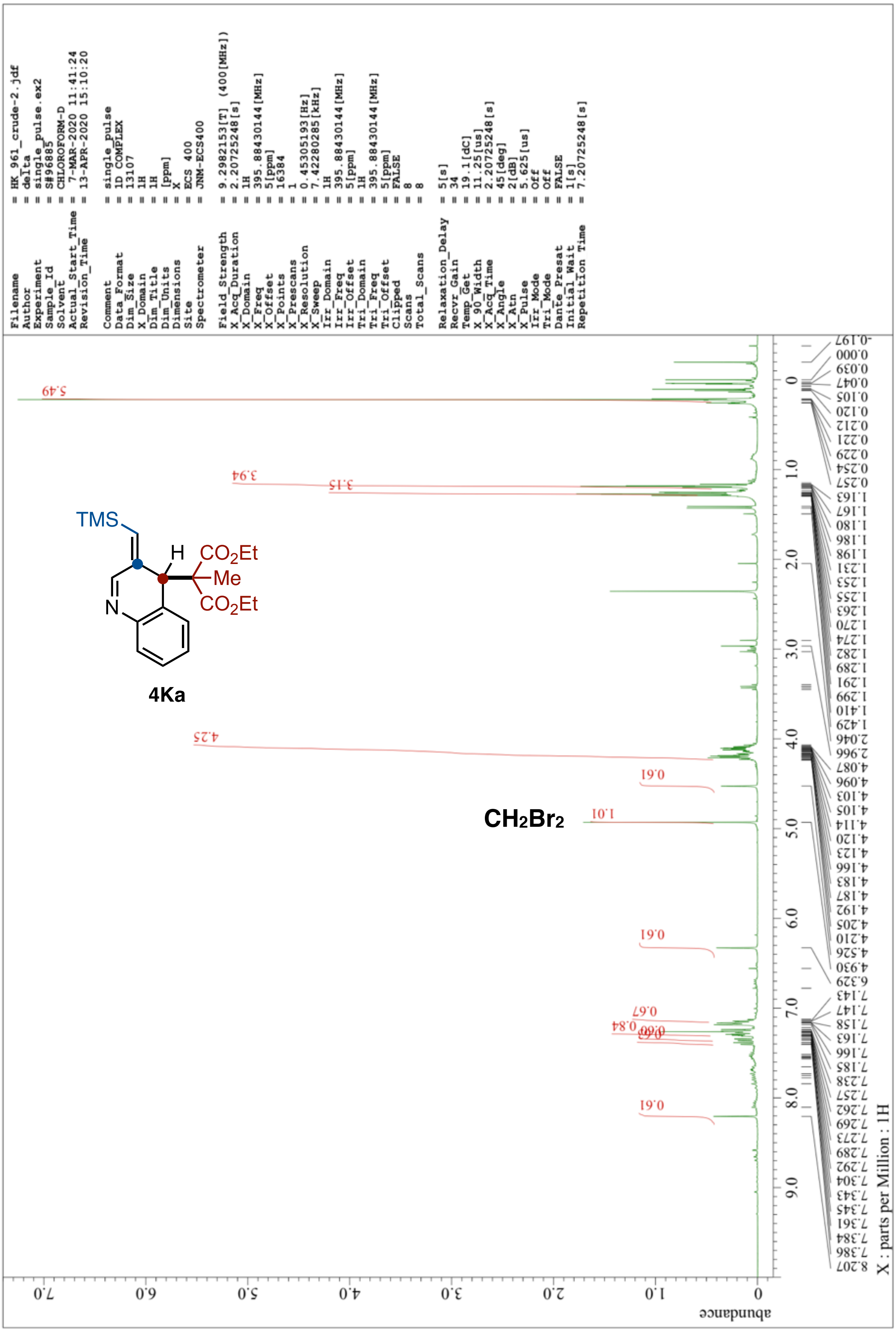

^{1}H NMR of **6Aa** (400 MHz, $CDCl_3$)

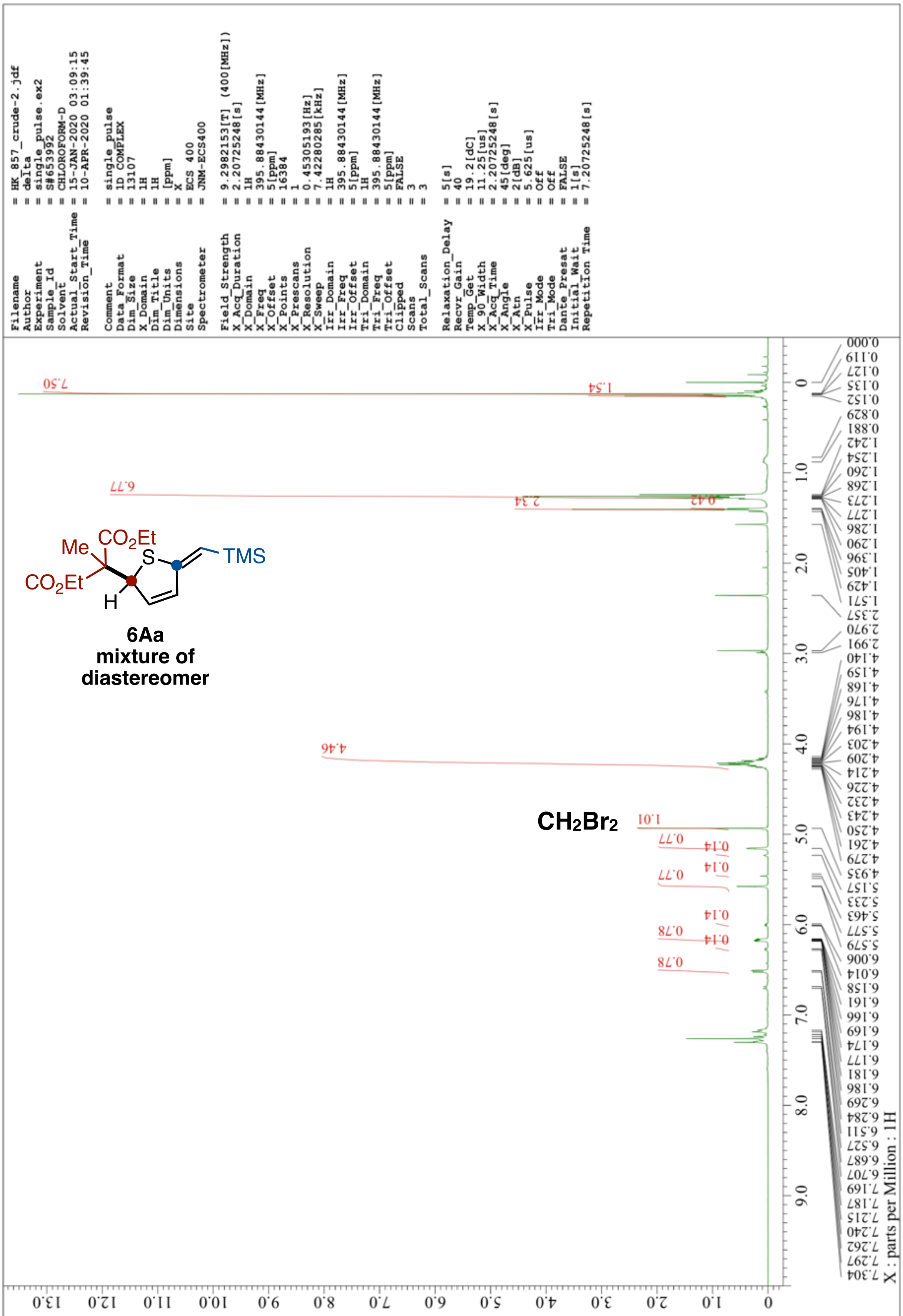

^{1}H NMR of **6Ab** (400 MHz, $CDCl_3$)

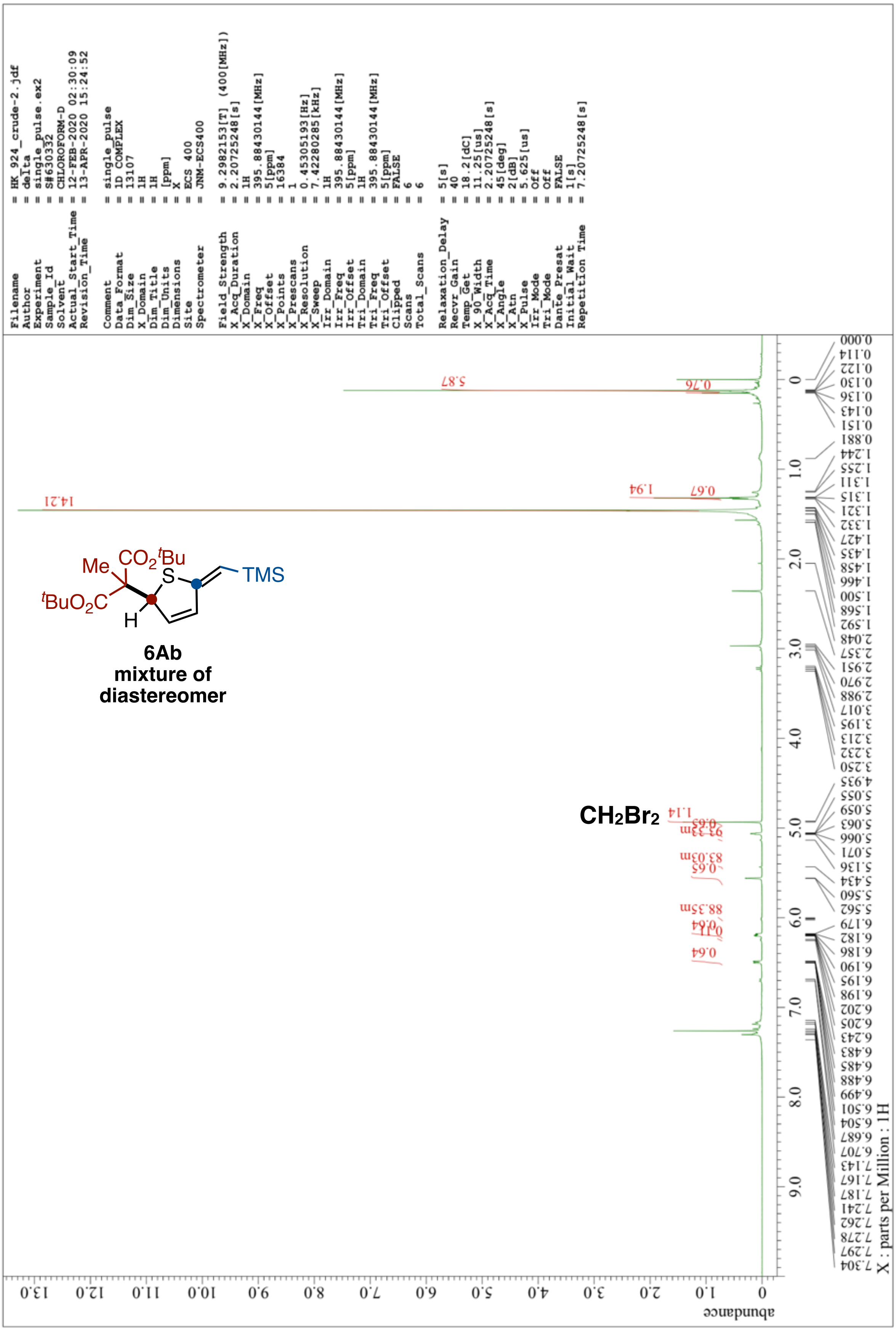

^{1}H NMR of **6Ba** (400 MHz, $CDCl_3$)

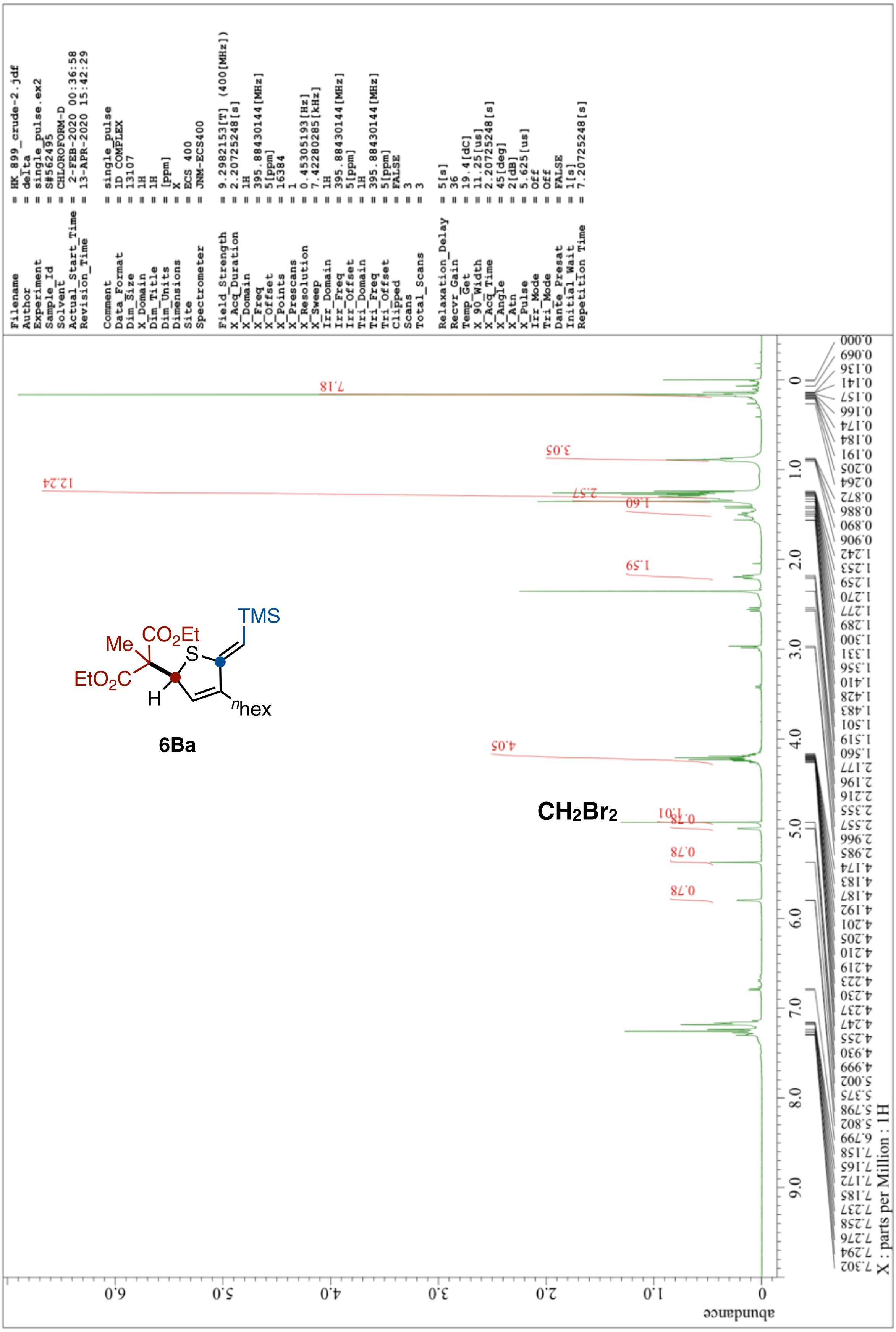

^{1}H NMR of **6Ca** (400 MHz, $CDCl_3$)

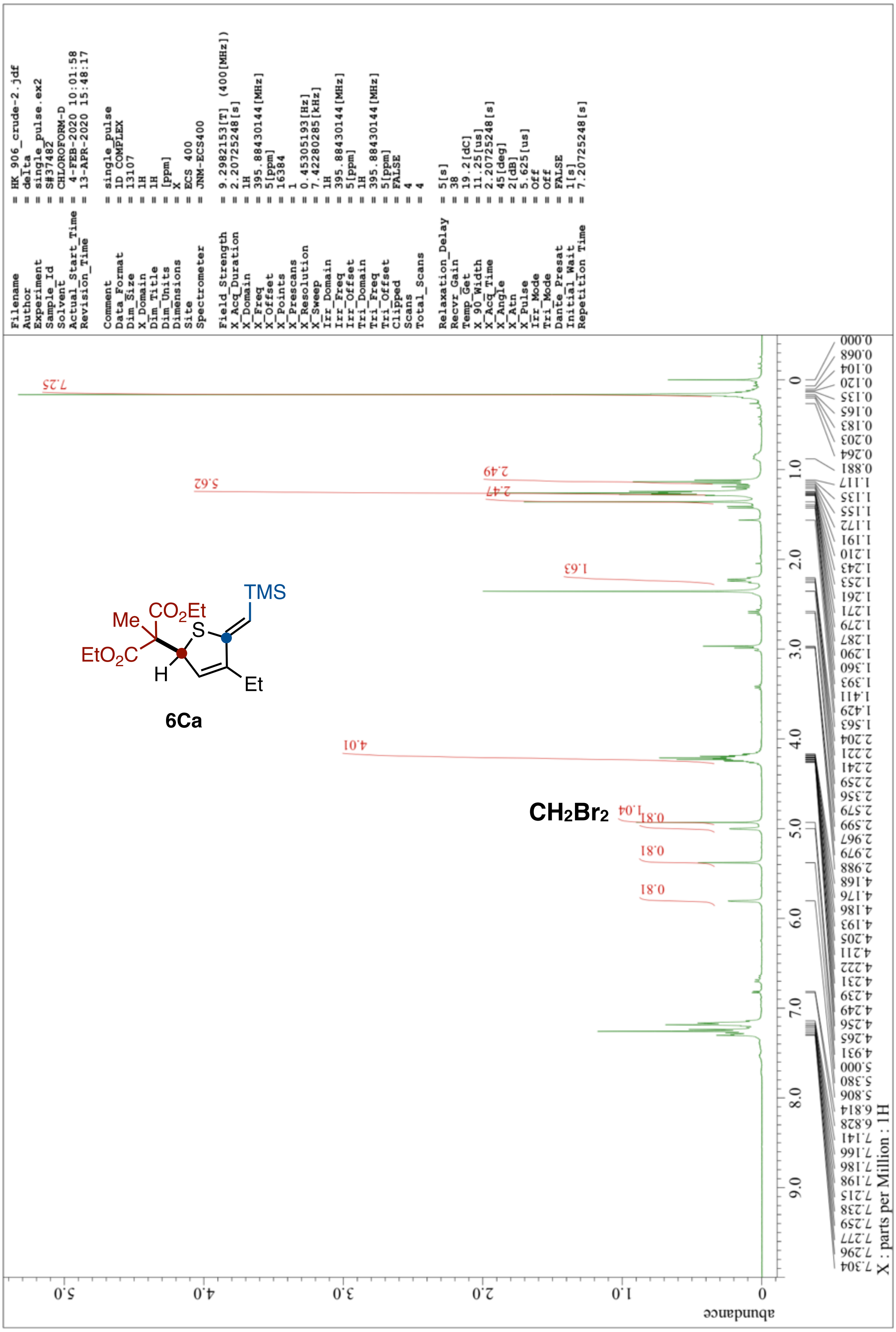

^{1}H NMR of **6Da** (400 MHz, $CDCl_3$)

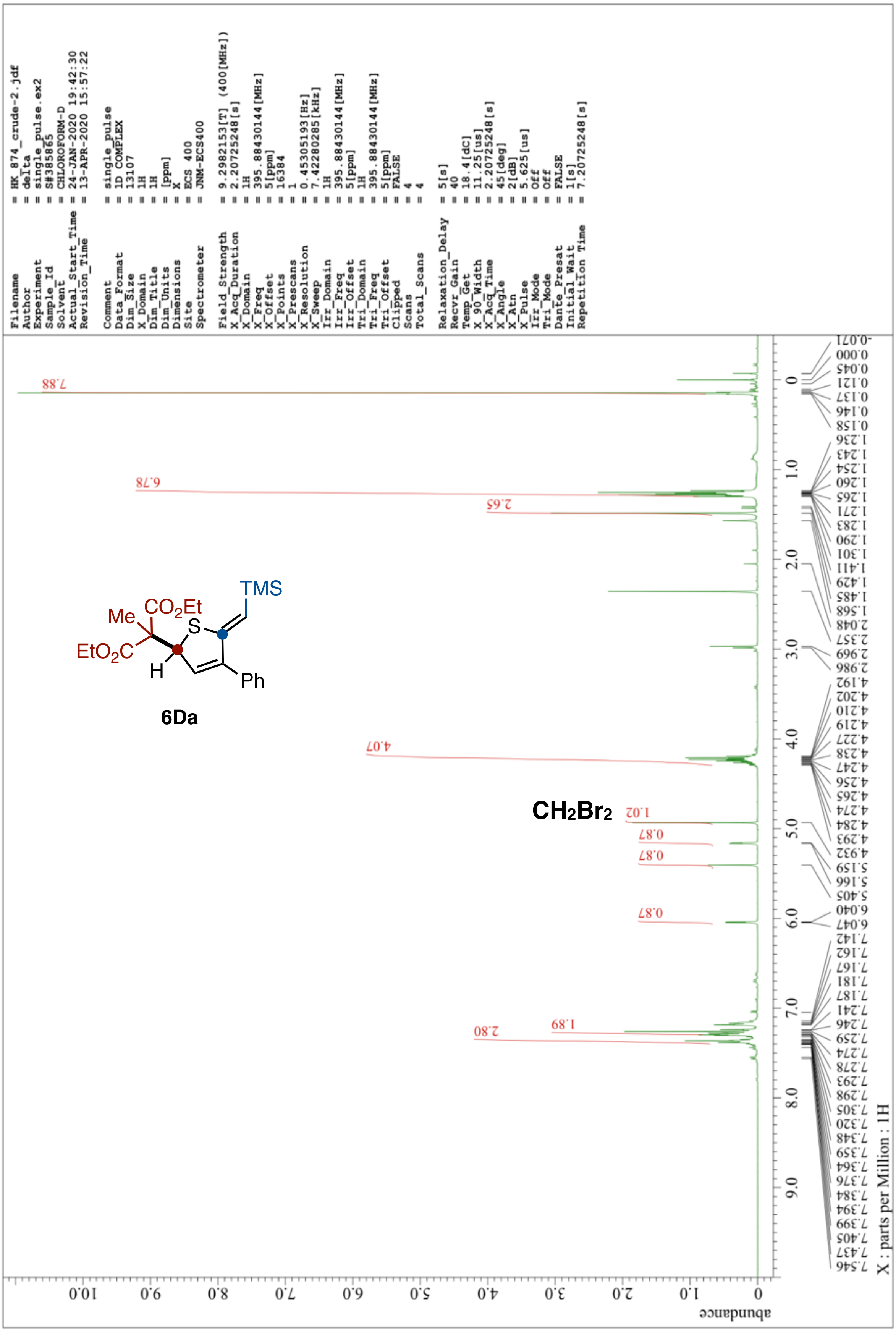

^{1}H NMR of **6Ea** (400 MHz, $CDCl_3$)

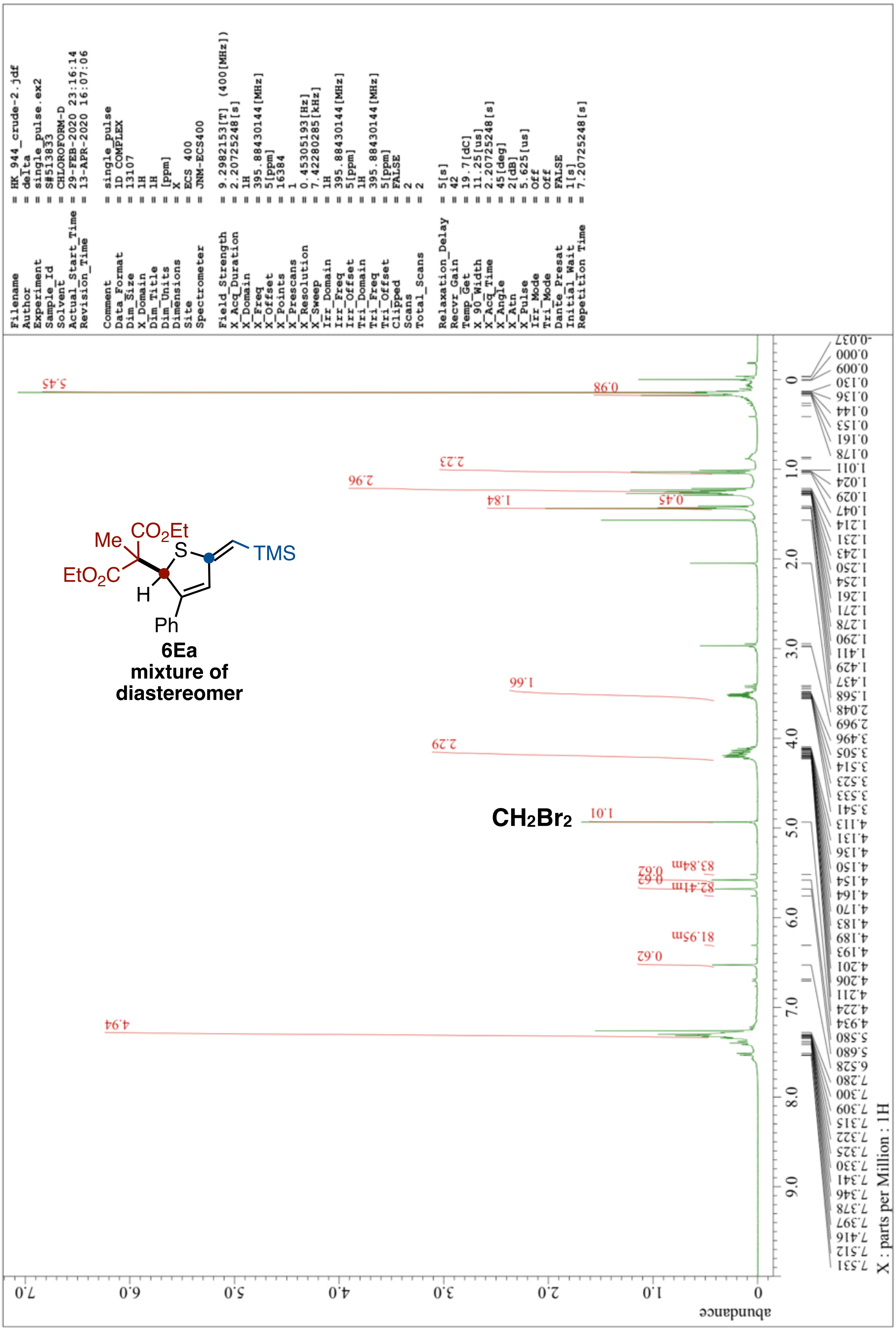

^{1}H NMR of **6Fa** (400 MHz, $CDCl_3$)

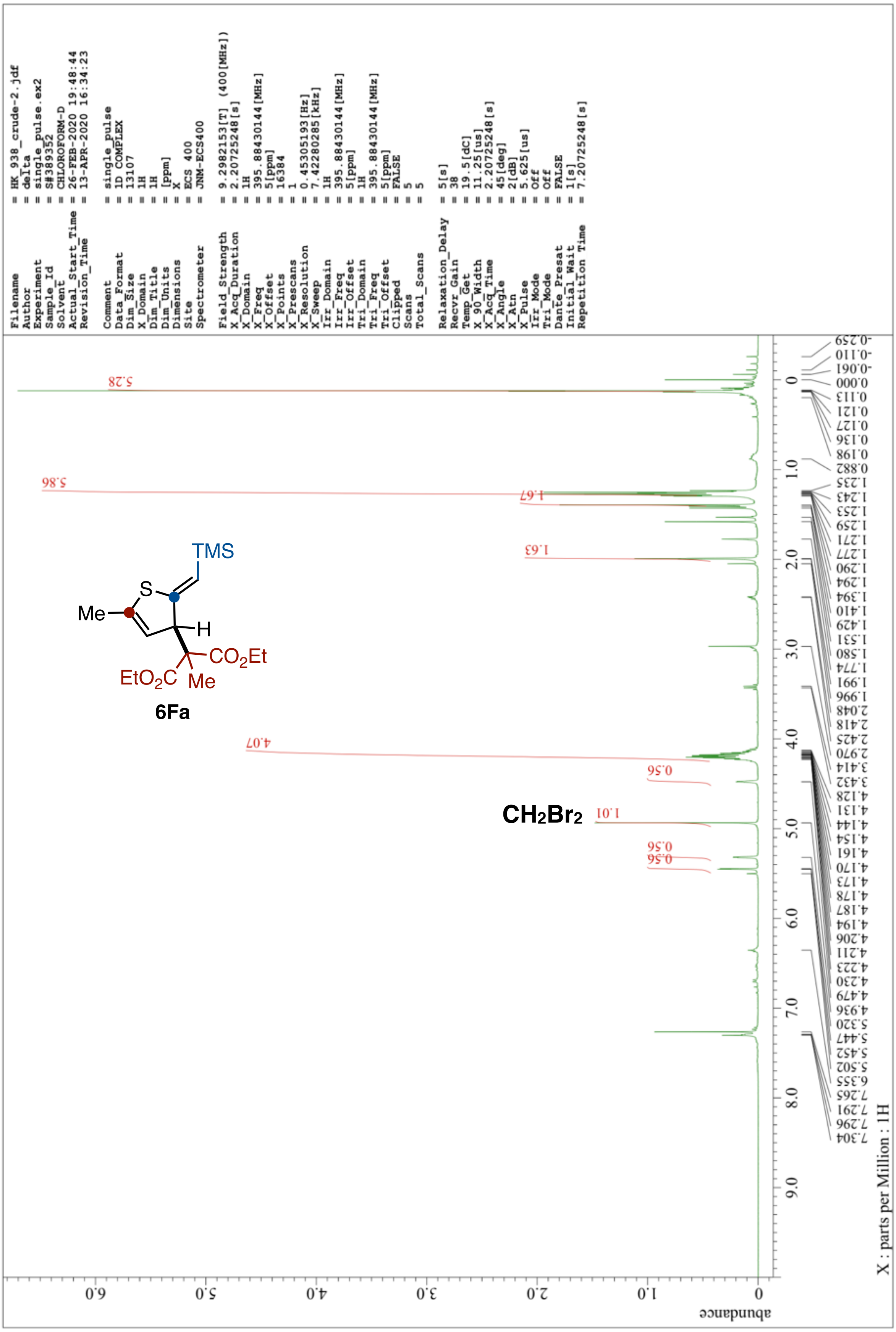

^{1}H NMR of **6Ga** (400 MHz, $CDCl_3$)

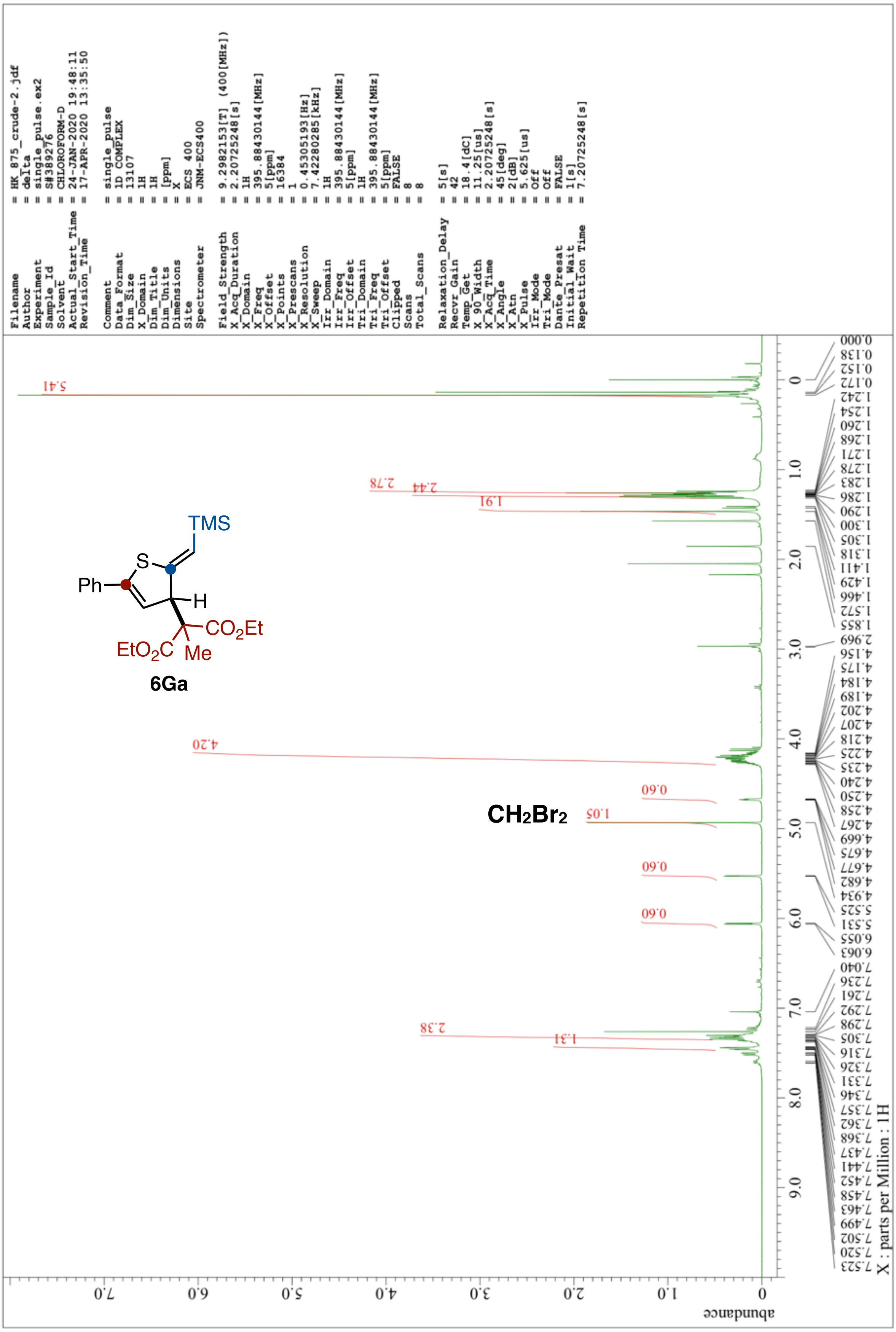

^{1}H NMR of **6Ha** (400 MHz, $CDCl_3$)

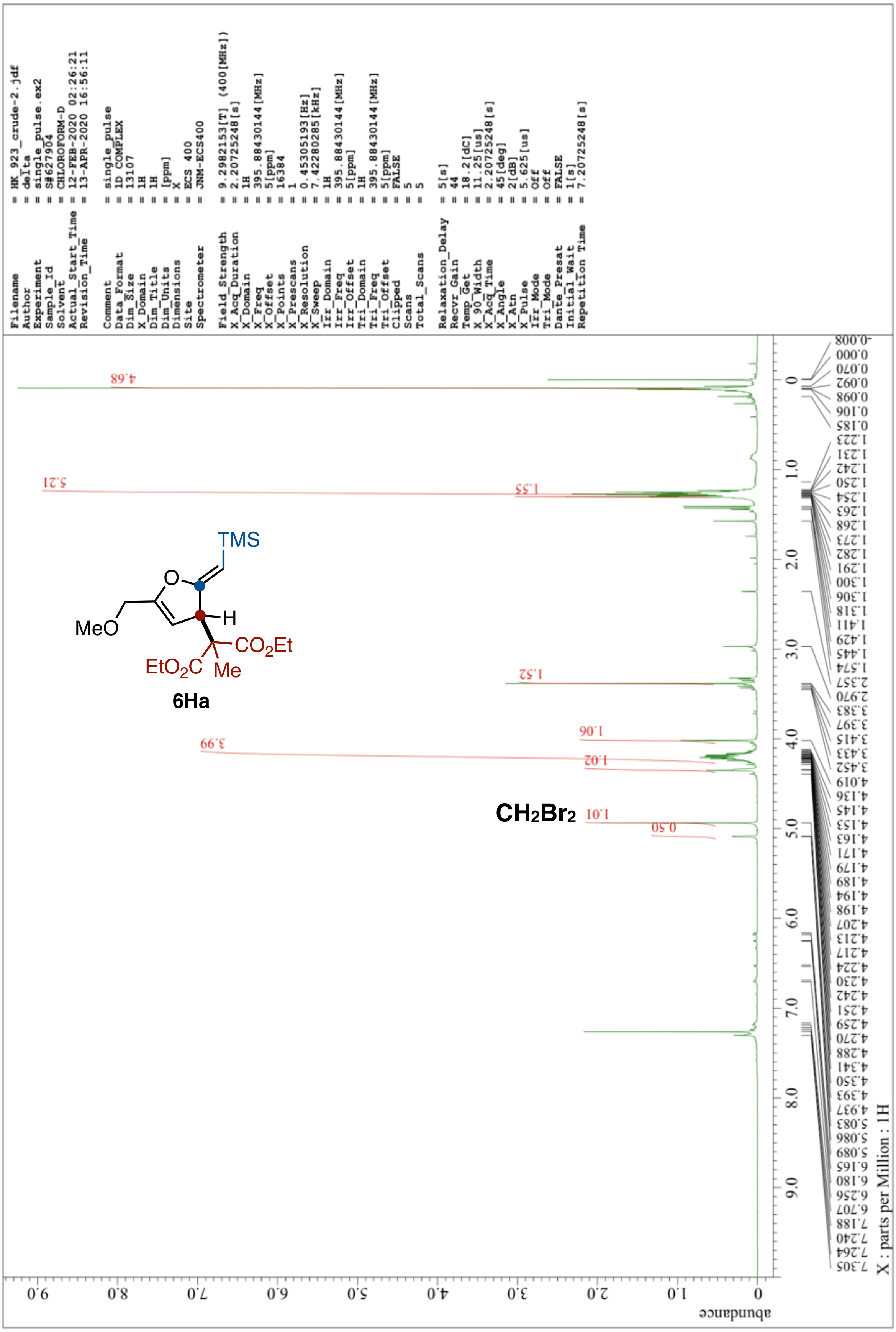

^{1}H NMR of **6Ia** (400 MHz, $CDCl_3$)

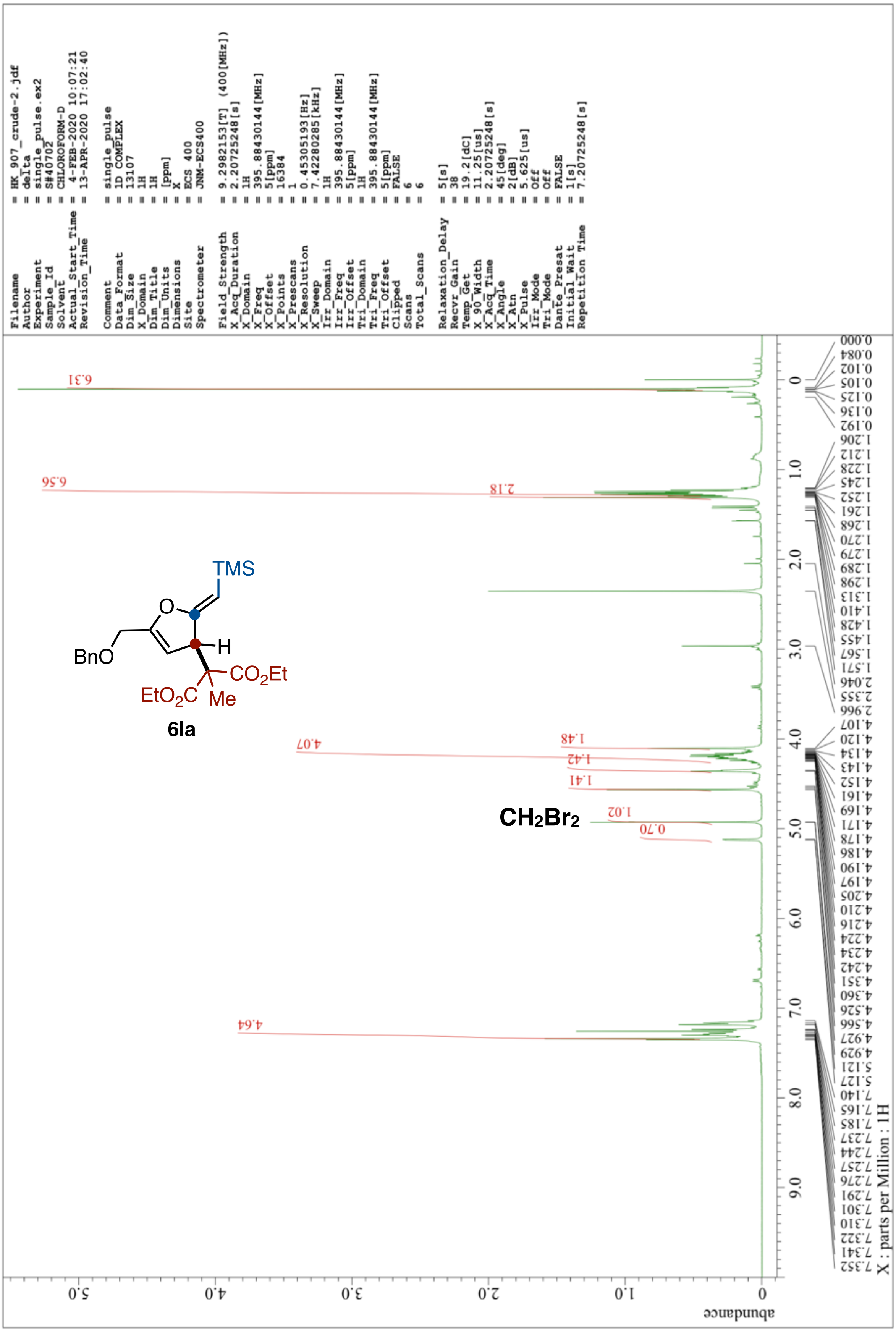

^{1}H NMR of **6Ja** (400 MHz, $CDCl_3$)

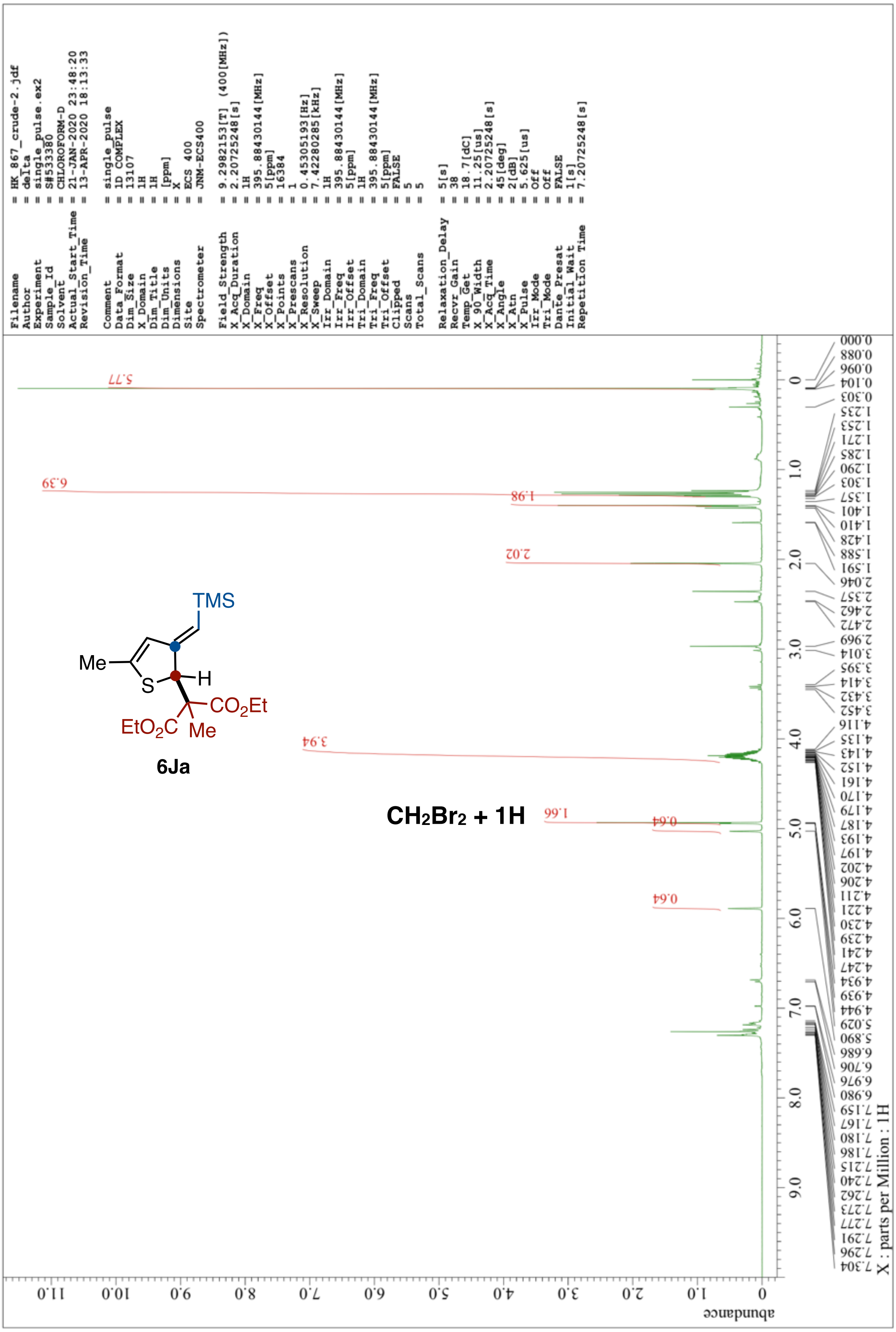

^{1}H NMR of **8Aa** (400 MHz, $CDCl_3$)

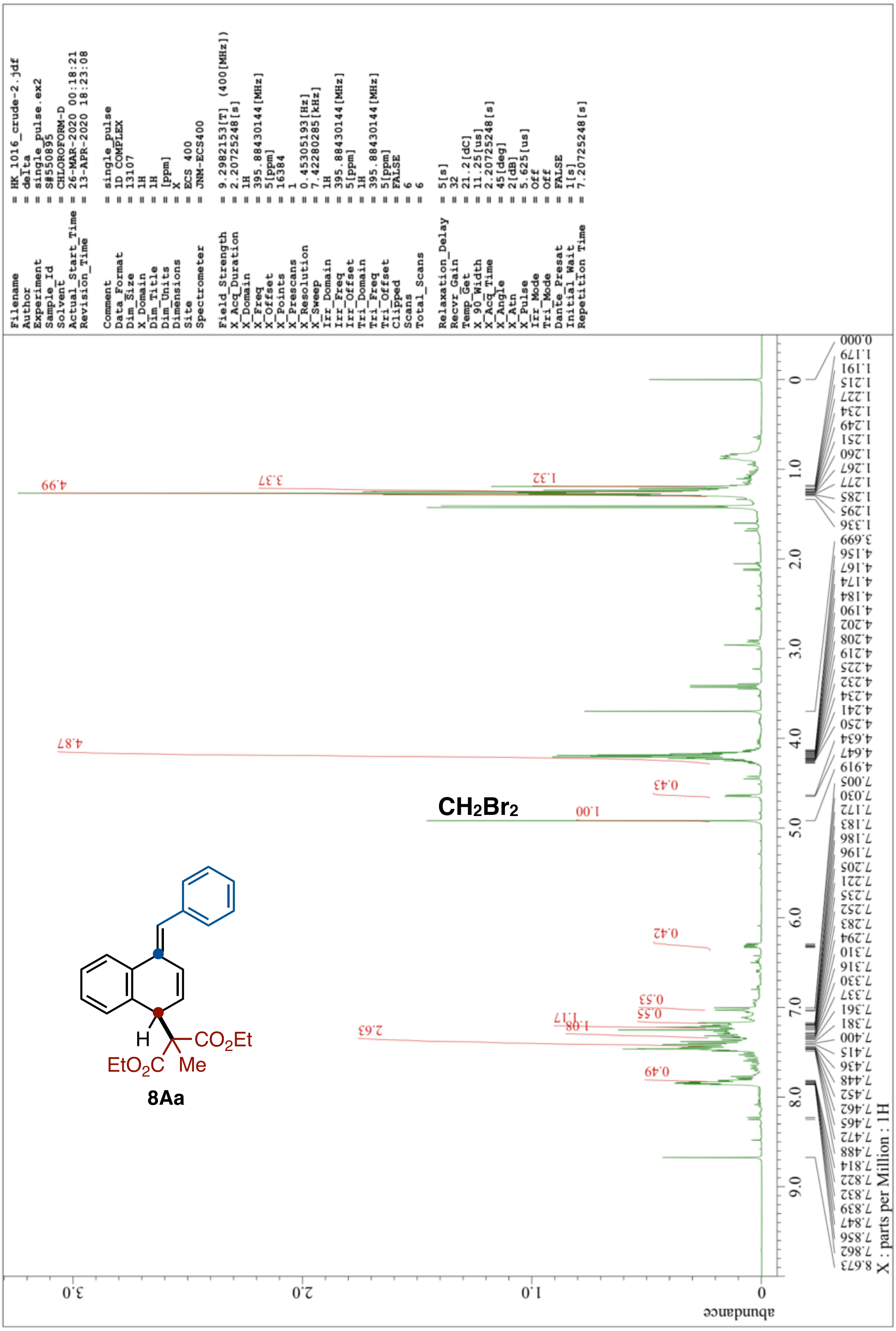

^{1}H NMR of **8Ab** (400 MHz, $CDCl_3$)

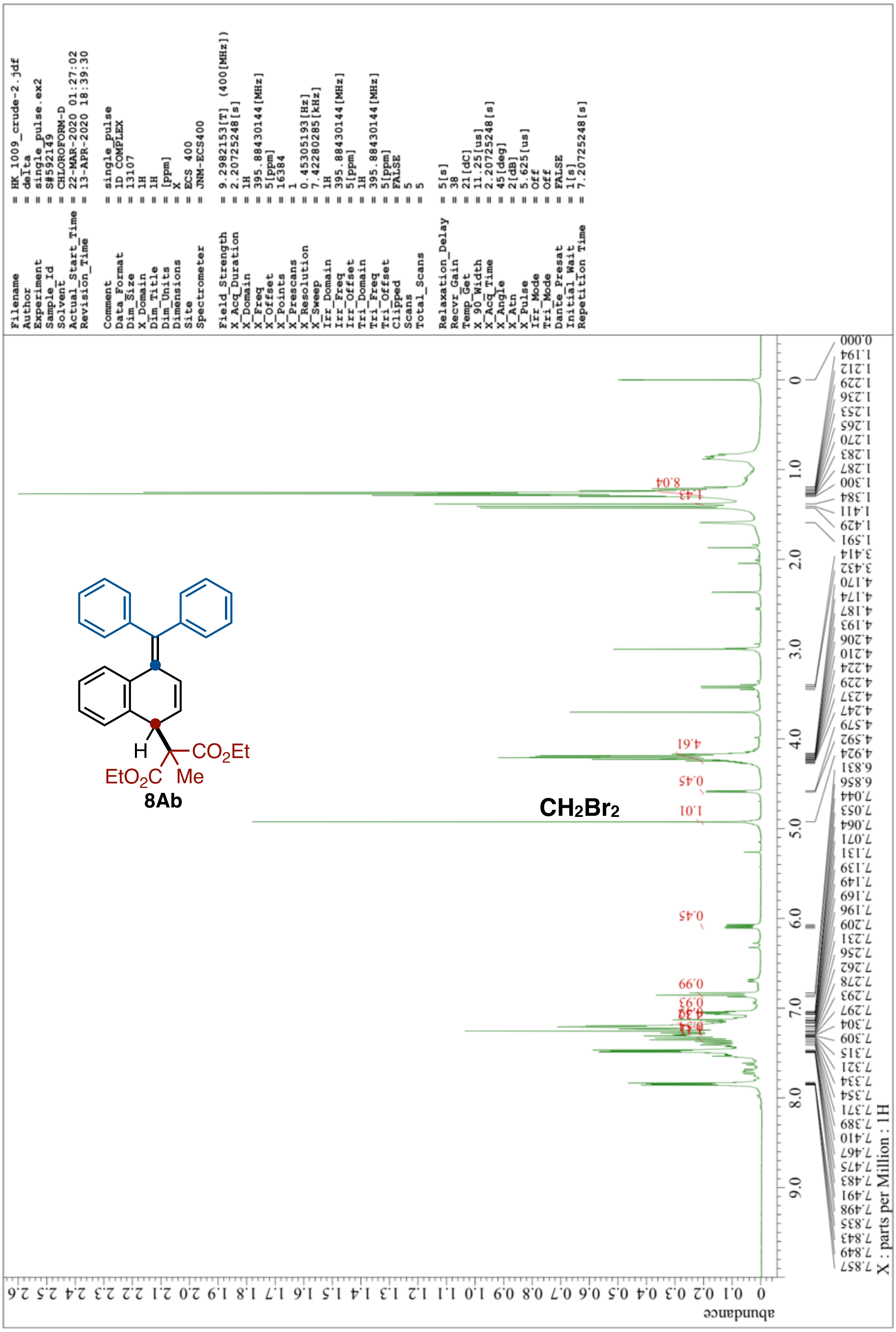

^{1}H NMR of **9Ac** (400 MHz, $CDCl_3$)

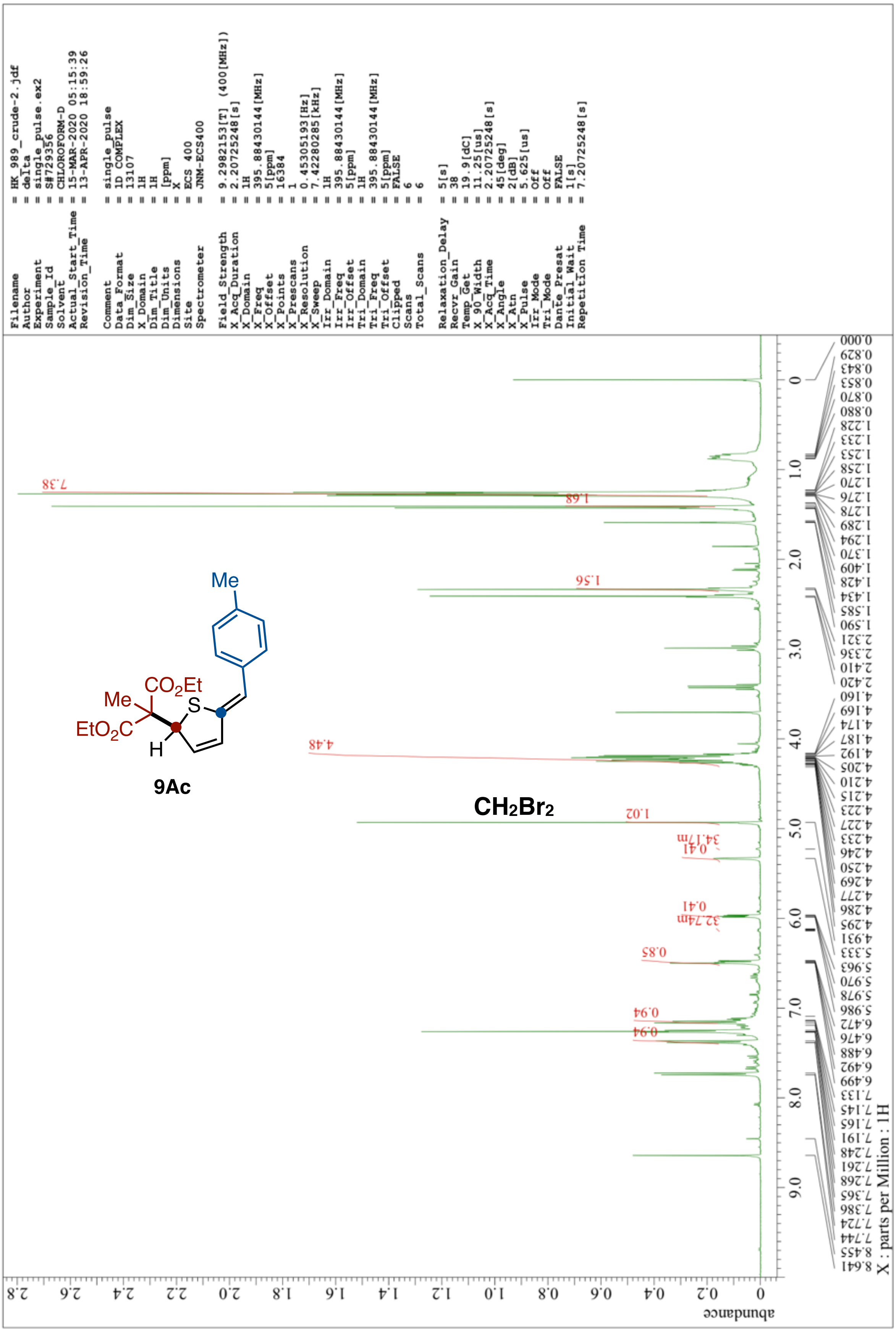

^{1}H NMR of **9Ad** (400 MHz, $CDCl_3$)

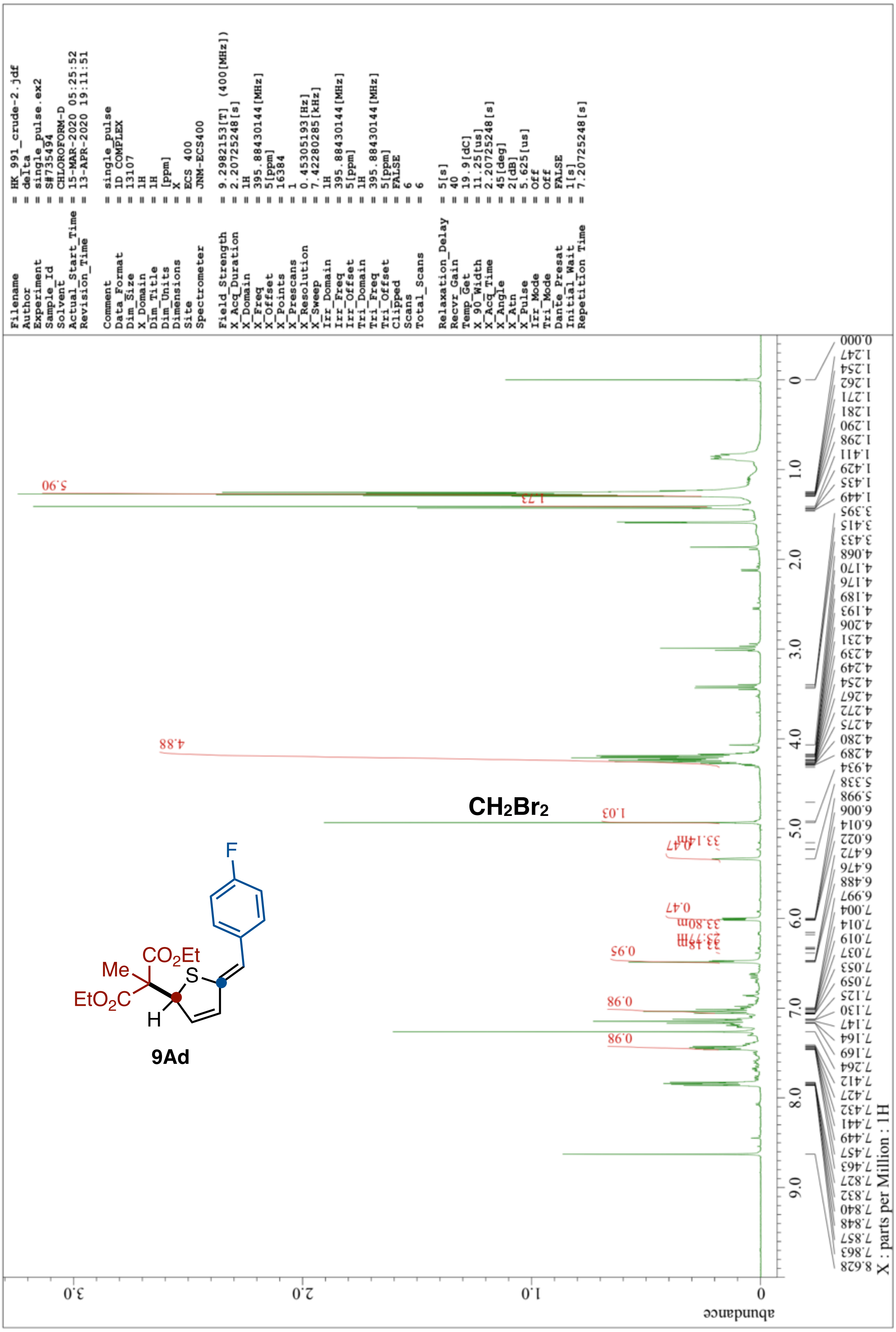

^{1}H NMR of **9Ab** (400 MHz, $CDCl_3$)

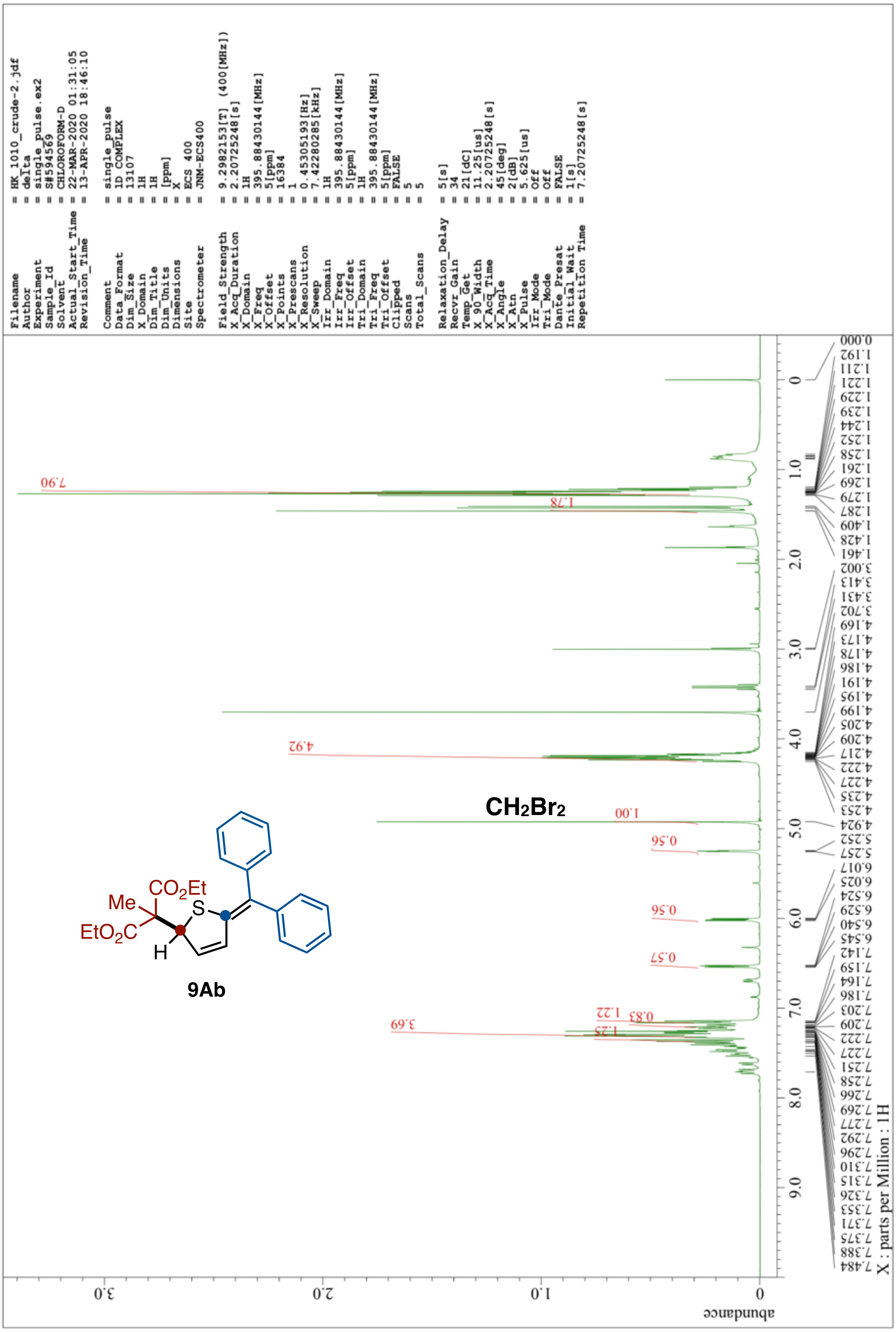

Supplement: SC-011-D0SC02881A-s001 [file SC-011-D0SC02881A-s001.pdf]
